# Copper-Catalyzed Diastereoselective Defluoroborylation of Pentafluoroethyl Alkenes Using (pin)B–B(dan)

**DOI:** 10.1021/acs.orglett.5c03558

**Published:** 2025-09-29

**Authors:** Yihan Tang, Gavin Chit Tsui

**Affiliations:** † Department of Chemistry, 26451 The Chinese University of Hong Kong, Shatin, New Territories, Hong Kong SAR, China; ‡ Shanghai-Hong Kong Joint Laboratory in Chemical Synthesis, 26451 The Chinese University of Hong Kong, Shatin, New Territories, Hong Kong SAR, China

## Abstract

We herein describe a highly diastereoselective Cu­(I)-catalyzed defluoroborylation of pentafluoroethyl alkenes using a diboron reagent (pin)­B–B­(dan). When 1,1- or 1,2-disubstituted pentafluoroethyl alkenes are employed as substrates, tetra- or trisubstituted fluoroalkenes can be obtained, respectively, in up to >99:1 diastereoselectivity. This protocol provides access to novel allyl–B­(dan) compounds and allylic alcohols with alkene moieties containing F and CF_3_ on the same carbon.

## Graphical abstract

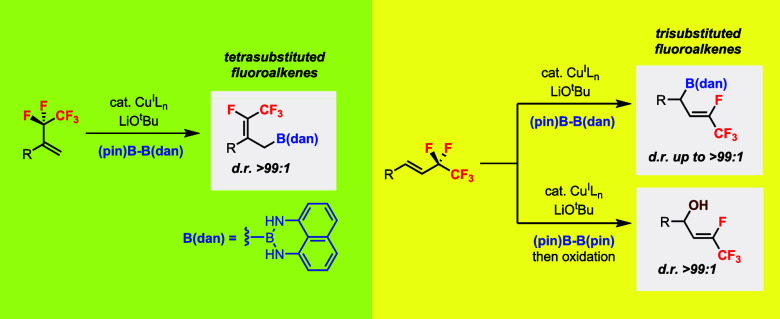

The selective functionalization of a carbon–fluorine bond^1^ in poly- or perfluorinated compounds can give access to partially fluorinated molecules that are difficult or even impossible to achieve by C–F bond formation.^2^ This is particularly important for obtaining structurally novel and previously unattainable fluorinated motifs with the advantage in patentability and therapeutic applications.^3,4^ Besides, some perfluorinated starting materials are less costly than their halogenated counterparts. The robust C–F bonds can withstand harsh reaction conditions and can be carried downstream in multistep synthetic sequences for late-stage functionalization. The intrinsically strong C–F bonds (BDE of ∼120 kcal/mol)^5^ have been a major hurdle in developing defluorinative functionalization^6^ methods. However, transition-metal-catalyzed C–F bond functionalization in recent years has dramatically broadened the reactivity profile.^7^

A well-studied application is the copper-catalyzed defluoroborylation of trifluoromethyl alkenes for generating *gem*-difluoroalkenes containing allyl boronate (Scheme 1a). In 2011, Hoveyda and co-workers reported an example of NHC/Cu­(I)-catalyzed defluoroborylation of α-(trifluoromethyl)­styrene (R_1_ = Ph and R_2_ = H) with B_2_(pin)_2_.^8^ Subsequently, asymmetric Cu-catalyzed defluoroborylation of 1-(trifluoromethyl)­alkenes (R_1_ = H and R_2_ = alkyl) was also developed by Ito’s^9^ and Shi’s^10^ groups independently. The reaction could be extended to CF_2_R-containing alkenes for the synthesis of monofluoroalkenes.^11,12^ Despite the progress of defluoroborylation reactions, the substrate scope is still largely limited to CF_3_-containing alkenes; other perfluoroalkyl alkenes were rarely explored. In 2022, Hoveyda and Liu et al. described an isolated example of Cu-catalyzed defluoroborylation of gaseous 3,3,4,4,4-pentafluorobut-1-ene (a pentafluoroethyl alkene) to generate the allyl–B­(pin) product (Scheme 1b), which was the key intermediate for the diastereo- and enantioselective synthesis of chiral homoallylic alcohols.^13^ Our group has a continuing interest in transition-metal-catalyzed C–F bond functionalization^14^ and the synthesis of pentafluoroethylated compounds.^15^ Previously, we have developed a Pd(0)-catalyzed diastereoselective defluoroborylation of *gem*-difluoroalkenes for the functionalization of a C­(sp^2^)–F bond (Scheme 1c).^16^ In this work, we describe a Cu­(I)-catalyzed defluoroborylation of pentafluoroethyl alkene **1** by activating a more challenging C­(sp^3^)–F bond (Scheme 1d). The reaction can proceed through the alkene insertion of the Cu–B bond followed by β-F elimination at the Cu center to generate allyl boronates **2**. Overall, an allylic C–F bond is cleaved and replaced with a C–B bond. Compounds **2** also belong to a novel class of functionalized fluoroalkenes where sp^2^ carbon is connected to both F and CF_3_, a hybrid of monofluoroalkene and trifluoromethyl alkene,^17^ with multiple substituent groups.

**Scheme 1:**
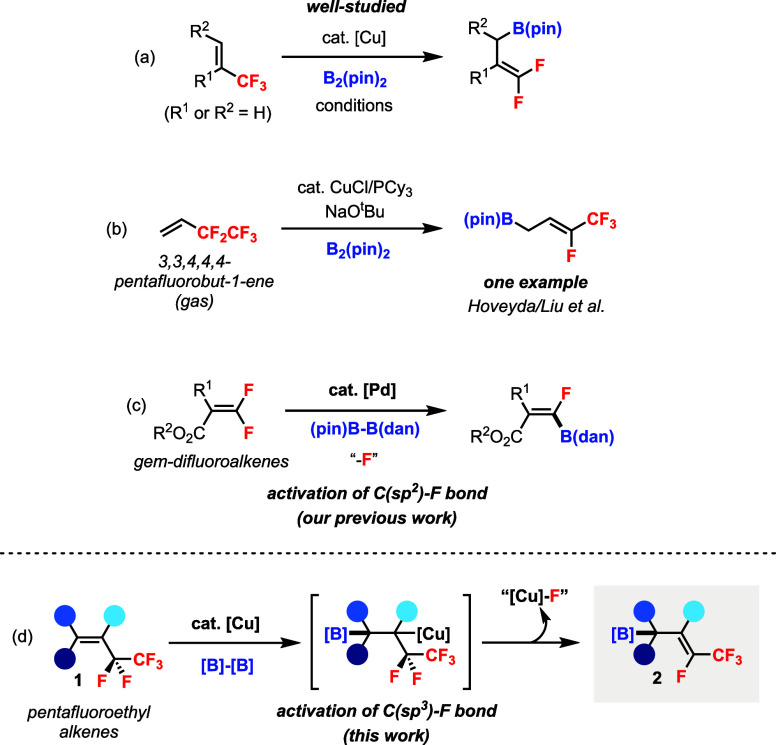
Copper-Catalyzed Defluoroborylation of Perfluoroalkyl Alkenes

One key difference between the defluoroborylation of pentafluoroethyl versus trifluoromethyl alkenes is that the products **2** can be (*E*) or (*Z*) isomers (cf. Scheme 1d vs Scheme 1a). For the control of diastereoselectivity by catalyst/ligand, substituent groups of **1** or the boron reagent was unpredictable at the outset. A 1,1-disubstituted pentafluoroethyl alkene **1a** was used as a standard substrate for condition optimization.^18^ The combination of CuCN/PCy_3_ was identified as an effective catalytic system using LiO^t^Bu as the base in THF at 40 °C to generate **2a** with (pin)­B–B­(dan) (Scheme 2). It was found that the diboron reagent had a dramatic effect on both the yield and diastereoselectivities. Using B_2_(pin)_2_ gave a moderate yield and d.r. (82:18), while even lower yields and d.r. values were observed with B_2_(cat)_2_ and B_2_(nep)_2_. On the other hand, the unsymmetrical diboron reagent (pin)­B–B­(dan) afforded the B­(dan) product **2a** in a quantitative yield and excellent d.r. as the (*Z*) product.

**Scheme 2:**
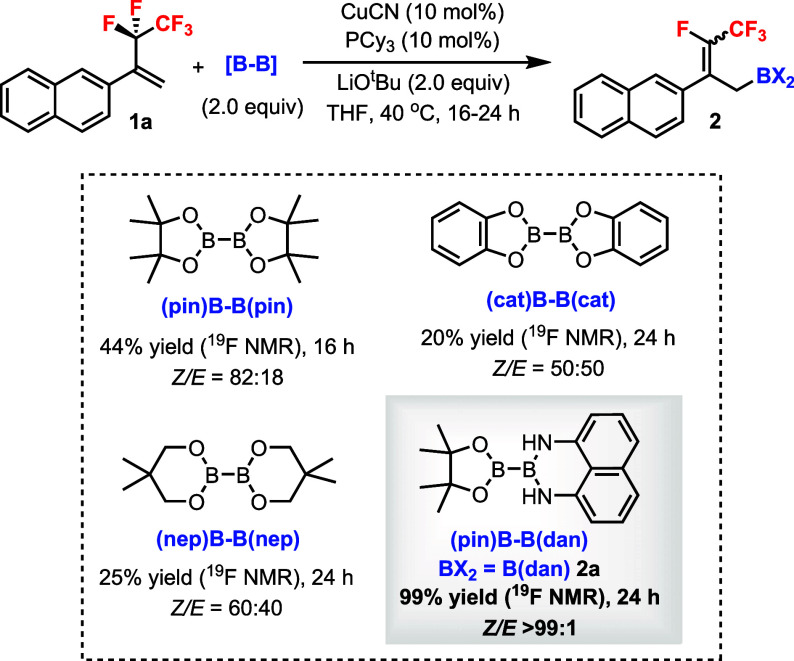
Effects of Diboron Reagents on Cu-Catalyzed Defluoroborylation of Pentafluoroethyl Alkene **1a**

The scope of the diastereoselective Cu-catalyzed defluoroborylation was subsequently explored using pentafluoroethyl alkenes **1** bearing various hetero­(aryl) substituent groups (Scheme 3). Products **2a** and **2b** could be isolated by column chromatography, even on 1.0 mmol scales. Electron-rich (**2c**) and -poor (**2d**) substituent groups as well as heteroatom groups, such as −SMe (**2e**), −Cl (**2f**), and −Br (**2g**), were tolerated on the benzene ring. Nitrile (**2h**) and a fused ring (**2i**) were compatible. Heteroaromatic groups, including benzofuran (**2j**), benzothiophene (**2k**), and quinoline (**2l**), were also tolerated, and a vinyl substituent group (**2m**) was demonstrated. In all cases, only the (*Z*) products were obtained in excellent diastereoselectivities (>99:1). The (*Z*)-alkene geometry was established by ^19^F–^1^H HOESY experiments of **2l**.^18^ Substrates containing alkyl substituents gave poor yields and diastereoselectivities under the same conditions.^18^

**Scheme 3:**
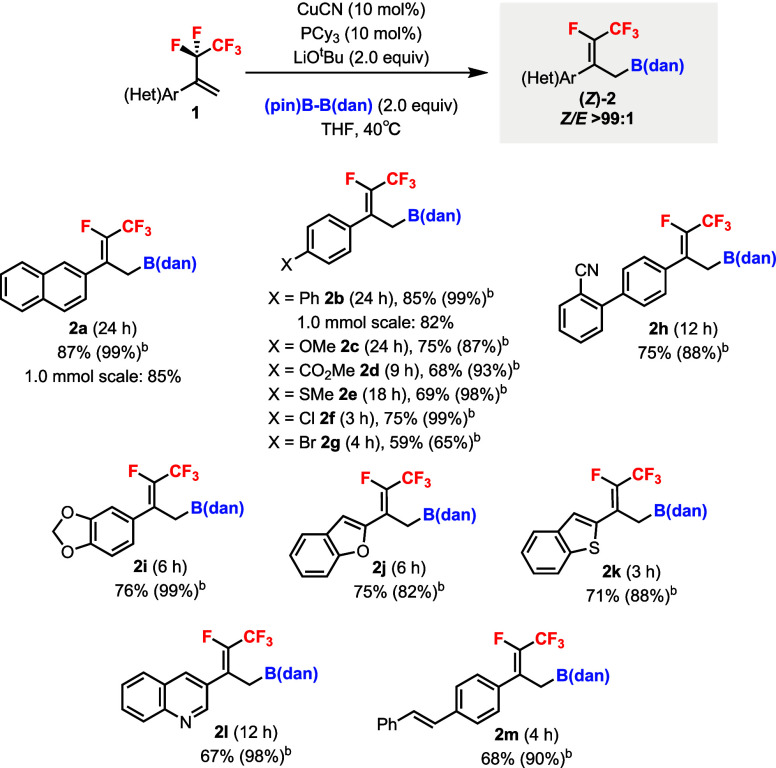
Diastereoselective Cu-Catalyzed Defluoroborylation of 1,1-Disubstituted Pentafluoroethyl Alkenes **1** Using (pin)­B–B­(dan)^a^

a Unless specified otherwise, reactions were carried out using **1** (0.2 mmol) under argon, with isolated yields. The *Z*/*E* ratios of **2** (>99:1) were determined by ^19^F NMR analysis of the isolated products.
b The yield was determined by ^19^F NMR analysis of the crude mixture using benzotrifluoride as an internal standard.

Next, 1,2-disubstituted pentafluoroethyl alkene **3a** was investigated in Cu-catalyzed defluoroborylation (Scheme 4). It was found during optimization that a NHC ligand could lead to the formation of product (*Z*)-**4a** in an excellent yield and diastereoselectivity.^18^ Intriguingly, using (*E*)-**3a** (*E*/*Z* > 99:1) or *E*/*Z* mixtures of **3a** (*E*/*Z* = 88:12 or 15:85) could afford (*Z*)-**4a** in comparable yields and excellent d.r. values (Scheme 4a). The (*Z*)-alkene geometry of **4a** was confirmed by ^19^F–^1^H HOESY experiments.^18^ This stereoconvergent phenomenon was advantageous since pentafluoroethyl alkenes, such as **3a**, could be conveniently obtained from readily available unactivated alkenes in one step as *E*/*Z* mixtures (Scheme 4b).^19^ Other examples were also demonstrated under the standard conditions to synthesize compounds (*Z*)-**4b**–**4e** in 95:5 to >99:1 d.r. values (Scheme 4c). The reaction could be performed on a 1.0 mmol scale (**4d**). Moreover, substrate **5** with a C_4_F_9_ group gave product (*Z*)-**6** containing F- and C_3_F_7_-substituted alkene in >99:1 d.r. Density functional theory (DFT) calculations were conducted to rationalize the stereoconvergent formation of (*Z*)-**4a** (Scheme 4d).^18^ The migratory insertion of L–Cu–B­(dan) into (*E*)-**3a** or (*Z*)-**3a** leads to intermediate **INT-A** or **INT-B**, respectively. The formation of product **4a** from these two intermediates by β-F elimination can take places via four transition states **TS-1**–**TS-4**. It was found that **TS-2** and **TS-3** had lower energy barriers, both of which give rise to the (*Z*) product. However, switching to a styrene-type substrate **7** did not afford the desired defluoroborylation product; instead, a hydrodefluorination (HDF) product **8** was detected (eq 1). This HDF process could be optimized by using B_2_(pin)_2_ and MeOH to give (*E*)-**8** in a good yield and excellent d.r. (eq 2).^20^

**Scheme 4:**
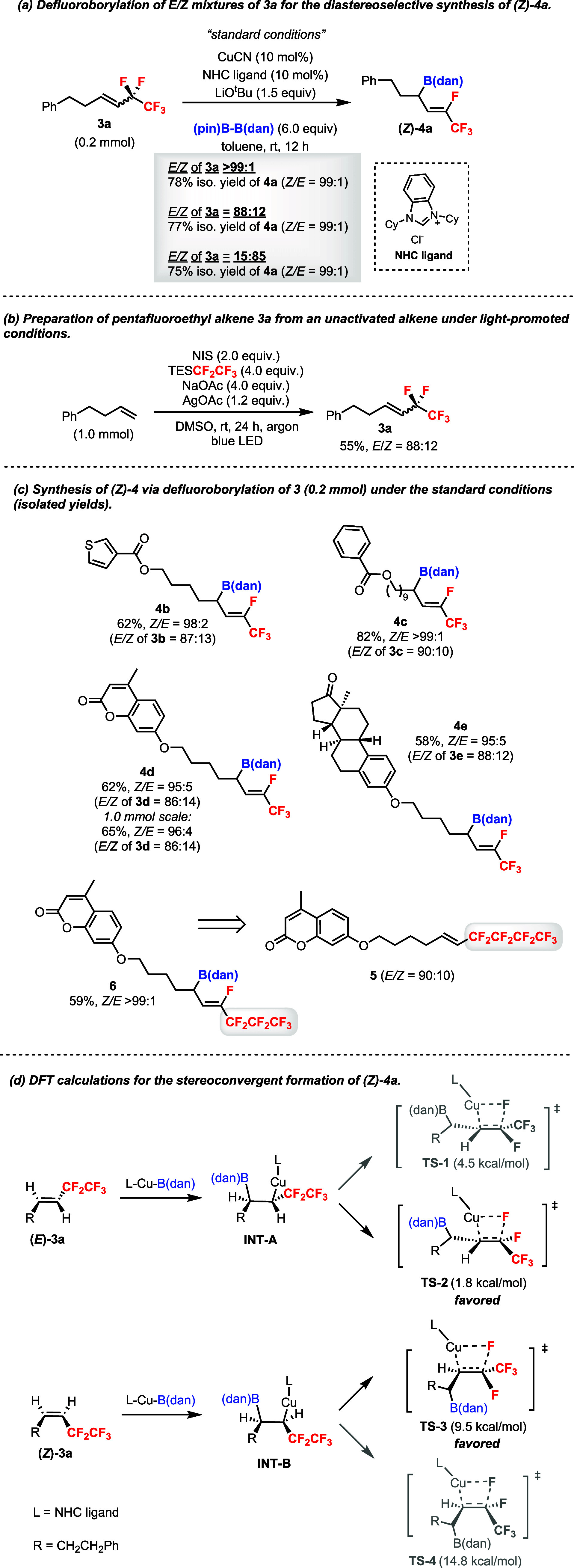
Diastereoselective Cu-Catalyzed Defluoroborylation of 1,2-Disubstituted Pentafluoroethyl Alkene **3**



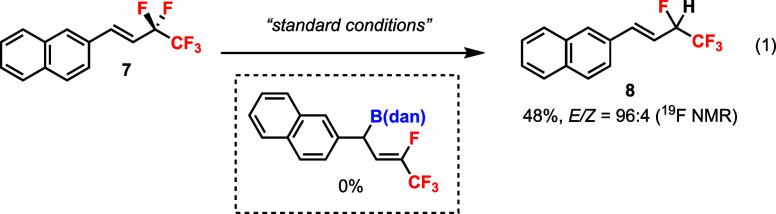

1




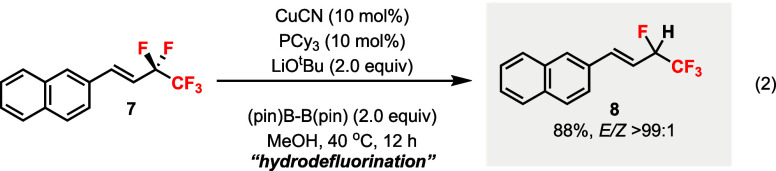

2


The defluoroborylation of **3** was equally efficient and selective with B_2_(pin)_2_ and (pin)­B–B­(dan) (see optimization tables in the Supporting Information),^18^ which was different from using substrates **1**. The B­(dan) products **4** were more stable than the B­(pin) products, which could be isolated by column chromatography; however, conversion of **4** to alcohols by oxidation was difficult due to side product formation and low yield (ca. 10%) of the desired alcohol product. We were able to develop a two-step protocol to synthesize useful allylic alcohols **9** from **3** via oxidation of the B­(pin) intermediate without isolating it (Scheme 5). The alcohols **9a**–**9d** with different substituents were successfully obtained in excellent diastereoselectivities as the (*Z*) isomers only. Moreover, the F- and C_2_F_5_-substituted product **11** could be prepared from substrate **10** with a C_3_F_7_ group.

**Scheme 5:**
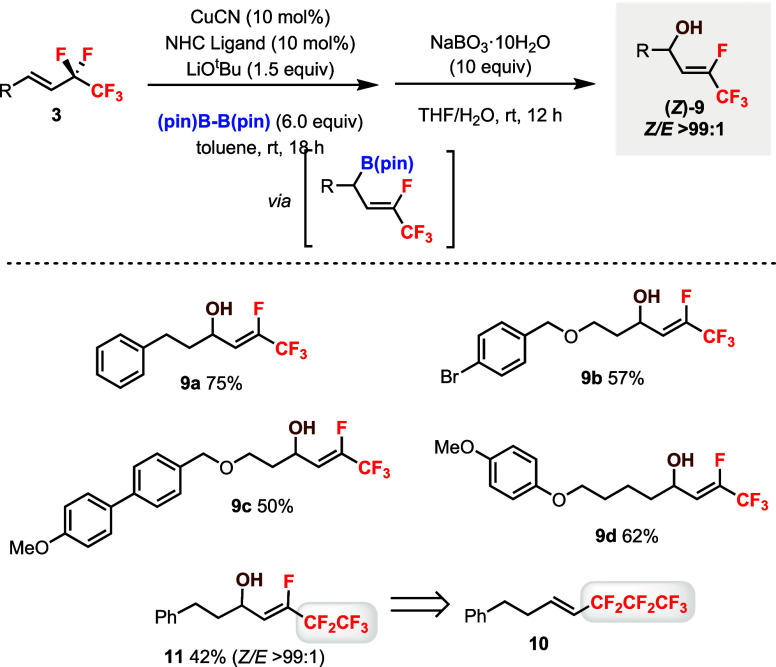
Diastereoselective Synthesis of Allylic Alcohols (*Z*)-**9** from Pentafluoroethyl Alkenes **3** ^a^

a Unless specified otherwise, reactions were carried out using **3** (0.2 mmol) under argon, with isolated yields over two steps. The *Z*/*E* ratios of **9**/**11** (>99:1) were determined by ^19^F NMR analysis of the isolated products.

Chiral ligands were screened based on the standard conditions in order to achieve the enantioselective defluoroborylation of (*E*)-**3a**.^18^ Preliminary results showed that (*R*)-DM-SEGPHOS was effective for reactivity and selectivity. Product (*Z*)-**4a** could be obtained in 75% yield with >99:1 diastereoselectivity and 93:7 enantioselectivity (Scheme 6). However, other substrates afforded the products (*Z*)-**4f** and (*Z*)-**4g** in lower yields and enantioselectivities.

**Scheme 6:**
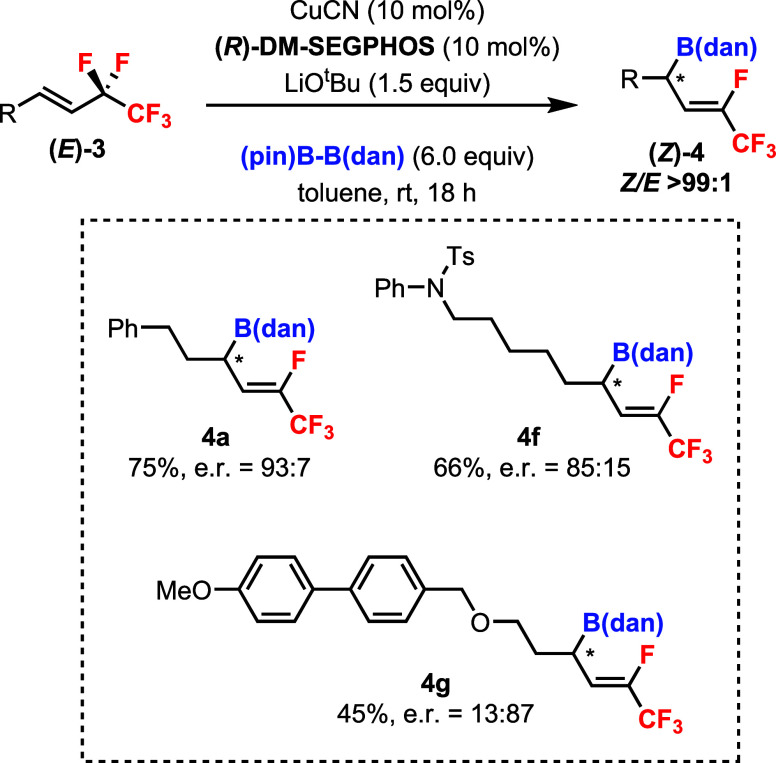
Preliminary Studies on the Enantioselective Defluoroborylation of **3**

In summary, we have developed a highly diastereoselective Cu­(I)-catalyzed defluoroborylation of pentafluoroethyl alkenes using the (pin)­B–B­(dan) reagent. A new class of allyl–B­(dan) compounds **2**/**4** can be synthesized with alkene moieties bearing F and CF_3_ on the same carbon. Starting from 1,1- or 1,2-disubstituted pentafluoroethyl alkenes, tetra- or trisubstituted fluoroalkenes were obtained, respectively, in up to >99:1 diastereoselectivity. Allylic alcohols could also be prepared in two steps from pentafluoroethyl alkenes using B_2_(pin)_2_. DFT calculations^18^ were carried out to decipher the reaction mechanism via (1) transmetalation between the Cu­(I) catalyst and (pin)­B–B­(dan) to generate a Cu–B­(dan) species, (2) migratory insertion of Cu–B­(dan) to the double bond of pentafluoroethyl alkene **1**/**3**, and (3) β-F elimination (stereodetermining step) at the Cu center to generate the borylated product **2**/**4** and Cu–F. Further optimization for the asymmetric version of this reaction and transformation of the borylated products are ongoing in our laboratory.

## Supplementary Material



## Data Availability

The data underlying this study are available in the published article and its Supporting Information.

## Notes

Experimental procedures, optimization data, characterization data, spectral data, and DFT calculations (PDF)
