# Supplementary material for: Copper-Catalyzed Diastereoselective Defluoroborylation of Pentafluoroethyl Alkenes Using (pin)B–B(dan)
Source: Org Lett. 2025 Sep 29;27(40):11273–7. doi: 10.1021/acs.orglett.5c03558 (PMC12519480; doi:10.1021/acs.orglett.5c03558)
Supplement: Supplementary file 1 [file ol5c03558_si_001.pdf]

Supporting Information

Copper-Catalyzed Diastereoselective Defluoroborylation of Pentafluoroethyl Alkenes Using (pin)B-B(dan)

Yihan Tang [a] *and Gavin Chit Tsui* [a,b]*

[a]Department of Chemistry, The Chinese University of Hong Kong, Shatin, New Territories, Hong Kong SAR, China

[b]Shanghai-Hong Kong Joint Laboratory in Chemical Synthesis, The Chinese University of Hong Kong, Shatin, New Territories, Hong Kong SAR, China

*Email: gctsui@cuhk.edu.hk

Experimental Procedures and Spectral Data

Table of Contents:

1. General Experimental **S1**
2. Materials **S1**
3. Instrumentation **S1**
4. Experimental Procedures **S2**
5. Optimization Studies **S11**
6. ^{1}H-^{19}F HOESY NMR Studies **S17**
7. Computational studies **S18**
8. Characterizations **S19**
9. References **S39**
10. Spectrum **S41**
11. Coordinations of DFT calculations **S124**

1. General Experimental

Unless otherwise noted, reactions were carried out in a 10 mL glass tube with magnetic stirring. Reactions that require heating were carried out in the oil bath. Analytical thin layer chromatography (TLC) was performed with Merck silica gel 60 F_{254} aluminum plates. Visualization was done under a UV lamp (254 nm) and by immersion in potassium permanganate ($KMnO_4$), followed by heating using a heat gun. Organic solutions were concentrated by rotary evaporation at 23-35 °C. Purification of reaction products were generally done by flash column chromatography with Silicycle 60-230 mesh silica gel.

2. Materials

Anhydrous LiO^tBu was purchased from Aladdin. CuCN was purchased from J&K. PCy_3 was purchased from Acros. The NHC ligands and chiral ligands were purchased from Aldrich. Pentafluoroalkenes and $Et_3SiC_2F_5$ were prepared according to literature procedure. Other chemicals for substrates preparation were purchased from Acros, J&K Scientific, Aldrich and Dieckmann.

3. Instrumentation

Proton nuclear magnetic resonance spectra (^1H NMR), carbon nuclear magnetic resonance spectra (^{13}C NMR) and fluorine nuclear magnetic resonance spectra (^{19}F NMR) were recorded at 23 °C on Bruker 400 MHz or 500 MHz spectrometer in $CDCl_3$. Chemical shifts of ^1H NMR spectra were reported as parts per million in δ scale using residual solvent signal ($CDCl_3$: 7.26 ppm) or tetramethylsilane (0.00 ppm) as internal standard. Chemical shifts of ^{13}C NMR spectra were reported using residual solvent signal of $CDCl_3$ (77.16 ppm) on the δ scale. Chemical shifts of ^{19}F NMR were reported as parts per million in δ scale using benzotrifluoride (-63.72 ppm) as internal standard. Data are represented as follows: chemical shift (δ ppm), multiplicity (s = singlet, d = doublet, t = triplet, q = quartet, qd = quartet of doublet, m = multiplet), coupling constant (J, Hz) and integration. GC-MS analysis results were obtained on a Shimadzu GCMSQP2010 SE GC-MS Spectrometer. High resolution mass spectra (HRMS) were obtained on a Finnigan MAT 95XL GC Mass Spectrometer or a Thermo Scientific Q Exactive Focus Mass Spectrometer or a Bruker SolariX 9.4T FTMS (mass analyzer type: orbitrap).

4. Experimental Procedures

Substrate 1

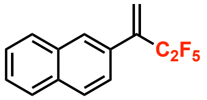

1a

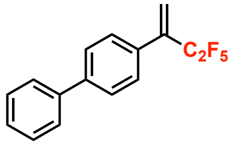

1b

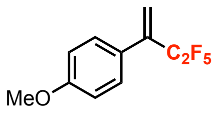

1c

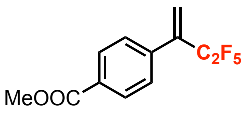

1d

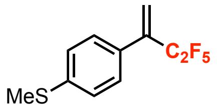

1e

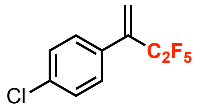

1f

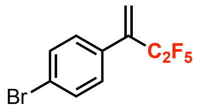

1g

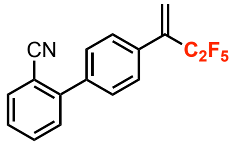

1h

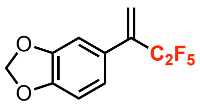

1i

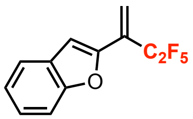

1j

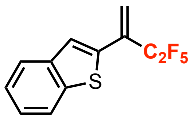

1k

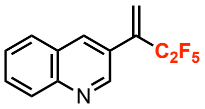

1l

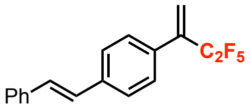

1m

Substrate 1a – 1m can be synthesized according to literature method. [1]

Substrate 3, 9 and 10

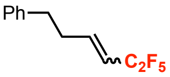
3a

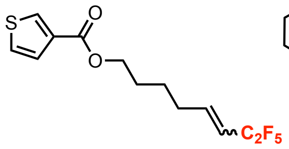
3b

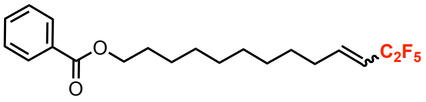
3c

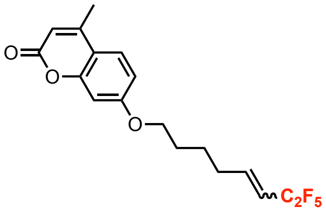
3d

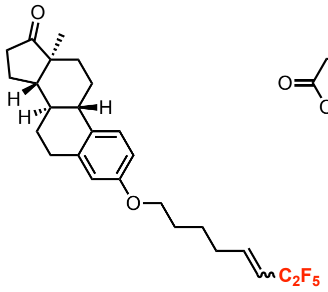
3e

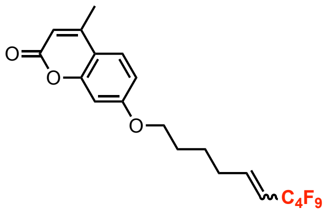
5

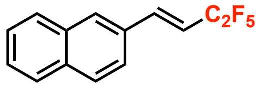
7

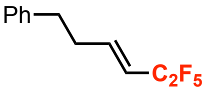
S9a

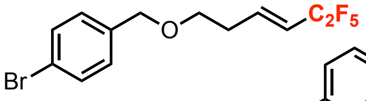
S9b

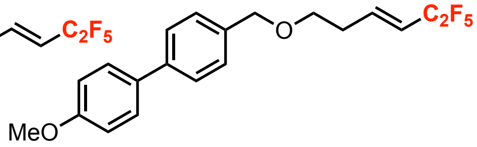
S9c

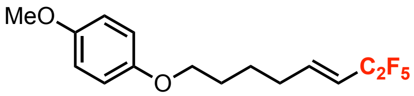
S9d

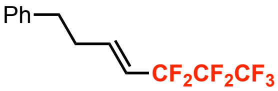
10

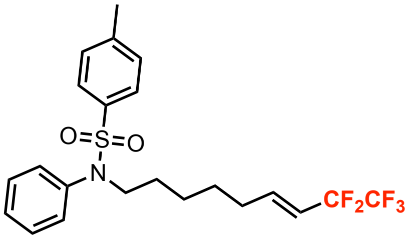
3f

Substrate 3a – 3e, 3g, 5, 7, S9a – S9d and 10 can be synthesized according to literature method. [2-4]

General Procedure (I) for the synthesis of $Et_3SiCF_2CF_3$.

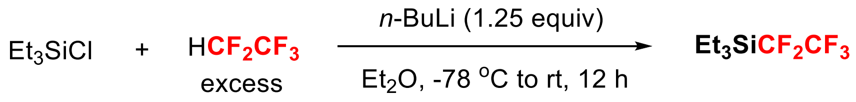

$Et_3SiCF_2CF_3$ was synthesized according to the literature procedure.[2] To a round bottom flask, 250 ml diethyl ether was cooled down to -78 °C. Pentafluoroethane was bubbled into diethyl ether for 3 hours followed by adding a solution of *n*-BuLi (100 ml, 2.5 M in hexane, 250 mmol, 1.25 equiv) carefully so that the temperature of the reaction system didn't exceed -60 °C. After stirring for 1 h, the solution of triethylchlorosilane (21.72 g, 200 mmol, 1 equiv) in diethyl ether was added within 5 min. The mixture was allowed to warm up to room temperature slowly for 12 h. The mixture was filtered through a pad of Celite and concentrated to dryness to afford the crude product. The product was purified by distillation in vacuo and obtained a colorless liquid (33.7 g, 72%). The purity of the product was determined by ^1H NMR and ^{19}F NMR. The spectra are in full accordance with the literature report.

General procedure (II) for the synthesis of pentafluoroethyl ketones S1:

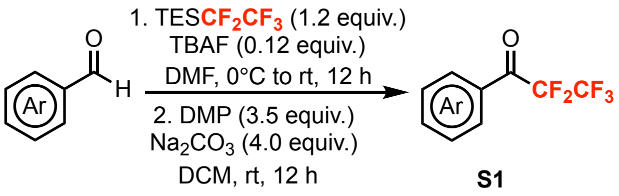

Pentafluoroethyl ketones S1 was synthesized according to literature procedure with minor changes.[1] To an oven-dried 50 mL round-bottom flask equipped with a stir bar, the solution of aldehyde (10 mmol, 1 equiv) in DMF (10 mL, 1.0 M) was added under argon, $TESCF_2CF_3$ (2.8 g, 12 mmol, 1.2 equiv) was added and the mixture was stirred in an ice bath. After approximately 10 min, TBAF (1.0 M in THF, 1.2 mmol, 0.12 equiv) was added through syringe. After 10 min, the ice bath was removed, and the solution was stirred for approximately 12 h at room temperature. After completion of the reaction (monitored by TLC), the mixture was extracted with Et_2O (20 mL × 3), washed with brine and then dried over anhydrous Na_2SO_4. After filtration and evaporation under vacuum, the residue was directly used for the next step without purification.

To a solution of the α-CF_2CF_3 alcohol (10 mmol, 1 equiv) in DCM (60 mL, 0.16 M) in a round bottom flask, Dess-Martin periodinane (14.8 g, 35 mmol, 3.5 equiv) and Na_2CO_3 (4.2 g, 40 mmol, 4 equiv) was added. The solution was stirred at room temperature for 12 h. Then water was added, and the obtained suspension was stirred for an additional 30 min, the mixture was extracted with DCM (60 mL × 3). The organic phase was washed with brine and then dried over anhydrous Na_2SO_4. The solvent was removed under vacuo by rotary evaporation to give the α-CF_2CF_3 ketones S1.

General procedure (III) for the synthesis of pentafluoroalkenes 1:

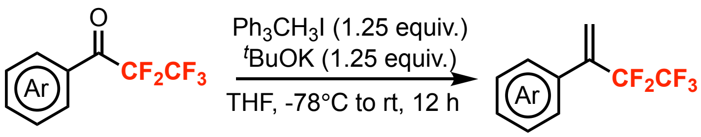

S1 **1**

To an oven-dried round bottom flask was added methyltriphenylphosphonium iodide (2.0 g, 5 mmol, 1.25 equiv) or methyltriphenylphosphonium bromide (1.8 g, 5 mmol, 1.25 equiv) and ^{t}BuOK (562 mg, 5.0 mmol, 1.25 equiv) in THF (10 mL) under argon. The mixture was stirred at room temperature for 30 mins and cooled to -78 °C, then α-CF_2CF_3 ketones (4 mmol, 1 equiv) in THF (5 mL) was added under -78 °C. The reaction mixture was allowed to warm up to room temperature with vigorously stirring over 12 h. Later the reaction was quenched with water (50 mL) and extracted with DCM (15 mL × 3). The organic layer was dried over anhydrous Na_2SO_4 and concentrated in vacuo. The residue was purified by flash column chromatography to afford the corresponding alkenes **1**. Substrate **1a – 1g**, **1i – 1l** were synthesized from this method.

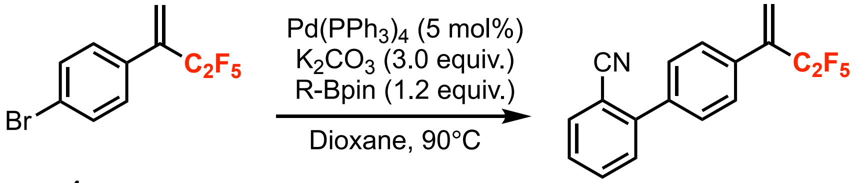

1g **1h**

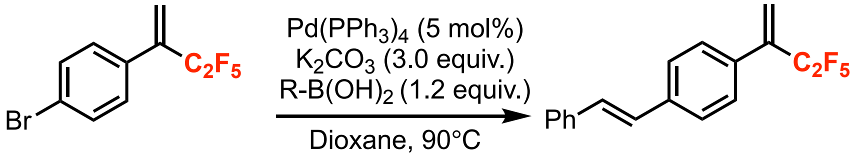

1g **1m**

1h and **1m** can be synthesized from the Suzuki cross-coupling of **1g**.

To an oven-dried glass tube equipped with a stir bar was added $Pd(PPh_3)_4$ (0.025 mmol, 5 mol%), corresponding boronic acid (0.6 mmol, 1.2 equiv.) and K_2CO_3 (15 mmol, 3.0 equiv.). The tube was sealed with a septum, evacuated and refilled with argon three times. A solution of **1g** (0.5 mmol) in 5.0 mL dioxane (0.1 M) was added under argon through syringe. The resulting mixture was heated at 90 °C with stirring in an oil bath for 12 h. After cooling to room temperature, the reaction mixture was extracted with CH_2Cl_2 (3 × 20 mL). The combined organic layers were washed with H_2O (2 × 10 mL), then brine (2 ×20 mL), dried over $MgSO_4$ and concentrated in vacuo. The residue was purified by flash column chromatography on silica gel to afford product **1h** or **1m.**

General Procedure (IV) for synthesis of (*E*)-alkenyl iodide S3

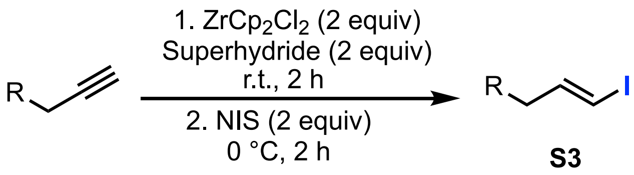

Alkenyl iodides **S3** were synthesized according to the literature procedure.[1] A round bottom flask charged with $ZrCp_2Cl_2$ (2.9 g, 10 mmol, 2 equiv) was added THF (10 mL). The superhydried solution (1.0 M in THF, 10 mmol, 2 equiv) was added. The solution was stirred in the dark for 2 h before a solution of alkyne (5 mmol, 1 equiv) in THF (10 mL) was added. The reaction was stirred for 15 min and cooled to 0°C, then the NIS (2.3 g, 10 mmol, 2 equiv) in THF(10 mL) solution was added. The reaction was stirred for 2 h and then quenched by NH_4Cl solution. The reaction mixture was extracted with Et_2O (3 × 30 ml). The combined organic layers were washed with brine, dried over anhydrous Na_2SO_4 and concentrated in vacuo. The residue was purified by flash column chromatography on silica gel to afford the corresponding alkenyl iodides **S3**.

General Procedure (V) for synthesis of (*E*)-pentafluoroethyl alkenes 3

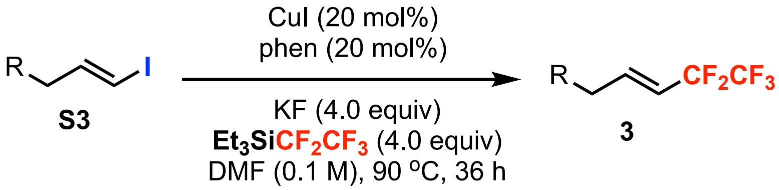

Pentafluoroethyl alkenes **3** was synthesized according to the literature procedure.[2] To an oven-dried 25 ml round bottom flask equipped with a stir bar was added alkenyl iodide (1.0 mmol, if it was a solid substrate), CuI (0.20 mmol, 0.2 equiv), 1,10-Phenanthroline (0.20 mmol, 0.2 equiv) and Potassium fluoride (4.0 mmol, 4.0 equiv) in the glovebox. The reaction vial was sealed with a rubber septum and removed from the glovebox. Pentafluoroethyl triethylsilane (4.0 mmol, 4.0 equiv), alkenyl iodide (if it was a liquid substrate) and DMF (10.0 ml, 0.1 M) was then added to the vial through the syringe under argon. The resulting mixture was stirred at 90°C for 36 hours. After cooling to room temperature, the crude sample was analyzed by ^{19}F NMR using benzotrifluoride (12 µL, 0.1 mmol) as internal standard. The reaction mixture was extracted with CH_2Cl_2 (3 × 50 ml). The combined organic layers were washed with H_2O (2 × 50 ml), then brine (2 × 250 ml), dried over $MgSO_4$ and concentrated in vacuo. The residue was purified by flash column chromatography on silica gel to afford pentafluoroethyl alkenes. Substrate **S9a – S9d** were synthesized from this method.

General procedure (VI) for the synthesis of heptafluoropropyl alkenes S10:

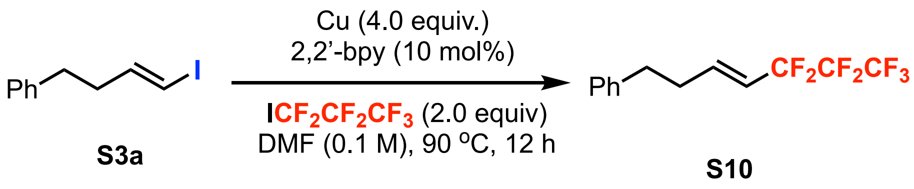

To an oven-dried round bottom flask equipped with a stir bar, the alkenyl iodide (1.4 mmol, 1.0 equiv), Cu (356 mg, 5.6 mmol, 4.0 equiv) and 2,2'-Bipyridine (22 mg, 0.14 mmol, 0.1 equiv) were added in the glovebox. The round bottom flask was sealed with a rubber septum and removed from the glovebox. C_3F_7I (828 mg, 2.8 mmol, 2.0 equiv) and DMSO (20 mL, 0.14 M) were then added to the flask through syringe under argon. The resulting mixture was stirred at 90°C for 12 h. After cooling to room temperature, the mixture was diluted by Et_2O and filtered through a pad of Celite. The combined organic layers were washed with H_2O (2 × 30 ml), then brine (2 × 30 ml), dried over anhydrous Na_2SO_4 and concentrated in vacuo. The residue was purified by flash column chromatography on silica gel to afford the corresponding heptafluoropropyl alkenes **S10** (336 mg, 1.1 mmol, 80% iso).

General Procedure (VII) for synthesis of pentafluoroethyl alkene *E*/*Z* mixtures 3

The pentafluoroethyl alkene *E*/*Z* mixtures were synthesized by modified method from literature.[3]

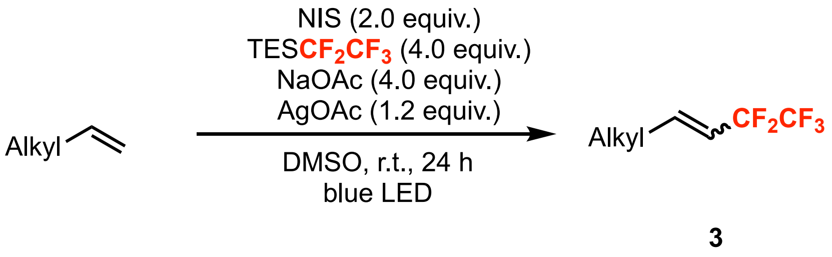

To an oven-dried 50 ml round bottom flask equipped with a magnetic stir bar were added alkene S3.1 (1.0 mmol), NIS (2.0 mmol, 2.0 equiv), NaOAc (4.0 mmol, 4.0 equiv) and AgOAc (1.2 mmol, 1.2 equiv). The tube was then sealed with a septum, evacuated and refilled with argon for three times. Anhydrous DMSO (15.0 mL, 0.06 M) was added via a syringe and the solution was further evacuated and refilled with argon for three times. Finally, $TESCF_2CF_3$ (4.0 mmol, 4.0 equiv) was added dropwise to the solution. The flask was sealed and placed under a 50 W blue LED (Kessil PhotoReaction PR160L-456-UK, 456 nm, Average Intensity of PR160 series 399mW/cm^2 (measured from 1 cm distance)) at room temperature and stirred for 24 h. The reaction was set up as following figure S1. The E/Z ratio was determined by ^{19}F NMR of the crude mixture. The reaction was quenched with H_2O, extracted with diethyl ether for three times. The organic layers were combined, washed with brine, dried over anhydrous Na_2SO_4, filtered and concentrated by rotary evaporator. The crude product was purified by flash column chromatography on. silica gel to afford the desired product.

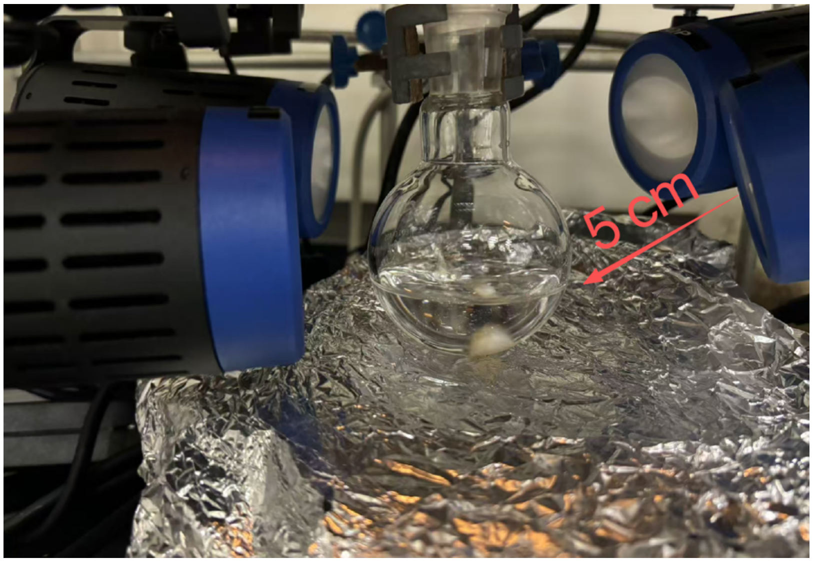

Figure S1. Light-promoted reaction equipment set up

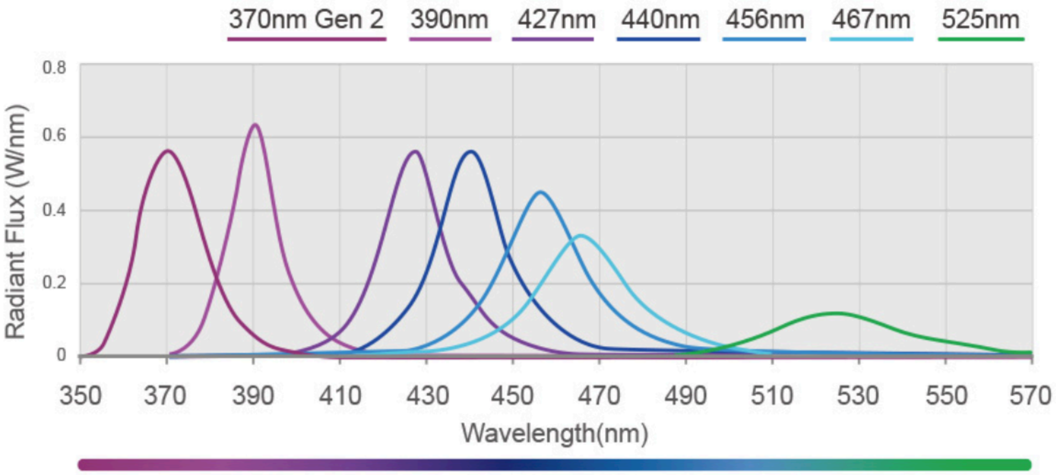

Figure S2. Kessil light spectrum

General procedure (VIII) for the defluoroborylation of alkene 1 (*c.f.* Scheme 3):

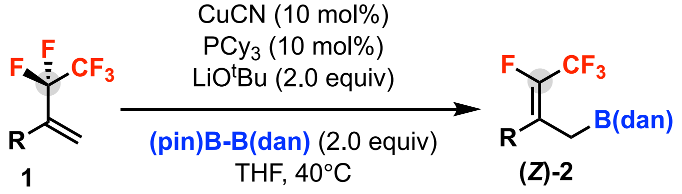

To an oven-dried 10 mL glass tube equipped with a stir bar was added CuCN (0.02 mmol, 0.1 equiv), PCy_3 (0.02 mmol, 0.1 equiv), LiO^{t}Bu (0.4 mmol, 2.0 equiv) and (pin)B-B(dan) (0.4 mmol, 2.0 equiv). Then the tube was sealed with a septum, evacuated and refilled with argon three times, THF (2.0 mL, 0.1 M) was added through syringe under argon and the mixture was stirring at room temperature for 20 min, then the alkene **1** (0.2 mmol) was added by syringe under argon. The resulting mixture was stirring at 40°C. The reaction process was monitored by TLC. After the completion of reaction, the reaction mixture was extracted with CH_2Cl_2 (3 × 10 mL). The combined organic layers were washed with H_2O (2 × 10 mL), then brine (2 × 10 mL), dried over anhydrous Na_2SO_4 and concentrated in vacuo. The residue was purified by flash column chromatography on silica gel to afford products **2**.

General procedure (IX) for the defluoroborylation of alkene 3 (*c.f.* Scheme 4):

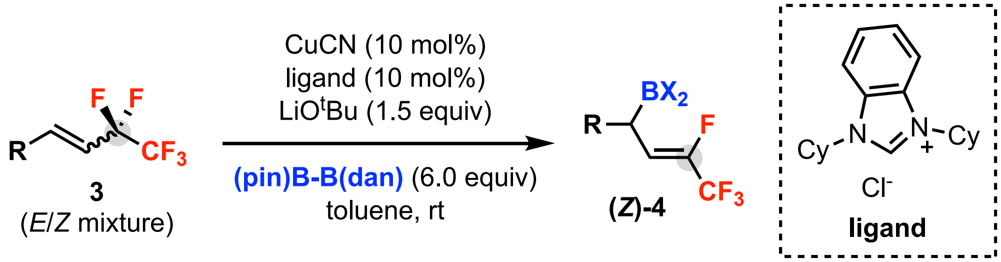

To an oven-dried 10 mL glass tube equipped with a stir bar was added CuCN (0.02 mmol, 0.1 equiv), 1,3-Dicyclohexylbenzimidazolium chloride (0.02 mmol) , 0.1 equiv, LiO^{t}Bu (0.3 mmol, 1.5 equiv) and (pin)B-B(dan) (1.2 mmol, 6.0 equiv). Then the tube was sealed with a septum, evacuated and refilled with argon three times, toluene (2.0 mL, 0.1 M) was added through syringe under argon and the mixture was stirring at room temperature for 20 min, then the alkene **3** or **5** (0.2 mmol) was added by syringe under argon. The resulting mixture was stirring at room temperature for 12 hours. After 12 hours, the reaction mixture was extracted with CH_2Cl_2 (3 × 10 mL). The combined organic layers were washed with H_2O (2 × 10 mL), then brine (2 × 10 mL), dried over anhydrous Na_2SO_4 and concentrated in vacuo. The residue was purified by flash column chromatography on silica gel to afford products **4** or **6**.

General procedure (X) for the one-pot two step defluoroborylation and oxidation of alkene 3 (*c.f.* Scheme 5):

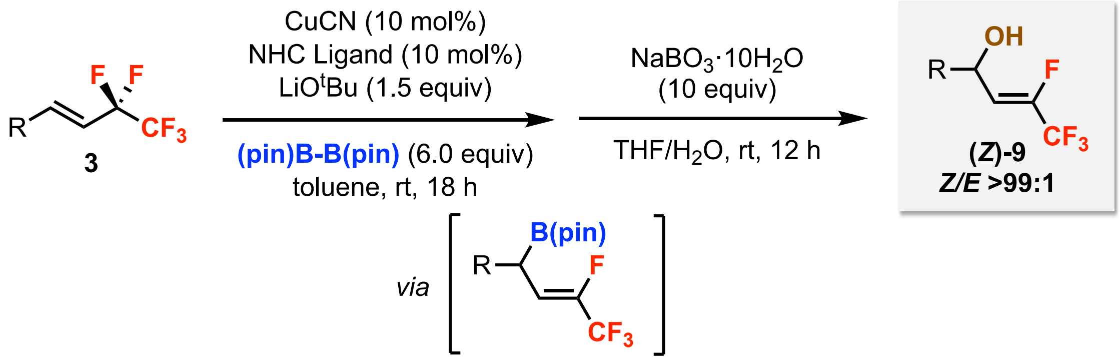

To an oven-dried 10 mL glass tube equipped with a stir bar was added CuCN (0.02 mmol, 0.1 equiv), 1,3-Dicyclohexylbenzimidazolium chloride (0.02 mmol, 0.1 equiv), LiO^{t}Bu (0.3 mmol, 1.5 equiv) and (pin)B-B(pin) (1.2 mmol, 6.0 equiv). Then the tube was sealed with a septum, evacuated and refilled with argon three times, toluene (2.0 mL, 0.1 M) was added through syringe under argon and the mixture was stirring at room temperature for 20 min, then the alkene **3** or **10** (0.2 mmol) was added by syringe under argon. The resulting mixture stirring at room temperature for 18 hours. After 18 hours, the yield of B(pin) product was determined by ^{19}F NMR by using trifluoromethyl benzene as an internal standard.

Then the crude mixture was filtered by micro filtration through a syringe and the solvent was removed by rotary evaporator. Sodium perborate (2.0 mmol, 10.0 equiv) was added into the residue and was dissolved in 2 ml THF/H_2O (v/v=5:1, 0.1 M) solution, then the mixture was stirring at room temperature for 12 hours. After 12 hours, the reaction mixture was extracted with CH_2Cl_2 (3 × 10 mL). The combined organic layers were washed with H_2O (2 × 10 mL), then brine (2 × 10 mL), dried over anhydrous Na_2SO_4 and concentrated in vacuo. The residue was purified by flash column chromatography on silica gel to afford products **9** or **11**.

General procedure (XI) for the enantioselective defluoroborylation of alkene 3. (*c.f.* Scheme 6):

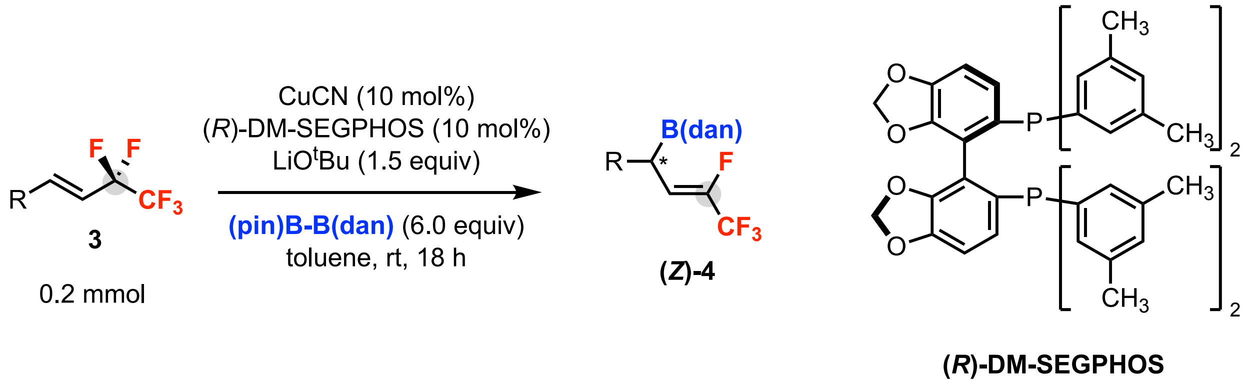

To an oven-dried 10 mL glass tube equipped with a stir bar was added CuCN (0.02 mmol, 0.1 equiv), (*R*)-DM-SEGPHOS (0.02 mmol, 0.1 equiv), LiO*t*Bu (0.3 mmol, 1.5 equiv) and (pin)B-B(dan) (1.2 mmol, 6.0 equiv). Then the tube was sealed with a septum, evacuated and refilled with argon three times, toluene (2.0 mL, 0.1 M) was added through syringe under argon and the mixture was stirring at room temperature for 20 min, then the alkene **3** (0.2 mmol) was added by syringe under argon. The resulting mixture was stirring at room temperature for 12 hours. After 12 hours, the reaction mixture was extracted with CH_2Cl_2 (3 × 10 mL). The combined organic layers were washed with H_2O (2 × 10 mL), then brine (2 × 10 mL), dried over anhydrous Na_2SO_4 and concentrated in vacuo. The residue was purified by flash column chromatography on silica gel to afford products **4**.

General procedure (XII) for the hydrodefluorination of alkene 7 (*c.f.* eq 2):

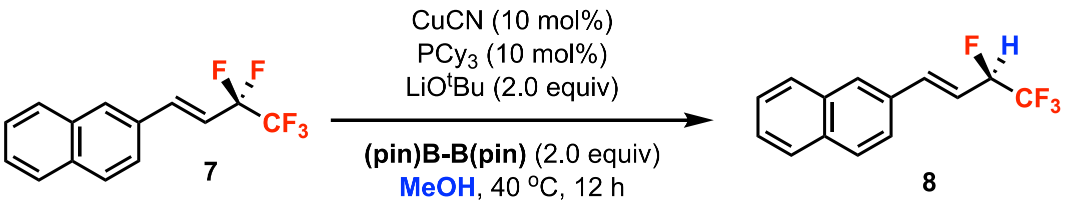

To an oven-dried 10 mL glass tube equipped with a stir bar was added CuCN (0.02 mmol, 0.1 equiv), PCy_3 (0.02 mmol, 0.1 equiv), LiO*t*Bu (0.4 mmol, 2.0 equiv) and (pin)B-B(pin) (0.4 mmol, 2.0 equiv). Then the tube was sealed with a septum, evacuated and refilled with argon three times, MeOH (1.0 mL) was added through syringe under argon and the mixture was stirring at room temperature for 20 min, then the alkene **7** (0.2 mmol) in 1 ml MeOH was added by syringe under argon. The resulting mixture was stirring at 40°C. The reaction process was monitored by TLC. After the completion of reaction, the reaction mixture was extracted with CH_2Cl_2 (3 × 10 mL). The combined organic layers were washed with H_2O (2 × 10 mL), then brine (2 × 10 mL), dried over anhydrous Na_2SO_4 and concentrated in vacuo. The residue was purified by flash column chromatography on silica gel to afford products **8.**

5. Optimization studies

Table S1. Optimization studies for Cu(I)-catalyzed defluoroborylation of pentafluoroethyl alkene 1a.[a]

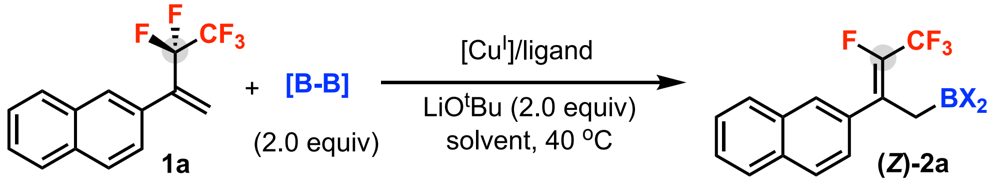

Entry	[CuI] (mol%)	[B-B]	BX2	Ligand (mol%)	Solvent	Time (h)	Yield (%)[b]	*Z/E*[b]
1	CuCN (10)	(pin)B-B(pin)	B(pin)	none	THF	16	41	80:20
2	CuCN (10)	(pin)B-B(pin)	B(pin)	L1 (10)	THF	16	44	82:18
3	CuCN (10)	(pin)B-B(pin)	B(pin)	L2 (10)	THF	16	46	90:10
4	CuCN (10)	(pin)B-B(pin)	B(pin)	L3 (10)	THF	16	53	87:13
5	CuCN (10)	(pin)B-B(pin)	B(pin)	L4 (10)	THF	16	66	75:25
6	CuCN (10)	(pin)B-B(pin)	B(pin)	L5 (10)	THF	16	43	75:25
7	CuCN (10)	(pin)B-B(pin)	B(pin)	L6 (10)	THF	16	11	82:18
8	CuCl (10)	(pin)B-B(pin)	B(pin)	L2 (10)	THF	16	21	76:24
9	$Cu(MeCN)_4PF_6$ (10)	(pin)B-B(pin)	B(pin)	L2 (10)	THF	16	27	81:19
10	CuCN (10)	(pin)B-B(dan)	B(dan)	L1 (10)	THF	16	80	>99:1
11	CuCN (10)	(cat)B-B(cat)	B(cat)	L1 (10)	THF	24	20	50:50
12	CuCN (10)	(nep)B-B(nep)	B(nep)	L1 (10)	THF	24	25	60:40
**13**	**CuCN (10)**	**(pin)B-B(dan)**	**B(dan)**	**L1** (10)	**THF**	**24**	**99**	**>99:1**
14	CuCN (10)	(pin)B-B(dan)	B(dan)	L1 (10)	toluene	24	99	>99:1
15	CuCN (10)	(pin)B-B(dan)	B(dan)	L1 (10)	CH_3CN	24	41	>99:1
16	CuCN (10)	(pin)B-B(dan)	B(dan)	L1 (10)	MeOH	24	47	43:57

[a]Unless specified otherwise, reactions were carried out using **1a** (0.1 mmol) in solvent (1.0 mL) under argon. [b]Yields and *Z/E* ratios were determined by ^{19}F NMR analysis of the crude mixture using benzotrifluoride as internal standard.

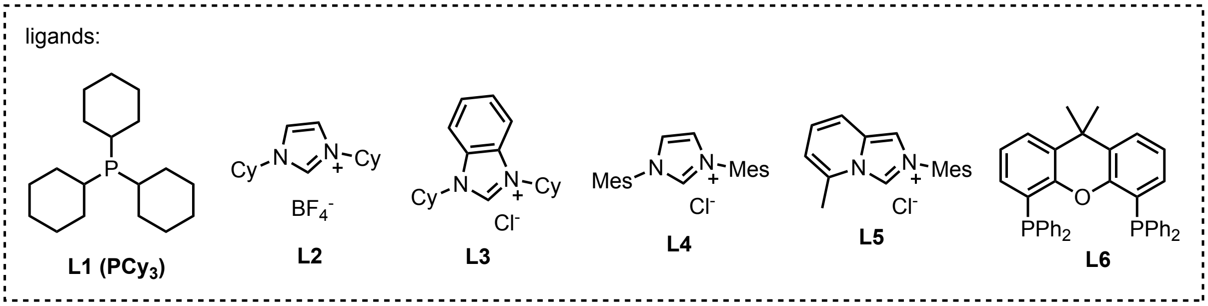

Table S2. Optimization studies for Cu(I)-catalyzed defluoroborylation of pentafluoroethyl alkene 3a.[a]

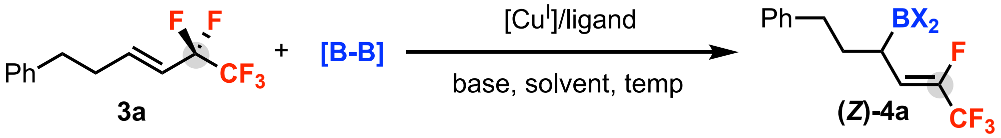

Entry	[Cu^I] (mol%)	[B-B] (equiv)	BX$_2$	Base (equiv)	Ligand (mol%)	solvent	Temp (°C)	Time (h)	Yield (%)[b]	Z/E[b]
1	CuSCN (10)	(pin)B-B(pin) (2.0)	B(pin)	LiO^{t}Bu (2.0)	none	THF	r.t.	12	59	90:10
2	CuI (10)	(pin)B-B(pin) (2.0)	B(pin)	LiO^{t}Bu (2.0)	none	THF	r.t.	12	15	75:25
3	CuCN (10)	(pin)B-B(pin) (2.0)	B(pin)	LiO^{t}Bu (2.0)	none	THF	r.t.	12	67	91:9
4	CuCN (10)	(pin)B-B(pin) (2.0)	B(pin)	LiO^{t}Bu (2.0)	none	THF	0	12	78	90:10
5	CuCN (10)	(pin)B-B(pin) (2.0)	B(pin)	LiO^{t}Bu (2.0)	none	THF	-20	12	68	95:5
6	CuCN (10)	(pin)B-B(pin) (2.0)	B(pin)	LiO^{t}Bu (2.0)	none	THF	-20	24	62	82:18
7	CuCN (10)	(pin)B-B(pin) (2.0)	B(pin)	KO^{t}Bu (2.0)	none	THF	-20	24	0	\
8	CuCN (10)	(pin)B-B(pin) (2.0)	B(pin)	NaO^{t}Bu (2.0)	none	THF	-20	24	41	70:30
9	CuCl (10)	(pin)B-B(pin) (2.0)	B(pin)	KO^{t}Bu (2.0)	none	THF	-20	24	0	\
10	CuCN (10)	(pin)B-B(pin) (2.0)	B(pin)	LiO^{t}Bu (2.0)	**L1** (10)	THF	0	12	54	95:5
11	CuCl (10)	(pin)B-B(pin) (2.0)	B(pin)	LiO^{t}Bu (2.0)	**L1** (10)	THF	0	12	19	74:26
12	Cu(MeCN)$_4$PF$_6$(10)	(pin)B-B(pin) (2.0)	B(pin)	LiO^{t}Bu (2.0)	**L1** (10)	THF	0	12	40	90:10
13	CuI (10)	(pin)B-B(pin) (2.0)	B(pin)	LiO^{t}Bu (2.0)	**L1** (10)	THF	0	12	19	74:26
14	CuCN (10)	(pin)B-B(pin) (2.0)	B(pin)	LiO^{t}Bu (2.0)	**L2** (10)	THF	0	12	62	90:10
15	CuCN (10)	(pin)B-B(pin) (2.0)	B(pin)	LiO^{t}Bu (2.0)	**L3** (10)	THF	0	12	0	\
16	CuCN (10)	(pin)B-B(pin) (2.0)	B(pin)	LiO^{t}Bu (2.0)	**L4** (10)	THF	0	12	13	77:23
17	CuCN (10)	(pin)B-B(pin) (2.0)	B(pin)	LiO^{t}Bu (2.0)	**L5** (10)	THF	0	12	59	92:8
18	CuCN (10)	(pin)B-B(pin) (2.0)	B(pin)	LiO^{t}Bu (2.0)	**L6** (10)	THF	0	12	66	88:12
19	CuCN (10)	(pin)B-B(pin) (2.0)	B(pin)	LiO^{t}Bu (2.0)	**L7** (10)	THF	0	12	29	83:17
20	CuCN (10)	(pin)B-B(pin) (2.0)	B(pin)	LiO^{t}Bu (2.0)	**L8** (10)	THF	0	12	41	88:12
21	CuCN (10)	(pin)B-B(pin) (2.0)	B(pin)	LiO^{t}Bu (2.0)	**L9** (10)	THF	0	12	11	55:45
22	CuCN (10)	(pin)B-B(pin) (2.0)	B(pin)	LiO^{t}Bu (2.0)	**L10** (10)	THF	0	12	36	89:11
23	CuCl (10)	(pin)B-B(pin) (2.0)	B(pin)	LiO^{t}Bu (2.0)	**L6** (10)	THF	0	12	57	90:10
24	Cu(MeCN)$_4$PF$_6$(10)	(pin)B-B(pin) (2.0)	B(pin)	LiO^{t}Bu (2.0)	**L6** (10)	THF	0	12	54	87:13
25	CuI (10)	(pin)B-B(pin) (2.0)	B(pin)	LiO^{t}Bu (2.0)	**L6** (10)	THF	0	12	60	85:15
26	CuCN (10)	(cat)B-B(cat) (2.0)	B(cat)	LiO^{t}Bu (2.0)	**L6** (10)	THF	0	12	0	0
27	CuCN (10)	(nep)B-B(nep) (2.0)	B(nep)	LiO^{t}Bu (2.0)	**L6** (10)	THF	0	12	13	\
28	CuCN (10)	(pin)B-B(pin) (1.1)	B(pin)	LiO^{t}Bu (2.0)	**L6** (10)	THF	0	12	32	94:6
29	CuCN (10)	(pin)B-B(pin) (4.0)	B(pin)	LiO^{t}Bu (2.0)	**L6** (10)	THF	0	12	46	93:7
30	CuCN (10)	(pin)B-B(pin) (8.0)	B(pin)	LiO^{t}Bu (2.0)	L6 (10)	THF	0	12	83	92:8
31	CuCN (10)	(pin)B-B(dan) (2.0)	B(dan)	LiO^{t}Bu (2.0)	L6 (10)	Toluene	r.t.	12	38	99:1
32	CuCN (10)	(pin)B-B(dan) (4.0)	B(dan)	LiO^{t}Bu (2.0)	L6 (10)	Toluene	r.t.	12	62	>99:1
**33**	**CuCN (10)**	**(pin)B-B(dan) (6.0)**	**B(dan)**	**LiO^{t}Bu (1.5)**	**L6 (10)**	**Toluene**	**r.t.**	**12**	**99**	**98:2**
**34**	**CuCN (10)**	**(pin)B-B(pin) (6.0)**	**B(pin)**	**LiO^{t}Bu (1.5)**	**L6 (10)**	**Toluene**	**r.t.**	**12**	**99**	**98:2**
35	CuCN (10)	(pin)B-B(dan) (6.0)	B(dan)	LiO^{t}Bu (1.5)	none	Toluene	r.t.	12	97	96:4
36	CuCN (10)	(pin)B-B(dan) (6.0)	B(dan)	LiO^{t}Bu (1.5)	PCy$_3$ (10)	Toluene	r.t.	12	89	98:2
37[c]	CuCN (10)	(pin)B-B(dan) (6.0)	B(dan)	LiO^{t}Bu (1.5)	L6 (10)	Toluene	r.t.	12	98	98:2
38	CuCN (10)	(pin)B-B(dan) (8.0)	B(dan)	LiO^{t}Bu (2.0)	L6 (10)	Toluene	r.t.	12	99	99:1

[a]Unless specified otherwise, reactions were carried out using **3a** (0.1 mmol) in solvent (1.0 mL) under argon. [b]Yields and *Z/E* ratios were determined by ^{19}F NMR analysis of the crude mixture using benzotrifluoride as internal standard. [c]Using a *E/Z* mixture of **3a** (*E/Z* = 15:85) as the starting material.

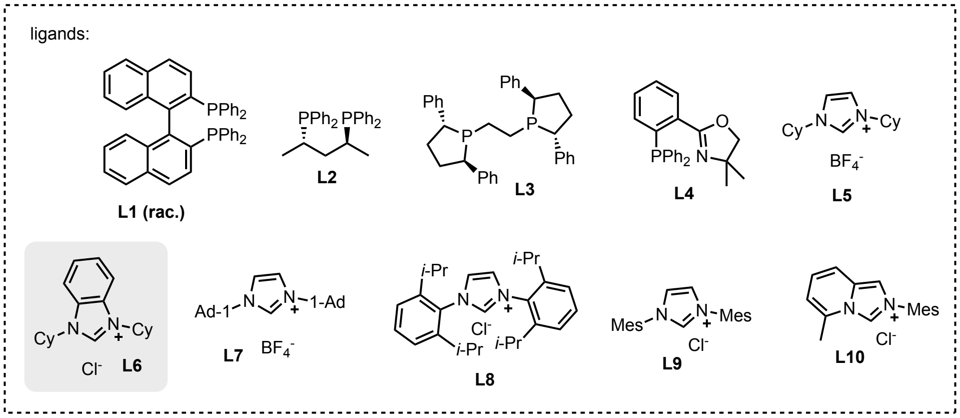

Ligands used for Table S2.

Table S3. Optimization studies for Cu(I)-catalyzed hydrodefluoroborylation of pentafluoroethyl alkene 7. [a]

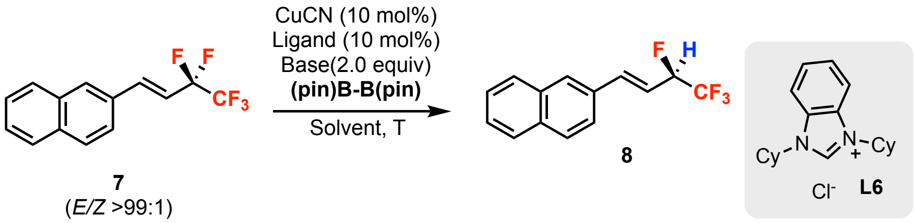

Entry	(pin)B-B(pin)	Base	Ligand	Solvent	T / °C	Yield / *E*/*Z* ratio[a]
1	4.0 equiv.	LiOTMS	**L6**	THF	0	74% (87:13)
2	4.0 equiv.	LiOTMS	**L6**	Toluene/THF = 20:1	0	57% (87:13)
3	4.0 equiv.	LiOTMS	**PCy_3**	THF	0	52% (>99:1)
4	2.0 equiv.	LiO*^t*Bu	**PCy_3**	MeOH	r.t.	76% (95:5)
5	2.0 equiv.	LiO*^t*Bu	**PCy_3**	EtOH	r.t.	71% (97:3)
**6**	**2.0 equiv.**	**LiO*^t*Bu**	**PCy_3**	**MeOH**	**40**	**97% (95:5)**

[a]Unless specified otherwise, reactions were carried out using **7** (0.1 mmol) in solvent (1.0 mL) under argon. Yields and *Z/E* ratios were determined by ^{19}F NMR analysis of the crude mixture using benzotrifluoride as internal standard.

Table S4. Optimization studies for Cu(I)-catalyzed enantioselective defluoroborylation of pentafluoroethyl alkene 3a.[a]

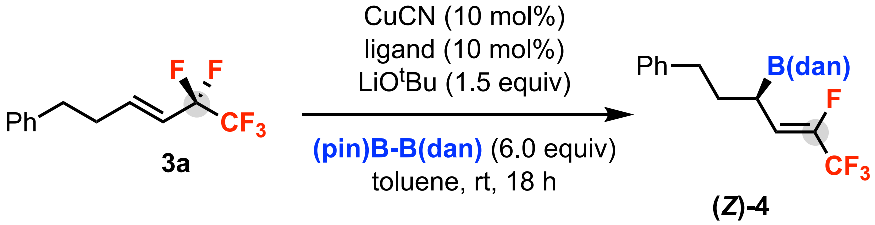

Entry	Ligand (10 mol%)	Solvent	Temp	Yield[b] (%)	*Z*/*E*[b]	e.r. [c]
1	(*R*)-BINAP	Toluene	r.t.	87	99:1	91:9
2	(*S*, *S*)-BDPP	Toluene	r.t.	62	98:2	60:40
3	(*R*)-^{t}Bu-Josiphos	Toluene	r.t.	0	-	-
4	(*R*)-Tol-BINAP	Toluene	r.t.	76	> 99:1	80:20
5	(*R*)-DM-BINAP	Toluene	r.t.	92	> 99:1	90:10
6	(*R*)-DTBM-BINAP	Toluene	r.t.	85	> 99:1	93:7
7	(*R*)-MeO-BIPHEP	Toluene	r.t.	86	> 99:1	93:7
**8**	**(*R*)-DM-Segphos**	**Toluene**	**r.t.**	**84**	**> 99:1**	**95:5**
9	(*R*)-Segphos	Toluene	r.t.	74	> 99:1	92:8
10	(*R*)-DTBM- Segphos	Toluene	r.t.	63	> 99:1	65:35
11	(*R*)-DM-Segphos	THF	r.t.	40%	> 99:1	-
12	(*R*)-DM-Segphos	1,4-dioxane	r.t.	73%	> 99:1	65:35
13	(*R*)-DM-Segphos	Toluene	0 °C	32%	> 99:1	-
14	(*R*)-DM-Segphos	Toluene	50 °C	94%	> 99:1	80:20

[a]Unless specified otherwise, reactions were carried out using **3a** (0.1 mmol) in toluene (1.0 mL) under argon. [b]Yields and *Z/E* ratios were determined by ^{19}F NMR analysis of the crude mixture using benzotrifluoride as internal standard. [c]The % er was determined by HPLC on a chiral stationary phase column.

Ligands

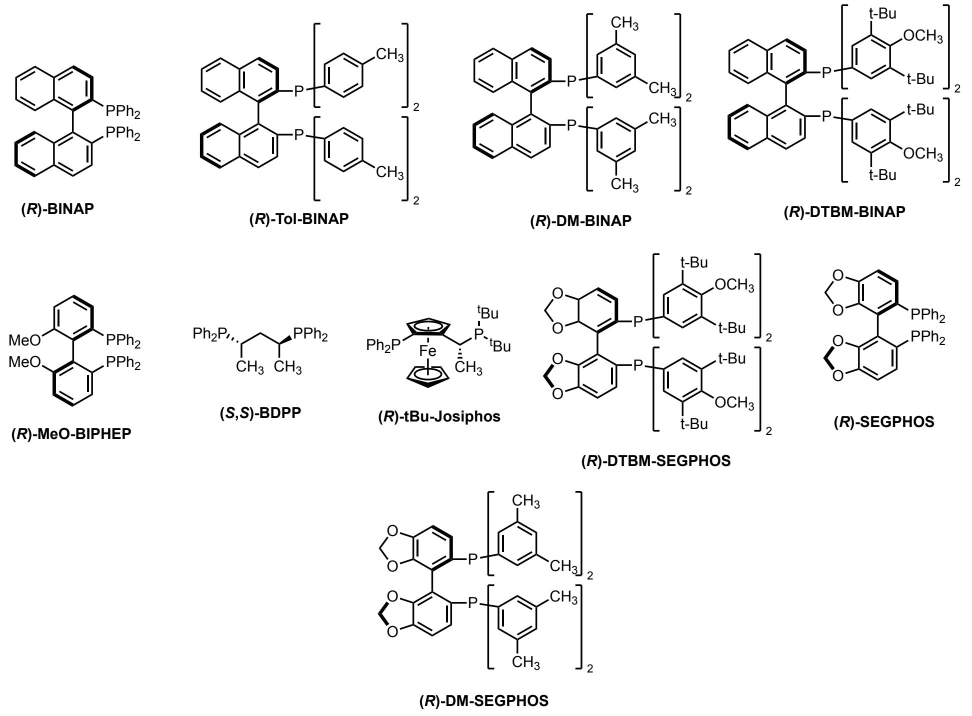

Alkyl-substituted 1,1-disubstituted pentafluoroethyl alkenes under standard conditions:

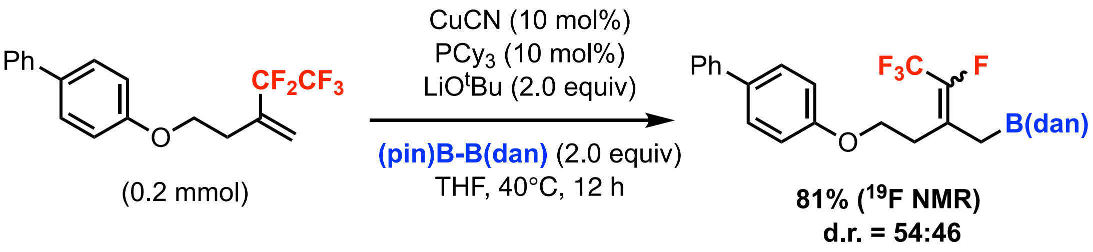

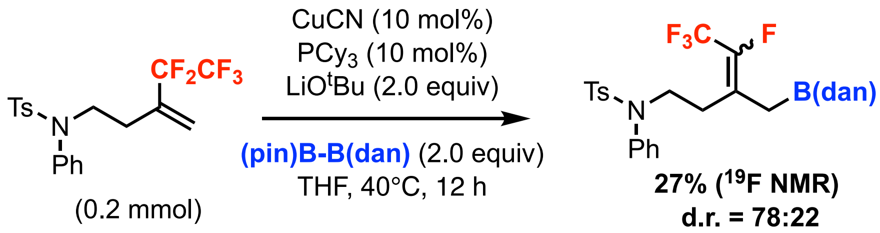

6. ^{19}F-^{1}H HOESY NMR Studies of 2l

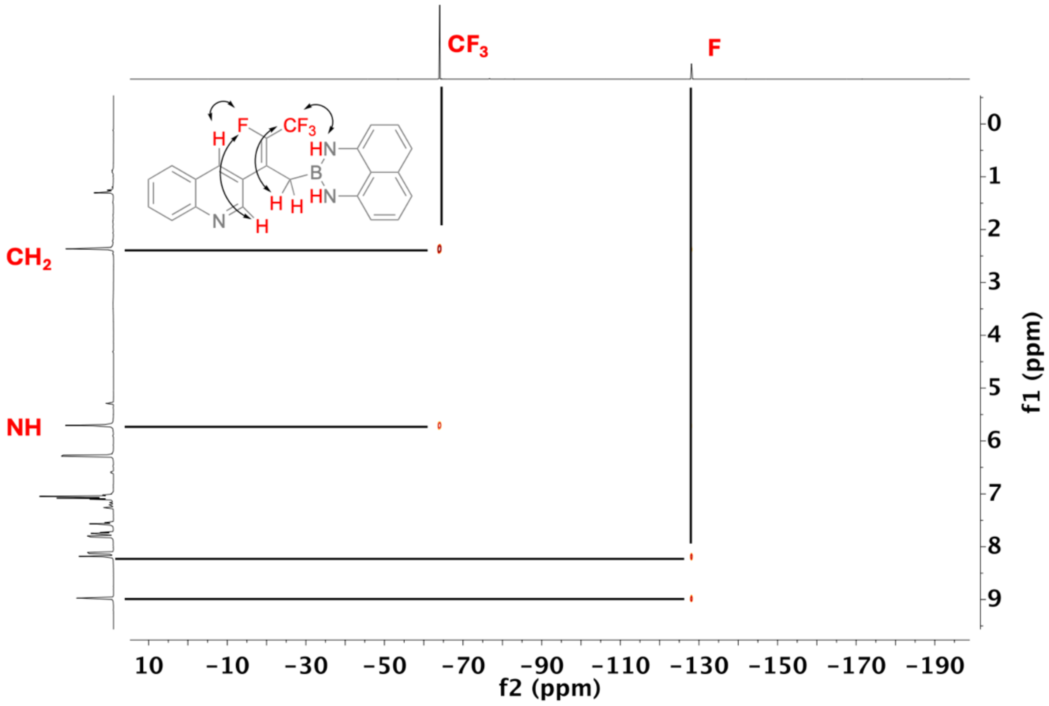

^{19}F-^{1}H HOESY NMR Studies of 4a

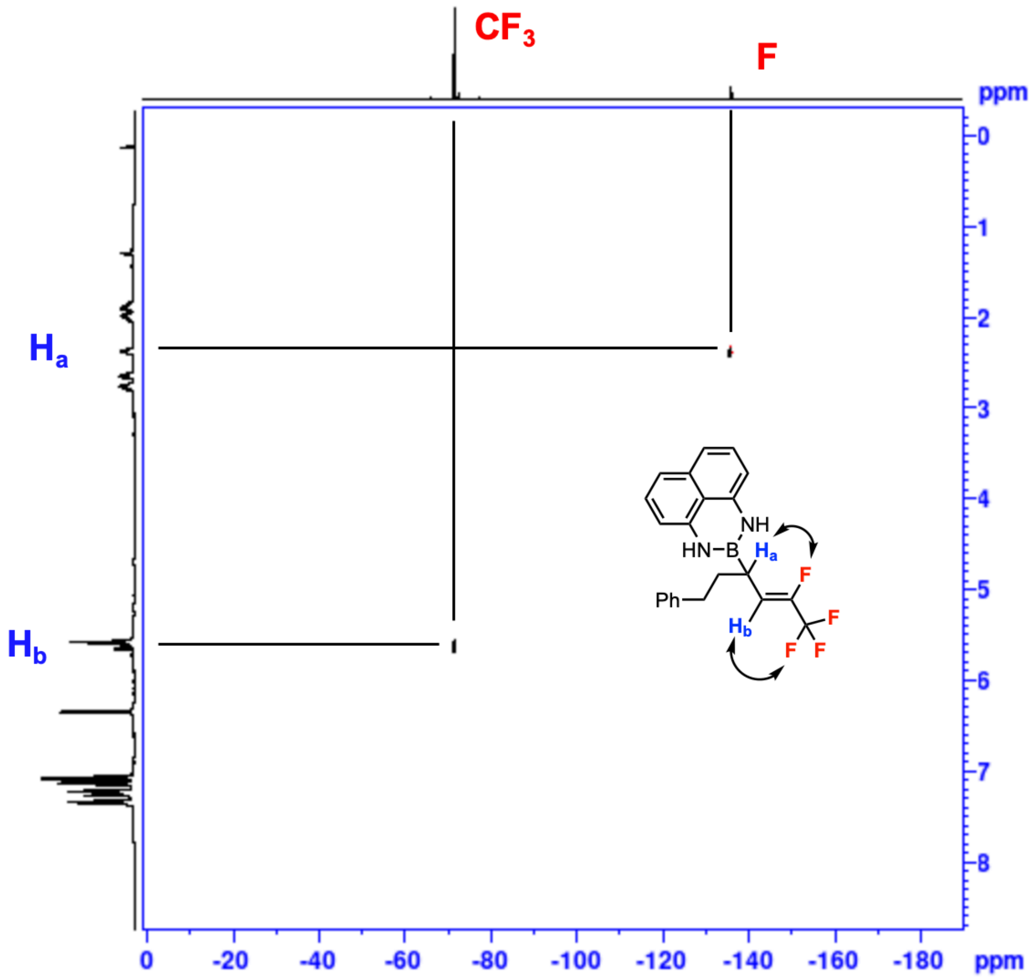

7. Computational Studies

7.1 Computation Method

All Density Functional theory (DFT) Calculations were performed using the Gaussian 16 software Package.[5] Reaction energy profile, geometries of the reactants, transition states, and products were optimized using the B3LYP functional[6-7] and Grimme's D3(BJ) dispersion correction[8-9] with def2-SVP basis set[10]. Vibrational frequency calculations were performed for all the stationary points to confirm each optimized structure is a local minimum or a transition state structure. Intrinsic Reaction Coordinate (IRC) was used to check whether the transition state connected the reactant and product correctly. The single point energies were calculated using PWPB95 functional[11] with RI approximation and Grimme's D4 dispersion correction[12] with def2-TZVPP basis set in SMD continuum solvation model[13] (Toluene) in ORCA, along with corresponding auxiliary basis set.[14-15] The IRI analysis[16] was performed by Multiwfn[16]. CYLview was used for better visualization of this work.[17]

7.2 Thermal correction to Gibbs Free Energy, Electronic Energies and Imaginary Frequencies

	$\mathbf{G_{corr}}$ / Hartree	**E** / Hartree	**G** / Hartree	ν_{imag} / cm^{-1}
Z-3a	0.161007	-962.9467862	-962.78578	
NHC-Cu-B(dan)	0.522711	-3009.640202	-3009.1175	
INT-1	0.714435	-3972.611858	-3971.8974	
TS1	0.715825	-3972.600111	-3971.8843	-167.8187
INT-2	0.713444	-3972.655964	-3971.9425	
TS_{2Z}	0.717437	-3972.657091	-3971.9397	-278.0540
TS_{2E}	0.718631	-3972.653895	-3971.9353	-261.9858
NHC-Cu-F	0.372534	-2589.359244	-2588.9867	
Z-4a	0.317824	-1383.302574	-1382.9847	

7.3 Energy profiles

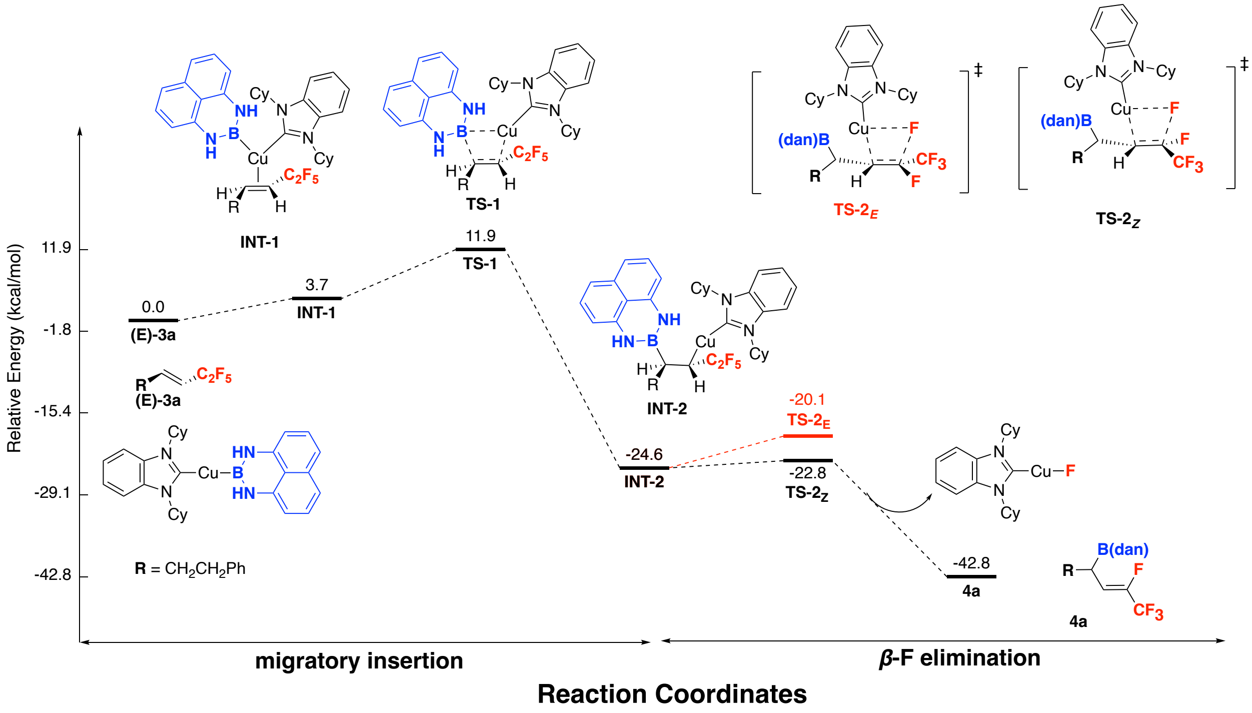

Scheme S1 Reaction energy profile of defluoroborylation of **3a**. All energies were calculated at the RI-PWPB95/def2-TZVPP/SMD(toluene)//B3LYP-D3(BJ)/def2-SVP level of theory.

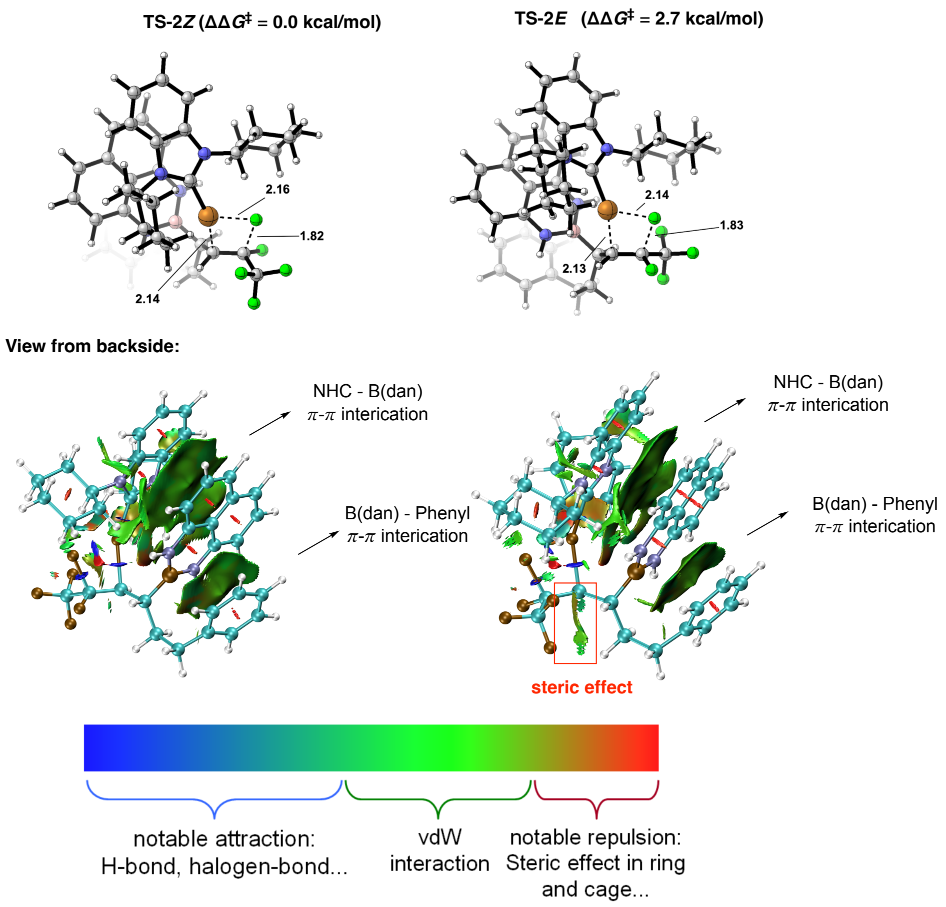

Scheme S2 IRI analysis of **TS-2*Z*** and **TS-2*E***.

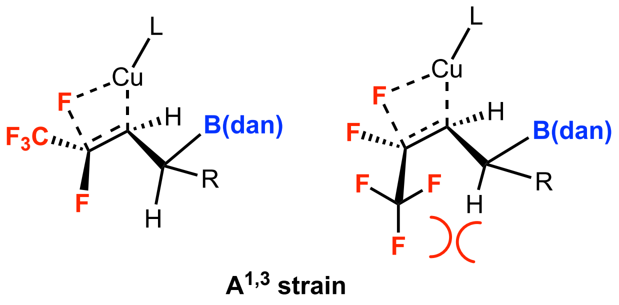

Scheme S3 A^{1,3} strain model of **TS-2*Z*** and **TS-2*E***. (R = $PhCH_2CH_2$)

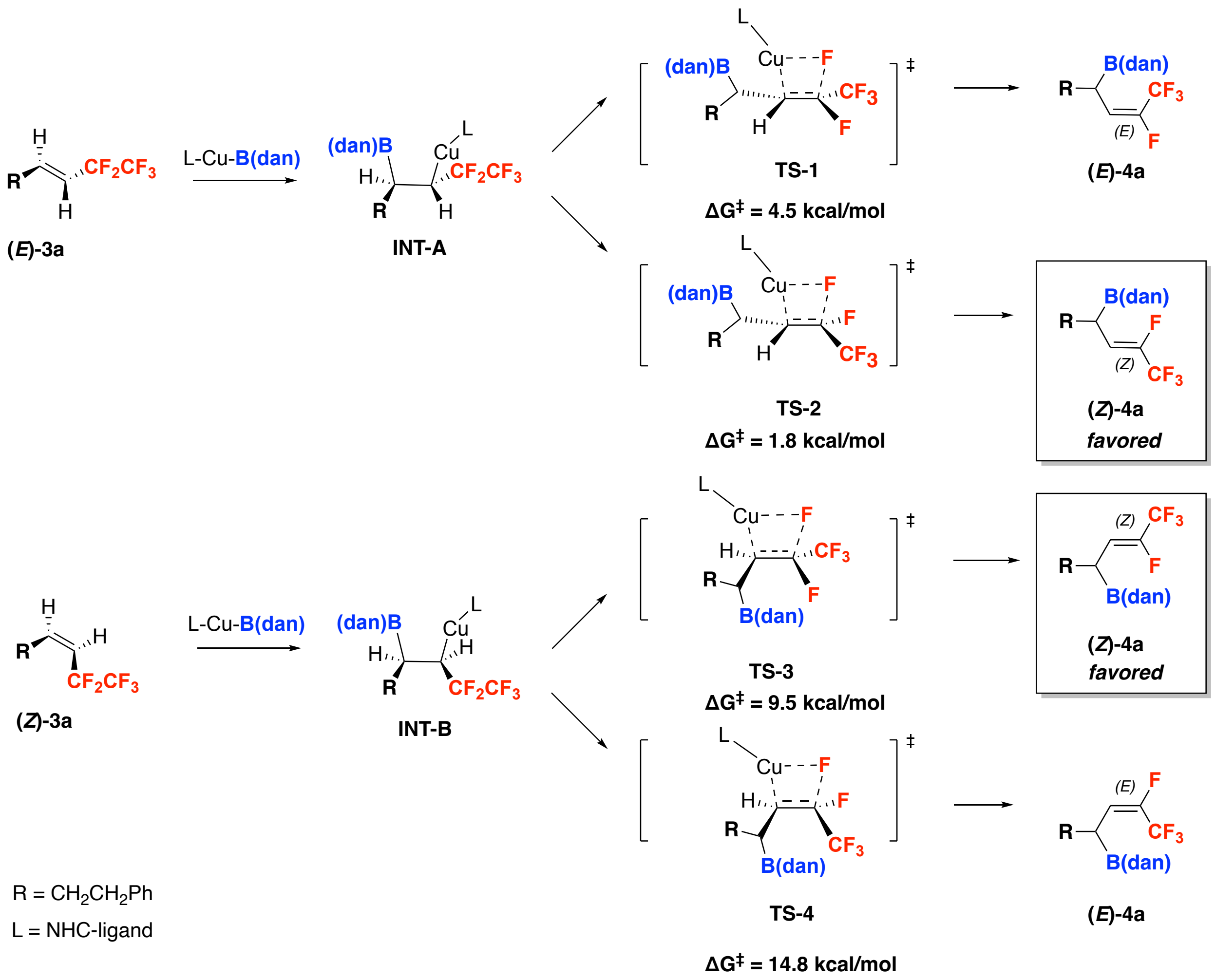

Scheme S4 DFT calculations for the stereoconvergent phenomenon. (L = NHC-ligand, R = $PhCH_2CH_2$)

8. Characterizations

1-methoxy-4-(3,3,4,4,4-pentafluorobut-1-en-2-yl)benzene (1c)

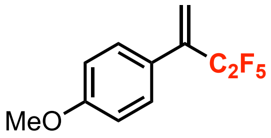

Following the general procedure (III), *a*-CF_2CF_3 ketone **S1c** (1.02 g, 4 mmol) was converted to **1c** as a colorless oil (0.64 g, 63% yield), $R_f = 0.43$ (hexane). **^{1}H NMR** (500 MHz, $CDCl_3$) δ 7.31 (d, *J* = 8.4 Hz, 2H), 6.89 (d, *J* = 8.8 Hz, 2H), 5.93 (s, 1H), 5.73 (s, 1H), 3.83 (s, 3H). **^{19}F NMR** (471 MHz, $CDCl_3$) δ -82.81 (s, 3F), -113.22 (s, 2F). **^{13}C NMR** (126 MHz, $CDCl_3$) δ 160.2, 138.1 (t, *J* = 21.2 Hz), 129.9, 127.3, 123.8 (t, *J* = 8.5 Hz), 123.2 – 115.4 (m), 113.9, 113.7 – 110.6 (m), 55.4. **HRMS** (APCI) m/z: $[M+H]^+$ Calcd for $C_{11}H_{10}F_5O$ 253.0646; Found 253.0642.

methyl 4-(3,3,4,4,4-pentafluorobut-1-en-2-yl)benzoate (1d)

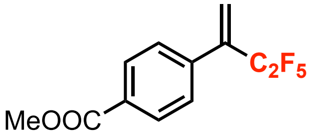

Following the general procedure (III), *a*-CF_2CF_3 ketone **S1d** (1.13 g, 4 mmol) was converted to **1d** as a colorless oil (0.64 g, 57% yield), $R_f = 0.3$ (hexane : DCM = 3:1). **^{1}H NMR** (500 MHz, $CDCl_3$) δ 8.03 (dd *J* = 7.5 Hz, 2H), 7.44 (d, *J* = 7.5 Hz, 2H), 6.07 (s, 1H), 5.84 (s, 1H), 3.92 (s, 3H). **^{19}F NMR** (471 MHz, $CDCl_3$) δ -82.19 – -83.55 (m, 3F), -107.35 – -124.03 (m, 2F). **^{13}C NMR** (126 MHz, $CDCl_3$) δ 166.5, 139.1, 137.8 (t, *J* = 21.7 Hz), 130.6, 129.6, 128.6, 125.8 (t, *J* = 8.4 Hz), 112.7 (tq, *J* = 254.9, 37.8 Hz), 52.2 (d, *J* = 3.4 Hz). **HRMS** (APCI) m/z: $[M+H]^+$ Calcd for $C_{12}H_{10}F_5O_2$ 281.0596; Found 281.0592.

methyl(4-(3,3,4,4,4-pentafluorobut-1-en-2-yl)phenyl)sulfane (1e)

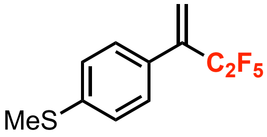

Following the general procedure (III), *a*-CF_2CF_3 ketone **S1e** (1.08 g, 4 mmol) was converted to **1e** as a colorless oil (0.65 g, 61% yield), $R_f = 0.55$ (hexane). **^{1}H NMR** (500 MHz, $CDCl_3$) δ 7.34 (d, *J* = 8.1 Hz, 2H), 7.27 (dd, *J* = 8.4, 1.5 Hz, 2H), 6.01 (d, *J* = 1.5 Hz, 1H), 5.80 (t, *J* = 1.5 Hz, 1H), 2.53 (s, 3H). **^{19}F NMR** (471 MHz, $CDCl_3$) δ -82.86 (s, 3F), -113.20 (s, 2F). **^{13}C NMR** (126 MHz, $CDCl_3$) δ 140.0 (d, *J* = 2.0 Hz), 138.0 (t, *J* = 21.4 Hz), 131.2, 128.8, 125.9, 124.3 (t, *J* = 8.6 Hz), 119.1 (qt, *J* = 287.0, 38.3 Hz), 113.0 (tq, *J* = 254.2, 37.8 Hz), 15.2 (d, *J* = 3.0 Hz). **HRMS** (APCI) m/z: $[M+H]^+$ Calcd for $C_{11}H_{10}F_5S$ 269.0418; Found 269.0418.

1-chloro-4-(3,3,4,4,4-pentafluorobut-1-en-2-yl)benzene (1f)

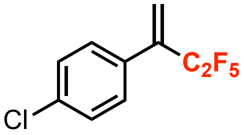

Following the general procedure (III), *a*-CF_2CF_3 ketone **S1f** (1.03 g, 4 mmol) was converted to **1f** as a colorless oil (0.27 g, 26% yield), $R_f = 0.78$ (hexane). **^{1}H NMR** (500 MHz, $CDCl_3$) δ 7.41 – 7.36 (m, 2H), 7.36 – 7.31 (m, 2H), 6.06 (t, *J* = 1.4 Hz, 1H), 5.81 (t, *J* = 1.7 Hz, 1H). **^{19}F NMR** (471 MHz, $CDCl_3$) δ -82.92 (s, 3F), -113.50 (s, 2F). **^{13}C NMR**

(126 MHz, $CDCl_3$) δ 137.6 (t, J = 21.7 Hz), 135.2, 133.2, 129.9, 128.6, 125.1 (t, J = 8.4 Hz), 119.0 (qt, J = 286.8, 38.1 Hz), 112.8 (tq, J = 254.7, 37.9 Hz). **HRMS** (APCI) m/z: $[M+H]^+$ Calcd for $C_{10}H_7ClF_5$ 257.0151; Found 257.0155.

4'-(3,3,4,4,4-pentafluorobut-1-en-2-yl)-[1,1'-biphenyl]-2-carbonitrile (1h)

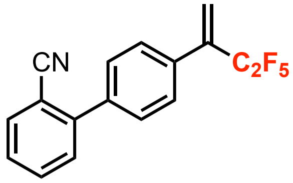

Following the general procedure (III), pentafluoroethyl alkene **1g** (150.5 mg, 0.5 mmol) was converted to **1h** as a yellow solid (137.4 mg, 85% yield), R_f = 0.38 (EA : Hexane = 1:20). ^{1}**H NMR** (500 MHz, $CDCl_3$) δ 7.80 (d, J = 7.8 Hz, 1H), 7.68 (t, J = 7.8 Hz, 1H), 7.61 (d, J = 8.8 Hz, 2H), 7.55 (t, J = 7.3 Hz, 3H), 7.49 (t, J = 7.7 Hz, 1H), 6.10 (s, 1H), 5.90 (s, 1H). ^{19}**F NMR** (471 MHz, $CDCl_3$) δ -82.78 (s, 3F), -112.99 (s, 2F). ^{13}**C NMR** (126 MHz, $CDCl_3$) δ 144.5, 138.7, 138.0 (t, J = 21.7 Hz), 135.1, 133.9, 133.0, 130.1, 128.9, 128.8, 128.0, 125.3 (t, J = 8.4 Hz), 119.1 (qt, J = 286.9, 38.2 Hz), 118.6, 113.0 (qt, J = 254.7, 38.0 Hz), 111.2. **HRMS** (ESI) m/z: $[M+Na]^+$ Calcd for $NaC_{17}H_{10}F_5N$ 346.0627; Found 346.0625.

5-(3,3,4,4,4-pentafluorobut-1-en-2-yl)benzo[*d*][1,3]dioxole (1i)

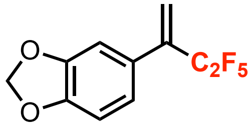

Following the general procedure (III), *a*-CF_2CF_3 ketone **S1i** (1.07 g, 4 mmol) was converted to **1i** as a colorless oil (0.66 g, 62% yield), R_f = 0.43 (hexane). ^{1}**H NMR** (500 MHz, $CDCl_3$): δ (ppm) 6.92 – 6.86 (m, 2H), 6.84 – 6.80 (m, 1H), 6.01 (s, 2H), 5.97 (s, 1H), 5.76 (s, 1H). ^{13}**C NMR** (126 MHz, $CDCl_3$): δ (ppm) 148.3, 147.7, 138.2 (t, J = 21.3 Hz), 128.6, 124.3 (t, J = 8.4 Hz), 122.7, 119.2 (qt, J = 286.8, 38.3 Hz), 113.1 (tq, J = 254.6, 37.8 Hz), 109.1, 108.3, 101.5. ^{19}**F NMR** (471 MHz, $CDCl_3$): δ (ppm) -82.86 (s, 3F), -113.28 (s, 2F). **HRMS** (APCI) m/z: $[M+H]^+$ Calcd for $C_{11}H_8F_5O_2$ 267.0439; Found 267.0435.

2-(3,3,4,4,4-pentafluorobut-1-en-2-yl)benzofuran (1j)

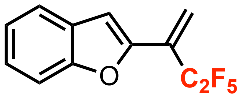

Following the general procedure (III), *a*-CF_2CF_3 ketone **S1j** (1.06 g, 4 mmol) was converted to **1j** as a colorless oil (0.48 g, 46% yield), R_f = 0.81 (hexane). ^{1}**H NMR** (500 MHz, $CDCl_3$): δ (ppm) 7.63 (d, J = 7.8 Hz, 1H), 7.53 (d, J = 8.2 Hz, 1H), 7.41 – 7.28 (m, 2H), 6.94 (s, 1H), 6.54 (s, 1H), 6.04 (s, 1H). ^{13}**C NMR** (126 MHz, $CDCl_3$): δ (ppm) 154.6, 148.5, 128.7, 128.4 (t, J = 23.6 Hz), 125.9, 123.4, 122.0 (t, J = 9.1 Hz), 119.1 (qt, J = 287.0, 38.4 Hz), 113.7 (tq, J = 254.8, 39.1 Hz), 111.2, 107.2. ^{19}**F NMR** (471 MHz, $CDCl_3$): δ (ppm) -83.89 (s, 3F), -114.40 (s, 2F). **HRMS** (APCI) m/z: $[M+H]^+$ Calcd for $C_{12}H_8F_5O$ 263.0490; Found 263.0486.

2-(3,3,4,4,4-pentafluorobut-1-en-2-yl)benzo[*b*]thiophene (1k)

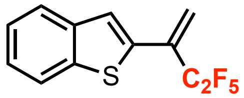

Following the general procedure (III), *a*-CF_2CF_3 ketone **S1k** (1.12 g, 4 mmol) was converted to **1k** as a white solid (0.58 g, 52% yield), R_f = 0.74 (hexane). **^{1}H NMR** (500 MHz, $CDCl_3$) δ 7.82 – 7.72 (m, 2H), 7.43 (d, *J* = 2.0 Hz, 1H), 7.37 (dd, *J* = 6.0, 3.1 Hz, 2H), 6.11 (d, *J* = 2.2 Hz, 1H), 5.99 (s, 1H). **^{19}F NMR** (471 MHz, $CDCl_3$) δ -83.20 (s, 3F), -113.22 (s, 2F). **^{13}C NMR** (126 MHz, $CDCl_3$) δ 140.1, 139.2, 135.8, 132.2 (t, *J* = 22.9 Hz), 125.7, 124.9, 124.6 (d, *J* = 3.5 Hz), 124.5, 124.3 (t, *J* = 8.5 Hz), 122.0, 119.0 (qt, *J* = 287.2, 38.0 Hz), 116.3 – 109.8 (m). **HRMS** (APCI) m/z: $[M+H]^+$ Calcd for $C_{12}H_8F_5S$ 279.0261; Found 279.0261.

3-(3,3,4,4,4-pentafluorobut-1-en-2-yl)quinoline (1l)

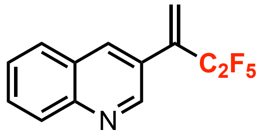

Following the general procedure (III), *a*-CF_2CF_3 ketone **S1l** (1.1 g, 4 mmol) was converted to **1l** as a brown oil (0.31 g, 28% yield), R_f = 0.31 (hexane : DCM = 3:1). **^{1}H NMR** (500 MHz, $CDCl_3$) δ 9.04 – 8.74 (m, 1H), 8.17 (d, *J* = 2.3 Hz, 1H), 8.12 (d, *J* = 8.5 Hz, 1H), 7.82 (d, *J* = 8.2 Hz, 1H), 7.78 – 7.69 (m, 1H), 7.57 (dd, *J* = 8.2, 6.9 Hz, 1H), 6.18 (d, *J* = 1.7 Hz, 1H), 5.95 (d, *J* = 1.8 Hz, 1H). **^{19}F NMR** (471 MHz, $CDCl_3$) δ -82.81 (s, 3F), -113.44 (s, 2F). **^{13}C NMR** (101 MHz, $CDCl_3$) δ 149.8, 148.0, 135.8 (t, *J* = 22.2 Hz), 135.7, 130.5, 129.4, 128.3, 127.8, 127.4, 127.2, 126.7 (t, *J* = 8.4 Hz), 119.1 (qt, *J* = 286.9, 38.0 Hz), 112.9 (tq, *J* = 254.9, 38.2 Hz). **HRMS** (APCI) m/z: $[M+H]^+$ Calcd for $C_{13}H_9F_5N$ 274.0650; Found 274.0644.

(*E*)-1-(3,3,4,4,4-pentafluorobut-1-en-2-yl)-4-styrylbenzene (1m)

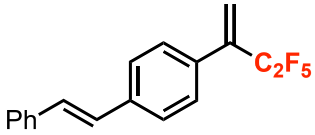

Following the general procedure (III), pentafluoroethyl alkene **1g** (150.5 mg, 0.5 mmol) was converted to **1m** as a yellow solid (113.4 mg, 70% yield), R_f = 0.28 (EA : Hexane = 1:50). **^{1}H NMR** (500 MHz, $CDCl_3$) δ 7.57 (dd, *J* = 13.9, 7.9 Hz, 4H), 7.44 (t, *J* = 7.9 Hz, 4H), 7.35 (t, *J* = 7.3 Hz, 1H), 7.25 – 7.12 (m, 2H), 6.07 (s, 1H), 5.86 (s, 1H). **^{19}F NMR** (471 MHz, $CDCl_3$) δ -82.72(s, 3F), -112.91(s, 2F). **^{13}C NMR** (126 MHz, $CDCl_3$) δ 138.3 (t, *J* = 21.4 Hz), 138.1, 137.2, 133.9, 129.9, 128.9, 128.9, 128.1, 127.9, 126.8, 126.6, 124.5 (t, *J* = 8.5 Hz), 119.2 (tq, *J* = 286.8, 38.3 Hz), 113.2 (tq, *J* = 254.5, 37.8 Hz). **HRMS** (APCI) m/z: $[M+H]^+$ Calcd for $C_{18}H_{14}F_5$ 325.1010; Found 325.1010.

(*E*)-(5,5,6,6,6-pentafluorohex-3-en-1-yl)benzene (3a)

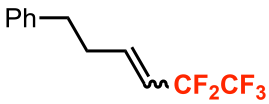

Following the general procedure (VII), alkene **S3a** (132.2 mg, 1.0 mmol) was converted to **3a** as a colorless oil (630 mg, 58% yield), R_f = 0.57 (hexane). The *E*/*Z* ratio of **3a** is 88:12. **^{1}H NMR** (500 MHz, $CDCl_3$) δ 7.46 – 7.34 (m, 2H), 7.27 (dt, *J* = 24.1, 6.3 Hz, 3H), 6.53 (ddd, *J* = 15.9, 6.5, 3.1 Hz, 1H), 5.66 (q, *J* = 13.4 Hz, 1H), 2.84 (t, *J* = 8.2 Hz, 2H), 2.57 (d, *J* = 8.5 Hz, 2H). **^{19}F NMR** (471 MHz, $CDCl_3$) *E*-isomer: δ -85.42 (s, 3F), -115.21 (d, *J* = 11.3 Hz, 2F). *Z*-isomer δ -85.72 (s, 3F), -110.75 (d, *J* = 15.3 Hz, 2F). The spectral data are in full accordance with the literature report.[2]

(E)-7,7,8,8,8-pentafluorooct-5-en-1-yl thiophene-3-carboxylate (3b)

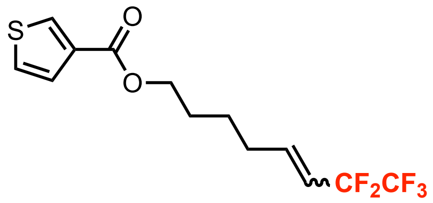

Following the general procedure (VII), alkene **S3b** (210.3 mg, 1.0 mmol) was converted to **3b** as a colorless oil (104.9 mg, 32% yield), R_f = 0.40 (EA/hexane=1:25). The *E*/*Z* ratio of **3b** is 87:13**.** **^{1}H NMR (500 MHz, $CDCl_3$)** δ 8.09 (s, 1H), 7.51 (d, *J* = 4.9 Hz, 1H), 7.29 (dd, *J* = 5.2, 2.9 Hz, 1H), 6.42 (dt, *J* = 14.9, 6.9 Hz, 1H), 5.60 (d, *J* = 12.4 Hz, 1H), 4.28 (t, *J* = 6.5 Hz, 2H), 2.26 (d, *J* = 7.7 Hz, 2H), 1.76 (p, *J* = 6.8 Hz, 2H), 1.59 (dq, *J* = 12.8, 6.7, 5.6 Hz, 2H). **^{19}F NMR (471 MHz, $CDCl_3$)** *E*-isomer: δ -85.48 (s, 3F), -115.19 (d, *J* = 11.8 Hz, 2F) *Z*-isomer: -85.80 (s, 3F), -110.63 (d, *J* = 15.6 Hz, 2F). **^{13}C NMR (126 MHz, $CDCl_3$)** δ 162.9, 142.5 (t, *J* = 8.7 Hz), 133.8, 132.7, 128.0, 126.1, 119.1 (qt, *J* = 285.2, 38.5 Hz), 117.2 (t, *J* = 23.1 Hz), 112.1 (tq, *J* = 249.5, 38.3 Hz), 64.2, 31.6, 28.2, 24.6. **HRMS** (ESI) m/z: $[M+Na]^+$ Calcd for $C_{13}H_{13}F_5O_2SNa$ 351.0449; Found 351.0448.

(*E*)-12,12,13,13,13-pentafluorotridec-10-en-1-yl benzoate (3c)

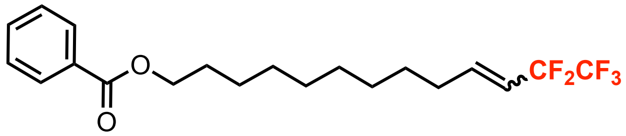

Following the general procedure (VII), alkene **S3c** (274.4 mg, 1 mmol) was converted to **3c** as a colorless oil (176.4 mg, 45% yield), R_f = 0.30 (EA/hexane=1:25). The *E*/*Z* ratio of **3c** is 90:10. **^{1}H** NMR (500 MHz, $CDCl_3$) δ 8.05 (d, *J* = 7.7 Hz, 2H), 7.55 (t, *J* = 7.4 Hz, 1H), 7.43 (t, *J* = 7.6 Hz, 2H), 6.41 (dt, *J* = 14.9, 7.0 Hz, 1H), 5.77 – 5.43 (m, 1H), 4.32 (t, *J* = 6.7 Hz, 2H), 2.35 – 2.05 (m, 2H), 1.77 (p, *J* = 6.9 Hz, 2H), 1.54 – 1.42 (m, 4H), 1.37 – 1.07 (m, 8H). **^{19}F NMR** (471 MHz, $CDCl_3$) *E*-isomer:δ -85.50 (s, 3F), -115.08 (d, *J* = 11.7 Hz). *Z*-isomer: δ -85.80 (s, 3F), -110.56 (d, *J* = 15.3 Hz). **^{13}C** NMR (126 MHz, $CDCl_3$) δ 166.8, 143.3 (t, *J* = 8.6 Hz), 132.9, 130.7, 129.6, 128.4, 119.2 (qt, *J* = 285.2, 38.6 Hz), 116.6 (t, *J* = 23.0 Hz), 112.3 (tq, *J* = 249.7, 38.3 Hz), 65.2, 32.1, 29.5, 29.4, 29.3, 29.0, 28.8, 28.0, 26.1. **HRMS** (ESI) m/z: $[M+Na]^+$ Calcd for $C_{20}H_{25}F_5O_2Na$ 415.1667; Found 415.1665.

(*E*)-4-methyl-7-((7,7,8,8,8-pentafluorooct-5-en-1-yl)oxy)-2*H*-chromen-2-one (3d)

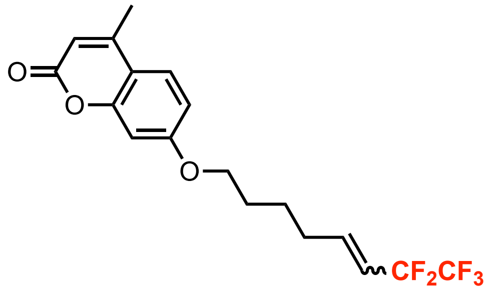

Following the general procedure (VII), alkene **S3d** (258.3 mg, 1 mmol) was converted to **3d** as a yellow oil (150.4 mg, 40% yield), R_f = 0.30 (EA/hexane=1:4). The *E*/*Z* ratio of **3d** is 86:14. **^{1}H NMR** (500 MHz, $CDCl_3$) δ 7.47 (d, *J* = 8.6 Hz, 1H), 6.83 (dd, *J* = 8.8, 2.5 Hz, 1H), 6.77 (d, *J* = 2.4 Hz, 1H), 6.43 (dtt, *J* = 15.9, 6.8, 2.3 Hz, 1H), 6.14 – 6.02 (m, 1H), 5.61 (dt, *J* = 15.9, 11.8 Hz, 1H), 4.01 (t, *J* = 6.1 Hz, 2H), 2.37 (d, *J* = 1.3 Hz, 3H), 2.32 – 2.22 (m, 2H), 1.87 – 1.79 (m, 2H), 1.72 – 1.62 (m, 2H). **^{19}F NMR** (471 MHz, $CDCl_3$) *E*-isomer: δ -85.41(s, 3F), -115.15 (d, *J* = 11.3 Hz, 2F). *Z*-isomer: -85.73(s, 3F), -110.57 (d, *J* = 15.7 Hz, 2F). **^{13}C NMR** (126 MHz, $CDCl_3$) δ 162.1, 161.4, 155.4, 152.7, 142.5 (t, *J* = 8.7 Hz), 125.6, 119.1 (dt, *J* = 285.3, 38.5 Hz), 117.2 (t, *J* = 23.1 Hz), 113.6, 112.2 (tq, *J* = 249.8, 38.3

Hz), 112.6, 112.0, 101.4, 68.1, 31.7, 28.5, 24.6, 18.7. **HRMS** (ESI) m/z: $[M+Na]^+$ Calcd for $C_{18}H_{17}F_5O_3Na$ 399.0990; Found 399.0987.

(8*R*,9*S*,13*S*,14*S*)-13-methyl-3-(((*E*)-7,7,8,8,8-pentafluorooct-5-en-1-yl)oxy)-6,7,8,9,11,12,13,14,15,16-decahydro-17*H*-cyclopenta[*a*]phenanthren-17-one (3e)

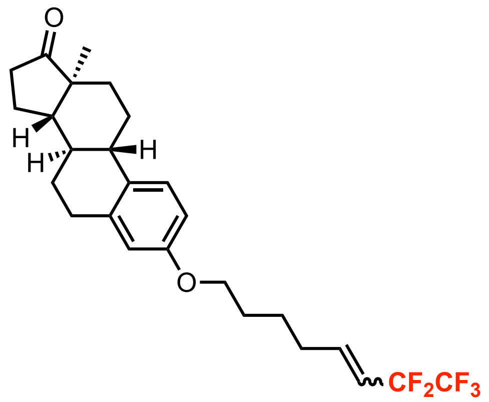

Following the general procedure (VII), alkene **S3e** (352.5 mg, 1 mmol) was converted to **3e** as a white solid (239.7 mg, 51% yield), R_f = 0.25 (EA/hexane=1:5). The *E*/*Z* ratio of **3e** is 88:12. **^{1}H NMR** (500 MHz, $CDCl_3$) δ 7.21 (d, *J* = 8.7 Hz, 1H), 6.72 (d, *J* = 8.6 Hz, 1H), 6.65 (s, 1H), 6.45 (dt, *J* = 15.1, 7.0 Hz, 1H), 5.62 (q, *J* = 12.7 Hz, 1H), 3.96 (t, *J* = 6.3 Hz, 2H), 3.01 – 2.84 (m, 2H), 2.51 (dd, *J* = 19.1, 8.8 Hz, 1H), 2.40 (d, *J* = 11.4 Hz, 1H), 2.26 (s, 3H), 2.15 (s, 1H), 2.10 – 1.98 (m, 2H), 1.99 – 1.92 (m, 1H), 1.81 (s, 2H), 1.71 – 1.58 (m, 4H), 1.56 – 1.40 (m, 4H), 0.92 (s, 3H). **^{19}F NMR** (471 MHz, $CDCl_3$) δ *E*-isomer: -85.39 (s, 3F), -115.08 (d, *J* = 11.6 Hz, 2F). *Z*-isomer: -85.70 (s, 3F), -110.54 (d, *J* = 14.9 Hz, 2F). **^{13}C NMR** (126 MHz, $CDCl_3$) δ 221.1, 157.0, 142.7 (t, *J* = 8.6 Hz), 137.9, 132.2, 126.4, 119.1 (dt, *J* = 285.2, 38.6 Hz), 117.0 (t, *J* = 23.1 Hz), 114.6, 112.1, 67.4, 50.5, 48.1, 44.1, 38.5, 36.0, 31.7, 31.7, 29.8, 28.7, 26.7, 26.0, 24.7, 21.7, 13.9. **HRMS** (ESI) m/z: $[M+Na]^+$ Calcd for $C_{26}H_{31}F_5O_2Na$ 493.2136; Found 493.2135.

(*E*)-1-bromo-4-(((5,5,6,6,6-pentafluorohex-3-en-1-yl)oxy)methyl)benzene (S9b)

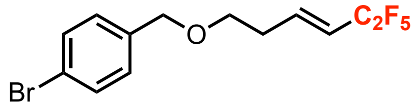

Following the general procedure (V), alkenyl iodide (367.0 mg, 1 mmol) was converted to **S9b** as a yellow oil (215.4 mg, 60% yield), R_f = 0.28 (Hexane). The *E*/*Z* ratio of **S9b** is >99:1. **^{1}H NMR** (500 MHz, $CDCl_3$) δ 7.48 (d, *J* = 8.3 Hz, 2H), 7.19 (d, *J* = 8.0 Hz, 2H), 6.51 – 6.42 (m, 1H), 5.68 (dt, *J* = 15.9, 11.9 Hz, 1H), 4.47 (s, 2H), 3.57 (t, *J* = 6.2 Hz, 2H), 2.55 – 2.45 (m, 2H). **^{19}F NMR** (471 MHz, $CDCl_3$) δ -85.35 (s, 3F), -115.33 (d, *J* = 11.8 Hz, 2F). **^{13}C NMR** (126 MHz, $CDCl_3$) δ 139.8 (t, *J* = 9.0 Hz), 137.2, 131.7, 129.3, 121.7, 119.1 (qt, J = 285.7, 38.5 Hz), 118.5 (t, J = 23.1 Hz), 112.1 (tq, J = 250.0, 38.4 Hz), 72.4, 68.3, 32.5. **HRMS** (ESI) m/z: $[M+Na]^+$ Calcd for $C_{13}H_{12}BrF_5ONa$ 380.9884; Found 380.9884.

(*E*)-4-methoxy-4'-(((5,5,6,6,6-pentafluorohex-3-en-1-yl)oxy)methyl)-1,1'-biphenyl (S9c)

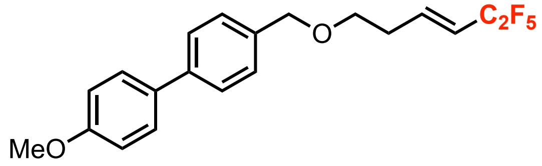

Following the general procedure (V), alkenyl iodide (394.2 mg, 1 mmol) was converted to **S9c** as a yellow oil (154.4 mg, 40% yield), R_f = 0.31 (EA : Hexane = 1:5). The *E*/*Z* ratio of **S9c** is >99:1.**^{1}H NMR** (500 MHz, $CDCl_3$) δ 7.64 –

7.56 (m, 4H), 7.43 (d, *J* = 7.5 Hz, 2H), 7.04 (d, *J* = 8.2 Hz, 2H), 6.64 – 6.40 (m, 1H), 5.76 (q, *J* = 12.7 Hz, 1H), 4.61 (d, *J* = 2.5 Hz, 2H), 3.90 (s, 3H), 3.66 (t, *J* = 6.3 Hz, 2H), 2.60 – 2.45 (m, 2H). ^{19}**F NMR** (471 MHz, $CDCl_3$) δ -85.3 (s, 3F), -115.2 (d, *J* = 12.1 Hz, 2F). ^{13}**C NMR** (126 MHz, $CDCl_3$) δ 159.3, 140.4, 140.0 (t, *J* = 8.9 Hz), 136.5, 133.5, 131.7, 128.2, 128.1, 126.9, 119.1 (dt, *J* = 285.1, 38.1 Hz), 114.3, 112.2 (q, *J* = 249.9, 38.3 Hz), 72.9, 68.2, 55.4, 32.5. **HRMS** (ESI) m/z: $[M+Na]^+$ Calcd for $C_{20}H_{19}F_5O_2Na$ 409.1197; Found 409.1198.

(*E*)-1-methoxy-4-((7,7,8,8,8-pentafluorooct-5-en-1-yl)oxy)benzene (S9d)

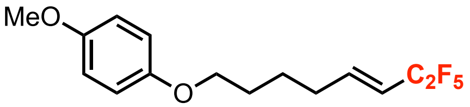

Following the general procedure (V), alkenyl iodide **S3.3d** (332.2mg, 1 mmol) was converted to **3.3d** as a yellow oil (188.0 mg, 58% yield), R_f = 0.20 (EA : Hexane = 1:5). The *E*/*Z* ratio of **S9d** is >99:1. ^{1}**H NMR** (500 MHz, $CDCl_3$) δ 6.87 (s, 4H), 6.48 (dtt, *J* = 15.9, 6.8, 2.3 Hz, 1H), 5.65 (dt, *J* = 16.1, 12.0 Hz, 1H), 3.96 (t, *J* = 6.2 Hz, 2H), 3.80 (s, 3H), 2.35 – 2.27 (m, 2H), 1.87 – 1.78 (m, 2H), 1.73 – 1.63 (m, 2H). ^{19}**F NMR** (471 MHz, $CDCl_3$) δ -85.45 (s, 3F), -115.12 (d, *J* = 11.8 Hz, 2F). ^{13}**C NMR** (126 MHz, $CDCl_3$) δ 154.0, 153.2, 142.8 (t, *J* = 8.7 Hz), 119.1 (qt, *J* = 285.3, 38.4 Hz), 117.1 (t, *J* = 23.0 Hz), 115.5, 114.8, 112.3 (tq, *J* = 249.7, 38.2 Hz), 68.1, 55.8, 31.8, 28.8, 24.8. **HRMS** (ESI) m/z: $[M+Na]^+$ Calcd for $C_{15}H_{17}F_5O_2Na$ 347.1041; Found 347.1040.

(*E*)-(5,5,6,6,7,7,7-heptafluorohept-3-en-1-yl)benzene (10)

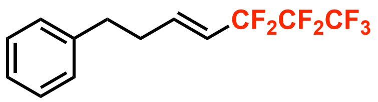

Following the general procedure (VI), alkenyl iodide **S7** (361 mg, 1.4 mmol) was converted to **7** as a colorless oil (336 mg, 80% yield), R_f = 0.58 (hexane). The *E*/*Z* ratio of **10** is >99:1. ^{1}**H NMR** (500 MHz, Chloroform-*d*) δ 7.33 (t, *J* = 7.5 Hz, 2H), 7.25 (d, *J* = 7.5 Hz, 1H), 7.23 – 7.13 (m, 2H), 6.47 (dddd, *J* = 15.9, 9.1, 6.8, 2.3 Hz, 1H), 5.63 (dt, *J* = 15.7, 12.1 Hz, 1H), 2.80 (t, *J* = 7.7 Hz, 2H), 2.55 (tdd, *J* = 9.1, 7.3, 6.1, 3.2 Hz, 2H). ^{19}**F NMR** (471 MHz, Chloroform-*d*) δ -80.43 (t, *J* = 9.2 Hz, 3F), -112.46 (p, *J* = 9.6 Hz, 3F), -127.92 (s, 2F). ^{13}**C NMR** (126 MHz, Chloroform-*d*) δ 142.3 (t, *J* = 9.1 Hz), 140.5, 128.7, 128.5, 126.5, 118.1 (qt, *J* = 287.1, 34.1 Hz), 117.5 (t, *J* = 23.0 Hz), 114.0 (tt, *J* = 251.5, 30.6 Hz), 108.8 (tq, *J* = 263.5, 37.4 Hz), 34.5, 33.9.**HRMS** (APCI) m/z: $[M+H]^+$ Calcd for $C_{13}H_{12}F_7$ 301.0822; Found 301.0824.

(*Z*)-4-methyl-*N*-phenyl-*N*-(8,9,9,9-tetrafluoro-6-(triethylsilyl)non-7-en-1-yl)benzenesulfonamide (3f)

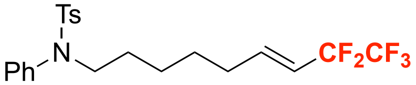

Following the general procedure (V), alkenyl iodide **S3f** (1.41 g, 3 mmol) was converted to **3g** as a colorless oil (1.07 g, 77% yield), R_f = 0.41 (hexane : DCM = 2:3). The *E*/*Z* ratio of **3f** is >99:1. ^{1}**H NMR** (500 MHz, $CDCl_3$): δ (ppm) 7.45 (d, *J* = 8.3 Hz, 2H), 7.34 – 7.21 (m, 5H), 7.06 – 7.04 (m, 2H), 6.42 – 6.34 (m, 1H), 5.53 (dt, *J* = 15.9, 11.9 Hz, 1H), 3.52 (t, *J* = 6.7 Hz, 2H), 2.41 (s, 3H), 2.18 – 2.11 (m, 2H), 1.52 – 1.28 (m, 6H). ^{13}**C NMR** (126 MHz, $CDCl_3$): δ (ppm) 143.4, 142.9 (t, *J* = 8.7 Hz), 139.1, 135.2, 129.4, 129.0, 128.7, 127.9, 127.7, 119.0 (qt, *J* = 285.3, 38.6 Hz), 116.6 (t, *J* = 23.1 Hz), 112.2 (tq, *J* = 249.7, 38.2 Hz), 50.1, 31.7, 27.8, 27.4, 25.7, 21.4. ^{9}**F NMR** (471 MHz, $CDCl_3$): δ (ppm) -85.40 (s, 3F), -115.04 (d, *J* = 11.5, 2F). **HRMS** (ESI) m/z: $[M+Na]^+$ Calcd for $C_{22}H_{24}F_5NO_2SNa$ 484.1340; Found 484.1343.

(Z)-2-(3,4,4,4-tetrafluoro-2-(naphthalen-2-yl)but-2-en-1-yl)-2,3-dihydro-1H-naphtho[1,8-de][1,3,2]diazaborinine (2a)

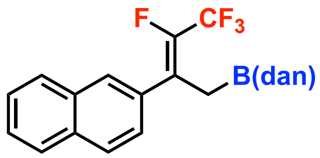

Following the general procedure (VIII) at 0.2 mmol scale, using alkene **1a** as starting material, **Appearance:** colorless oil; **Yield:** 73.1 mg, 87%**;** R_f = 0.30 (EA: Hexane = 1:25), *Z*/*E* > 99:1 (^{19}F NMR). **^{1}H NMR** (500 MHz, $CDCl_3$) δ 7.92 – 7.88 (m, 2H), 7.88 – 7.84 (m, 2H), 7.59 – 7.47 (m, 3H), 7.09 (t, *J* = 7.7 Hz, 2H), 7.04 (d, *J* = 8.3 Hz, 2H), 6.24 (d, *J* = 7.2 Hz, 2H), 5.54 (s, 2H), 2.37 (s, 2H). **^{19}F NMR** (471 MHz, $CDCl_3$) δ -64.1 (d, *J* = 8.3 Hz, 3F), -130.0 (q, *J* = 8.7 Hz, 1F). **^{11}B NMR** (160 MHz, $CDCl_3$) δ 30.05. **^{13}C NMR** (126 MHz, $CDCl_3$) δ 141.1 (dq, *J* = 251.7, 37.6 Hz), 140.5, 136.2, 133.3, 133.2, 133.1, 128.6, 128.4, 127.8, 127.6, 127.5 (d, *J* = 3.5 Hz), 127.0, 126.8, 126.2 (dq, *J* = 9.4, 2.7 Hz), 125.4 (d, *J* = 3.6 Hz), 120.4 (qd, J = 273.1, 42.3 Hz), 119.7, 118.1, 106.1, 18.8. **HRMS** (ESI) m/z: $[M]^+$ Calcd for $C_{24}H_{17}BF_4N_2$ 420.1420; Found 420.1414.

(*Z*)-2-(2-([1,1'-biphenyl]-4-yl)-3,4,4,4-tetrafluorobut-2-en-1-yl)-2,3-dihydro-1*H*-naphtho[1,8-*de*][1,3,2]diazaborinine (2b)

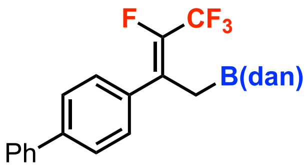

Following the general procedure (VIII) at 0.2 mmol scale, using alkene **1b** as starting material, **Appearance:** yellow oil; **Yield:** 75.8 mg, 85%**;** R_f = 0.30 (EA: Hexane = 1:25), *Z*/*E* > 99:1 (^{19}F NMR). **^{1}H NMR** (400 MHz, $CDCl_3$) δ 7.74 – 7.61 (m, 4H), 7.57 – 7.46 (m, 4H), 7.43 (t, *J* = 7.3 Hz, 1H), 7.21 – 7.05 (m, 4H), 6.30 (d, *J* = 7.1 Hz, 2H), 5.57 (s, 2H), 2.32 (s, 2H). **^{19}F NMR** (471 MHz, $CDCl_3$) δ -64.0 (t, *J* = 6.6 Hz), -130.0 (q, *J* = 7.7 Hz). **^{11}B NMR** (160 MHz, $CDCl_3$) δ 30.31. **^{13}C NMR** (126 MHz, $CDCl_3$) δ 141.8, 140.9 (dq, *J* = 251.2, 37.8 Hz), 140.5, 140.2, 136.3, 134.7, 129.0, 128.5, 128.5, 127.9, 127.6, 127.5, 127.2, 120.3 (qd, *J* = 273.3, 42.5 Hz), 118.2, 106.2, 18.5. **HRMS** (ESI) m/z: $[M-H]^+$ Calcd for $C_{26}H_{18}BF_4N_2$ 445.1509; Found 445.1506

(*Z*)-2-(3,4,4,4-tetrafluoro-2-(4-methoxyphenyl)but-2-en-1-yl)-2,3-dihydro-1*H*-naphtho[1,8-*de*][1,3,2]diazaborinine (2c)

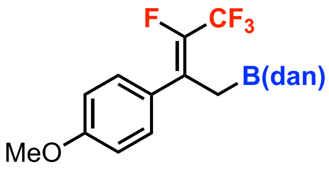

Following the general procedure (VIII) at 0.2 mmol scale, using alkene **1c** as starting material, **Appearance:** yellow oil; **Yield:** 60.0 mg, 75%**;** R_f = 0.30 (EA: Hexane = 1:5), *Z*/*E* > 99:1 (^{19}F NMR). **^{1}H NMR** (400 MHz, $CDCl_3$) δ 7.40 (d, *J* = 7.4 Hz, 2H), 7.13 (t, *J* = 7.7 Hz, 2H), 7.07 (d, *J* = 8.3 Hz, 2H), 6.97 (d, *J* = 8.8 Hz, 2H), 6.28 (d, *J* = 7.2 Hz, 2H), 5.53 (s, 2H), 3.85 (s, 3H), 2.26 (s, 2H). **^{19}F NMR** (471 MHz, $CDCl_3$) δ -64.3 (d, *J* = 8.1 Hz, 3F), -128.3 (q, *J* = 8.4 Hz, 1F). **^{11}B NMR** (128 MHz, $CDCl_3$) δ 29.93. **^{13}C NMR** (101 MHz, $CDCl_3$) δ 160.0, 140.6, 140.5 (dq, *J* = 250.4, 37.4 Hz), 136.3, 129.5 (d, *J* = 4.1 Hz), 127.9, 127.6, 125.4 (dq, *J* = 8.5, 2.7 Hz), 120.4 (qd, *J* = 272.8, 42.5 Hz), 119.7, 118.1, 114.2, 106.1, 55.4, 18.4. **HRMS** (ESI) m/z: $[M+H]^+$ Calcd for $C_{21}H_{18}BF_4N_2O$ 401.1447; Found 401.1442

methyl (Z)-4-(3,4,4,4-tetrafluoro-1-(1H-naphtho[1,8-de][1,3,2]diazaborinin-2(3H)-yl)but-2-en-2-yl)benzoate (2d)

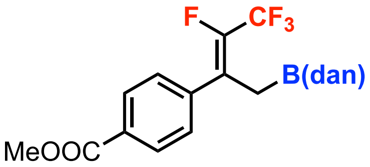

Following the general procedure (VIII) at 0.2 mmol scale, using alkene **1d** as starting material, **Appearance:** yellow oil; **Yield:** 58.2 mg, 68%**;** R_f = 0.30 (EA: Hexane = 1:5), *Z*/*E* > 99:1 (^{19}F NMR). ^{1}**H NMR** (500 MHz, $CDCl_3$) δ 8.08 (d, *J* = 8.6 Hz, 2H), 7.47 (d, *J* = 8.0 Hz, 2H), 7.09 (t, *J* = 7.7 Hz, 2H), 7.04 (d, *J* = 8.2 Hz, 2H), 6.26 (d, *J* = 7.2 Hz, 2H), 5.51 (s, 2H), 3.93 (s, 3H), 2.26 (s, 2H). ^{19}**F NMR** (471 MHz, $CDCl_3$) δ -64.3 (d, *J* = 8.3 Hz, 3F), -128.4 (q, *J* = 7.6 Hz, 2F). ^{11}**B NMR** (160 MHz, $CDCl_3$) δ 30.64. ^{13}**C NMR** (126 MHz, $CDCl_3$) δ 166.5, 141.3 (dq, *J* = 253.6, 37.8 Hz), 140.5, 140.3, 136.3, 130.5, 130.1, 128.1 (d, *J* = 3.6 Hz), 127.6, 125.4 (dq, *J* = 9.2, 2.8 Hz), 120.0 (qd, *J* = 273.1, 41.5 Hz), 119.7, 118.3, 106.2, 52.4, 18.6. **HRMS** (ESI) m/z: $[M+Cl]^+$ Calcd for $C_{22}H_{17}BF_4N_2O_2Cl$ 463.1017; Found 463.1019

(*Z*)-2-(3,4,4,4-tetrafluoro-2-(4-(methylthio)phenyl)but-2-en-1-yl)-2,3-dihydro-1*H*-naphtho[1,8-*de*][1,3,2]diazaborinine (2e)

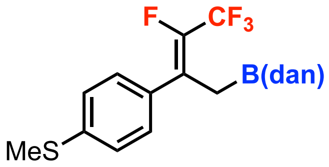

Following the general procedure (VIII) at 0.2 mmol scale, using alkene **1e** as starting material, **Appearance:** brown liquid; **Yield:** 57.4 mg, 69%**;** R_f = 0.30 (EA: Hexane = 1:5), *Z*/*E* > 99:1 (^{19}F NMR). ^{1}**H NMR** (500 MHz, $CDCl_3$) δ 7.39 (d, *J* = 8.1 Hz, 2H), 7.32 (d, *J* = 8.2 Hz, 2H), 7.15 (t, *J* = 7.7 Hz, 3H), 7.09 (d, *J* = 8.2 Hz, 3H), 6.31 (d, *J* = 7.3 Hz, 2H), 5.57 (s, 2H), 2.53 (s, 3H). ^{19}**F NMR (**471 MHz, $CDCl_3$) δ -63.93 (d, *J* = 8.4 Hz, 3F), -130.28 (q, *J* = 8.6 Hz, 1F).^{11}**B NMR** (160 MHz, $CDCl_3$) δ 30.63. ^{13}**C NMR** (126 MHz, $CDCl_3$) δ 140.8 (dq, *J* = 251.7, 37.5 Hz), 140.4, 140.1, 136.2, 132.1, 128.4 (d, *J* = 4.0 Hz), 127.6, 126.2, 125.4 (dt, *J* = 9.6, 2.5 Hz), 120.3 (qd, *J* = 273.1, 42.5 Hz), 119.6, 118.1, 106.1, 18.3, 15.3. **HRMS** (ESI) m/z: $[M]^+$ Calcd for $C_{21}H_{17}BF_4N_2S$ 416.1140; Found 416.1134

(Z)-2-(2-(4-chlorophenyl)-3,4,4,4-tetrafluorobut-2-en-1-yl)-2,3-dihydro-1H-naphtho[1,8-de][1,3,2]diazaborinine (2f)

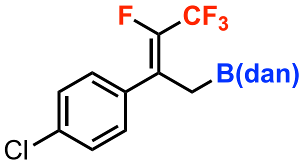

Following the general procedure (VIII) at 0.2 mmol scale, using alkene **1f** as starting material, **Appearance:** yellow solid; **Yield:** 60.7 mg, 75%**;** R_f = 0.30 (EA: Hexane = 1:5), *Z*/*E* > 99:1 (^{19}F NMR). ^{1}**H NMR** (400 MHz, $CDCl_3$) δ 7.41 (d, *J* = 8.3 Hz, 2H), 7.35 (d, *J* = 8.2 Hz, 2H), 7.13 (t, *J* = 7.8 Hz, 2H), 7.08 (d, *J* = 8.2 Hz, 2H), 6.29 (d, *J* = 7.2 Hz, 2H), 5.51 (s, 2H), 2.24 (s, 2H). ^{11}**B NMR** (128 MHz, $CDCl_3$) δ 29.84. ^{19}**F NMR** (471 MHz, $CDCl_3$) δ -64.16 (d, *J* = 7.9 Hz), -125.45 – -132.27 (m). ^{13}**C NMR** (101 MHz, $CDCl_3$) δ 141.1 (dq, *J* = 252.6, 37.7 Hz), 140.3, 136.3, 135.0, 134.3, 129.4 (d, *J* = 3.6 Hz), 129.1, 127.6, 125.1 (d, *J* = 9.0 Hz), 120.1 (qd, *J* = 272.9, 42.0 Hz), 119.7, 118.3, 106.2, 18.6. **HRMS** (ESI) m/z: $[M]^+$ Calcd for $C_{20}H_{14}BClF_4N_2$ 404.0873; Found 404.0870

(Z)-2-(2-(4-bromophenyl)-3,4,4,4-tetrafluorobut-2-en-1-yl)-2,3-dihydro-1H-naphtho[1,8-de][1,3,2]diazaborinine (2g)

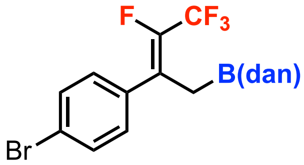

Following the general procedure (VIII) at 0.2 mmol scale, using alkene **1g** as starting material, **Appearance:** yellow oil; **Yield:** 52.9 mg, 59%**;** R_f = 0.30 (EA: Hexane = 1:5), *Z*/*E* > 99:1 (^{19}F NMR). **^{1}H NMR** (500 MHz, $CDCl_3$) δ 7.58 (d, *J* = 8.3 Hz, 2H), 7.31 (d, *J* = 8.2 Hz, 2H), 7.14 (t, *J* = 7.7 Hz, 2H), 7.09 (d, *J* = 8.1 Hz, 2H), 6.31 (d, *J* = 7.2 Hz, 2H), 5.53 (s, 2H), 2.25 (t, *J* = 2.6 Hz, 2H). **^{19}F NMR** (471 MHz, $CDCl_3$) δ -64.2 (d, *J* = 8.1 Hz), -129.1 (q, *J* = 8.5 Hz). **^{11}B NMR** (160 MHz, $CDCl_3$) δ 30.52. **^{13}C NMR** (126 MHz, $CDCl_3$) δ 141.2 (dd, *J* = 252.7 Hz, = 37.6 Hz), 140.3, 136.3, 134.8, 132.1, 129.6 (d, *J* = 3.7 Hz), 127.7, 125.5 – 124.7 (m), 123.2, 120.1 (dq, *J* = 273.1, 42.3 Hz), 119.7, 118.3, 106.2, 18.6. **HRMS** (ESI) m/z: $[M+Cl]^+$ Calcd for $C_{20}H_{14}BBrF_4N_2Cl$ 485.0046; Found 485.0042

(*Z*)-4'-(3,4,4,4-tetrafluoro-1-(1*H*-naphtho[1,8-*de*][1,3,2]diazaborinin-2(3*H*)-yl)but-2-en-2-yl)-[1,1'-biphenyl]-2-carbonitrile (2h)

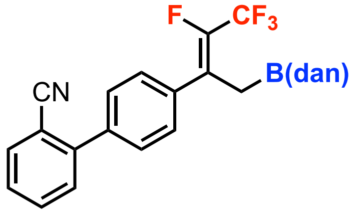

Following the general procedure (VIII) at 0.2 mmol scale, using alkene **1h** as starting material, **Appearance:** yellow solid; **Yield:** 70.7 mg, 75%**;** R_f = 0.30 (EA: Hexane = 1:5), *Z*/*E* > 99:1 (^{19}F NMR). **^{1}H NMR** (500 MHz, $CDCl_3$) δ 7.78 (d, *J* = 7.8 Hz, 1H), 7.66 (td, *J* = 7.7, 1.3 Hz, 1H), 7.61 (d, *J* = 8.3 Hz, 2H), 7.53 (t, *J* = 7.6 Hz, 3H), 7.47 (td, *J* = 7.6, 1.2 Hz, 1H), 7.13 – 7.06 (m, 2H), 7.03 (d, *J* = 8.4 Hz, 2H), 6.29 (d, *J* = 7.2 Hz, 2H), 5.55 (s, 2H), 2.30 (t, *J* = 2.6 Hz, 2H). **^{19}F NMR** (471 MHz, $CDCl_3$) δ -64.20 (d, *J* = 7.9 Hz, 3F), -129.31 (q, *J* = 8.4 Hz, 1F). **^{11}B NMR** (160 MHz, $CDCl_3$) δ 30.72. **^{13}C NMR** (126 MHz, $CDCl_3$) δ 144.5, 140.5, 141.15 (dq, *J* = 252.7, 37.7 Hz), 138.7, 136.4, 136.3, 134.0, 133.1, 130.1, 129.2, 128.5 (d, *J* = 3.7 Hz), 128.1, 127.7, 125.45 (dd, *J* = 8.8, 2.7 Hz), 120.19 (qd, *J* = 273.3, 42.1 Hz), 119.7, 118.7, 118.2, 111.3, 106.2, 18.6. **HRMS** (ESI) m/z: $[M+Cl]^+$ Calcd for $C_{27}H_{18}BF_4N_3Cl$ 506.1225; Found 506.1229

(*Z*)-2-(2-(benzo[*d*][1,3]dioxol-5-yl)-3,4,4,4-tetrafluorobut-2-en-1-yl)-2,3-dihydro-1*H*-naphtho[1,8-*de*][1,3,2]diazaborinine (2i)

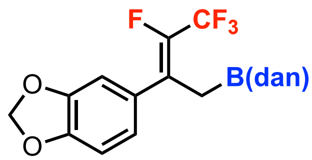

Following the general procedure (VIII) at 0.2 mmol scale, using alkene **1i** as starting material, **Appearance:** pink oil; **Yield:** 62.9 mg, 76%**;** R_f = 0.30 (EA: Hexane = 1:5), *Z*/*E* > 99:1 (^{19}F NMR). **^{1}H NMR** (500 MHz, $CDCl_3$) δ 7.11 (t, *J* = 7.7 Hz, 2H), 7.05 (d, *J* = 8.1 Hz, 2H), 6.92 (d, *J* = 8.8 Hz, 2H), 6.86 (d, *J* = 8.0 Hz, 1H), 6.28 (d, *J* = 7.2 Hz, 2H), 6.00 (s, 2H), 5.53 (s, 2H), 2.22 (s, 2H). **^{19}F NMR** (471 MHz, $CDCl_3$) δ -63.92, -130.34 – -130.45 (m). **^{11}B NMR** (160 MHz, $CDCl_3$) δ 31.14. **^{13}C NMR** (126 MHz, $CDCl_3$) δ 148.1, 148.0, 140.8 (dq, J = 251.1, 37.5 Hz), 140.5, 136.3,

129.3, 127.6, 125.6 (dt, *J* = 8.9, 2.7 Hz), 122.0, 121.9, 120.3 (qd, J = 272.8, 42.3 Hz), 119.7, 118.1, 108.6, 106.1, 101.6, 18.7. **HRMS** (ESI) m/z: $[M]^+$ Calcd for $C_{21}H_{15}BF_4N_2O_2$ 414.1161; Found 414.1155

(*Z*)-2-(2-(benzofuran-2-yl)-3,4,4,4-tetrafluorobut-2-en-1-yl)-2,3-dihydro-1*H*-naphtho[1,8-*de*][1,3,2]diazaborinine (2j)

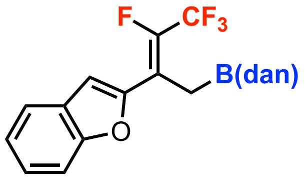

Following the general procedure (VIII) at 0.2 mmol scale, using alkene **1j** as starting material, **Appearance:** yellow solid; **Yield:** 61.5 mg, 75%**;** R_f = 0.30 (EA: Hexane = 1:5), *Z*/*E* > 99:1 (^{19}F NMR).. ^{1}**H NMR** (500 MHz, $CDCl_3$) δ 7.67 (d, *J* = 7.8 Hz, 1H), 7.54 (d, *J* = 8.3 Hz, 1H), 7.39 (t, *J* = 7.8 Hz, 1H), 7.35 – 7.27 (m, 2H), 7.13 (t, *J* = 7.6 Hz, 2H), 7.07 (d, *J* = 8.2 Hz, 2H), 6.31 (d, *J* = 7.3 Hz, 2H), 5.71 (s, 2H), 2.44 (s, 2H). ^{19}**F NMR** (471 MHz, $CDCl_3$) δ -63.43 (d, *J* = 7.6 Hz, 3F), -121.33 (q, *J* = 8.5 Hz, 2F). ^{11}**B NMR** (160 MHz, $CDCl_3$) δ 30.79. ^{13}**C NMR** (126 MHz, $CDCl_3$) δ 154.3, 149.0 (t, *J* = 2.8 Hz), 142.1 (dd, *J* = 264.1, 37.8 Hz), 140.6, 136.3 (d, *J* = 2.3 Hz), 128.4, 127.6 (d, *J* = 2.2 Hz), 126.0 (d, *J* = 2.1 Hz), 123.7 (d, *J* = 2.1 Hz), 122.0 (d, *J* = 2.2 Hz), 120.2 (dd, *J* = 272.6, 41.8 Hz), 119.7 (d, *J* = 2.2 Hz), 118.1 (d, *J* = 2.2 Hz), 116.4 – 115.8 (m), 111.5 (d, *J* = 2.1 Hz), 110.5 (d, *J* = 16.8 Hz), 106.1 (d, *J* = 2.1 Hz), 13.4. **HRMS** (ESI) m/z: $[M]^+$ Calcd for $C_{22}H_{15}BF_4N_2O$ 410.1212; Found 410.1207

(*Z*)-2-(2-(benzo[*b*]thiophen-2-yl)-3,4,4,4-tetrafluorobut-2-en-1-yl)-2,3-dihydro-1*H*-naphtho[1,8-*de*][1,3,2]diazaborinine (2k)

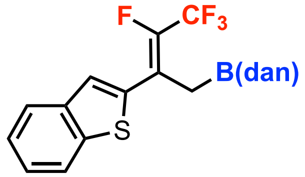

Following the general procedure (VIII) at 0.2 mmol scale, using alkene **1k** as starting material, **Appearance:** white solid; **Yield:** 60.5 mg, 71%**;** R_f = 0.30 (EA: Hexane = 1:5), *Z*/*E* > 99:1 (^{19}F NMR). ^{1}**H NMR** (500 MHz, $CDCl_3$) δ 7.92 – 7.76 (m, 2H), 7.66 (s, 1H), 7.42 – 7.35 (m, 2H), 7.12 (t, *J* = 7.8 Hz, 2H), 7.07 (d, *J* = 8.3 Hz, 2H), 6.31 (d, *J* = 7.3 Hz, 2H), 5.65 (s, 2H), 2.44 (s, 2H). ^{11}**B NMR** (128 MHz, $CDCl_3$) δ 30.96. ^{19}**F NMR** (471 MHz, $CDCl_3$) δ -63.00 (d, *J* = 7.3 Hz, 3F), -121.63 (q, *J* = 7.7 Hz, 2F). ^{13}**C NMR** (126 MHz, $CDCl_3$) δ 141.9 (dq, *J* = 261.9, 37.6 Hz), 140.5, 140.4, 138.8, 136.7 (d, *J* = 5.2 Hz), 136.3, 127.6, 126.0 (d, *J* = 7.6 Hz), 125.9, 124.9, 124.5, 122.1, 120.4 (*J* = 273.9, 41.0 Hz), 119.7, 119.0, 118.9, 118.2, 17.0. **HRMS** (ESI) m/z: $[M]^+$ Calcd for $C_{22}H_{15}BF_4N_2S$ 426.0984; Found 426.0979

(*Z*)-2-(3,4,4,4-tetrafluoro-2-(quinolin-3-yl)but-2-en-1-yl)-2,3-dihydro-1*H*-naphtho[1,8-*de*][1,3,2]diazaborinine (2l)

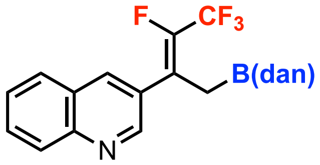

Following the general procedure (VIII) at 0.2 mmol scale, using alkene **1l** as starting material, **Appearance:** yellow solid; **Yield:** 56.4 mg, 67%**;** R_f = 0.30 (EA: Hexane = 1:5), *Z*/*E* > 99:1 (^{19}F NMR). ^{1}**H NMR** (500 MHz, $CDCl_3$) δ 8.98 (t, *J* = 2.0 Hz, 1H), 8.18 (d, *J* = 2.3 Hz, 1H), 8.12 (d, *J* = 8.5 Hz, 1H), 7.80 (dd, *J* = 8.3, 1.4 Hz, 1H), 7.78 – 7.72 (m, 1H), 7.60 – 7.53 (m, 1H), 7.12 – 7.01 (m, 4H), 6.28 (dd, *J* = 7.2, 1.2 Hz, 2H), 5.72 (s, 2H), 2.36 (s, 2H). ^{19}**F NMR**

(471 MHz, $CDCl_3$) δ -64.08 (d, *J* = 8.0 Hz), -128.09 (d, *J* = 8.5 Hz). **^{11}B NMR** (160 MHz, $CDCl_3$) δ 30.45. **^{13}C NMR** (126 MHz, $CDCl_3$) δ 149.2 (d, *J* = 4.7 Hz), 147.7, 140.3, 136.2, 135.4 (d, *J* = 3.6 Hz), 130.7, 129.2, 129.0, 128.3, 127.6, 127.5, 127.4, 123.2 (dd, *J* = 9.1, 2.7 Hz), 120.0 (qd, *J* = 273.3, 42.1 Hz), 118.3, 106.3, 18.5. **HRMS** (ESI) m/z: $[M+H]^+$ Calcd for $C_{23}H_{17}BF_4N_3$ 422.1450; Found 422.1443

2-((*Z*)-3,4,4,4-tetrafluoro-2-(4-((*E*)-styryl)phenyl)but-2-en-1-yl)-2,3-dihydro-1*H*-naphtho[1,8-*de*][1,3,2]diazaborinine (2m)

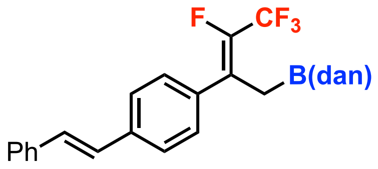

Following the general procedure (VIII) at 0.2 mmol scale, using alkene **1m** as starting material, **Appearance:** yellow oil; **Yield:** 64.2 mg, 68%**;** R_f = 0.30 (EA: Hexane = 1:5), *Z*/*E* > 99:1 (^{19}F NMR). **^{1}H NMR** (500 MHz, $CDCl_3$) δ 7.56 (t, *J* = 8.8 Hz, 4H), 7.43 (d, *J* = 8.1 Hz, 2H), 7.40 (t, *J* = 7.6 Hz, 2H), 7.31 (t, *J* = 7.3 Hz, 1H), 7.19 – 7.09 (m, 4H), 7.06 (d, *J* = 8.2 Hz, 2H), 6.27 (d, *J* = 7.2 Hz, 2H), 5.53 (s, 2H), 2.36 – 2.13 (m, 2H). **^{19}F NMR** (471 MHz, $CDCl_3$) δ -64.0 (d, *J* = 8.0 Hz, 3F), -130.0 (q, *J* = 8.4 Hz, 1F). **^{11}B NMR** (160 MHz, $CDCl_3$) δ 31.8. **^{13}C NMR** (126 MHz, $CDCl_3$) δ 140.9 (dq, *J* = 252.0, 37.4 Hz), 140.5, 138.1, 137.1, 136.3, 134.9, 130.1, 128.9, 128.5 (d, *J* = 4.0 Hz), 128.1, 127.7, 127.6, 126.8, 126.8, 125.6 (dd, *J* = 9.0, 2.8 Hz), 120.3 (dq, *J* = 273.0, 42.4 Hz), 119.7, 118.2, 106.2, 18.6. **HRMS** (ESI) m/z: $[M]^+$ Calcd for $C_{28}H_{21}BF_4N_2$ 472.1733; Found 472.1730

(*Z*)-2-(5,6,6,6-tetrafluoro-1-phenylhex-4-en-3-yl)-2,3-dihydro-1*H*-naphtho[1,8-*de*][1,3,2]diazaborinine (4a)

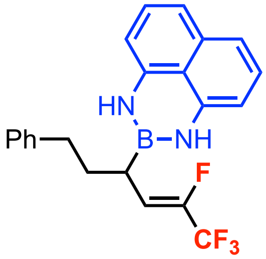

Following the general procedure (IX) at 0.2 mmol scale, using alkene **3a** (*E*/*Z* = 88:12) as starting material, **Appearance:** colorless oil; **Yield:** 61.3 mg, 77%**;** R_f = 0.25 (EA: Hexane = 1:50), *Z*/*E* = 99:1 (^{19}F NMR)**. ^{1}H NMR** (500 MHz, $CDCl_3$) δ 7.42 (t, *J* = 7.5 Hz, 2H), 7.33 (t, *J* = 7.5 Hz, 1H), 7.31 – 7.25 (d, *J* = 7.2 Hz, 2H), 7.21 (t, *J* = 7.8 Hz, 2H), 7.15 (d, *J* = 8.3 Hz, 2H), 6.39 (d, *J* = 7.3 Hz, 2H), 5.66 (dd, *J* = 22.9 Hz, 10.9 Hz, 1H), 5.61 (s, 2H), 2.94 – 2.75 (m, 1H), 2.75 – 2.60 (m, 1H), 2.42 (td, *J* = 10.5, 5.2 Hz, 1H), 2.13 – 1.97 (m, 1H), 1.97 – 1.82 (m, 1H). **^{19}F NMR** (471 MHz, $CDCl_3$) δ -71.61 (d, *J* = 11.6 Hz, 3F), -135.89 (dq, *J* = 35.0, 12.1 Hz, 1F). **^{11}B NMR** (128 MHz, $CDCl_3$) δ 30.33. **^{13}C NMR** (126 MHz, $CDCl_3$) δ 145.7 (dq, *J* = 253.4, 38.7 Hz), 141.2, 140.4, 136.3, 128.6, 128.5, 127.7, 126.3, 119.8, 118.7 (qd, *J* = 270.9, 42.2 Hz), 118.3, 115.4 (dq, *J* = 9.8, 3.2 Hz), 106.2, 35.3, 32.4, 23.1. **HRMS** (ESI) m/z: $[M]^+$ Calcd for $C_{22}H_{19}BF_4N_2$ 398.1576; Found 398.1574.

(*Z*)-7,8,8,8-tetrafluoro-5-(1*H*-naphtho[1,8-*de*][1,3,2]diazaborinin-2(3*H*)-yl)oct-6-en-1-yl thiophene-3-carboxylate (4b)

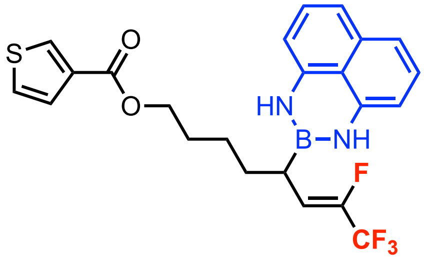

Following the general procedure (IX) at 0.2 mmol scale, using alkene **3b** (*E*/*Z* = 87:13) as starting material, **Appearance:** colorless oil; **Yield:** 59.0 mg, 62%**;** R_f = 0.23 (EA: Hexane = 1:10), *Z*/*E* = 98:2 (^{19}F NMR)**.** ^{1}**H NMR** (500 MHz, $CDCl_3$) δ 8.08 (dd, *J* = 3.0, 1.2 Hz, 1H), 7.50 (dd, *J* = 5.0, 1.2 Hz, 1H), 7.27 (dd, *J* = 5.1, 3.1 Hz, 1H), 7.15 – 7.08 (m, 2H), 7.05 (d, *J* = 8.2 Hz, 2H), 6.34 (dd, *J* = 7.3, 1.0 Hz, 2H), 5.62 (s, 2H), 5.55 (dd, *J* = 33.6, 10.9 Hz, 1H), 4.42 – 4.21 (m, 2H), 2.34 (td, *J* = 9.9, 5.7 Hz, 1H), 1.86 – 1.74 (m, 3H), 1.68 – 1.44 (m, 3H). ^{19}**F NMR** (471 MHz, $CDCl_3$) δ -71.73 (d, *J* = 11.6 Hz, 3F), -136.37 (dq, *J* = 34.2, 11.9 Hz, 1F). ^{11}**B NMR** (160 MHz, $CDCl_3$) δ 30.99. ^{13}**C NMR** (126 MHz, $CDCl_3$) δ 163.0, 145.7 (dq, *J* = 253.5, 38.6 Hz), 140.5, 136.3, 133.8, 132.8, 127.9, 127.7, 126.2, 119.8, 118.7 (dd, *J* = 271.3, 42.4 Hz), 118.3, 115.3 (dd, *J* = 9.8, 3.3 Hz), 106.3, 64.3, 30.2, 28.6, 25.6, 23.5. **HRMS** (ESI) m/z: $[M+Na]^+$Calcd for $C_{23}H_{21}BF_4N_2O_2SNa$ 499.1249; Found 499.1246.

(*Z*)-12,13,13,13-tetrafluoro-10-(1*H*-naphtho[1,8-*de*][1,3,2]diazaborinin-2(3*H*)-yl)tridec-11-en-1-yl benzoate (4c)

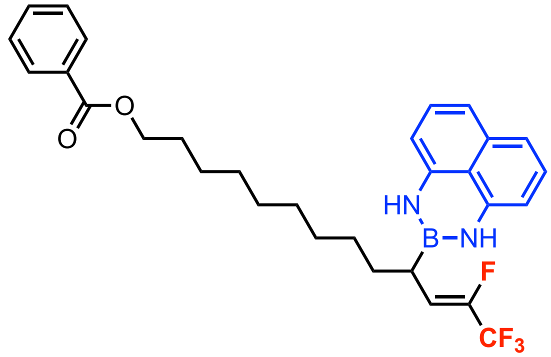

Following the general procedure (IX) at 0.2 mmol scale, using alkene **3c** (*E*/*Z* = 90:10) as starting material, **Appearance:** colorless oil; **Yield:** 88.5 mg, 82%**;** R_f = 0.20 (EA: Hexane = 1:50), *Z*/*E* > 99:1 (^{19}F NMR). ^{1}**H NMR** (500 MHz, $CDCl_3$) δ 8.06 (d, *J* = 7.7 Hz, 2H), 7.56 (t, *J* = 7.4 Hz, 1H), 7.44 (t, *J* = 7.6 Hz, 2H), 7.12 (t, *J* = 7.7 Hz, 2H), 7.05 (d, *J* = 8.2 Hz, 2H), 6.35 (d, *J* = 7.3 Hz, 2H), 5.62 (s, 2H), 5.55 (dd, *J* = 33.8, 10.9 Hz, 1H), 4.33 (t, *J* = 6.7 Hz, 2H), 2.32 (td, *J* = 10.2, 5.7 Hz, 1H), 1.77 (p, *J* = 7.0 Hz, 2H), 1.68 – 1.57 (m, 1H), 1.55 – 1.49 (m, 1H), 1.48 – 1.41 (m, 3H), 1.40 – 1.28 (m, 9H). ^{19}**F NMR** (471 MHz, $CDCl_3$) δ -71.71 (d, *J* = 12.0 Hz, 3F), -136.89 (dq, *J* = 34.9, 12.0 Hz, 1F). ^{11}**B NMR** (160 MHz, $CDCl_3$) δ 31.15. ^{13}**C NMR** (126 MHz, $CDCl_3$) δ 166.9, 145.5 (dq, *J* = 253.1, 38.8 Hz), 140.6, 136.3, 133.0, 130.6, 129.7, 128.5, 127.7, 119.8, 118.7 (dq, *J* = 270.8, 42.3 Hz), 118.2, 115.7 (dq, *J* = 9.3, 3.0 Hz), 106.2, 65.2, 30.7, 29.5, 29.5, 29.4, 29.3, 29.2, 28.8, 26.1, 23.7. **HRMS** (ESI) m/z: $[M+Na]^+$ Calcd for $C_{30}H_{33}BF_4N_2O_2Na$ 563.2469; Found 563.2460.

(*Z*)-4-methyl-7-((7,8,8,8-tetrafluoro-5-(1*H*-naphtho[1,8-*de*][1,3,2]diazaborinin-2(3*H*)-yl)oct-6-en-1-yl)oxy)-2*H*-chromen-2-one (4d)

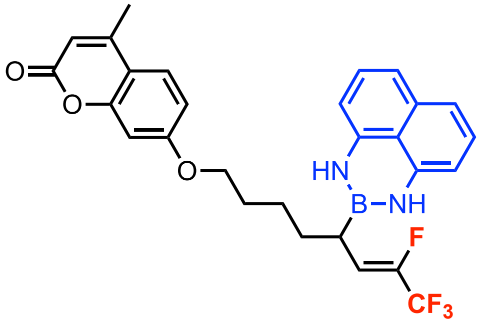

Following the general procedure (IX) at 0.2 mmol scale, using alkene **3d** (*E*/*Z* = 86:14) as starting material, **Appearance:** yellow oil; **Yield:** 64.9 mg, 62%**;** R_f = 0.24 (EA: Hexane = 1:5), *Z*/*E* = 95:5 (^{19}F NMR). **^{1}H NMR** (500 MHz, $CDCl_3$) δ 7.47 (d, *J* = 8.8 Hz, 1H), 7.11 (t, *J* = 7.8 Hz, 2H), 7.04 (d, *J* = 8.2 Hz, 2H), 6.83 (dd, *J* = 8.8, 2.5 Hz, 1H), 6.80 – 6.76 (m, 1H), 6.34 (d, *J* = 7.2 Hz, 2H), 6.13 (d, *J* = 1.4 Hz, 1H), 5.69 – 5.47 (m, 3H), 4.03 (t, *J* = 6.3 Hz, 2H), 2.39 (d, *J* = 1.3 Hz, 3H), 1.94 – 1.79 (m, 2H), 1.79 – 1.69 (m, 2H), 1.67 – 1.56 (m, 1H), 1.56 – 1.46 (m, 2H). **^{11}B NMR** (160 MHz, $CDCl_3$) δ 31.58. **^{19}F NMR** (471 MHz, $CDCl_3$) δ -71.67 (d, *J* = 12.0 Hz), -136.55 (dq, *J* = 34.8, 11.9 Hz). **^{13}C NMR** (126 MHz, $CDCl_3$) δ 162.1, 161.5, 155.3, 152.8 (d, *J* = 2.2 Hz), 145.6 (dq, *J* = 252.6, 38.3 Hz), 140.5 (d, *J* = 2.2 Hz), 136.3, 127.6, 125.6, 119.8, 118.7 (qd, *J* = 270.8, 42.3 Hz), 118.2, 115.4 (dq, *J* = 6.1, 3.4 Hz), 113.6, 112.8, 111.9, 106.2, 101.3, 68.2, 30.3, 28.8, 25.7, 23.6, 18.7. **HRMS** (ESI) m/z: $[M+Na]^+$ Calcd for $C_{28}H_{25}BF_4N_2O_3Na$ 547.1792; Found 547.1788

(8*R*,9*S*,13*S*,14*S*)-13-methyl-3-(((*Z*)-7,8,8,8-tetrafluoro-5-(1*H*-naphtho[1,8-*de*][1,3,2]diazaborinin-2(3*H*)-yl)oct-6-en-1-yl)oxy)-6,7,8,9,11,12,13,14,15,16-decahydro-17*H*-cyclopenta[*a*]phenanthren-17-one (4e)

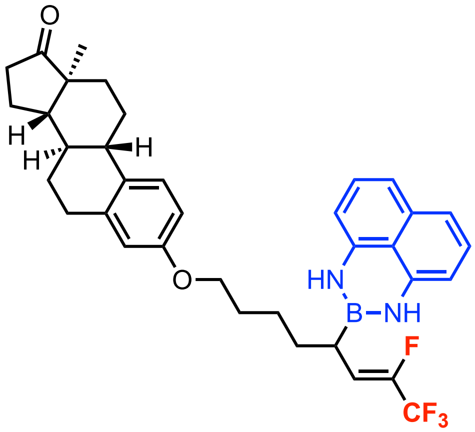

Following the general procedure (IX) at 0.2 mmol scale, using alkene **3e** (*E*/*Z* = 88:12) as starting material, **Appearance:** white solid; **Yield:** 71.7 mg, 58%**;** R_f = 0.24 (EA: Hexane = 1:5), *Z*/*E* = 95:5 (^{19}F NMR)**. ^{1}H NMR** (500 MHz, $CDCl_3$) δ 7.20 (d, *J* = 8.6 Hz, 1H), 7.12 (t, *J* = 7.8 Hz, 2H), 7.05 (d, *J* = 8.2 Hz, 2H), 6.74 (d, *J* = 10.2 Hz, 1H), 6.66 (s, 1H), 6.34 (d, *J* = 7.3 Hz, 2H), 5.64 (s, 2H), 5.57 (dd, *J* = 33.6, 10.9 Hz, 1H), 3.97 (t, *J* = 6.3 Hz, 2H), 2.91 – 2.88 (m, 2H), 2.57 – 2.45 (m, 2H), 2.43 – 2.32 (m, 2H), 2.30 – 2.21 (m, 1H), 2.21 – 2.10 (m, 1H), 2.09 – 1.94 (m, 3H), 1.89 – 1.78 (m, 2H), 1.77 – 1.69 (m, 1H), 1.66 – 1.55 (m, 4H), 1.56 – 1.45 (m, 4H), 0.92 (s, 3H). **^{19}F NMR** (471 MHz, $CDCl_3$) δ -71.7 (d, *J* = 11.7 Hz, 3F), -136.5 (dq, *J* = 34.7, 12.1 Hz, 1F). **^{11}B NMR** (160 MHz, $CDCl_3$) δ 31.62. **^{13}C NMR** (126 MHz, $CDCl_3$) δ 221.2, 162.1, 161.5, 155.3, 152.8 (d, *J* = 2.2 Hz), 145.6 (dq, *J* = 252.8, 38.6 Hz), 140.5 (d, *J* = 2.2 Hz), 136.3, 127.6, 125.6, 119.8, 118.7 (qd, *J* = 271.1, 42.1 Hz), 118.2, 115.7 – 114.8 (m), 113.6, 112.8, 111.9, 106.2, 101.3, 68.2, 60.5, 41.4, 36.2, 34.8, 34.6, 31.1, 30.3, 29.8, 29.2, 28.8, 25.7, 23.6, 22.7, 18.7, 11.5. **HRMS** (ESI) m/z: $[M+Na]^+$ Calcd for $C_{36}H_{39}BF_4N_2O_2Na$ 641.2942; Found 641.2934.

(*Z*)-4-methyl-7-((7,8,8,9,9,10,10,10-octafluoro-5-(1*H*-naphtho[1,8-*de*][1,3,2]diazaborinin-2(3*H*)-yl)dec-6-en-1-yl)oxy)-2*H*-chromen-2-one (6)

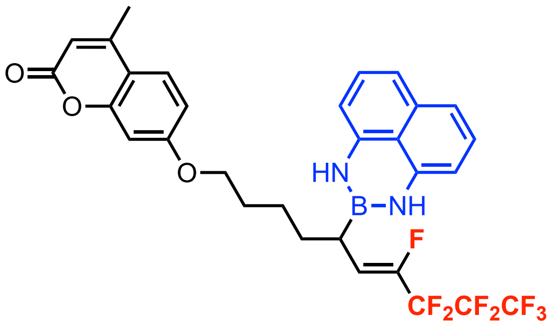

Following the general procedure (IX) at 0.2 mmol scale, using alkene **5** (*E*/*Z* = 90:10) as starting material, **Appearance:** yellow oil; **Yield:** 73.6 mg, 59%**;** R_f = 0.25 (EA: Hexane = 1:5), *Z*/*E* > 99:1 (^{19}F NMR)**.** ^{1}**H NMR** (500 MHz, $CDCl_3$) δ 7.45 (d, *J* = 8.8 Hz, 1H), 7.11 (t, *J* = 7.8 Hz, 2H), 7.04 (d, *J* = 8.2 Hz, 2H), 6.82 (d, *J* = 10.0 Hz, 1H), 6.77 (d, *J* = 2.3 Hz, 1H), 6.34 (d, *J* = 7.2 Hz, 2H), 6.12 (s, 1H), 5.70 (s, 2H), 5.64 (dd, *J* = 22.9, 11.1 Hz, 1H), 4.00 (t, *J* = 6.3 Hz, 2H), 2.50 – 2.40 (m, 1H), 2.36 (s, 3H), 1.94 – 1.82 (m, 2H), 1.80 – 1.70 (m, 1H), 1.67 – 1.58 (m, 2H), 1.55 – 1.46 (m, 1H). ^{19}**F NMR** (471 MHz, $CDCl_3$) δ -80.69 (t, *J* = 9.0 Hz, 3F), -116.95 – -120.03 (m, 2F), -127.24 (d, *J* = 8.4 Hz, 2F), -131.92 – -133.29 (m, 1F). ^{11}**B NMR** (160 MHz, $CDCl_3$) δ 31.90. ^{13}**C NMR** (126 MHz, $CDCl_3$) δ 162.1, 161.5, 155.3, 152.8, 145.3 (dt, *J* = 255.2, 29.3 Hz), 140.5, 136.3, 127.7, 125.6, 119.8, 118.7 – 118.5 (m), 118.2, 113.6, 112.8, 111.9, 110.9 – 107.4 (m, 3C), 106.2, 101.3, 68.1, 30.2, 28.8, 25.6, 24.0, 18.7. **HRMS** (ESI) m/z: $[M+H]^+$ Calcd for $C_{30}H_{25}BF_8N_2O_3$ 625.1909; Found 625.1896.

(*E*)-2-(3,4,4,4-tetrafluorobut-1-en-1-yl)naphthalene (8)

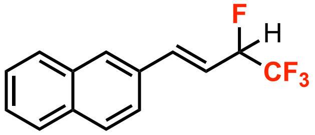

Following the general procedure (XII) at 0.2 mmol scale, using alkene **7** (*E*/*Z* > 99:1) as starting material, **Appearance:** white solid; **Yield:** 44.7 mg, 88%; R_f = 0.30 (Hexane), *E*/*Z* > 99:1 (^{19}F NMR) ^{1}**H NMR** (500 MHz, $CDCl_3$): δ (ppm) 7.92 – 7.77 (m, 4H), 7.68 – 7.46 (m, 3H), 7.07 (dd, *J* = 15.9, 5.0 Hz, 1H), 6.41 – 6.28 (m, 1H), 5.36 – 5.18 (m, 1H). ^{13}**C NMR** (126 MHz, $CDCl_3$): δ (ppm) 139.8 (d, *J* = 11.4 Hz), 133.9, 133.4, 132.2 (d, *J* = 2.3 Hz), 128.8, 128.4, 128.4 (d, *J* = 2.3 Hz), 127.9, 127.0, 126.8, 123.4, 122.5 (qd, *J* = 281.2, 28.3 Hz), 116.8 (dd, *J* = 18.6, 1.8 Hz), 88.7 (dq, *J* = 182.6, 35.2 Hz). ^{19}**F NMR** (471 MHz, $CDCl_3$): δ (ppm) -78.92 (dd, *J* = 14.3, 5.6 Hz, 3F), -190.23 – -190.45 (m, 1F). **HRMS** (ESI) m/z: $[M-H]^-$ Calcd for $C_{14}H_9F_4$ 253.0646; Found 253.0649.

(*Z*)-5,6,6,6-tetrafluoro-1-phenylhex-4-en-3-ol (9a)

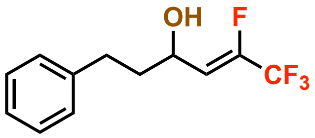

Following the general procedure (X) at 0.2 mmol scale, using alkene **3a** (*E*/*Z* > 99:1) as starting material, **Appearance:** colorless liquid; **Yield:** 37.2mg, 75%**;** R_f = 0.30 (EA: Hexane = 1:8), *Z*/*E* > 99:1 (^{19}F NMR). ^{1}**H NMR** (500 MHz, $CDCl_3$) δ 7.30 (t, *J* = 7.5 Hz, 2H), 7.21 (dd, *J* = 8.9, 7.2 Hz, 3H), 5.65 (dd, *J* = 33.6, 8.5 Hz, 1H), 4.67 (q, *J* = 7.5 Hz, 1H), 2.77 (ddd, *J* = 15.2, 9.5, 6.0 Hz, 1H), 2.70 (ddd, *J* = 13.9, 9.2, 6.6 Hz, 1H), 2.05 – 1.95 (m, 1H), 1.94 – 1.84 (m, 1H), 1.78 (s, 1H). ^{19}**F NMR** (471 MHz, $CDCl_3$) δ -72.76 (d, *J* = 11.2 Hz, 3F), -132.79 (dq, *J* = 33.5, 11.1 Hz, 1F). ^{13}**C NMR** (126 MHz, $CDCl_3$) δ 145.8 (dq, *J* = 260.5, 39.4 Hz), 141.0, 128.7, 128.5, 126.3, 118.3 (qd, *J* = 271.6, 41.7 Hz), 115.7 (td, *J* = 5.8, 2.6 Hz), 64.3 (d, *J* = 3.0 Hz), 38.2, 31.3. **HRMS** (ESI) m/z: $[M-H]^+$ Calcd for $C_{12}H_{11}F_4O$ 247.0752; Found 247.0751.

(*Z*)-1-((4-bromobenzyl)oxy)-5,6,6,6-tetrafluorohex-4-en-3-ol (9b)

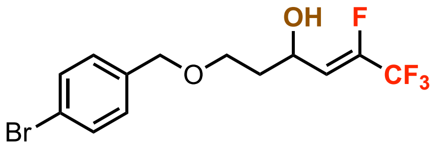

Following the general procedure (X) at 0.2 mmol scale, using alkene **S9b** (*E*/*Z* > 99:1) as starting material, **Appearance:** yellow liquid; **Yield:** 40.7mg, 57%**;** R_f = 0.20 (EA: Hexane = 1:10), *Z*/*E* > 99:1 (^{19}F NMR)**.** ^{1}**H NMR** (500 MHz, $CDCl_3$) δ 7.48 (d, *J* = 8.0 Hz, 2H), 7.19 (d, *J* = 8.0 Hz, 2H), 5.66 (dd, *J* = 34.0, 8.2 Hz, 1H), 4.86 (d, *J* = 9.4 Hz, 1H), 4.47 (s, 2H), 3.76 – 3.59 (m, 2H), 3.22 (s, 1H), 2.00 – 1.91 (m, 1H), 1.90 – 1.79 (m, 1H). ^{19}**F NMR** (471 MHz, $CDCl_3$) δ -72.8 (d, *J* = 11.2 Hz, 3F), -133.0 (dq, *J* = 33.6, 11.2 Hz, 1F). ^{13}**C NMR** (126 MHz, $CDCl_3$) δ 145.3 (dq, *J* = 260.2, 39.4 Hz), 136.7, 131.8, 129.5, 122.0, 118.3 (qd, *J* = 271.5, 41.6 Hz), 115.7 (dq, *J* = 6.3, 3.1 Hz), 72.8, 68.1, 64.4 (d, *J* = 3.2 Hz), 35.9. **HRMS** (ESI) m/z: $[M+Na]^+$ Calcd for $C_{13}H_{13}BrF_4O_2Na$ 378.9927; Found 378.9926.

(Z)-5,6,6,6-tetrafluoro-1-((4'-methoxy-[1,1'-biphenyl]-4-yl)methoxy)hex-4-en-3-ol (9c)

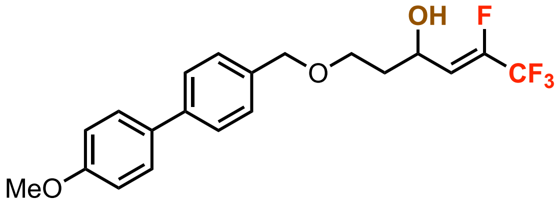

Following the general procedure (X) at 0.2 mmol scale, using alkene **S9c** (*E*/*Z* > 99:1) as starting material, **Appearance:** yellow solid; **Yield:** 38.4mg, 50%**;** R_f = 0.23 (EA: Hexane = 1:5), *Z*/*E* > 99:1 (^{19}F NMR)**.** ^{1}**H NMR** (500 MHz, $CDCl_3$) δ 7.54 (t, *J* = 9.5 Hz, 4H), 7.38 (d, *J* = 7.7 Hz, 2H), 6.99 (d, *J* = 10.5 Hz, 2H), 5.69 (dd, *J* = 34.1, 8.1 Hz, 1H), 4.88 (d, *J* = 9.2 Hz, 1H), 4.57 (d, *J* = 2.3 Hz, 2H), 3.86 (d, *J* = 1.5 Hz, 3H), 3.79 – 3.65 (m, 2H), 3.37 (s, 1H), 2.03 – 1.83 (m, 2H). ^{19}**F NMR** (471 MHz, $CDCl_3$) δ -72.72 (d, *J* = 11.0 Hz, 3F), -133.05 (dq, *J* = 33.8, 11.2 Hz, 1F). ^{13}**C NMR** (126 MHz, $CDCl_3$) δ 159.4, 145.3 (dq, *J* = 260.4, 39.3 Hz), 140.7, 135.9, 133.4, 128.4, 128.2, 127.0, 118.3 (qd, *J* = 271.6, 41.7 Hz), 115.9 (dd, *J* = 6.3, 3.2 Hz), 114.4, 73.4, 68.2, 64.8 (d, *J* = 3.2 Hz), 55.5, 35.9. **HRMS** (ESI) m/z: $[M+Na]^+$ Calcd for $C_{20}H_{20}F_4O_3Na$ 407.1241; Found 407.1238.

(Z)-1,1,1,2-tetrafluoro-8-(4-methoxyphenoxy)oct-2-en-4-ol (9d)

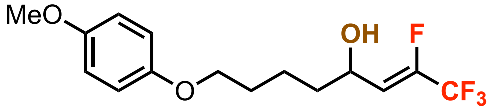

Following the general procedure (X) at 0.2 mmol scale, using alkene **S9d** (*E*/*Z* > 99:1) as starting material, **Appearance:** white solid; **Yield:** 39.9 mg, 62%**;** R_f = 0.20 (EA: Hexane = 1:5), *Z*/*E* > 99:1 (^{19}F NMR)**.** ^{1}**H NMR** (500 MHz, $CDCl_3$) δ 6.83 (s, 4H), 5.62 (dd, *J* = 33.7, 8.6 Hz, 1H), 4.67 (dt, *J* = 10.9, 6.4 Hz, 1H), 3.92 (t, *J* = 6.3 Hz, 2H), 3.77 (d, *J* = 1.1 Hz, 3H), 2.34 – 2.21 (m, 1H), 1.87 – 1.68 (m, 3H), 1.66 – 1.48 (m, 3H). ^{19}**F NMR** (471 MHz, $CDCl_3$) δ -72.73 (d, *J* = 11.2 Hz, 3F), -133.21 (dq, *J* = 33.7, 11.1 Hz, 1F). ^{13}**C NMR** (126 MHz, $CDCl_3$) δ 153.9, 153.2, 145.7 (dq, *J* = 260.1, 39.4 Hz), 118.3 (dq, *J* = 271.7, 41.9 Hz), 115.8 (dd, *J* = 6.7, 3.2 Hz), 115.6, 114.8, 68.4, 64.8 (d, *J* = 3.0 Hz), 55.8, 36.4, 29.1, 21.7.**HRMS** (ESI) m/z: $[M+Na]^+$ Calcd for $C_{15}H_{18}F_4O_3Na$ 345.1084; Found 345.1082.

(Z)-5,6,6,7,7,7-hexafluoro-1-phenylhept-4-en-3-ol (11)

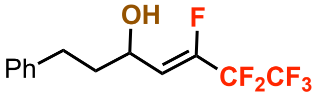

Following the general procedure (X) at 0.2 mmol scale, using alkene **10** (*E*/*Z* > 99:1) as starting material, **Appearance:** colorless liquid; **Yield:** 25.0 mg, 42%**;** R_f = 0.40 (EA: Hexane = 1:5), *Z*/*E* > 99:1 (^{19}F NMR)**.** ^{1}**H NMR** (500 MHz, $CDCl_3$) δ 7.31 (t, *J* = 7.4 Hz, 2H), 7.24 – 7.15 (m, 3H), 5.71 (dd, *J* = 34.0, 8.4 Hz, 1H), 4.70 (q, *J* = 7.3 Hz, 1H), 2.83 – 2.63 (m, 2H), 2.08 – 1.94 (m, 2H), 1.95 – 1.82 (m, 1H). ^{19}**F NMR** (471 MHz, $CDCl_3$) δ -83.18 – -84.82 (m, 3F), -121.49 (dd, *J* = 25.6, 14.6 Hz, 2F), -129.45 (dtd, *J* = 35.2, 14.0, 13.6, 7.0 Hz, 1F). ^{13}**C NMR** (126 MHz, $CDCl_3$) δ 145.8 (dt, *J* = 261.4, 29.2 Hz), 141.0, 128.7, 128.5, 126.3, 118.4 (dt, *J* = 7.0, 3.6 Hz), 119.8 – 116.5 (m), 110.8 – 104.7 (m), 64.6 (d, *J* = 3.1 Hz), 38.3, 31.3. **HRMS** (ESI) m/z: $[M+Cl]^+$ Calcd for $C_{13}H_{12}F_6OCl$ 333.0486; Found 333.0490.

(*Z*)-2-(5,6,6,6-tetrafluoro-1-phenylhex-4-en-3-yl)-2,3-dihydro-1*H*-naphtho[1,8-*de*][1,3,2]diazaborinine (chiral-4a)

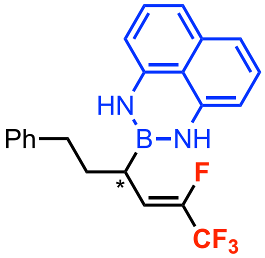

Following the general procedure (XI) at 0.2 mmol scale, using alkene **S9a** (*E*/*Z* > 99:1) as starting material, **Appearance**: colorless oil; **Yield**: 59.7 mg, 75%; R_f = 0.25 (EA: Hexane = 1:50); *Z*/*E* > 99:1 (^{19}F NMR), **e.r.**: 93: 7; $[\alpha]_D{}^{25}$ = -33 (c = 0.08, $CHCl_3$); **HPLC condition**: Daicel Chiralpak IB column, isopropanol : hexane = 10:90, flow rate = 1.0 mL/min, UV = 254 nm, t_1 = 20.9 min (major) and t_2 = 27.3 min (minor); ^{1}**H NMR** (500 MHz, $CDCl_3$) δ 7.42 (t, *J* = 7.5 Hz, 2H), 7.33 (t, *J* = 7.5 Hz, 1H), 7.31 – 7.25 (d, *J* = 7.2 Hz, 2H), 7.21 (t, *J* = 7.8 Hz, 2H), 7.15 (d, *J* = 8.3 Hz, 2H), 6.39 (d, *J* = 7.3 Hz, 2H), 5.66 (dd, *J* = 22.9 Hz, 10.9 Hz, 1H), 5.61 (s, 2H), 2.94 – 2.75 (m, 1H), 2.75 – 2.60 (m, 1H), 2.42 (td, *J* = 10.5, 5.2 Hz, 1H), 2.13 – 1.97 (m, 1H), 1.97 – 1.82 (m, 1H). ^{19}**F NMR** (471 MHz, $CDCl_3$) δ -71.61 (d, *J* = 11.6 Hz, 3F), -135.89 (dq, *J* = 35.0, 12.1 Hz, 1F). ^{11}**B NMR** (128 MHz, $CDCl_3$) δ 30.33. ^{13}**C NMR** (126 MHz, $CDCl_3$) δ 145.7 (dq, *J* = 253.4, 38.7 Hz), 141.2, 140.4, 136.3, 128.6, 128.5, 127.7, 126.3, 119.8, 118.7 (qd, *J* = 270.9, 42.2 Hz), 118.3, 115.4 (dq, *J* = 9.8, 3.2 Hz), 106.2, 35.3, 32.4. **HRMS** (ESI) m/z: $[M]^+$ Calcd for $C_{22}H_{19}BF_4N_2$ 398.1576; Found 398.1574.

(*Z*)-4-methyl-*N*-phenyl-*N*-(8,9,9,9-tetrafluoro-6-(1*H*-naphtho[1,8-*de*][1,3,2]diazaborinin-2(3*H*)-yl)non-7-en-1-yl)benzenesulfonamide (4f)

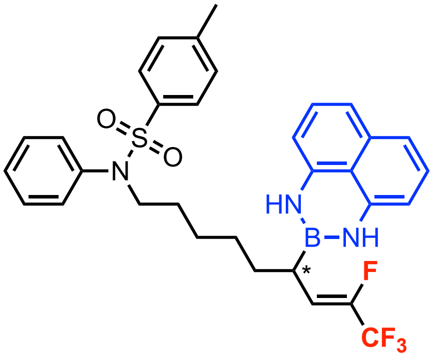

Following the general procedure (XI) at 0.2 mmol scale, using alkene **3f** (*E*/*Z* > 99:1) as starting material, **Appearance**: yellow oil; **Yield**: 80.4 mg, 66%; R_f = 0.30 (EA: Hexane = 1:10); *Z*/*E* > 99:1 (^{19}F NMR), **e.r.**: 85: 15; $[\alpha]_D{}^{25}$ = -45 (c = 0.12, $CHCl_3$); **HPLC condition**: Daicel Chiralpak IB column, isopropanol : hexane = 10:90, flow rate = 1.0 mL/min, UV = 254 nm, t_1 = 41.2 min (major) and t_2 = 51.5 min (minor); ^{1}**H NMR** (500 MHz, $CDCl_3$) δ 7.47 (d, *J* = 8.2 Hz,

2H), 7.34 – 7.29 (m, 3H), 7.23 (d, J = 8.0 Hz, 2H), 7.11 (t, J = 7.8 Hz, 2H), 7.09 – 7.02 (m, 4H), 6.36 (d, J = 7.2 Hz, 2H), 5.67 (s, 2H), 5.54 (dd, J = 33.8, 10.9 Hz, 1H), 3.60 – 3.48 (m, 2H), 2.42 (s, 3H), 2.28 (td, J = 9.7, 5.8 Hz, 1H), 1.71 – 1.34 (m, 8H). **^{19}F NMR** (471 MHz, $CDCl_3$) δ -71.65 (d, J = 11.9 Hz, 3F), -136.80 (dq, J = 34.9, 12.0 Hz, 1F). **^{11}B NMR** (128 MHz, $CDCl_3$) δ 31.54. **^{13}C NMR** (126 MHz, $CDCl_3$) δ 145.36 (dq, J = 252.5, 38.9 Hz), 143.5, 140.6, 139.1, 136.3, 135.3, 129.5, 129.1, 128.9, 128.0, 127.7, 127.6, 119.8, 118.7 (qd, J = 270.9, 42.3 Hz), 118.1, 116.1 – 115.2 (m), 106.2, 50.2, 30.4, 28.5, 27.9, 25.9, 23.5, 21.6. **HRMS** (ESI) m/z: $[M+Na]^+$ Calcd for $C_{32}H_{32}BF_4N_3O_2SNa$ 632.2142; Found 632.2133.

(*Z*)-2-(5,6,6,6-tetrafluoro-1-((4'-methoxy-[1,1'-biphenyl]-4-yl)methoxy)hex-4-en-3-yl)-2,3-dihydro-1*H*-naphtho[1,8-*de*][1,3,2]diazaborinine (4g)

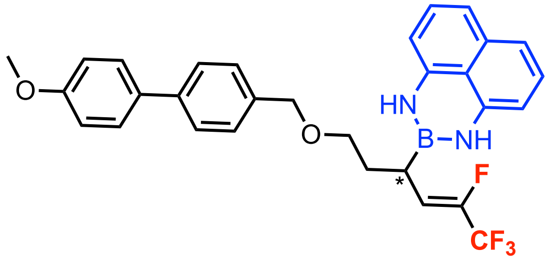

Following the general procedure (XI) at 0.2 mmol scale, using alkene **S9c** (*E*/*Z* > 99:1) as starting material, **Appearance**: yellow oil; **Yield**: 48.0 mg, 45%; R_f = 0.30 (EA: Hexane = 1:15); *Z*/*E* > 99:1 (^{19}F NMR), **e.r.**: 13: 87; **$[\alpha]_D^{25}$** = -30 (c = 0.09, $CHCl_3$); **HPLC condition**: Daicel Chiralpak IA column, isopropanol : hexane = 10:90, flow rate = 1.0 mL/min, UV = 254 nm, t_1 = 15.4 min (major) and t_2 = 12.3 min (minor); **^{1}H NMR** (500 MHz, $CDCl_3$) δ 7.56 (d, J = 3.2 Hz, 2H), 7.54 (d, J = 2.6 Hz, 2H), 7.42 (d, J = 8.0 Hz, 2H), 7.13 – 7.07 (m, 2H), 7.07 – 7.04 (m, 2H), 7.04 – 7.00 (m, 2H), 6.24 (dd, J = 7.1, 1.1 Hz, 2H), 5.77 (s, 2H), 5.65 (dd, J = 33.7, 11.0 Hz, 1H), 4.57 (s, 2H), 3.88 (s, 3H), 3.68 – 3.60 (m, 1H), 3.56 (s, 1H), 2.68 – 2.50 (m, 1H), 2.06 – 1.97 (m, 1H), 1.88 – 1.73 (m, 1H). **^{19}F NMR** (471 MHz, $CDCl_3$) δ -71.6 (d, J = 12.0 Hz), -136.2 (dq, J = 34.6, 11.8 Hz). **^{11}B NMR** (160 MHz, $CDCl_3$) δ 31.15. **^{13}C NMR** (126 MHz, $CDCl_3$) δ 159.3, 145.5 (dq, J = 254.0, 38.6 Hz), 140.6, 140.5, 136.4, 136.3, 133.5, 128.5, 128.2, 127.7, 126.9, 119.8, 118.7 (dd, J = 270.9, 42.0 Hz), 118.1, 115.3 (dd, J = 10.0, 3.6 Hz), 114.3, 106.2, 73.3, 69.2, 55.5, 30.8, 20.8. **HRMS** (ESI) m/z: $[M+Na]^+$ Calcd for $C_{30}H_{27}BF_4N_2O_2Na$ 557.1999; Found 557.1990

9. Reference

1. Ichitsuka, T.; Fujita, T.; Arita, T.; Ichikawa, J., Double C-F bond activation through beta-fluorine elimination: nickel-mediated [3+2] cycloaddition of 2-trifluoromethyl-1-alkenes with alkynes. *Angew Chem Int Ed Engl* **2014,** *53* (29), 7564-8.
2. Tang, Y.; Tsui, G. C., Copper-catalyzed pentafluoroethylation of aryl/alkenyl iodides with pentafluoroethylsilane. *Org Chem Front* **2024,** *11* (16), 4366-4370.
3. Yang, X.; Tsui, G. C., Trifluoromethylation of Unactivated Alkenes with Me_3SiCF_3 and N-Iodosuccinimide. *Org Lett* **2019,** *21* (5), 1521-1525.
4. Tang, L.; Lv, G.; Fu, Y.; Chang, X. P.; Cheng, R.; Wang, L.; Zhou, Q., Bifunctional 1,8-Diazabicyclo[5.4.0]undec-7-ene for Visible Light-Induced Heck-Type Perfluoroalkylation of Alkenes. *J Org Chem* **2022,** *87* (21), 14763-14777.
5. Frisch, M. J.; Trucks, G. W.; Schlegel, H. B.; Scuseria, G. E.; Robb, M. A.; Cheeseman, J. R.; Scalmani, G.; Barone, V.; Petersson, G. A.; Nakatsuji, H.; Li, X.; Caricato, M.; Marenich, A. V.; Bloino, J.; Janesko, B. G.; Gomperts, R.; Mennucci, B.; Hratchian, H. P.; Ortiz, J. V.; Izmaylov, A. F.; Sonnenberg, J. L.; Williams; Ding, F.; Lipparini, F.; Egidi, F.; Goings, J.; Peng, B.; Petrone, A.; Henderson, T.; Ranasinghe, D.; Zakrzewski, V. G.; Gao, J.; Rega, N.; Zheng, G.; Liang, W.; Hada, M.; Ehara, M.; Toyota, K.; Fukuda, R.; Hasegawa, J.; Ishida, M.; Nakajima, T.; Honda, Y.; Kitao, O.; Nakai, H.; Vreven, T.; Throssell, K.; Montgomery Jr., J. A.; Peralta, J. E.; Ogliaro, F.; Bearpark, M. J.; Heyd, J. J.; Brothers, E. N.; Kudin, K. N.; Staroverov, V. N.; Keith, T. A.; Kobayashi, R.; Normand, J.; Raghavachari, K.; Rendell, A. P.; Burant, J. C.; Iyengar, S. S.; Tomasi, J.; Cossi, M.; Millam, J. M.; Klene, M.; Adamo, C.; Cammi, R.; Ochterski, J. W.; Martin, R. L.; Morokuma, K.; Farkas, O.; Foresman, J. B.; Fox, D. J. *Gaussian 16 Rev. C.01*, Wallingford, CT, 2016.
6. Becke, A. D., Density-functional thermochemistry. III. The role of exact exchange. *J Chem Phys* **1993,** *98* (7), 5648-5652.
7. Lee, C.; Yang, W.; Parr, R. G., Development of the Colle-Salvetti correlation-energy formula into a functional of the electron density. *Phys Rev B Condens Matter* **1988,** *37* (2), 785-789.
8. Grimme, S.; Antony, J.; Ehrlich, S.; Krieg, H., A consistent and accurate ab initio parametrization of density functional dispersion correction (DFT-D) for the 94 elements H-Pu. *J Chem Phys* **2010,** *132* (15), 154104.
9. Grimme, S.; Ehrlich, S.; Goerigk, L., Effect of the damping function in dispersion corrected density functional theory. *J Comput Chem* **2011,** *32* (7), 1456-65.
10. Weigend, F.; Ahlrichs, R., Balanced basis sets of split valence, triple zeta valence and quadruple zeta valence quality for H to Rn: Design and assessment of accuracy. *Phys Chem Chem Phys* **2005,** *7* (18), 3297-305.
11. Goerigk, L.; Grimme, S., Efficient and Accurate Double-Hybrid-Meta-GGA Density Functionals-Evaluation with the Extended GMTKN30 Database for General Main Group Thermochemistry, Kinetics, and Noncovalent Interactions. *J Chem Theory Comput* **2011,** *7* (2), 291-309.
12. Caldeweyher, E.; Bannwarth, C.; Grimme, S., Extension of the D3 dispersion coefficient model. *J Chem Phys* **2017,** *147* (3), 034112.
13. Marenich, A. V.; Cramer, C. J.; Truhlar, D. G., Universal solvation model based on solute electron density and on a continuum model of the solvent defined by the bulk dielectric constant and atomic surface tensions. *J Phys Chem B* **2009,** *113* (18), 6378-96.
14. Weigend, F., Accurate Coulomb-fitting basis sets for H to Rn. *Phys Chem Chem Phys* **2006,**

8 (9), 1057-65.
15. Hellweg, A.; Hättig, C.; Höfener, S.; Klopper, W., Optimized accurate auxiliary basis sets for RI-MP2 and RI-CC2 calculations for the atoms Rb to Rn. *Theor Chem Acc* **2007,** *117* (4), 587-597.
16. Lu, T.; Chen, F., Multiwfn: a multifunctional wavefunction analyzer. *J Comput Chem* **2012,** *33* (5), 580-92.
17. Legault, C. Y. CYLview20. http://www.cylview.org.

10. Spectrum

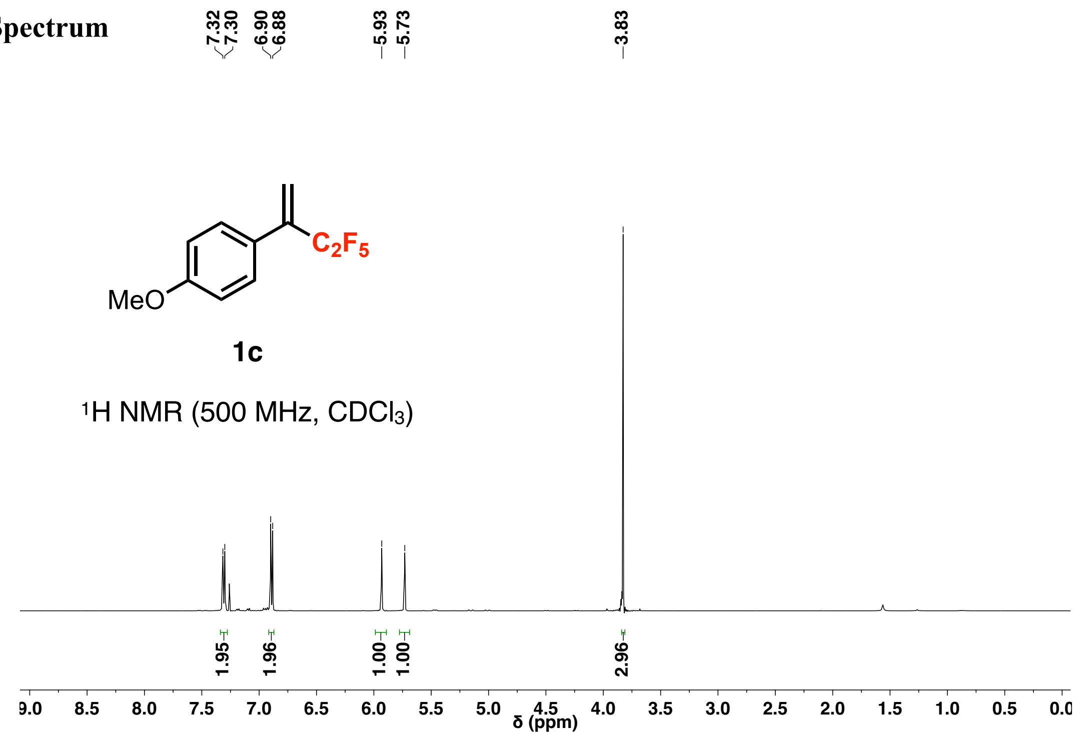

7.32
7.30
6.90
6.88
5.93
5.73
3.83
C_2F_5
MeO
1c
^1H NMR (500 MHz, $CDCl_3$)
1.95
1.96
1.00
1.00
2.96
δ (ppm)

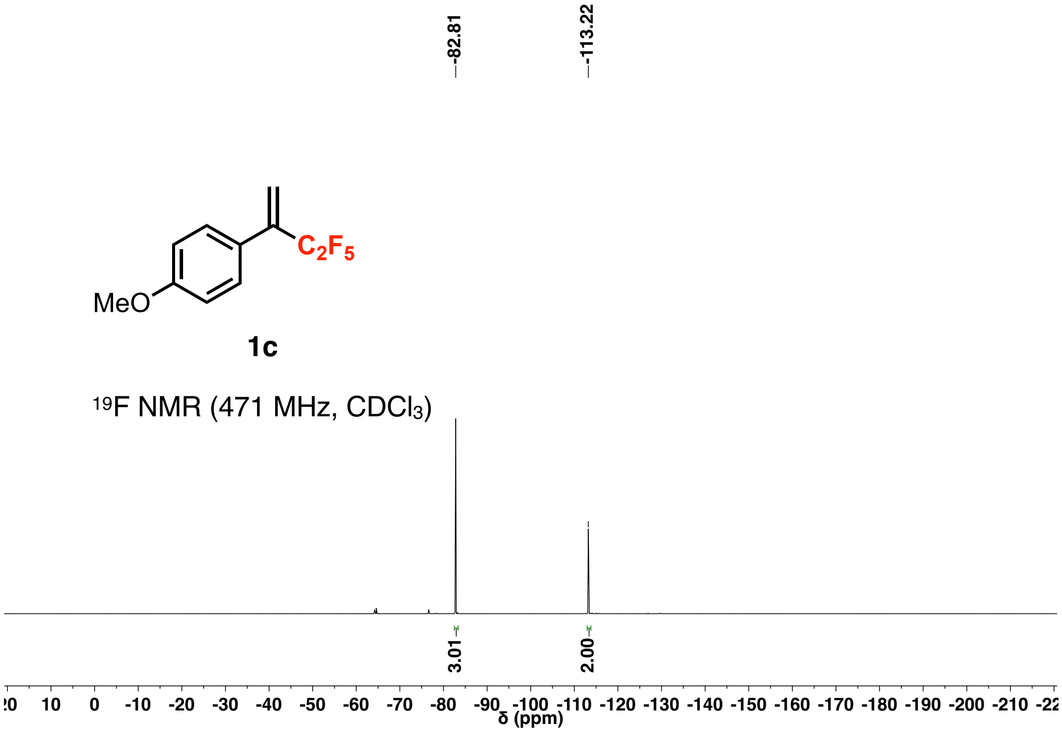

-82.81
-113.22
C_2F_5
MeO
1c
^{19}F NMR (471 MHz, $CDCl_3$)
3.01
2.00
δ (ppm)

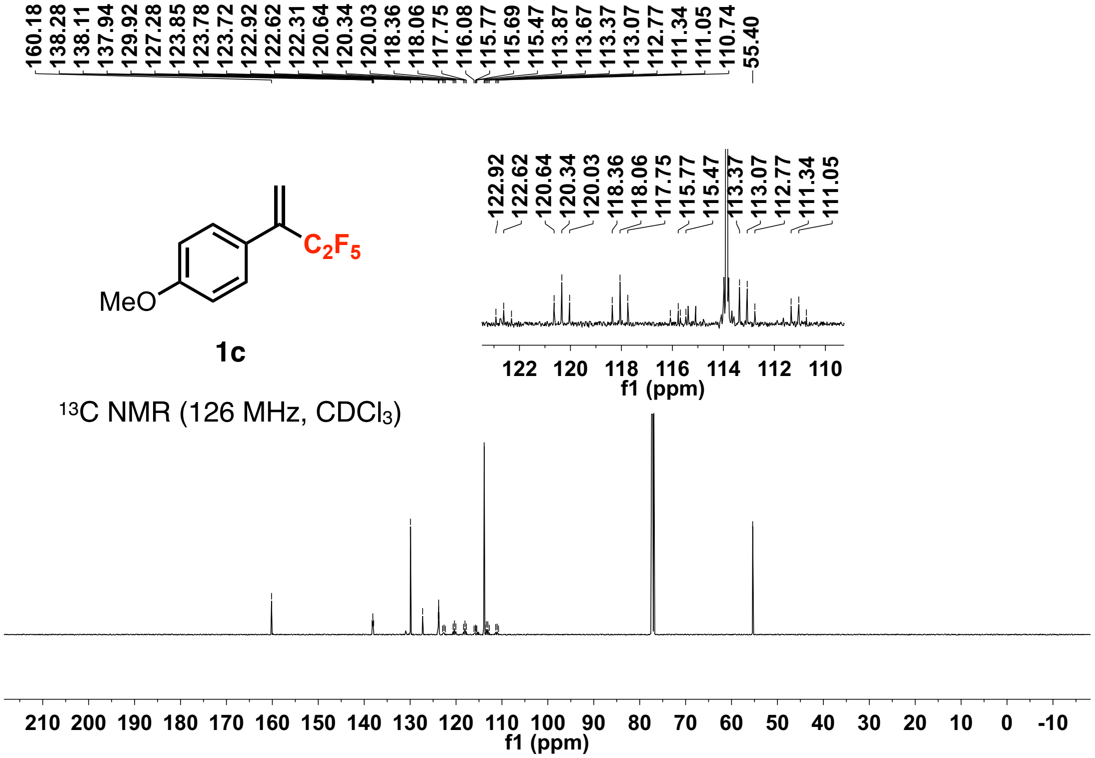
160.18
138.28
138.11
137.94
129.92
127.28
123.85
123.78
123.72
122.92
122.62
122.31
120.64
120.34
120.03
118.36
118.06
117.75
116.08
115.77
115.69
115.47
113.87
113.67
113.37
113.07
112.77
111.34
111.05
110.74
55.40
MeO
C_2F_5
1c
^{13}C NMR (126 MHz, $CDCl_3$)
122.92
122.62
120.64
120.34
120.03
118.36
118.06
117.75
115.77
115.47
113.37
113.07
112.77
111.34
111.05
122 120 118 116 114 112 110
f1 (ppm)
210 200 190 180 170 160 150 140 130 120 110 100 90 80 70 60 50 40 30 20 10 0 -10
f1 (ppm)

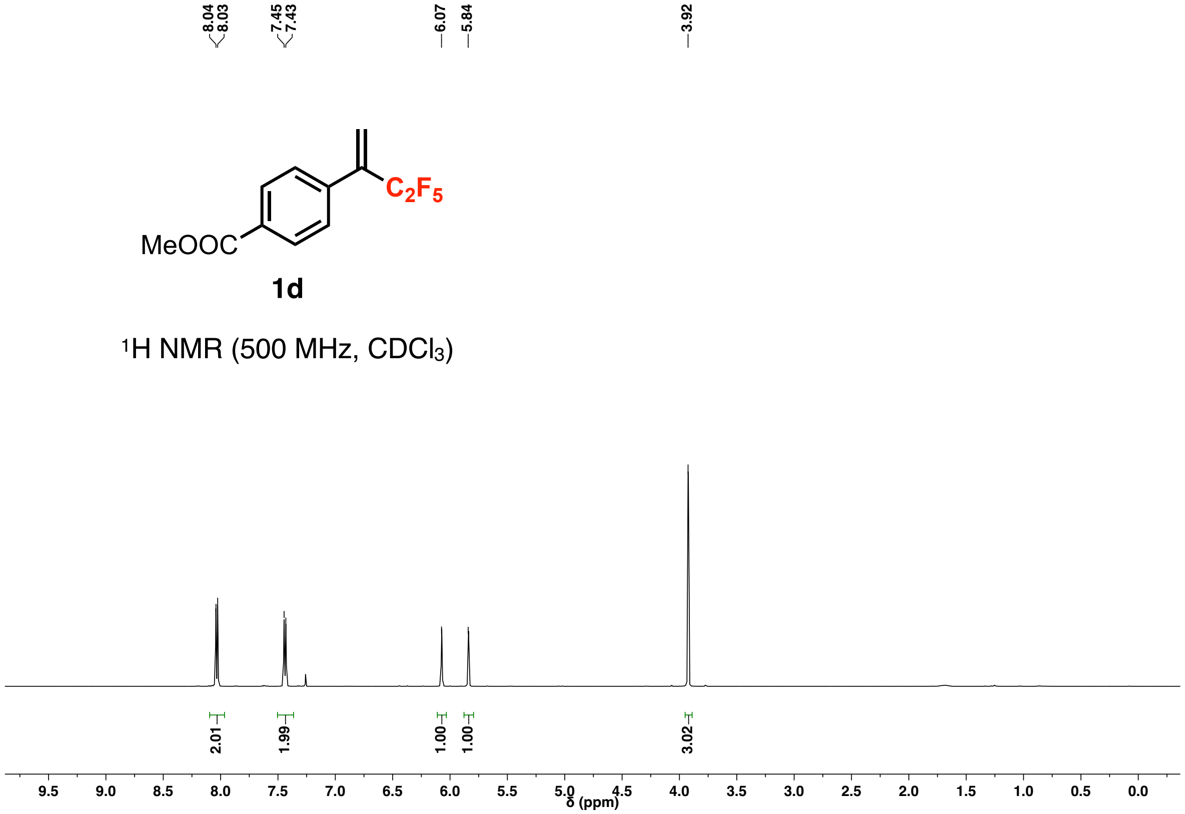
8.04
8.03
7.45
7.43
6.07
5.84
3.92
MeOOC
C_2F_5
1d
^{1}H NMR (500 MHz, $CDCl_3$)
2.01
1.99
1.00
1.00
3.02
9.5 9.0 8.5 8.0 7.5 7.0 6.5 6.0 5.5 5.0 4.5 4.0 3.5 3.0 2.5 2.0 1.5 1.0 0.5 0.0
δ (ppm)

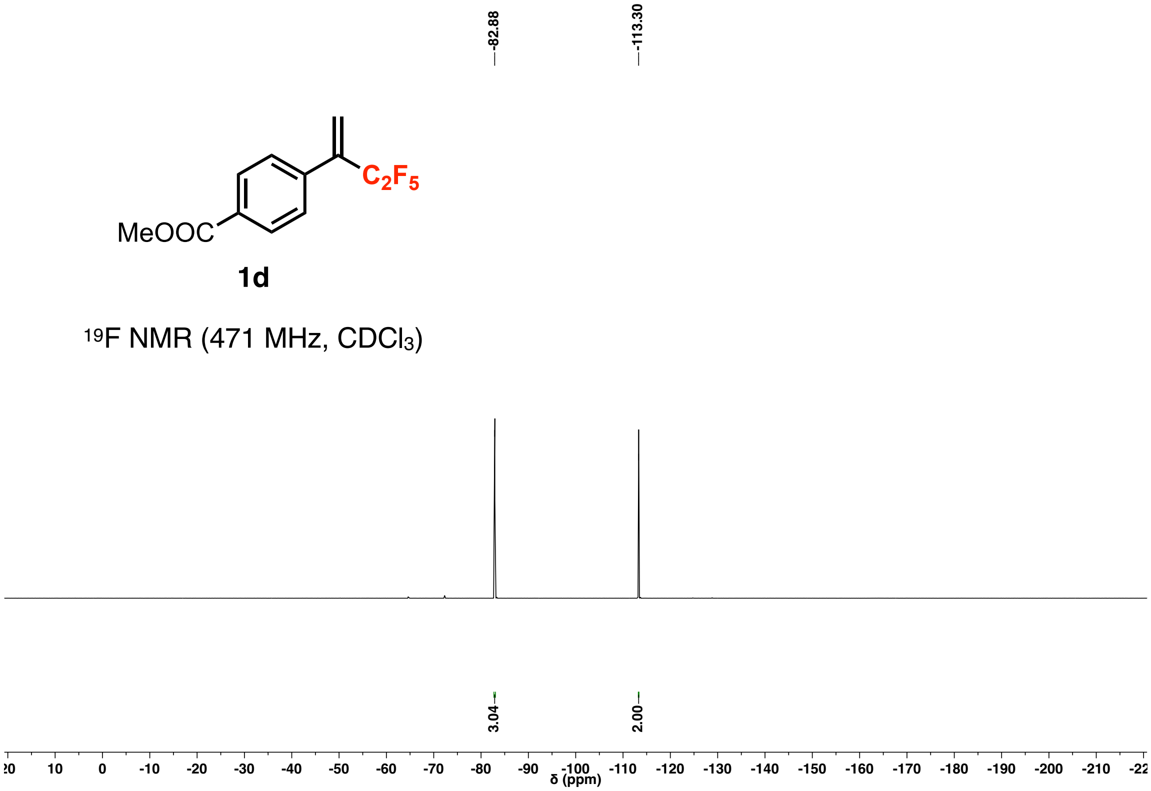

^{19}F NMR (471 MHz, $CDCl_3$)

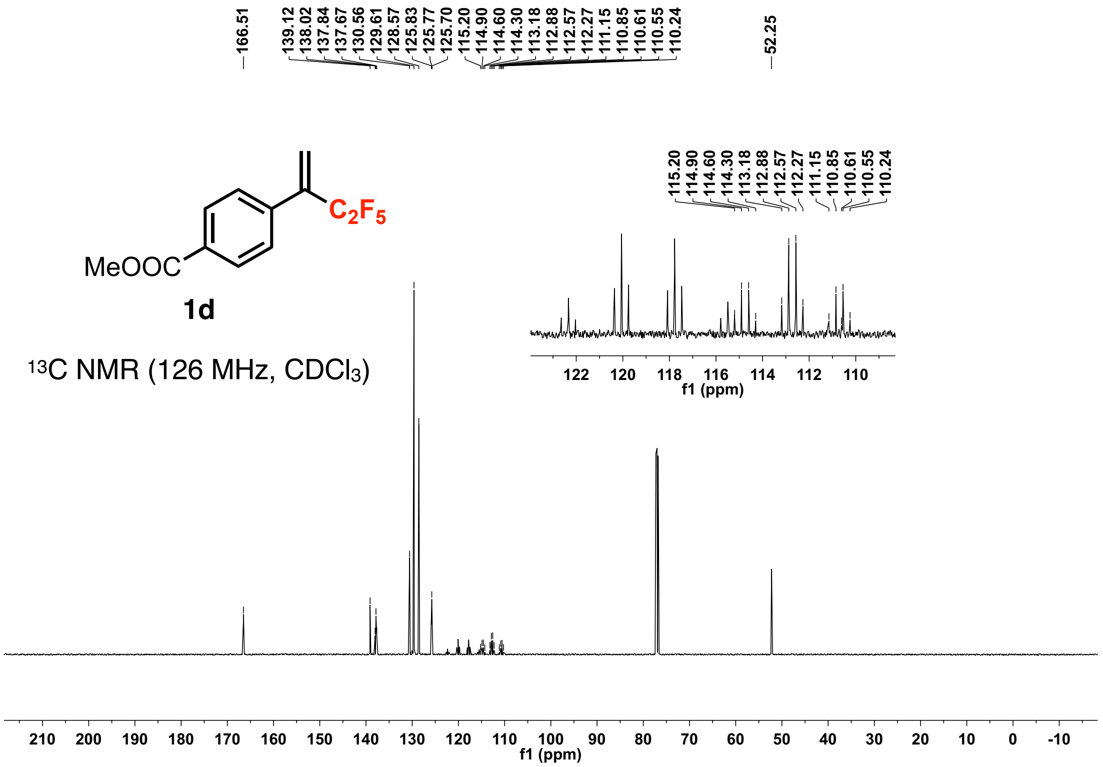

^{13}C NMR (126 MHz, $CDCl_3$)

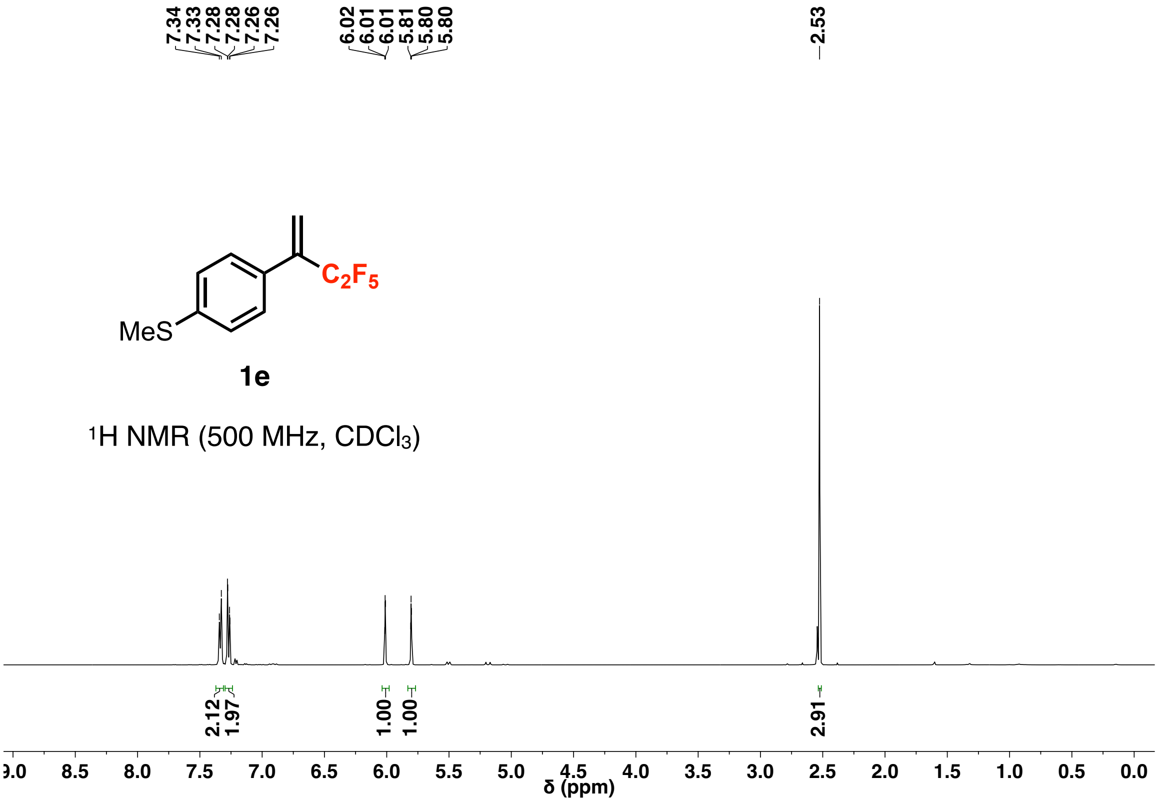

1e

^{1}H NMR (500 MHz, $CDCl_3$)

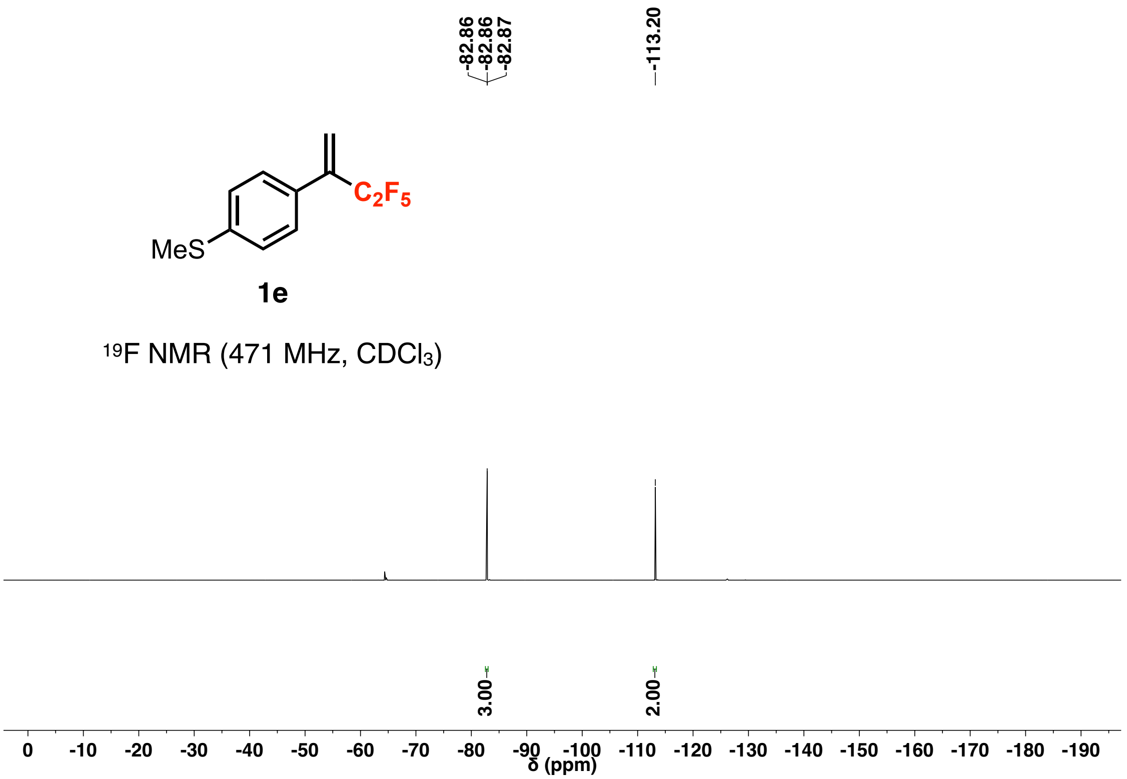

1e

^{19}F NMR (471 MHz, $CDCl_3$)

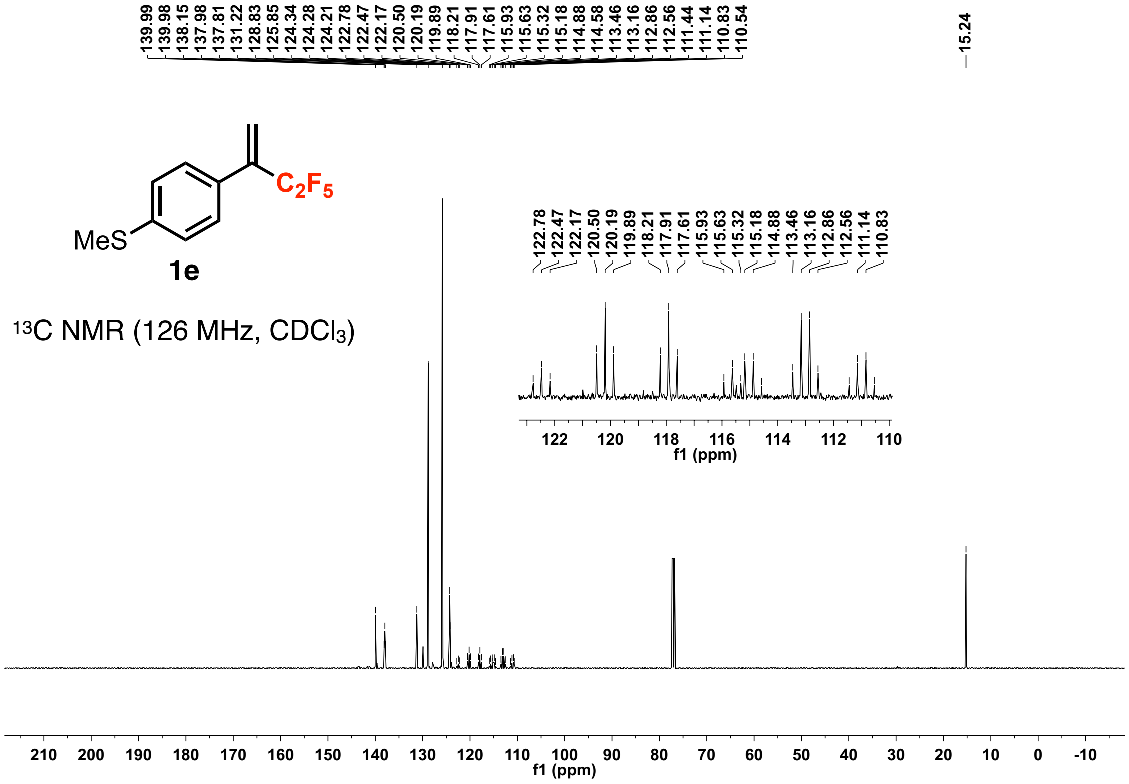
139.99
139.98
138.15
137.98
137.81
131.22
128.83
125.85
124.34
124.28
124.21
122.78
122.47
122.17
120.50
120.19
119.89
118.21
117.91
117.61
115.93
115.63
115.32
115.18
114.88
114.58
113.46
113.16
112.86
112.56
111.44
111.14
110.83
110.54
15.24
MeS
C2F5
1e
13C NMR (126 MHz, CDCl3)
f1 (ppm)

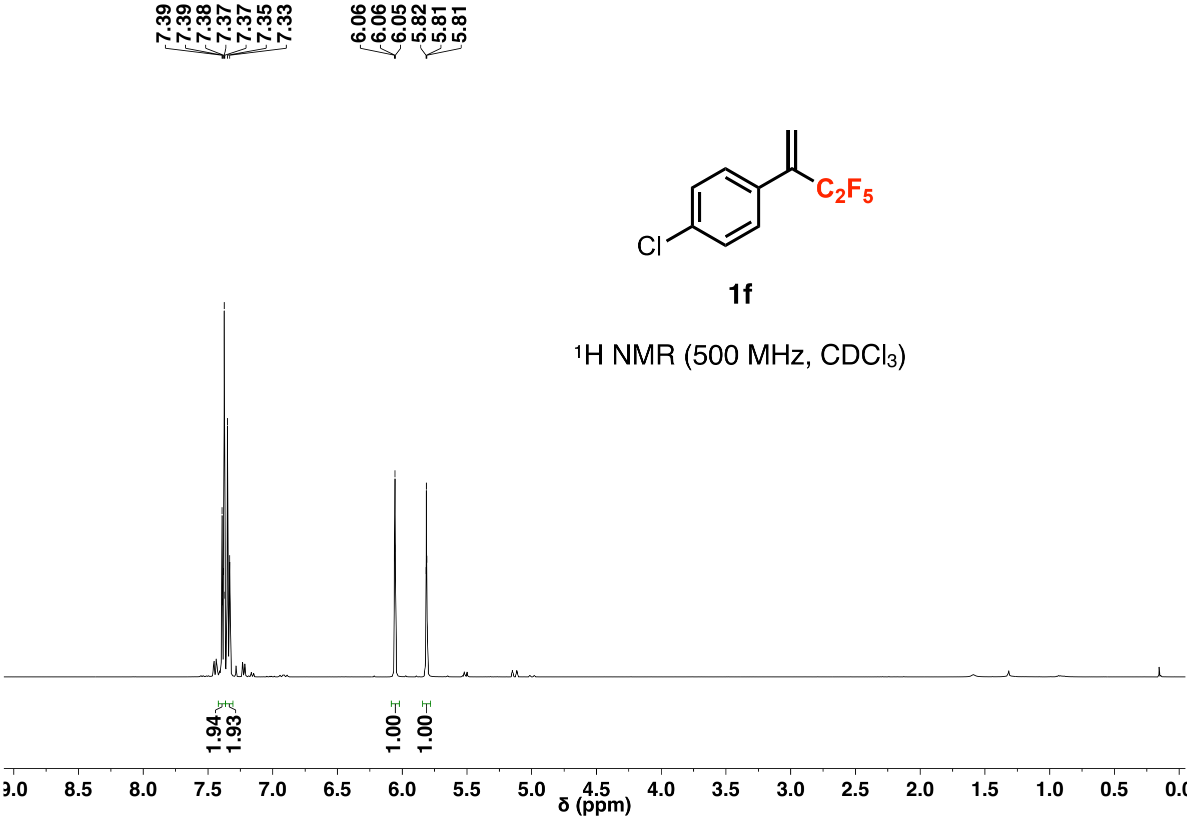
7.39
7.39
7.38
7.37
7.37
7.35
7.33
6.06
6.06
6.05
5.82
5.81
5.81
Cl
C2F5
1f
1H NMR (500 MHz, CDCl3)
1.94
1.93
1.00
1.00
δ (ppm)

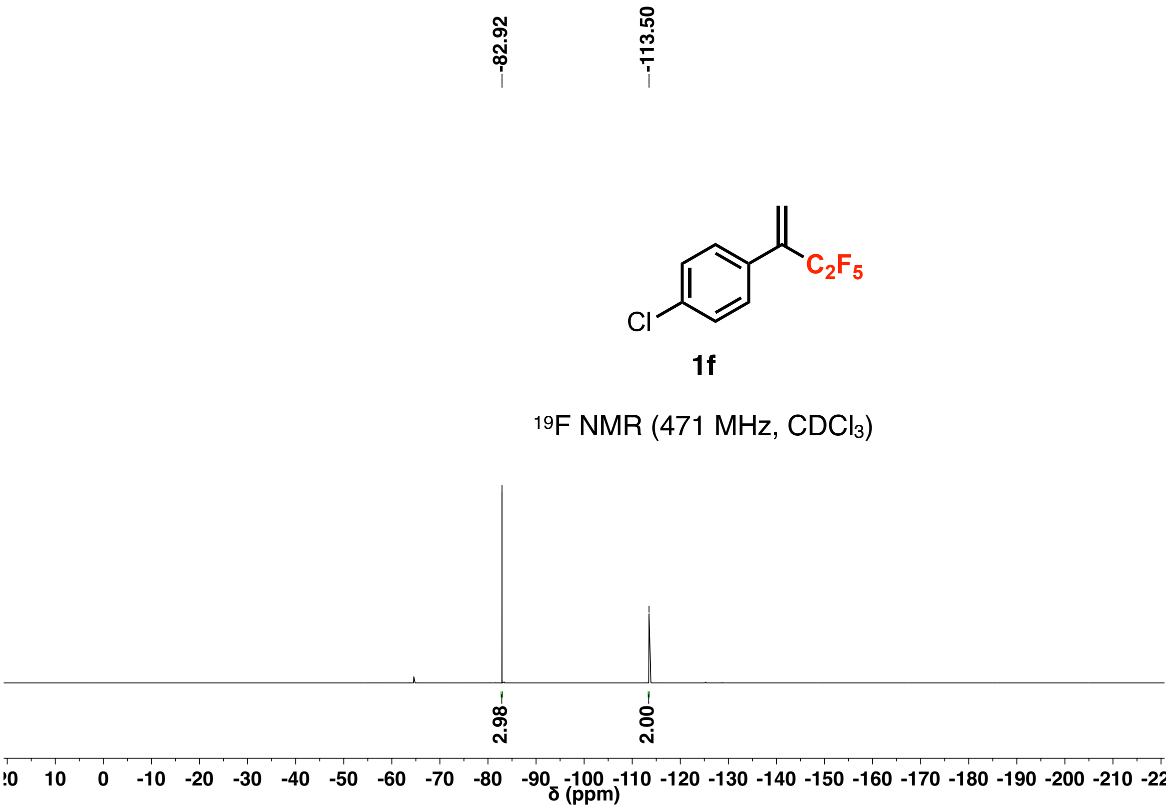

-82.92
-113.50
C2F5
Cl
1f
19F NMR (471 MHz, CDCl3)
2.98
2.00
20 10 0 -10 -20 -30 -40 -50 -60 -70 -80 -90 -100 -110 -120 -130 -140 -150 -160 -170 -180 -190 -200 -210 -22
δ (ppm)

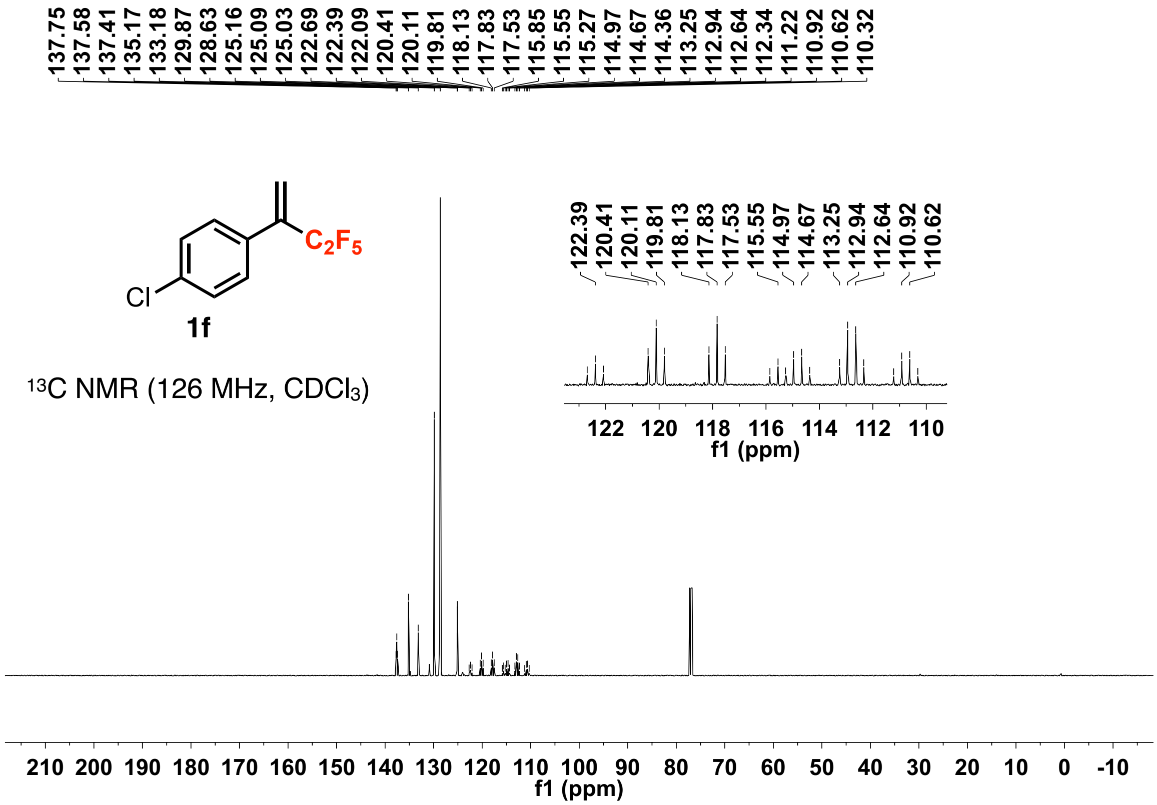

137.75
137.58
137.41
135.17
133.18
129.87
128.63
125.16
125.09
125.03
122.69
122.39
122.09
120.41
120.11
119.81
118.13
117.83
117.53
115.85
115.55
115.27
114.97
114.67
114.36
113.25
112.94
112.64
112.34
111.22
110.92
110.62
110.32
C2F5
Cl
1f
13C NMR (126 MHz, CDCl3)
122.39
120.41
120.11
119.81
118.13
117.83
117.53
115.55
114.97
114.67
113.25
112.94
112.64
110.92
110.62
122 120 118 116 114 112 110
f1 (ppm)
210 200 190 180 170 160 150 140 130 120 110 100 90 80 70 60 50 40 30 20 10 0 -10
f1 (ppm)

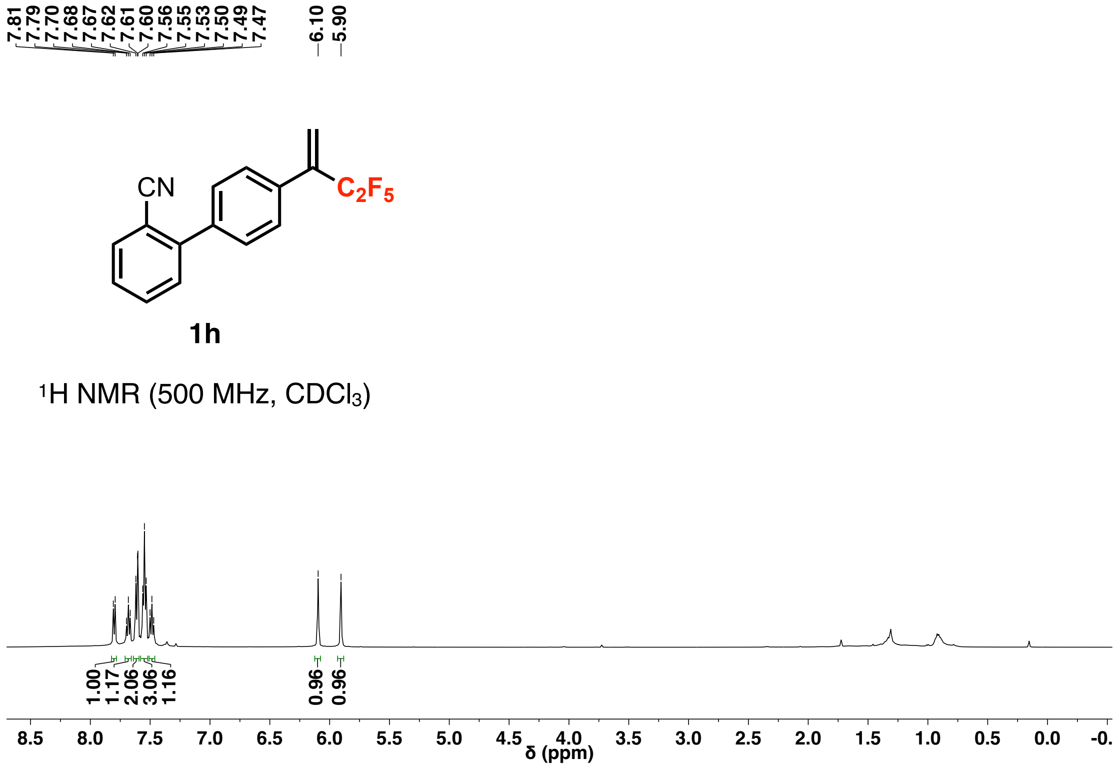
7.81
7.79
7.70
7.68
7.67
7.62
7.61
7.60
7.56
7.55
7.53
7.50
7.49
7.47
6.10
5.90
CN
C2F5
1h
1H NMR (500 MHz, CDCl3)
1.00
1.17
2.06
3.06
1.16
0.96
0.96
8.5 8.0 7.5 7.0 6.5 6.0 5.5 5.0 4.5 4.0 3.5 3.0 2.5 2.0 1.5 1.0 0.5 0.0
δ (ppm)

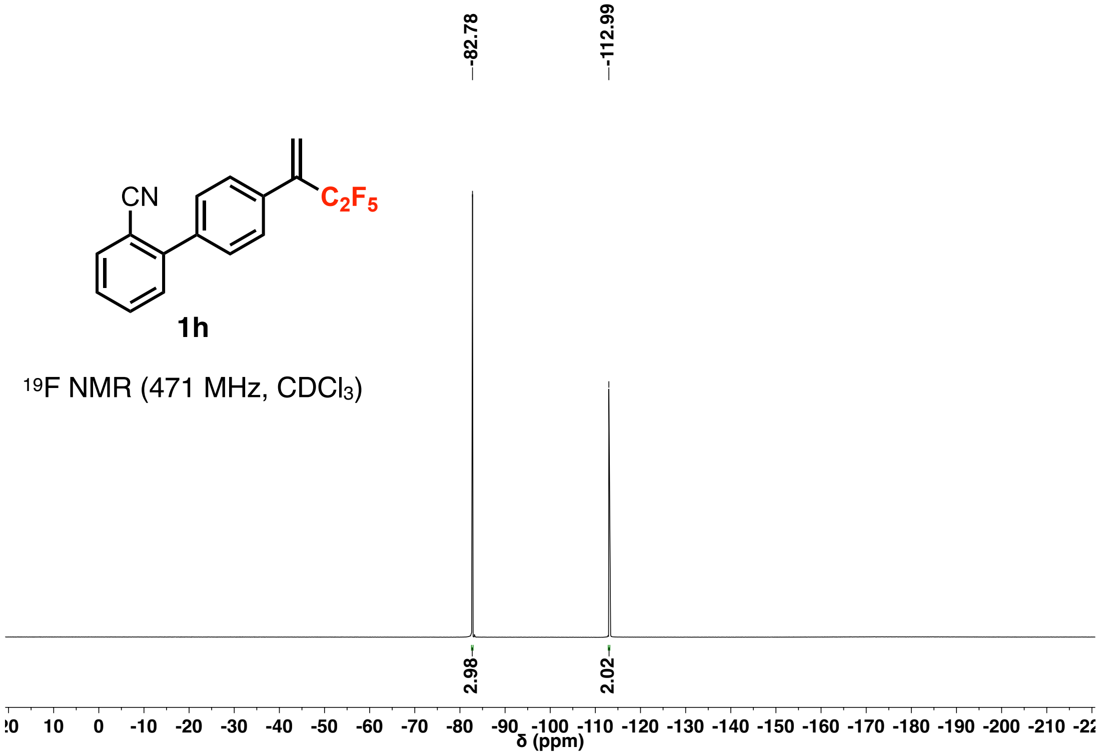
-82.78
-112.99
CN
C2F5
1h
19F NMR (471 MHz, CDCl3)
2.98
2.02
10 0 -10 -20 -30 -40 -50 -60 -70 -80 -90 -100 -110 -120 -130 -140 -150 -160 -170 -180 -190 -200 -210
δ (ppm)

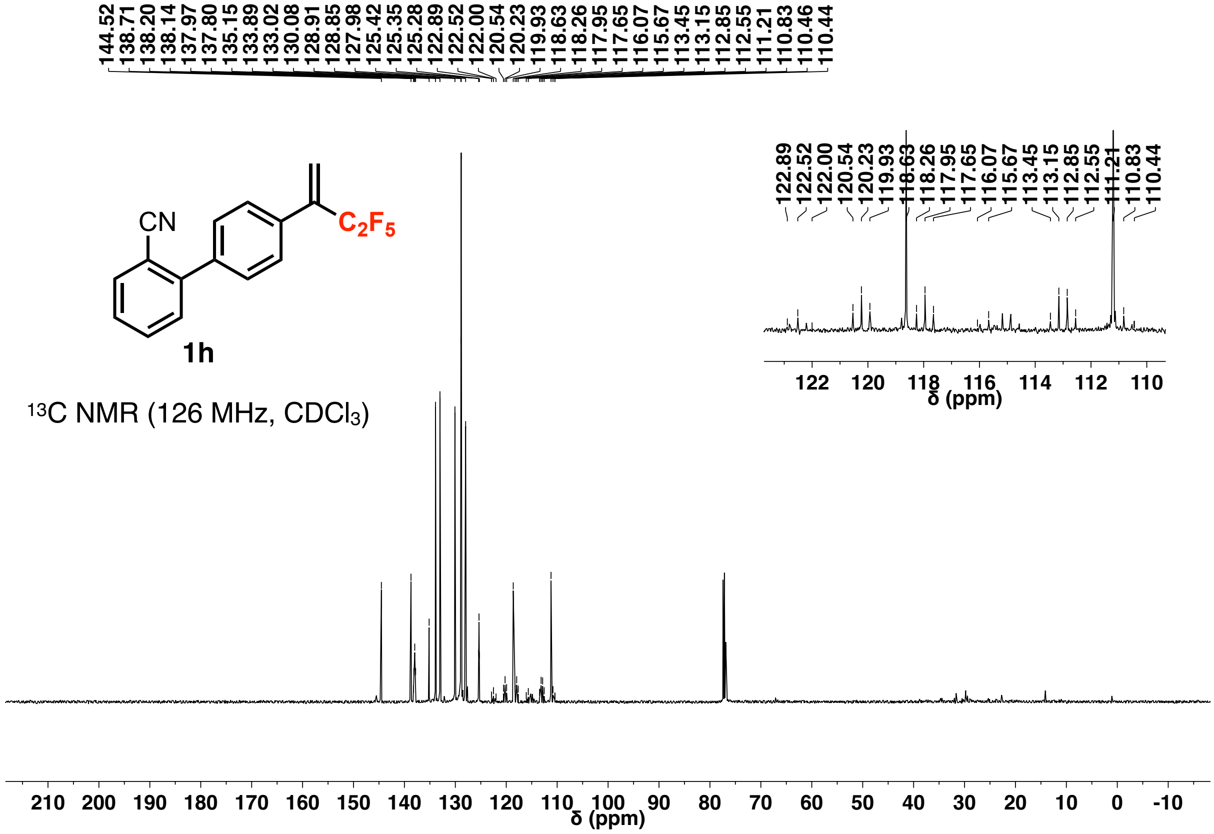
144.52 138.71 138.20 138.14 137.97 137.80 135.15 133.89 133.02 130.08 128.91 128.85 127.98 125.42 125.35 125.28 122.89 122.52 122.00 120.54 120.23 119.93 118.63 118.26 117.95 117.65 116.07 115.67 113.45 113.15 112.85 112.55 111.21 110.83 110.46 110.44
122.89 122.52 122.00 120.54 120.23 119.93 118.63 118.26 117.95 117.65 116.07 115.67 113.45 113.15 112.85 112.55 111.21 110.83 110.44
CN
C2F5
1h
13C NMR (126 MHz, CDCl3)
δ (ppm)

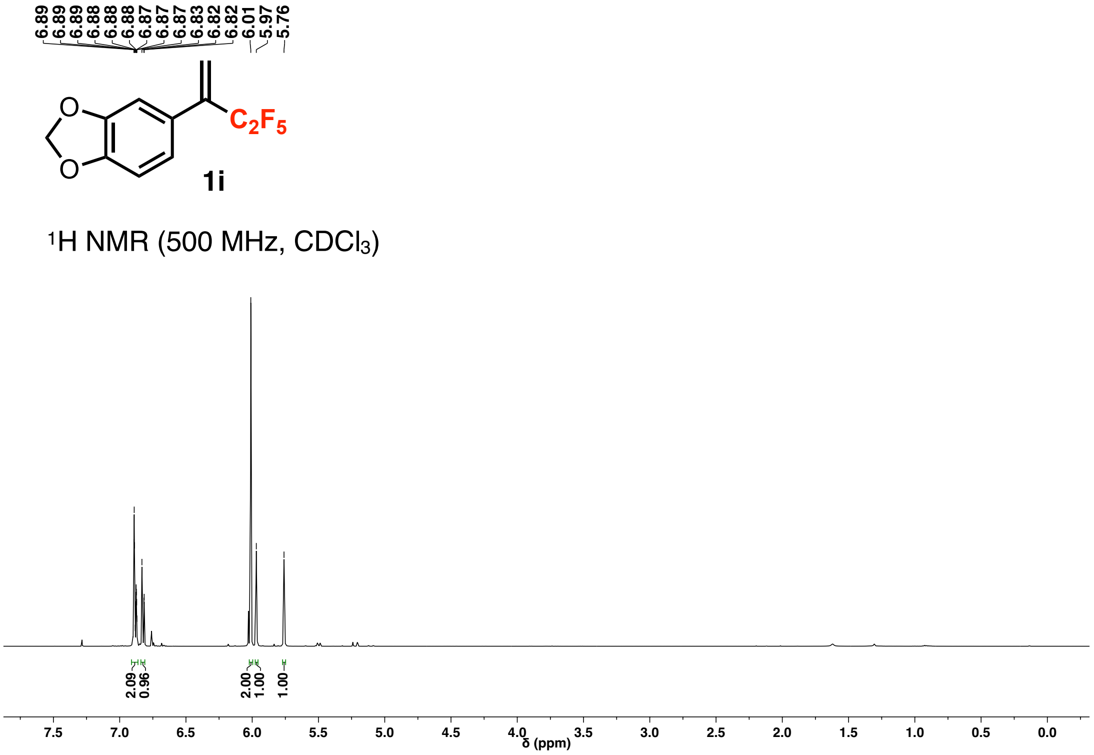
6.89 6.89 6.88 6.88 6.88 6.87 6.87 6.83 6.82 6.82 6.01 5.97 5.76
O
O
C2F5
1i
1H NMR (500 MHz, CDCl3)
2.09 0.96 2.00 1.00 1.00
δ (ppm)

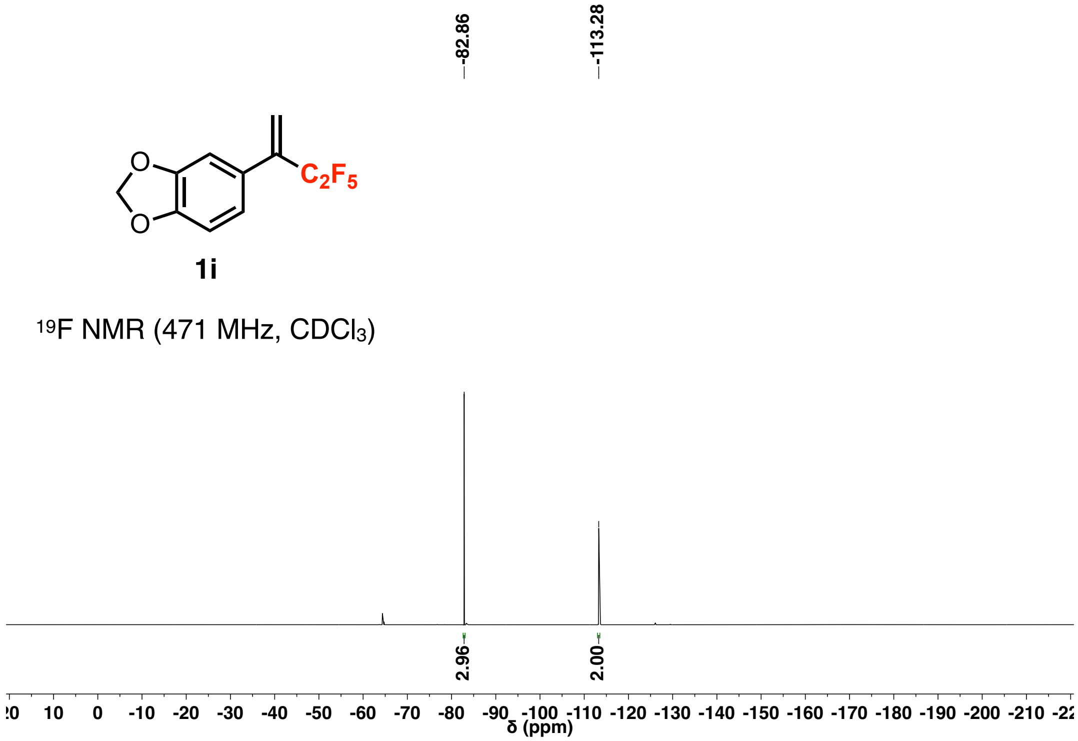

-82.86
-113.28
C2F5
1i
19F NMR (471 MHz, CDCl3)
2.96
2.00
δ (ppm)

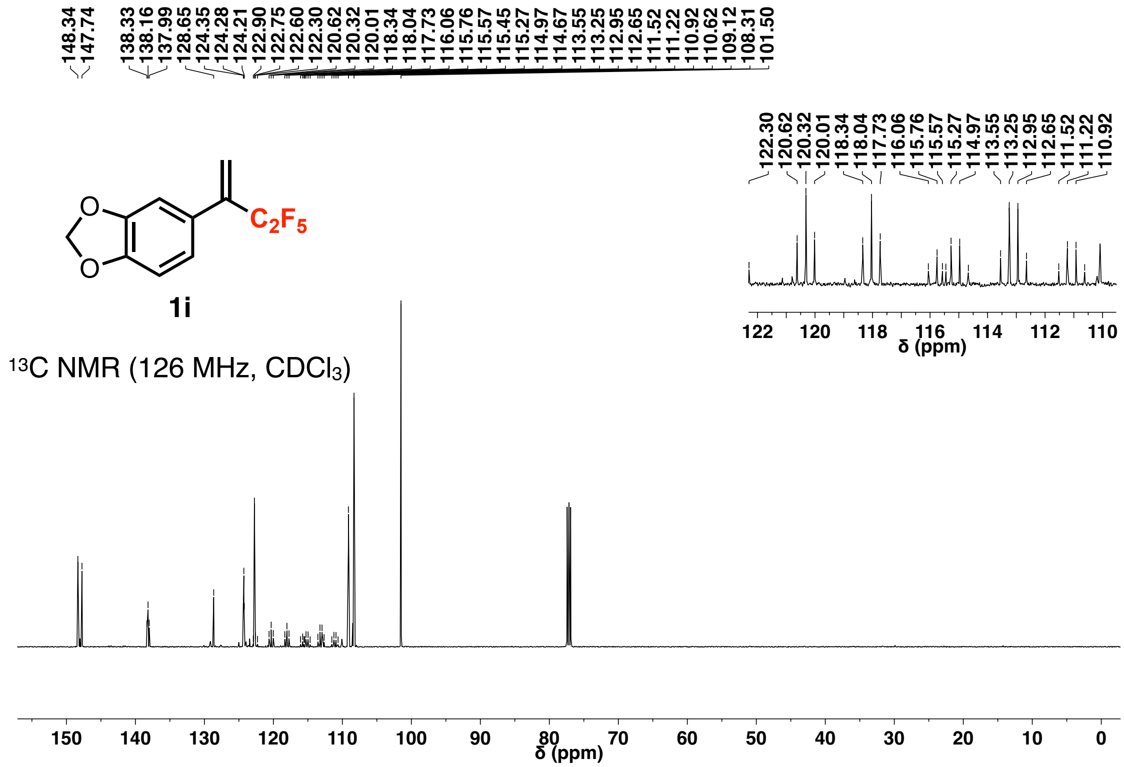

148.34
147.74
138.33
138.16
137.99
128.65
124.35
124.28
124.21
122.90
122.75
122.60
122.30
120.62
120.32
120.01
118.34
118.04
117.73
116.06
115.76
115.57
115.45
115.27
114.97
114.67
113.55
113.25
112.95
112.65
111.52
111.22
110.92
110.62
109.12
108.31
101.50
C2F5
1i
13C NMR (126 MHz, CDCl3)
δ (ppm)

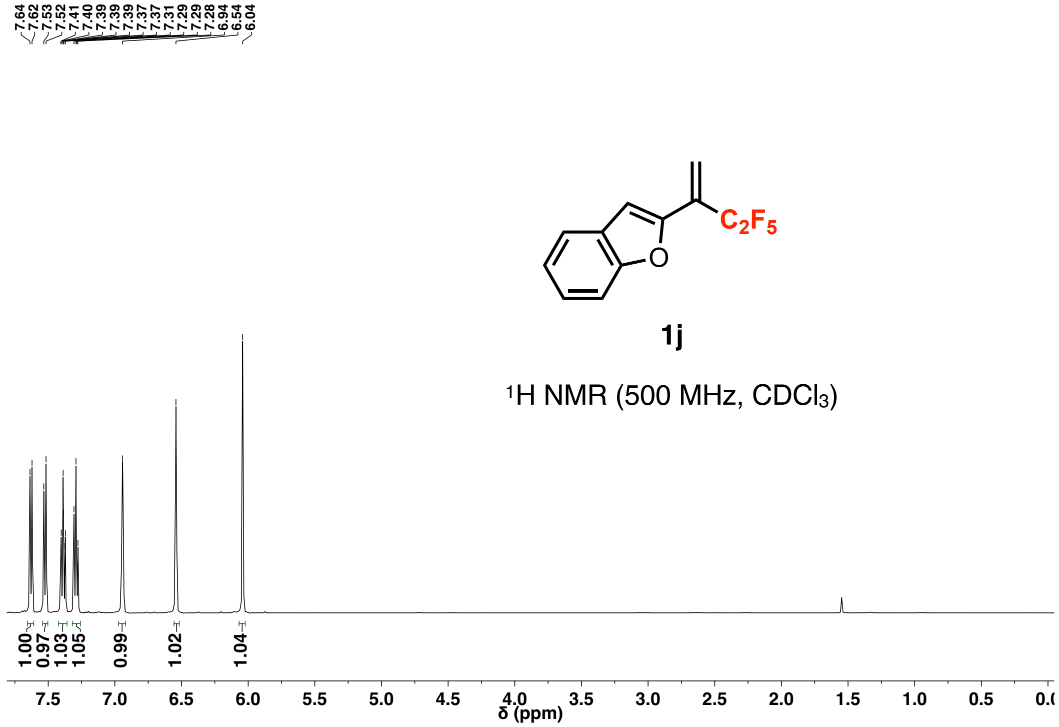

1j
^{1}H NMR (500 MHz, CDCl$_3$)
δ (ppm)

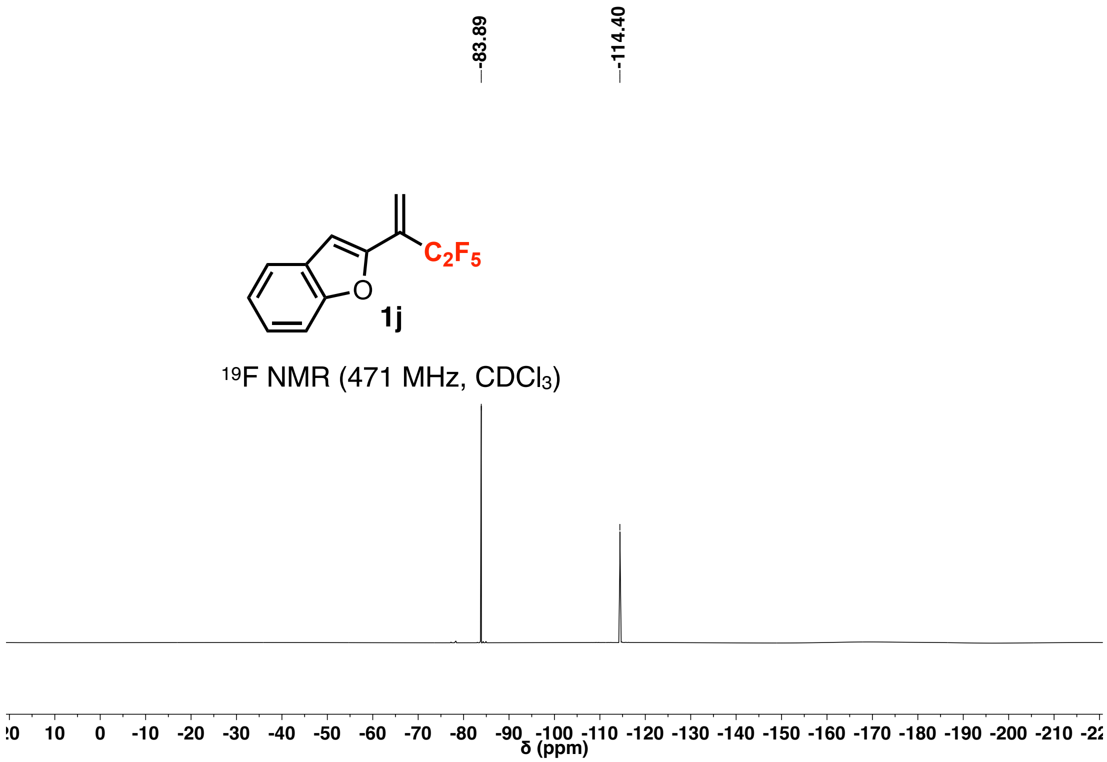

1j
^{19}F NMR (471 MHz, CDCl$_3$)
-83.89
-114.40
δ (ppm)

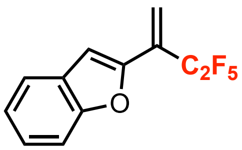

1j

^{13}C NMR (126 MHz, $CDCl_3$)

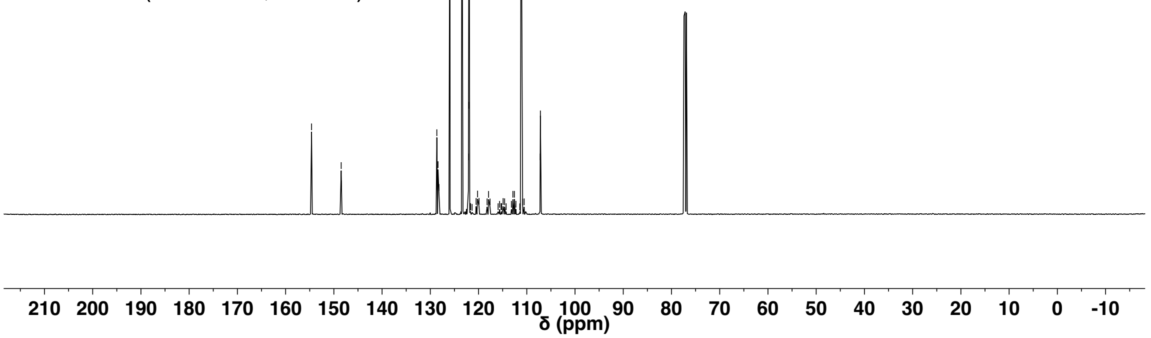

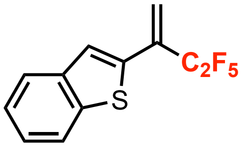

1k

^{1}H NMR (500 MHz, $CDCl_3$)

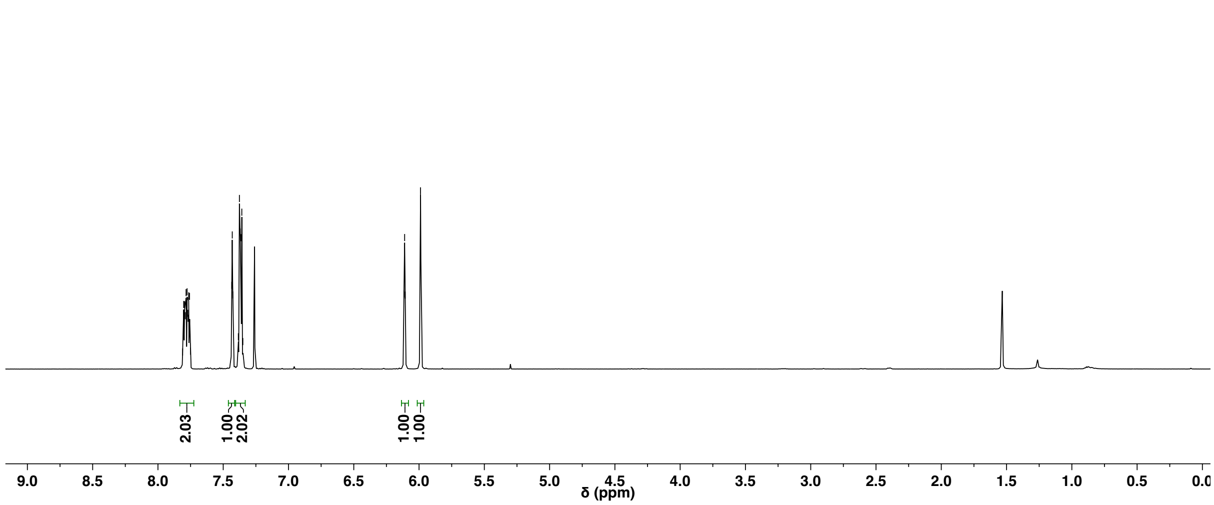

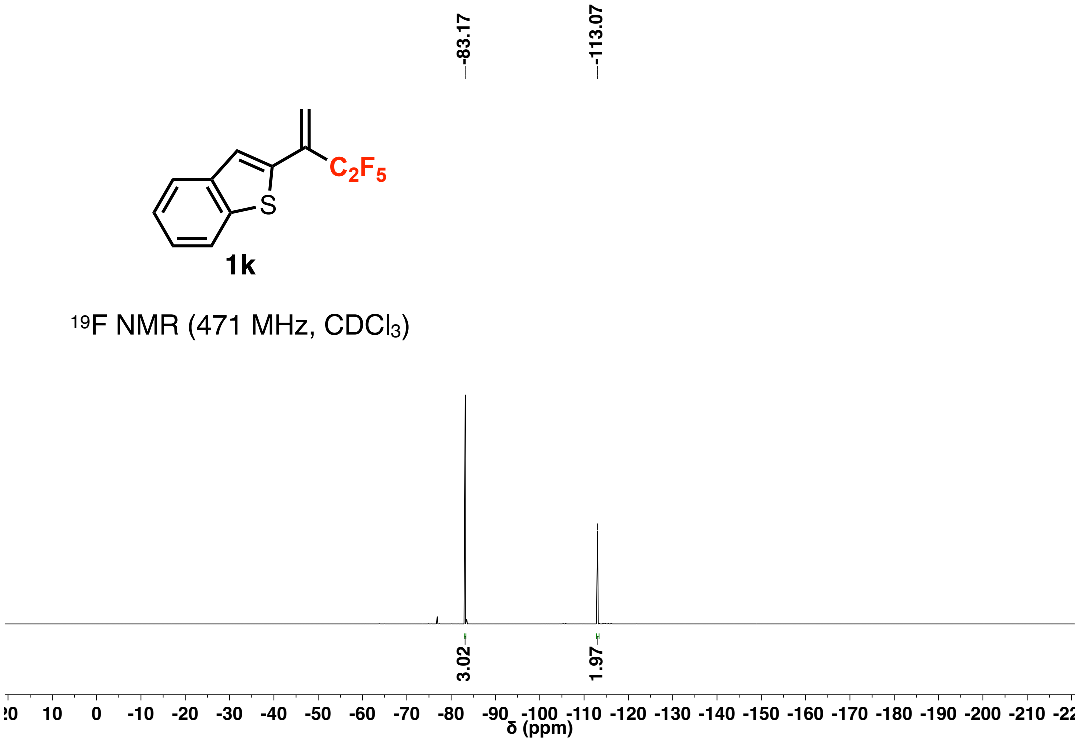

1k

^{19}F NMR (471 MHz, $CDCl_3$)

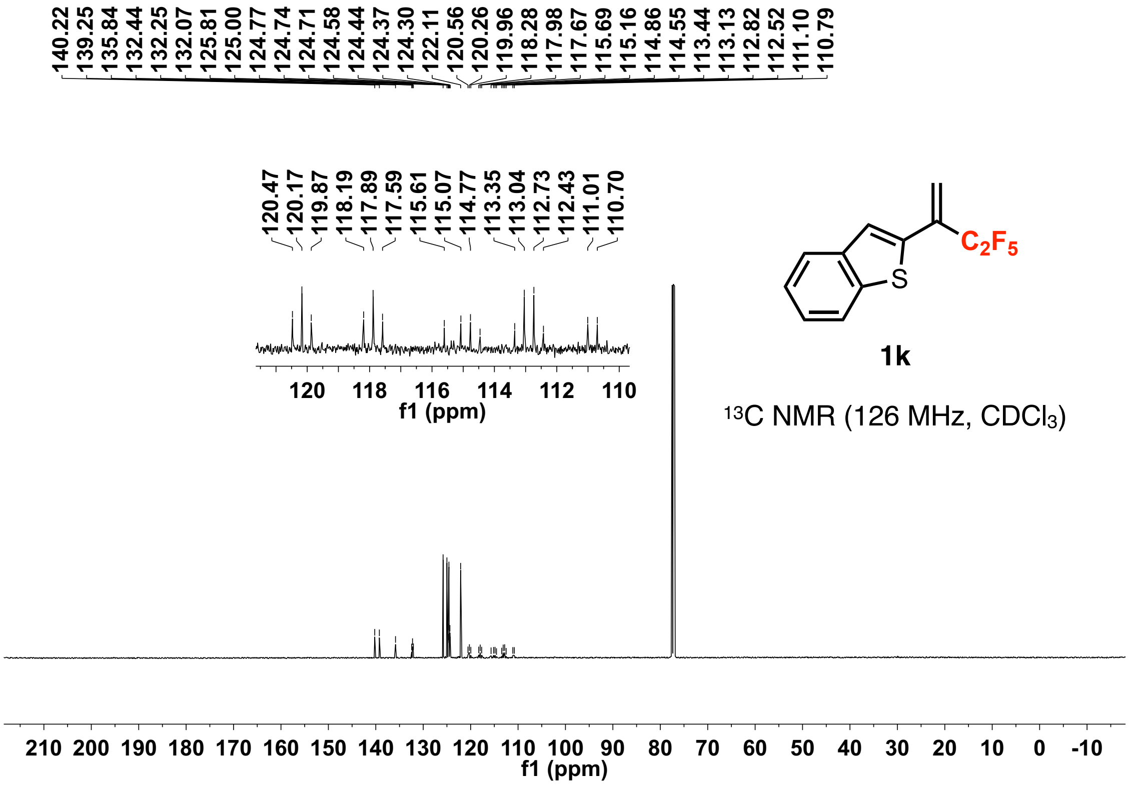

1k

^{13}C NMR (126 MHz, $CDCl_3$)

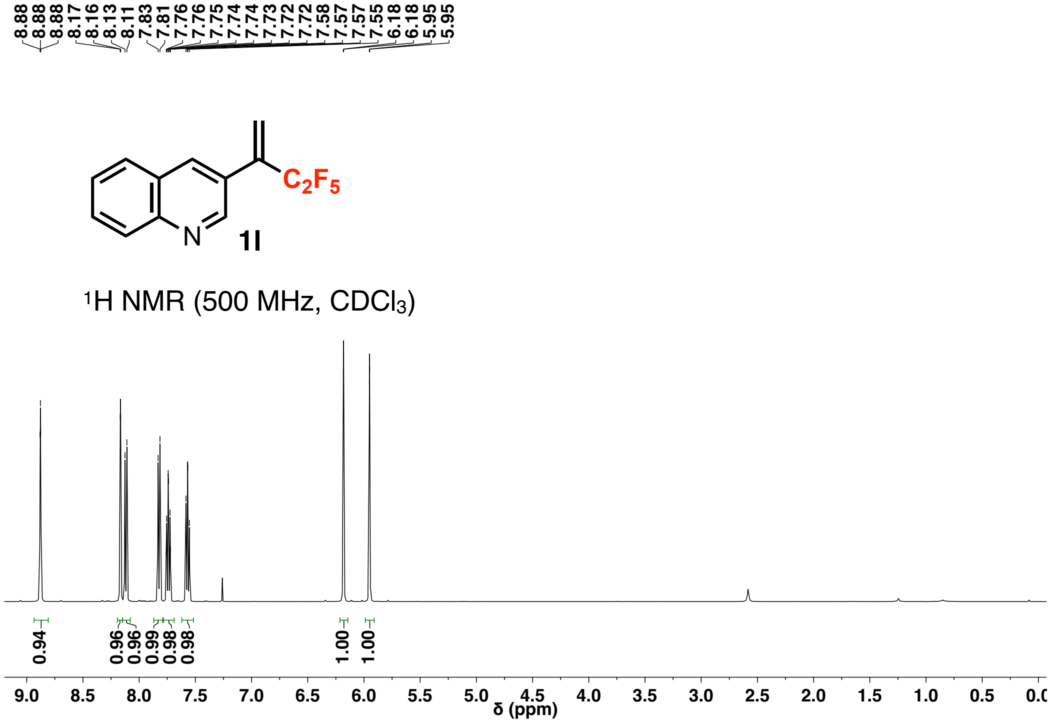
8.88
8.88
8.88
8.17
8.16
8.13
8.11
7.83
7.81
7.76
7.76
7.75
7.74
7.74
7.73
7.72
7.72
7.58
7.57
7.57
7.55
6.18
6.18
5.95
5.95
C2F5
N
1l
1H NMR (500 MHz, CDCl3)
0.94
0.96
0.96
0.99
0.98
0.98
1.00
1.00
9.0 8.5 8.0 7.5 7.0 6.5 6.0 5.5 5.0 4.5 4.0 3.5 3.0 2.5 2.0 1.5 1.0 0.5 0.0
δ (ppm)

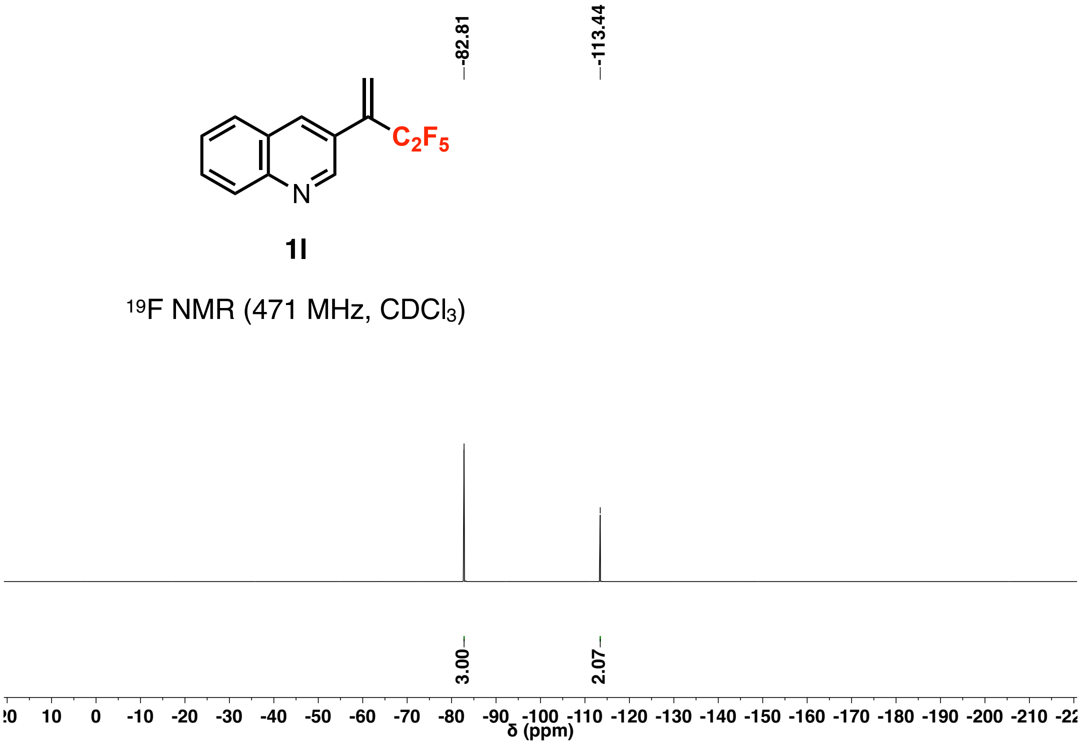
-82.81
-113.44
C2F5
N
1l
19F NMR (471 MHz, CDCl3)
3.00
2.07
20 10 0 -10 -20 -30 -40 -50 -60 -70 -80 -90 -100 -110 -120 -130 -140 -150 -160 -170 -180 -190 -200 -210 -220
δ (ppm)

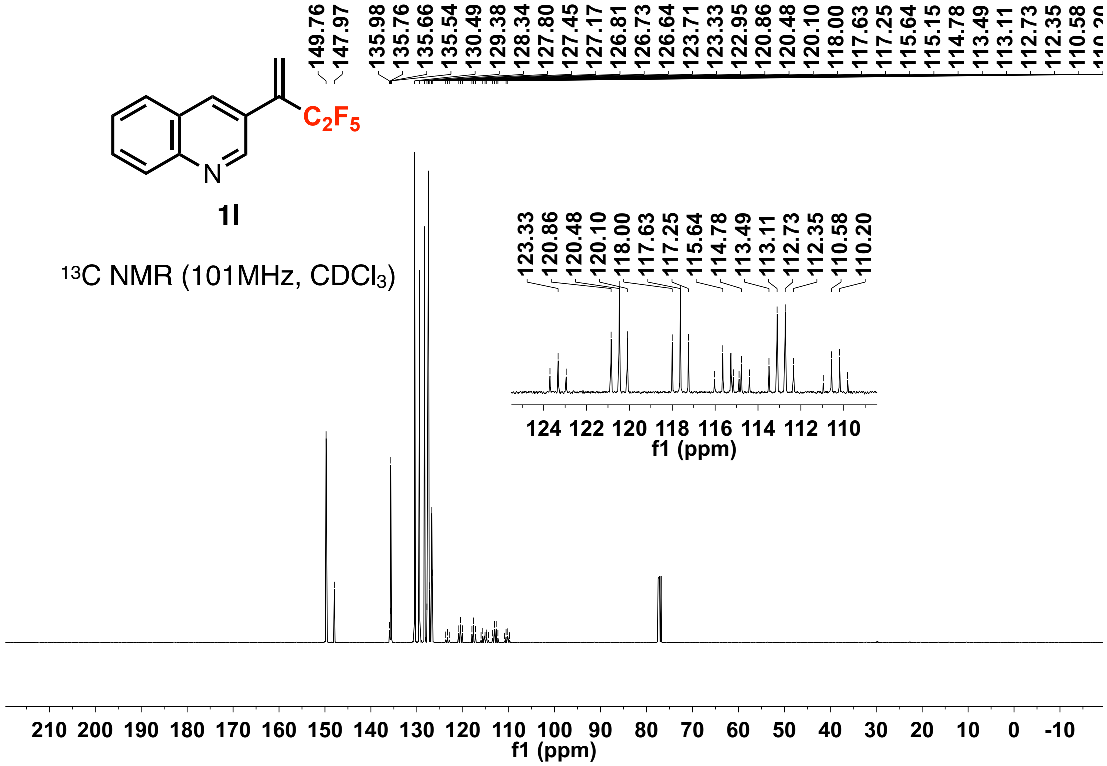
149.76
147.97
135.98
135.76
135.66
135.54
130.49
129.38
128.34
127.80
127.45
127.17
126.81
126.73
126.64
123.71
123.33
122.95
120.86
120.48
120.10
118.00
117.63
117.25
115.64
115.15
114.78
113.49
113.11
112.73
112.35
110.58
C_2F_5
N
1l
^{13}C NMR (101MHz, $CDCl_3$)
123.33
120.86
120.48
120.10
118.00
117.63
117.25
115.64
114.78
113.49
113.11
112.73
112.35
110.58
110.20
124 122 120 118 116 114 112 110
f1 (ppm)
210 200 190 180 170 160 150 140 130 120 110 100 90 80 70 60 50 40 30 20 10 0 -10
f1 (ppm)

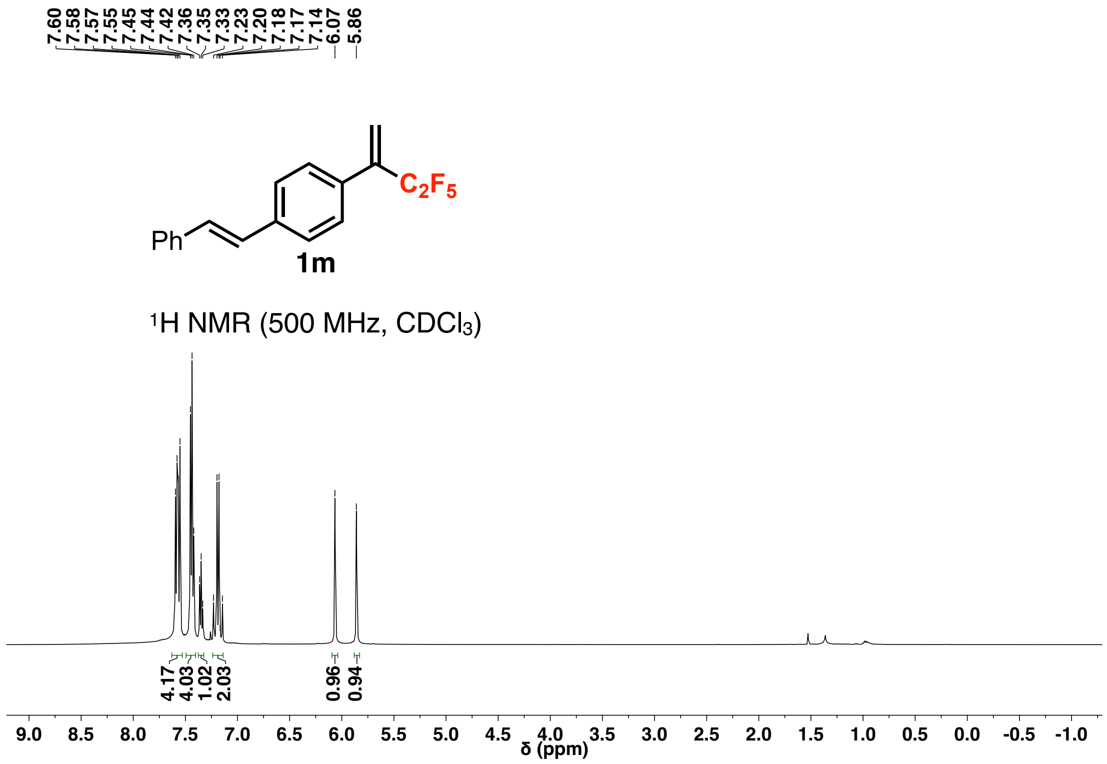
7.60
7.58
7.57
7.55
7.45
7.44
7.42
7.36
7.35
7.33
7.23
7.20
7.18
7.17
7.14
6.07
5.86
C_2F_5
Ph
1m
^{1}H NMR (500 MHz, $CDCl_3$)
4.17
4.03
1.02
2.03
0.96
0.94
9.0 8.5 8.0 7.5 7.0 6.5 6.0 5.5 5.0 4.5 4.0 3.5 3.0 2.5 2.0 1.5 1.0 0.5 0.0 -0.5 -1.0
δ (ppm)

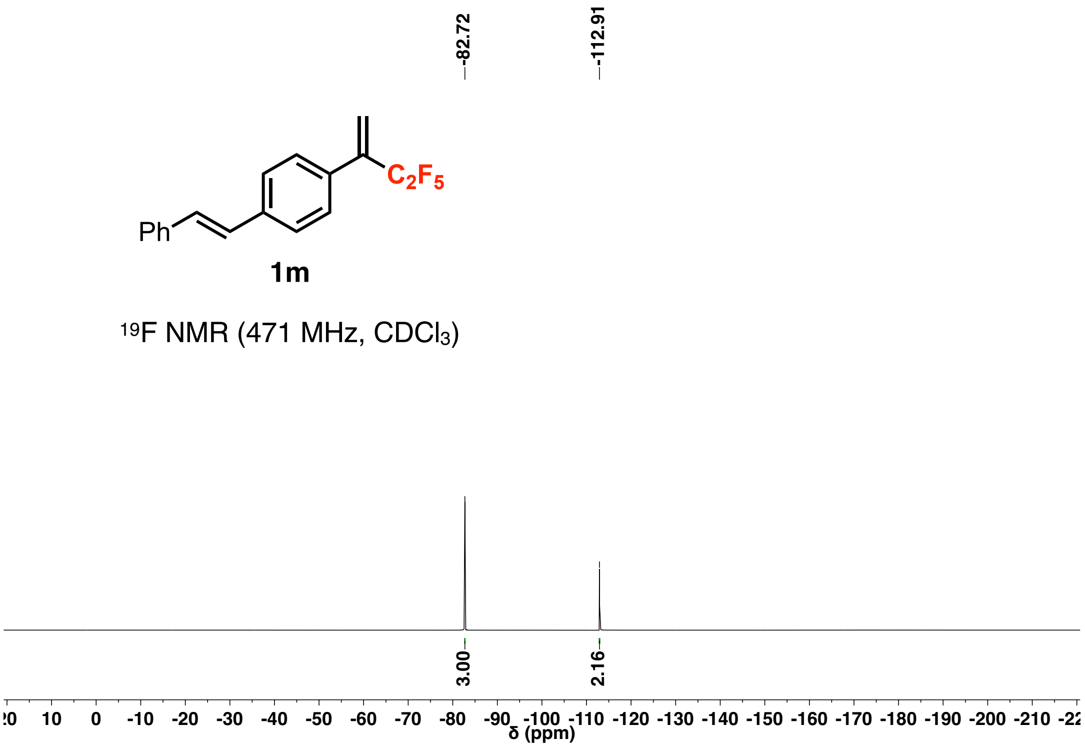

1m

^{19}F NMR (471 MHz, $CDCl_3$)

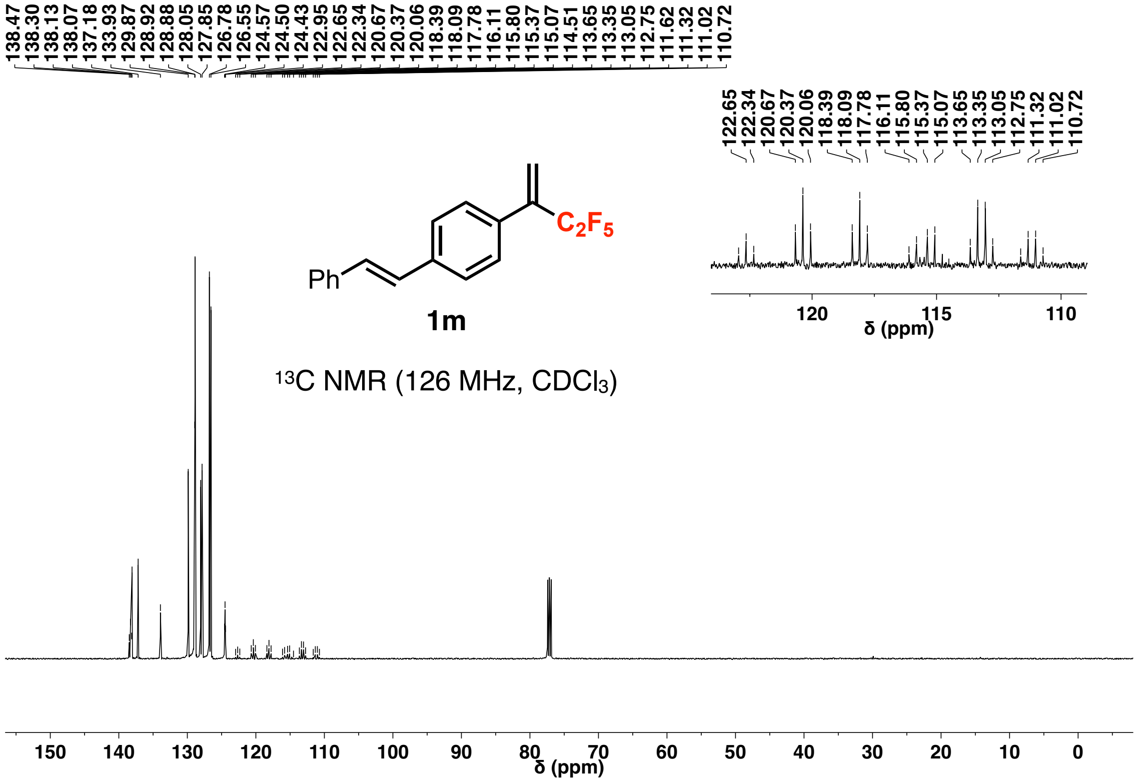

1m

^{13}C NMR (126 MHz, $CDCl_3$)

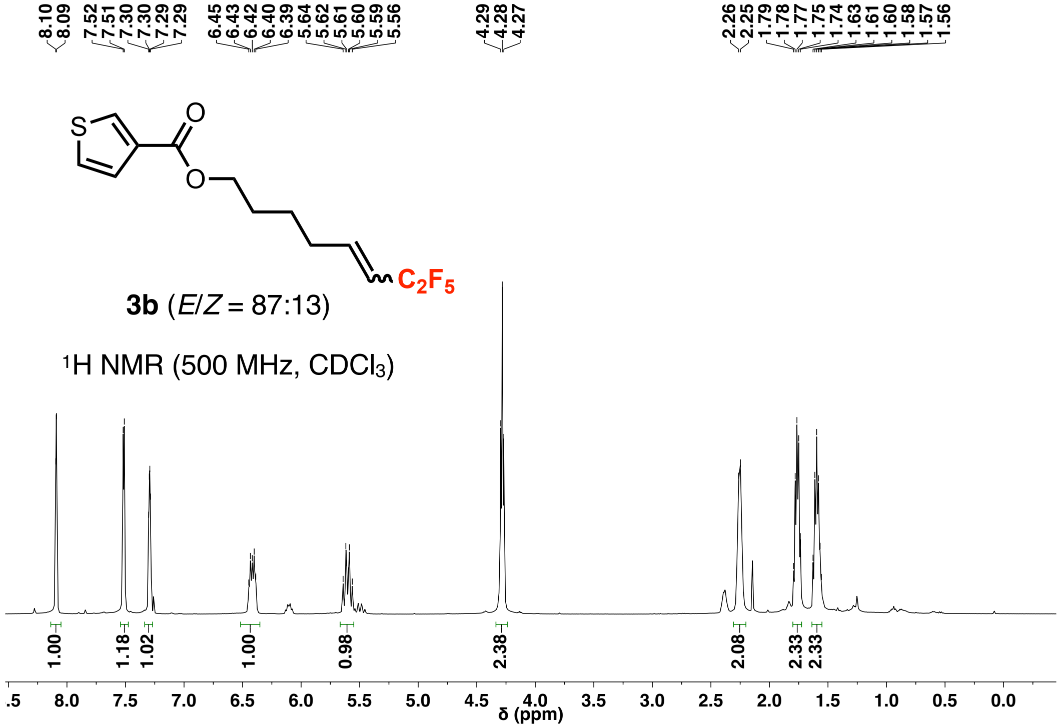

3b (E/Z = 87:13)
1H NMR (500 MHz, CDCl3)
δ (ppm)

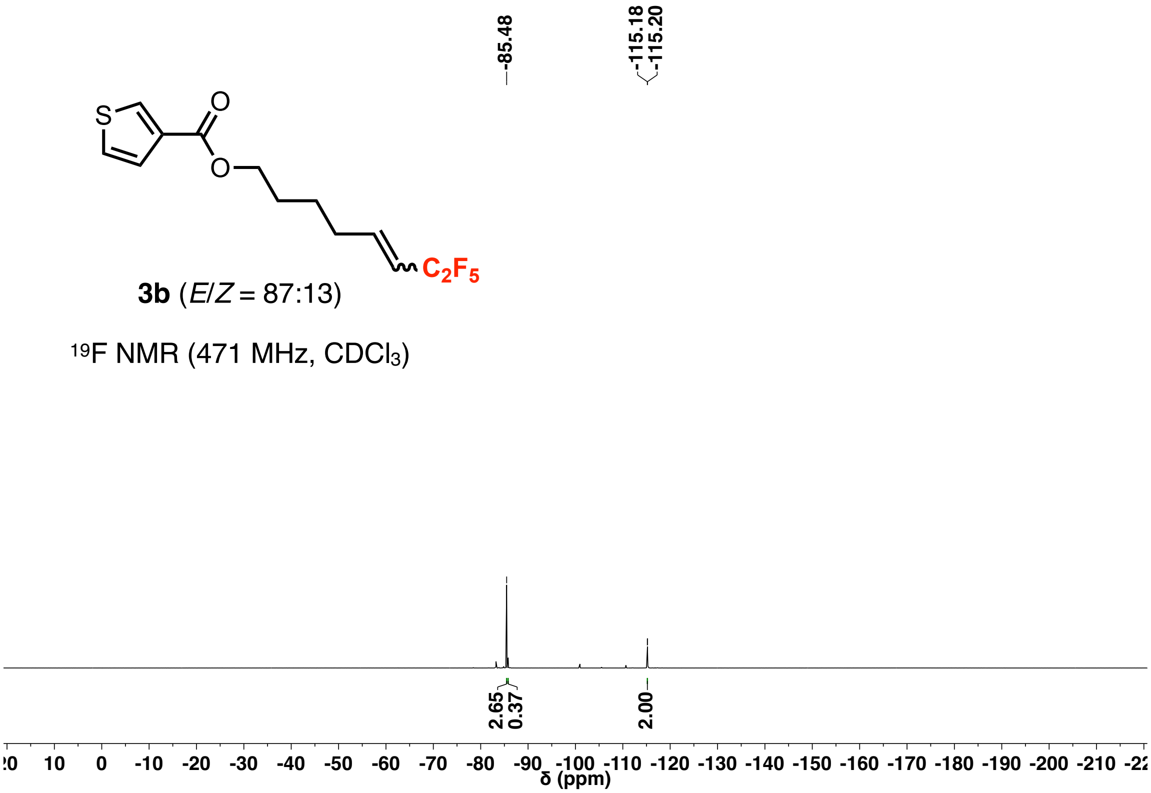

3b (E/Z = 87:13)
19F NMR (471 MHz, CDCl3)
δ (ppm)

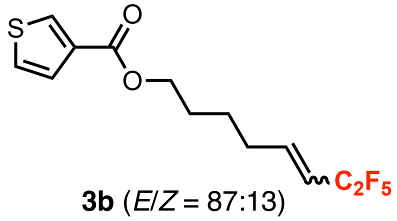

3b (*E*/*Z* = 87:13)

^{13}C NMR (126 MHz, CDCl$_3$)

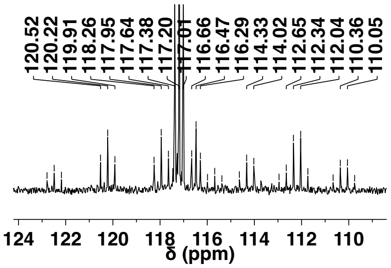

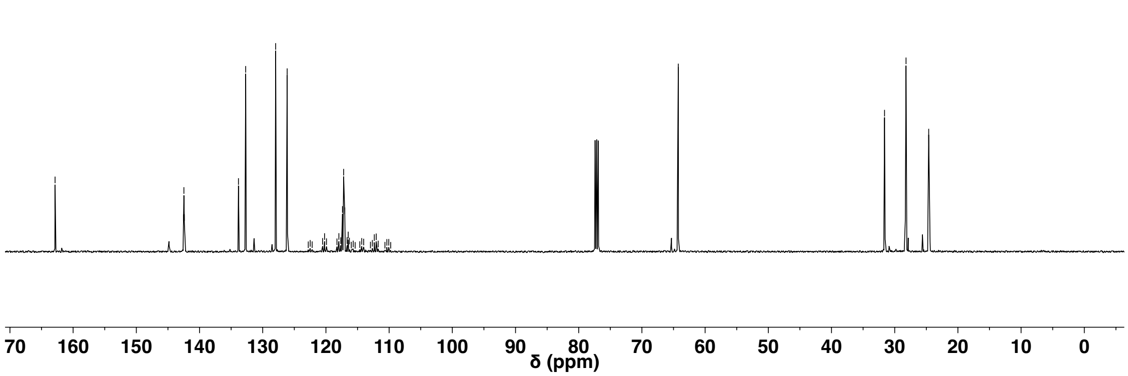

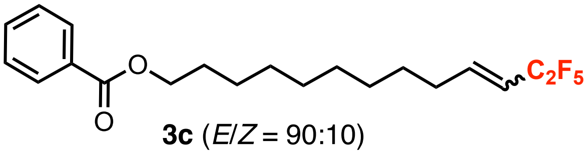

3c (*E*/*Z* = 90:10)

^{1}H NMR (500 MHz, CDCl$_3$)

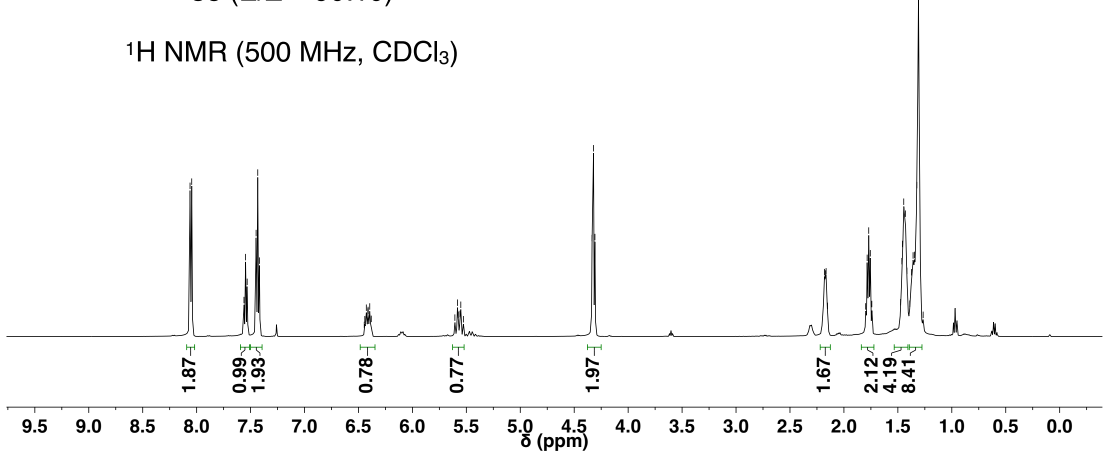

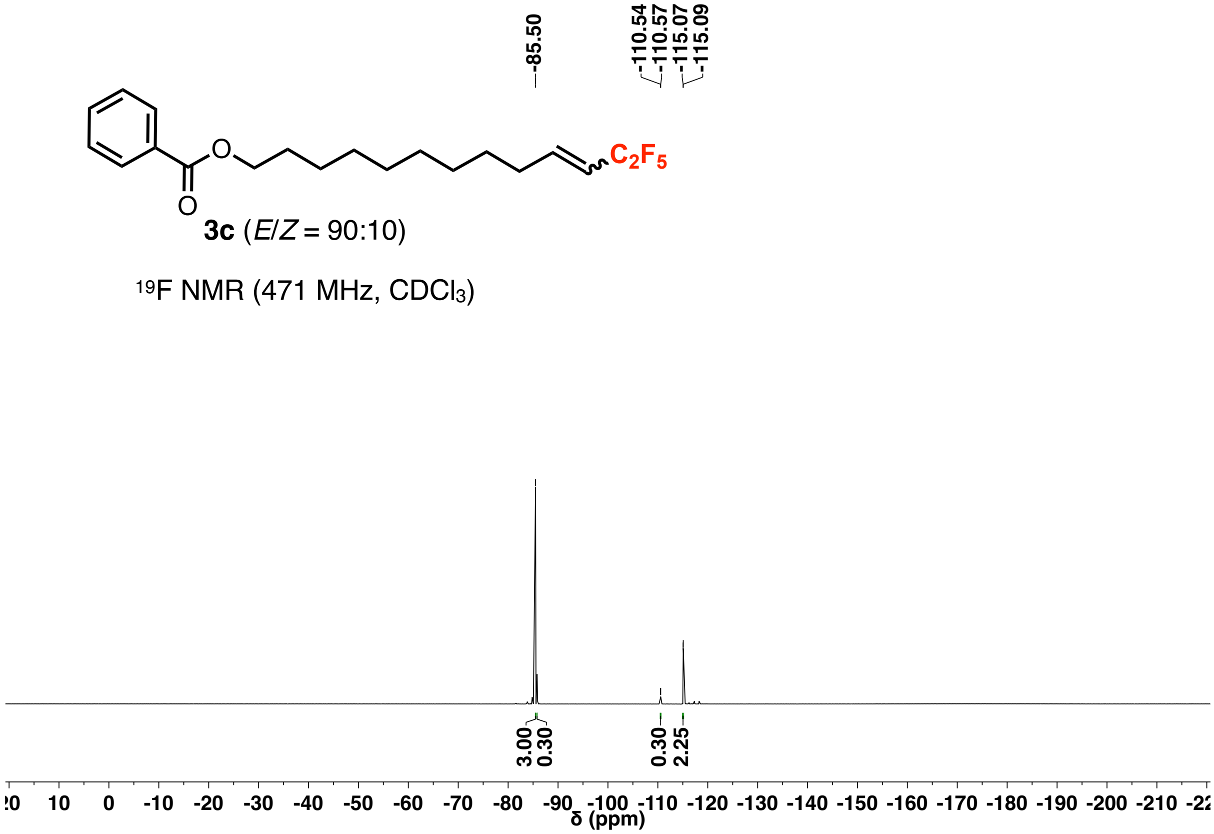

-85.50
-110.54
-110.57
-115.07
-115.09
C2F5
O
O
3c (E/Z = 90:10)
19F NMR (471 MHz, CDCl3)
3.00
0.30
0.30
2.25
20 10 0 -10 -20 -30 -40 -50 -60 -70 -80 -90 -100 -110 -120 -130 -140 -150 -160 -170 -180 -190 -200 -210 -220
δ (ppm)

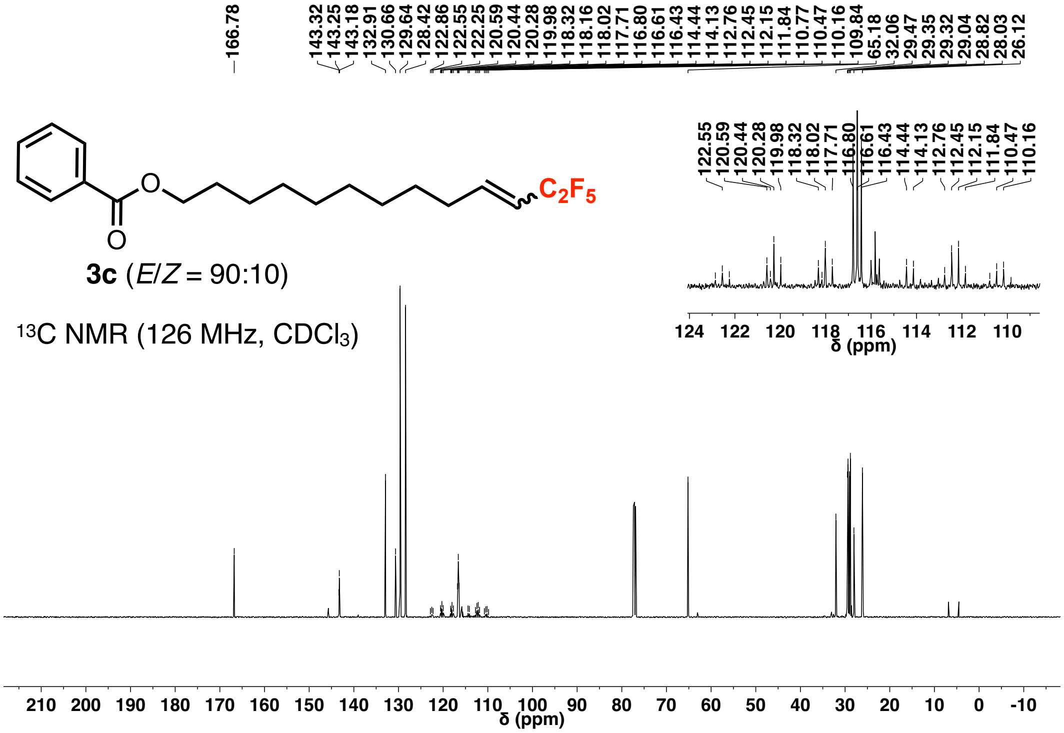

166.78
143.32
143.25
143.18
132.91
130.66
129.64
128.42
122.86
122.55
122.25
120.59
120.44
120.28
119.98
118.32
118.16
118.02
117.71
116.80
116.61
116.43
114.44
114.13
112.76
112.45
112.15
111.84
110.77
110.47
110.16
109.84
65.18
32.06
29.47
29.35
29.32
29.04
28.82
28.03
26.12
C2F5
O
O
3c (E/Z = 90:10)
13C NMR (126 MHz, CDCl3)
122.55
120.59
120.44
120.28
119.98
118.32
118.02
117.71
116.80
116.61
116.43
114.44
114.13
112.76
112.45
112.15
111.84
110.47
110.16
124 122 120 118 116 114 112 110
δ (ppm)
210 200 190 180 170 160 150 140 130 120 110 100 90 80 70 60 50 40 30 20 10 0 -10
δ (ppm)

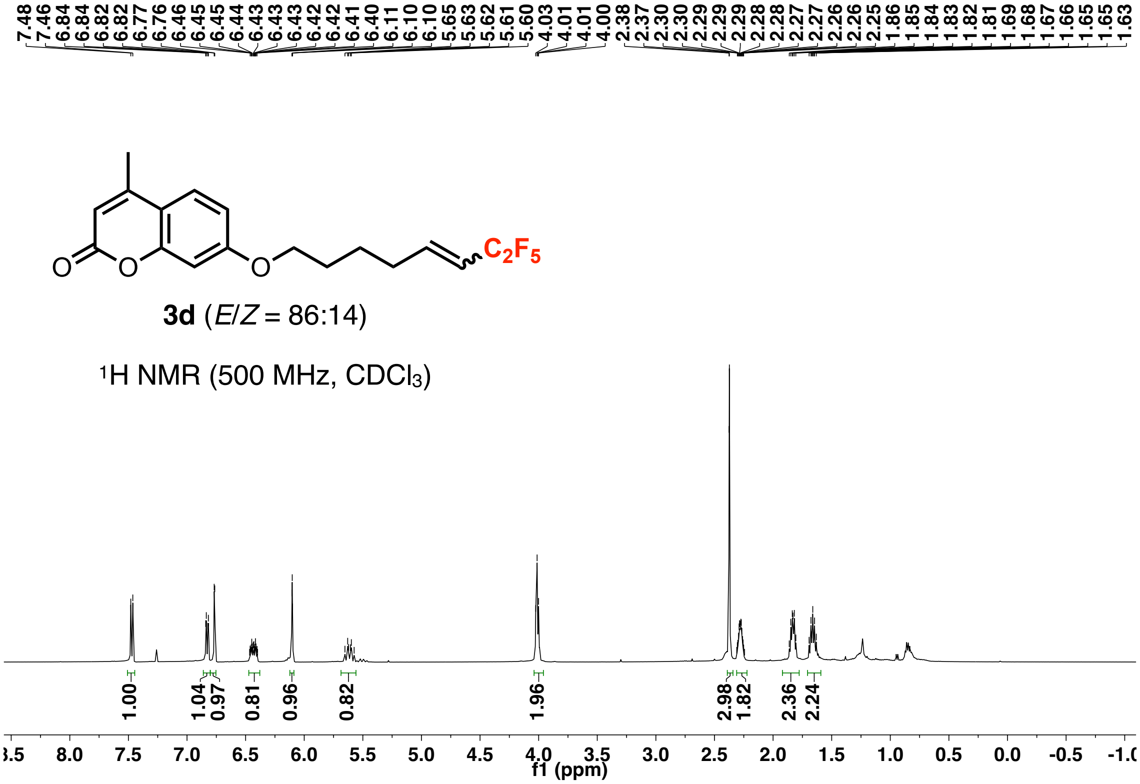
7.48
7.46
6.84
6.84
6.82
6.82
6.77
6.76
6.46
6.45
6.45
6.44
6.43
6.43
6.43
6.42
6.42
6.41
6.40
6.11
6.10
6.10
5.65
5.63
5.62
5.61
5.60
4.03
4.01
4.01
4.00
2.38
2.37
2.30
2.30
2.29
2.29
2.29
2.28
2.28
2.27
2.27
2.26
2.26
2.25
1.86
1.85
1.84
1.83
1.82
1.81
1.69
1.68
1.67
1.66
1.65
1.65
1.63
O
O
O
C_2F_5
3d (E/Z = 86:14)
^{1}H NMR (500 MHz, CDCl$_3$)
1.00
1.04
0.97
0.81
0.96
0.82
1.96
2.98
1.82
2.36
2.24
f1 (ppm)

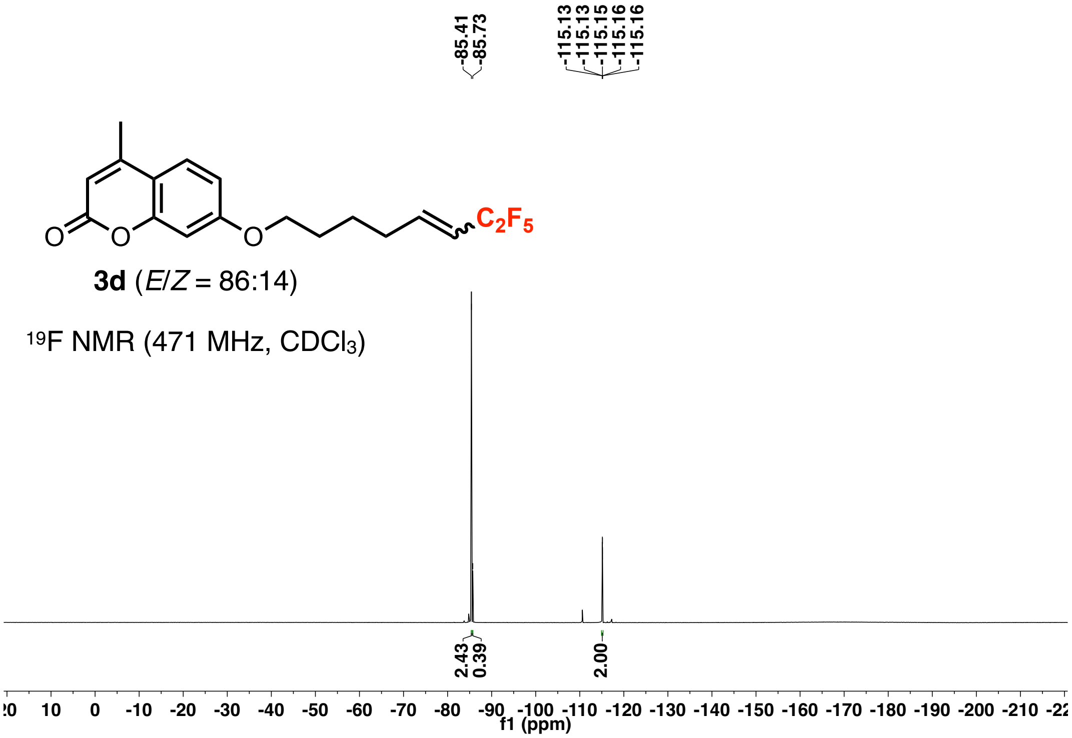
-85.41
-85.73
-115.13
-115.13
-115.15
-115.16
-115.16
O
O
O
C_2F_5
3d (E/Z = 86:14)
^{19}F NMR (471 MHz, CDCl$_3$)
2.43
0.39
2.00
f1 (ppm)

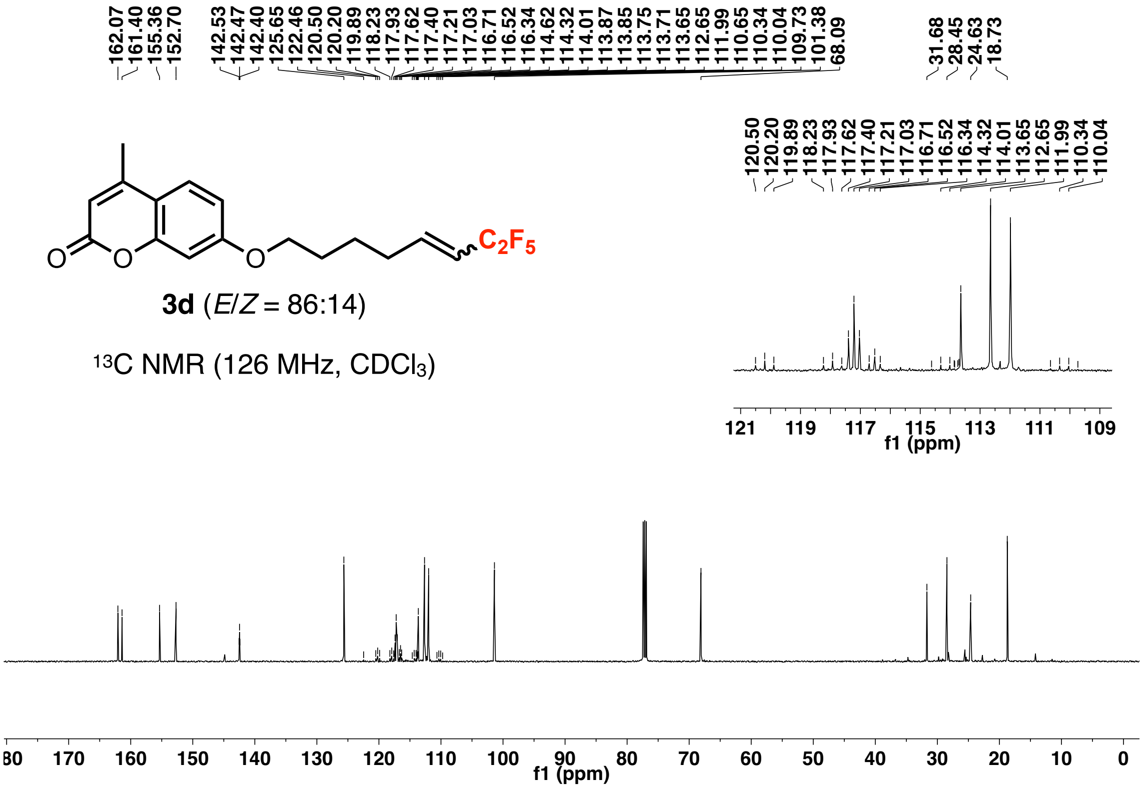
162.07
161.40
155.36
152.70
142.53
142.47
142.40
125.65
122.46
120.50
120.20
119.89
118.23
117.93
117.62
117.40
117.21
117.03
116.71
116.52
116.34
114.62
114.32
114.01
113.87
113.85
113.75
113.71
113.65
112.65
111.99
110.65
110.34
110.04
109.73
101.38
68.09
31.68
28.45
24.63
18.73
C2F5
3d (E/Z = 86:14)
13C NMR (126 MHz, CDCl3)
f1 (ppm)

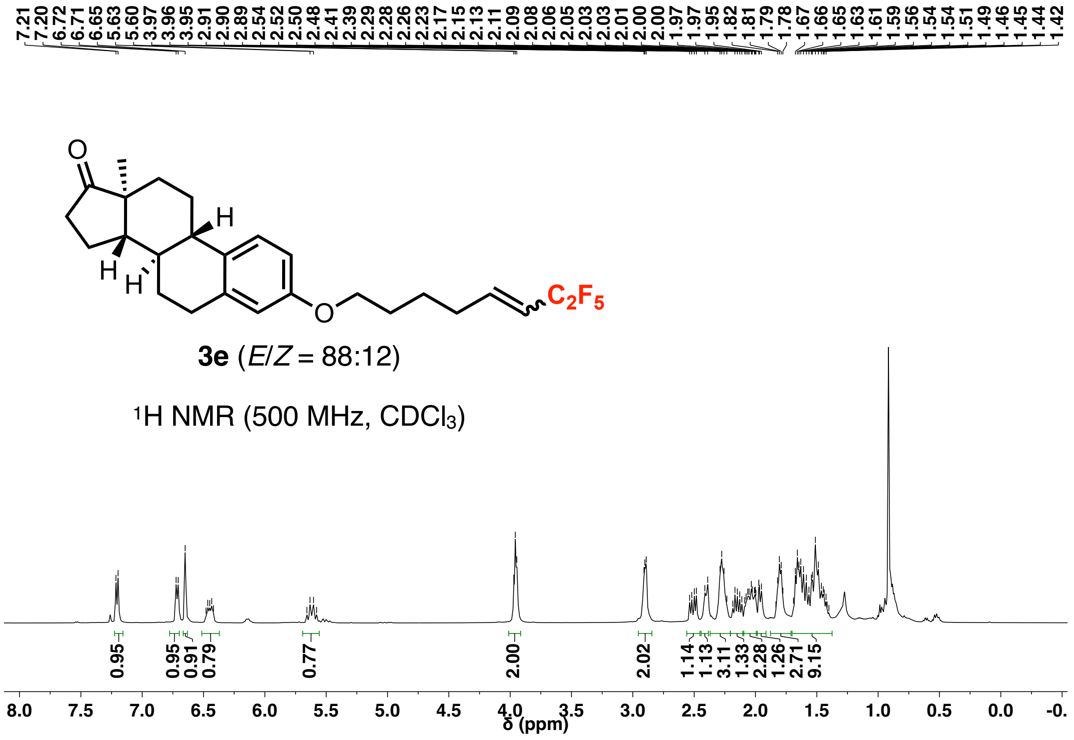
7.21
7.20
6.72
6.71
6.65
5.63
5.60
3.97
3.96
3.95
2.91
2.90
2.89
2.54
2.52
2.50
2.48
2.41
2.39
2.29
2.28
2.26
2.23
2.17
2.15
2.13
2.11
2.09
2.08
2.06
2.05
2.03
2.03
2.01
2.00
1.97
1.95
1.82
1.81
1.79
1.78
1.67
1.66
1.65
1.63
1.61
1.59
1.56
1.54
1.51
1.49
1.46
1.45
1.44
1.42
O
H
H
H
O
C2F5
3e (E/Z = 88:12)
1H NMR (500 MHz, CDCl3)
0.95
0.95
0.91
0.79
0.77
2.00
2.02
1.14
1.13
3.11
1.33
2.28
1.26
2.71
9.15
δ (ppm)

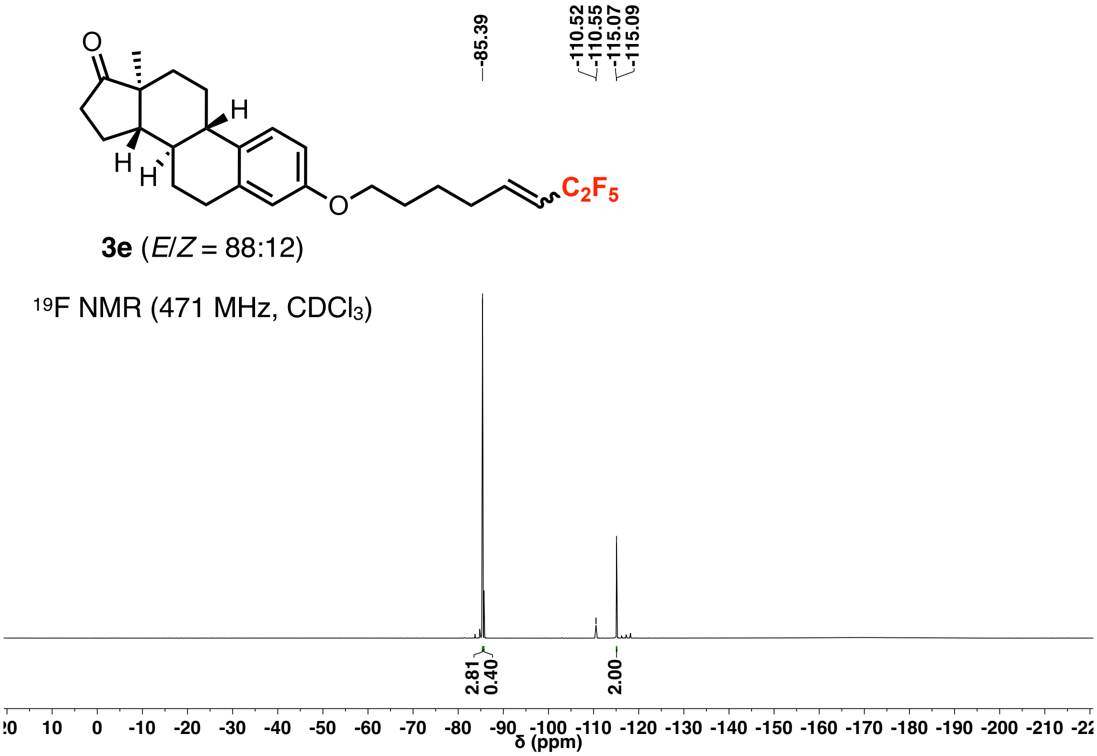

-85.39
-110.52
-110.55
-115.07
-115.09
3e (E/Z = 88:12)
19F NMR (471 MHz, CDCl3)
2.81
0.40
2.00
δ (ppm)

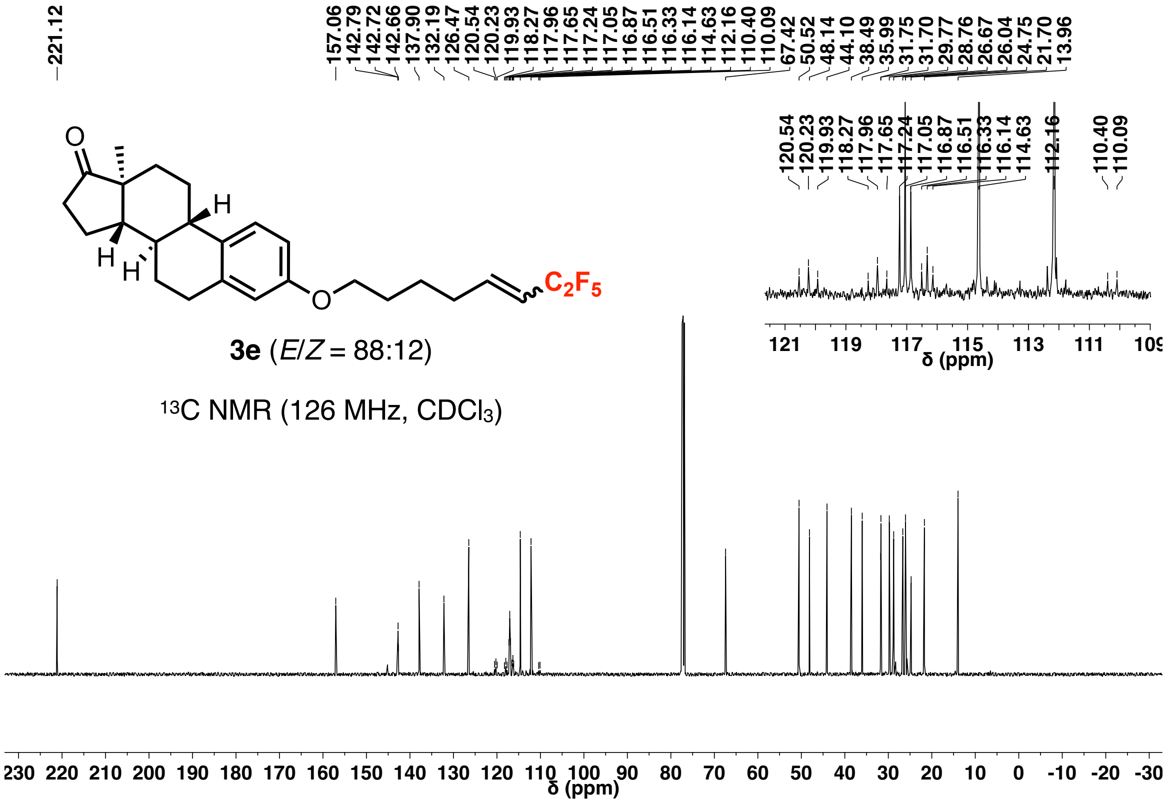

221.12
157.06
142.79
142.72
142.66
137.90
132.19
126.47
120.54
120.23
119.93
118.27
117.96
117.65
117.24
117.05
116.87
116.51
116.33
116.14
114.63
112.16
110.40
110.09
67.42
50.52
48.14
44.10
38.49
35.99
31.75
31.70
29.77
28.76
26.67
26.04
24.75
21.70
13.96
3e (E/Z = 88:12)
13C NMR (126 MHz, CDCl3)
δ (ppm)

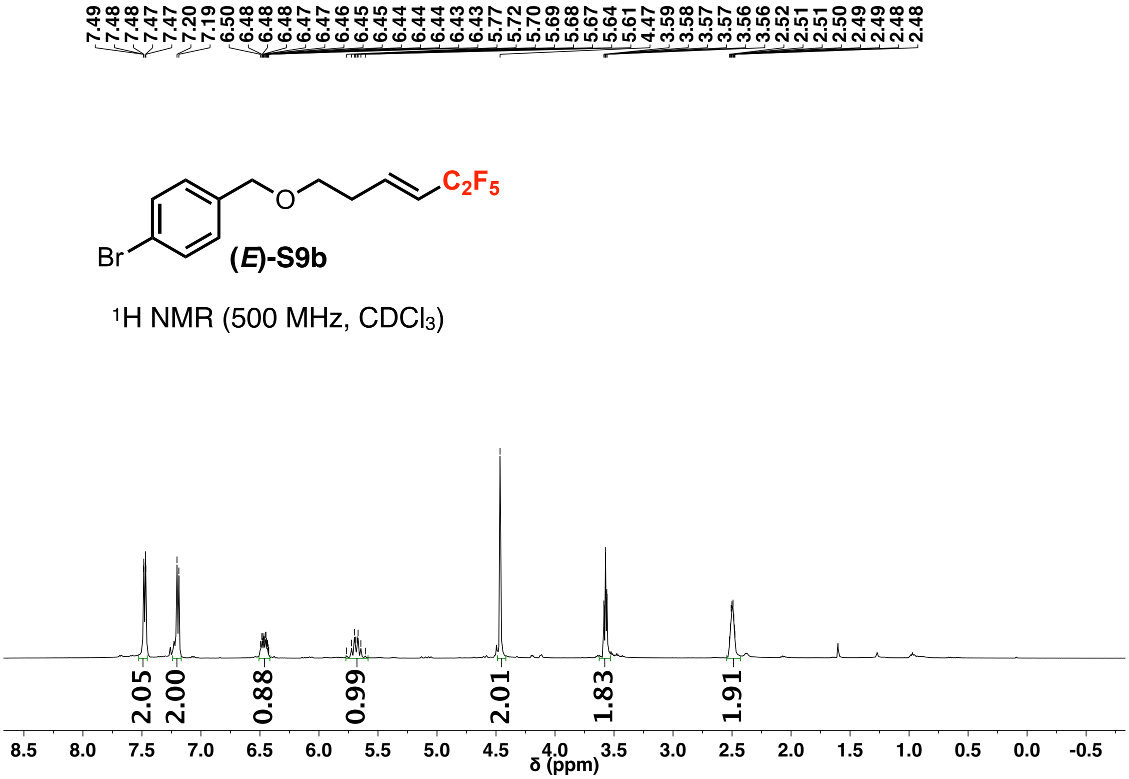

(E)-S9b
C2F5
Br
O
1H NMR (500 MHz, CDCl3)
2.05
2.00
0.88
0.99
2.01
1.83
1.91
δ (ppm)

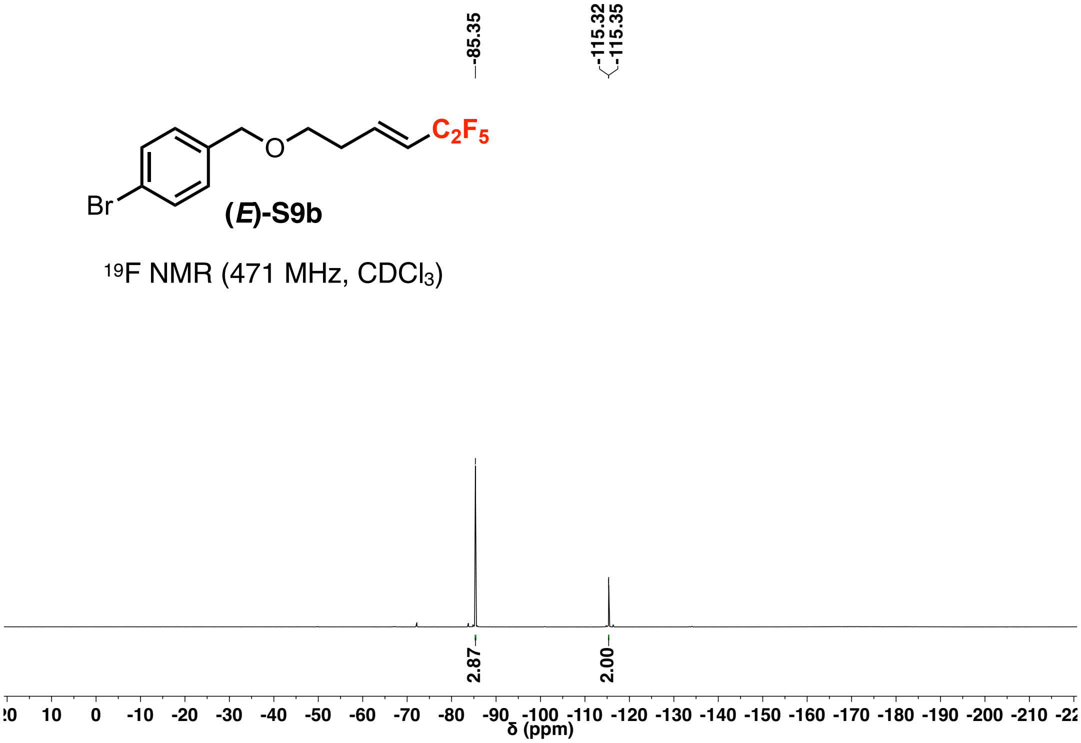

-85.35
-115.32
-115.35
(E)-S9b
C2F5
Br
O
19F NMR (471 MHz, CDCl3)
2.87
2.00
δ (ppm)

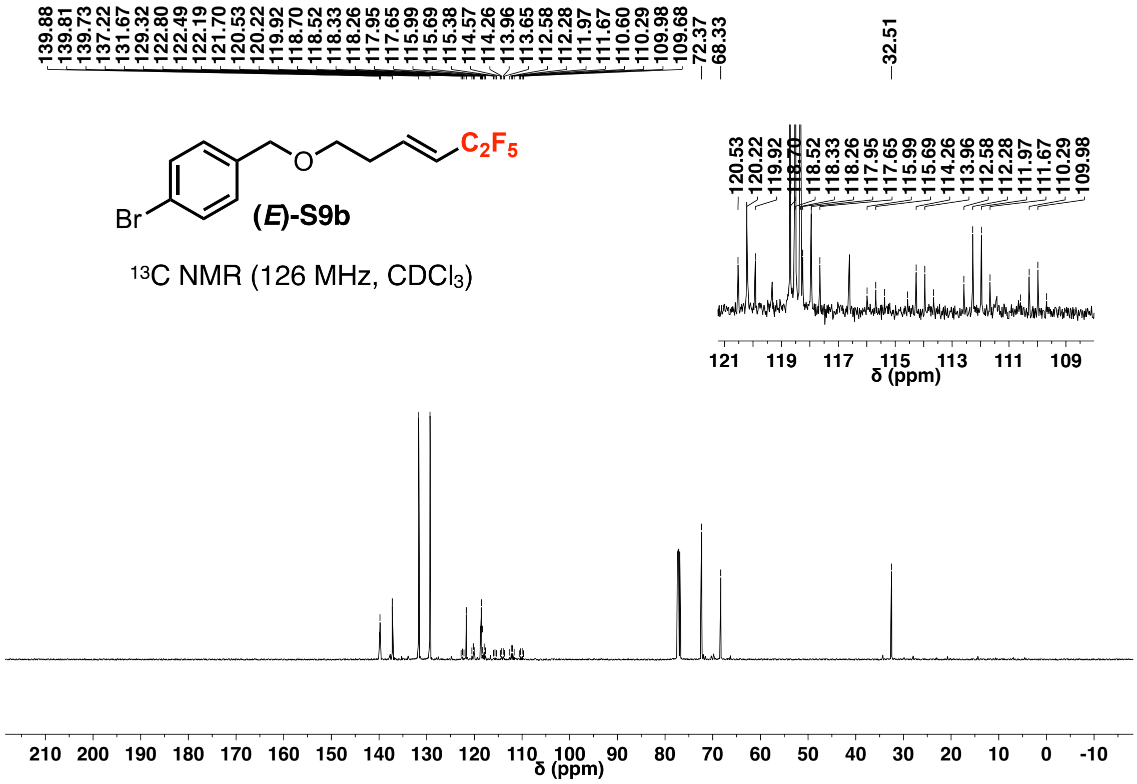
139.88 139.81 139.73 137.22 131.67 129.32 122.80 122.49 122.19 121.70 120.53 120.22 119.92 118.70 118.52 118.33 118.26 117.95 117.65 115.99 115.69 115.38 114.57 114.26 113.96 113.65 112.58 112.28 111.97 111.67 110.60 110.29 109.98 109.68 72.37 68.33
32.51
C2F5
Br
O
(E)-S9b
13C NMR (126 MHz, CDCl3)
120.53 120.22 119.92 118.70 118.52 118.33 118.26 117.95 117.65 115.99 115.69 114.26 113.96 112.58 112.28 111.97 111.67 110.29 109.98
δ (ppm)
δ (ppm)

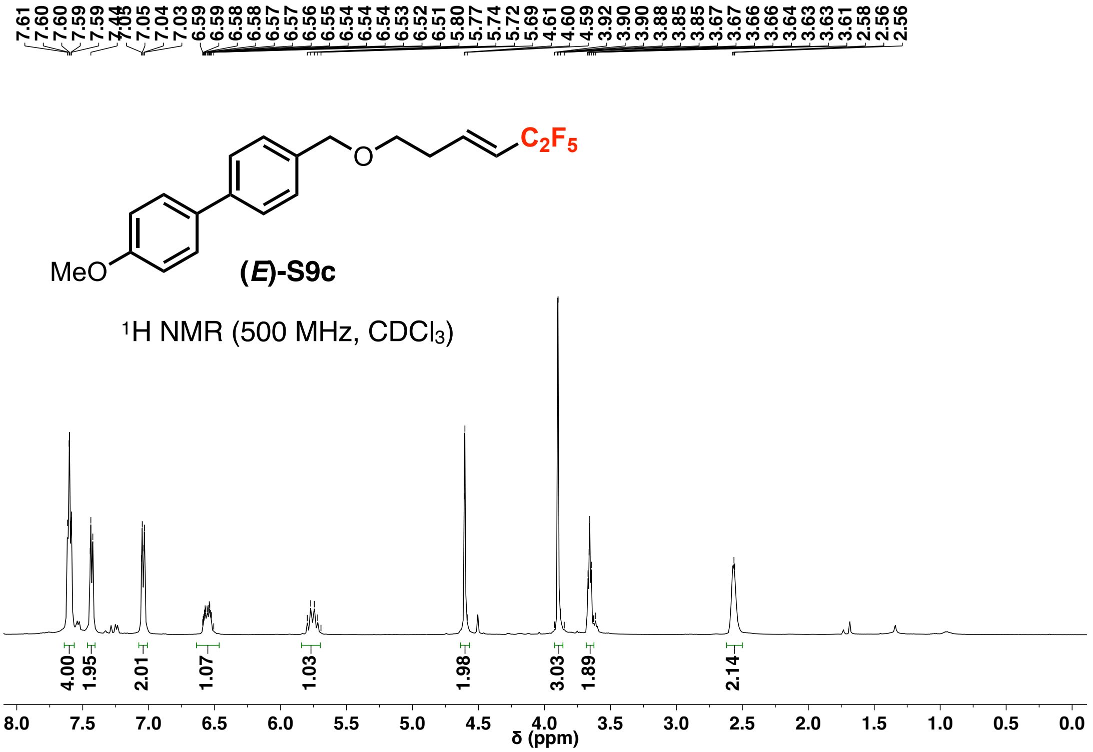
7.61 7.60 7.60 7.59 7.59 7.44 7.45 7.05 7.04 7.03 6.59 6.59 6.58 6.57 6.56 6.55 6.54 6.54 6.53 6.52 6.51 5.80 5.77 5.74 5.72 5.69 4.61 4.60 4.59 3.92 3.90 3.88 3.85 3.67 3.67 3.66 3.64 3.63 3.63 3.61 2.58 2.56 2.56
C2F5
O
MeO
(E)-S9c
1H NMR (500 MHz, CDCl3)
4.00 1.95 2.01 1.07 1.03 1.98 3.03 1.89 2.14
δ (ppm)

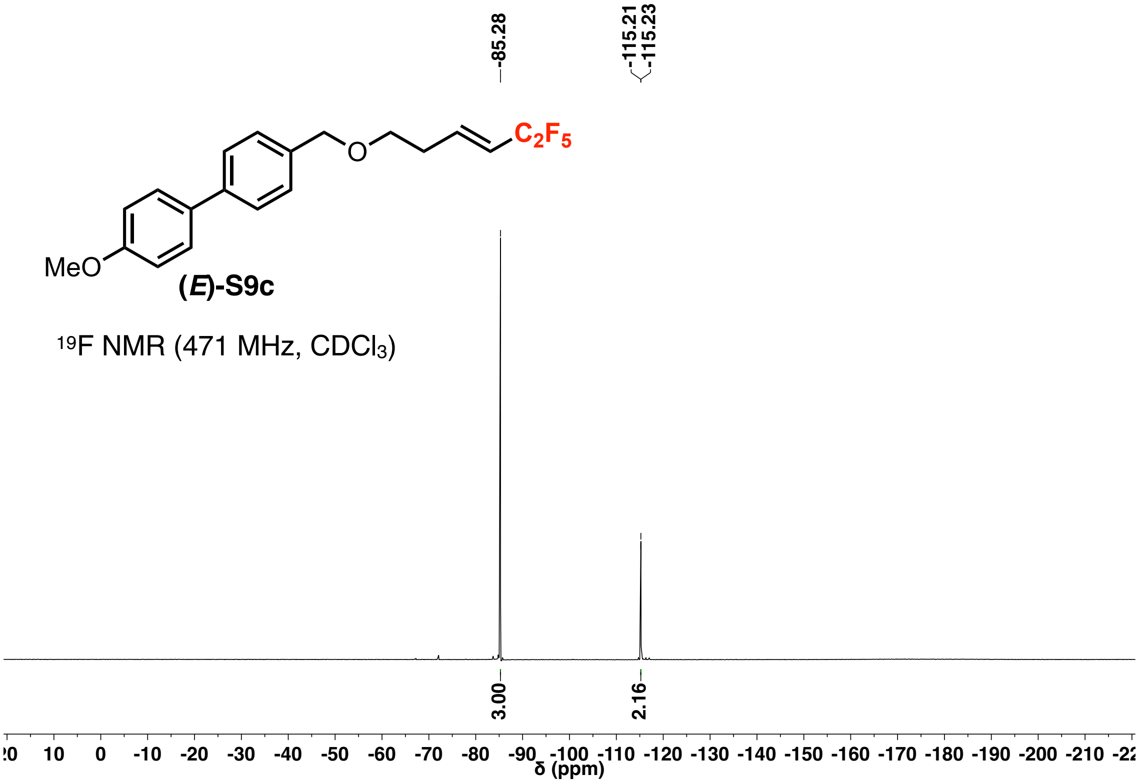
-85.28
-115.21
-115.23
C2F5
O
MeO
(E)-S9c
19F NMR (471 MHz, CDCl3)
3.00
2.16
δ (ppm)

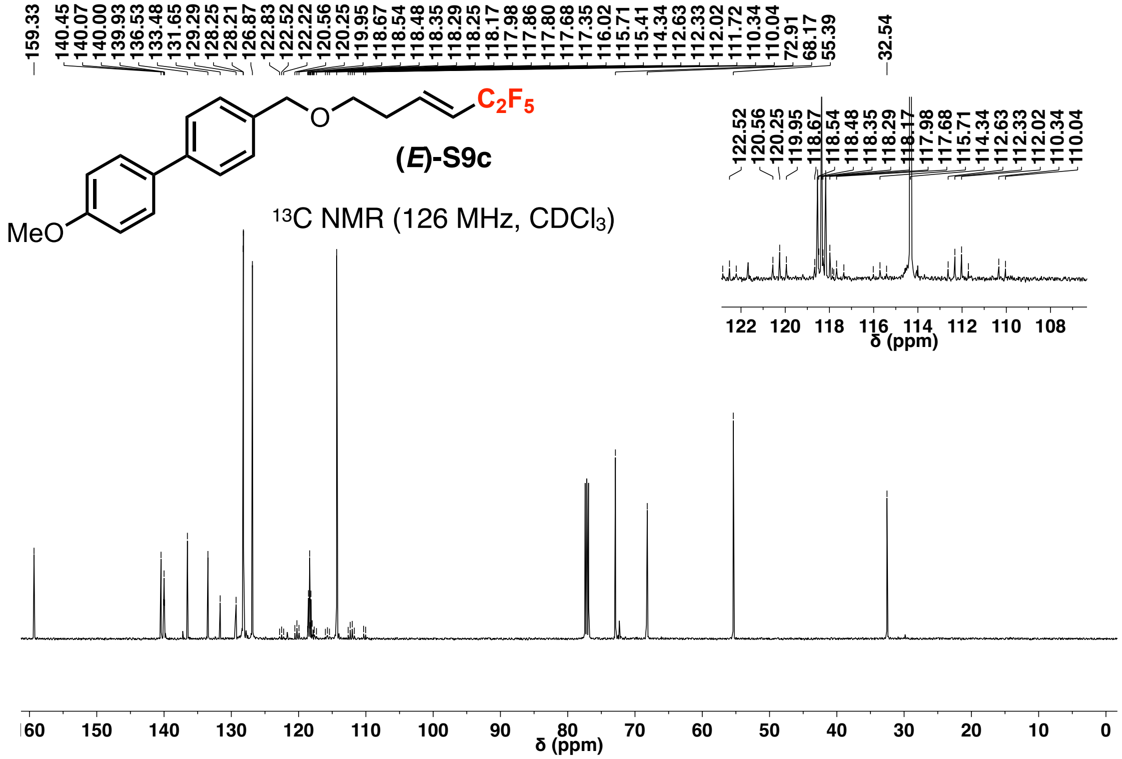
159.33
140.45
140.07
140.00
139.93
136.53
133.48
131.65
129.29
128.25
128.21
126.87
122.83
122.52
122.22
120.56
120.25
119.95
118.67
118.54
118.48
118.35
118.29
118.25
118.17
117.98
117.86
117.80
117.68
117.35
116.02
115.71
115.41
114.34
112.63
112.33
112.02
111.72
110.34
110.04
72.91
68.17
55.39
32.54
C2F5
O
MeO
(E)-S9c
13C NMR (126 MHz, CDCl3)
δ (ppm)

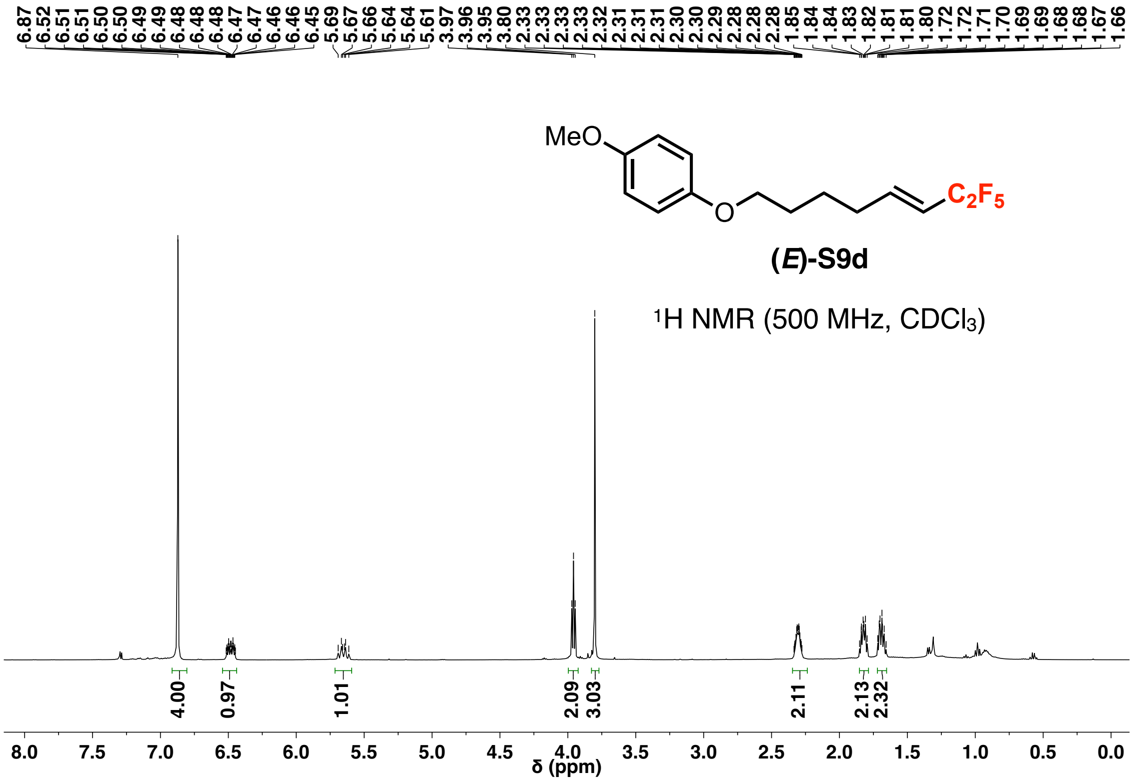
MeO
O
C2F5
(E)-S9d
1H NMR (500 MHz, CDCl3)
δ (ppm)

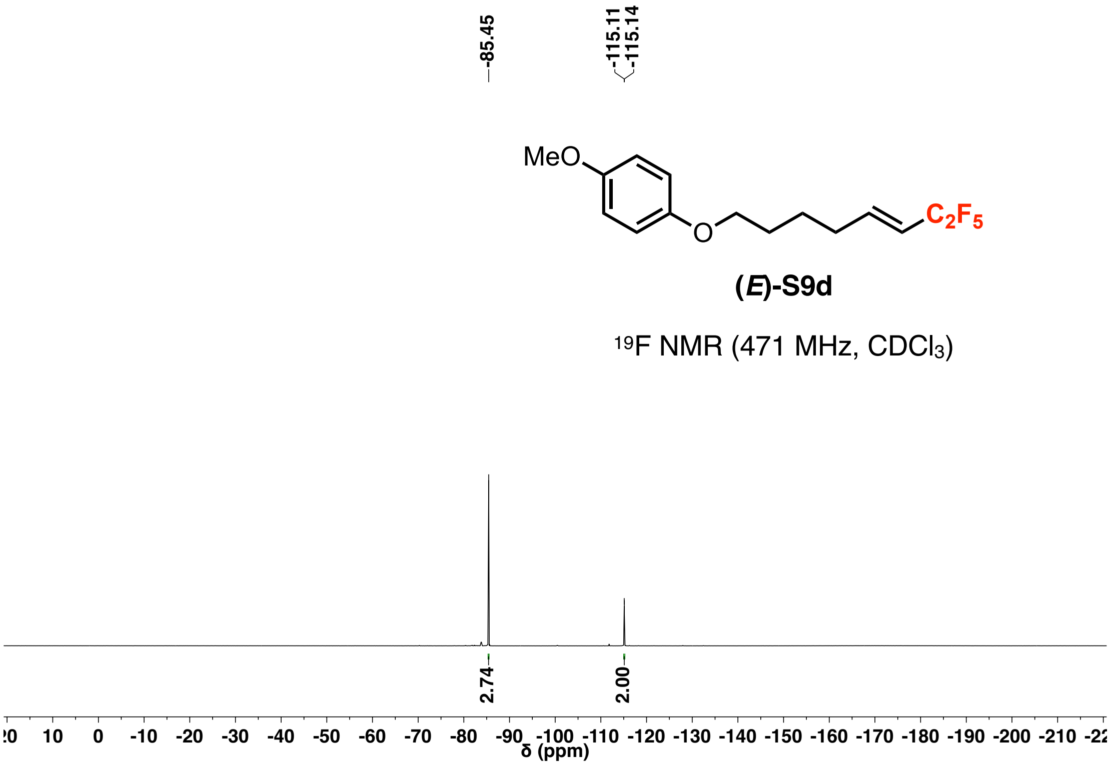
-85.45
-115.11
-115.14
MeO
O
C2F5
(E)-S9d
19F NMR (471 MHz, CDCl3)
2.74
2.00
δ (ppm)

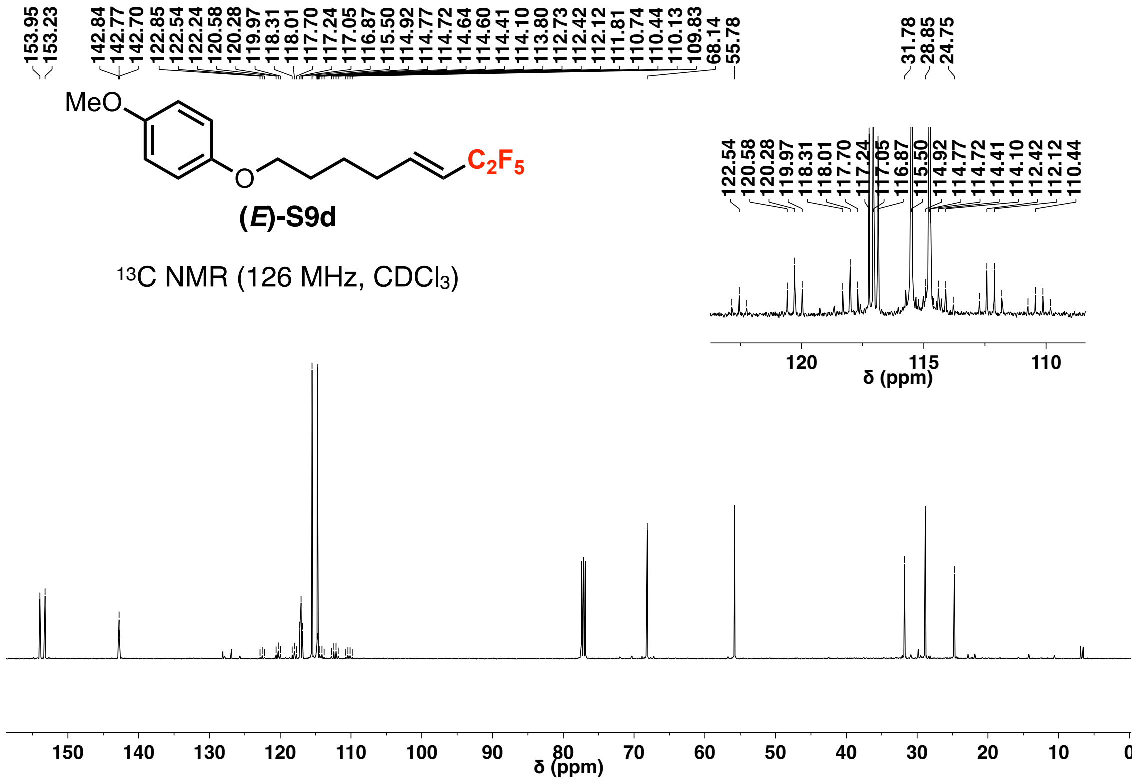

153.95
153.23
142.84
142.77
142.70
122.85
122.54
122.24
120.58
120.28
119.97
118.31
118.01
117.70
117.24
117.05
116.87
115.50
114.92
114.77
114.72
114.64
114.60
114.41
114.10
113.80
112.73
112.42
112.12
111.81
110.74
110.44
110.13
109.83
68.14
55.78
31.78
28.85
24.75
MeO
O
C_2F_5
(*E*)-S9d
^{13}C NMR (126 MHz, $CDCl_3$)
122.54
120.58
120.28
119.97
118.31
118.01
117.70
117.24
117.05
116.87
115.50
114.92
114.77
114.72
114.41
114.10
112.42
112.12
110.44
120
115
110
δ (ppm)
150
140
130
120
110
100
90
80
70
60
50
40
30
20
10
0
δ (ppm)

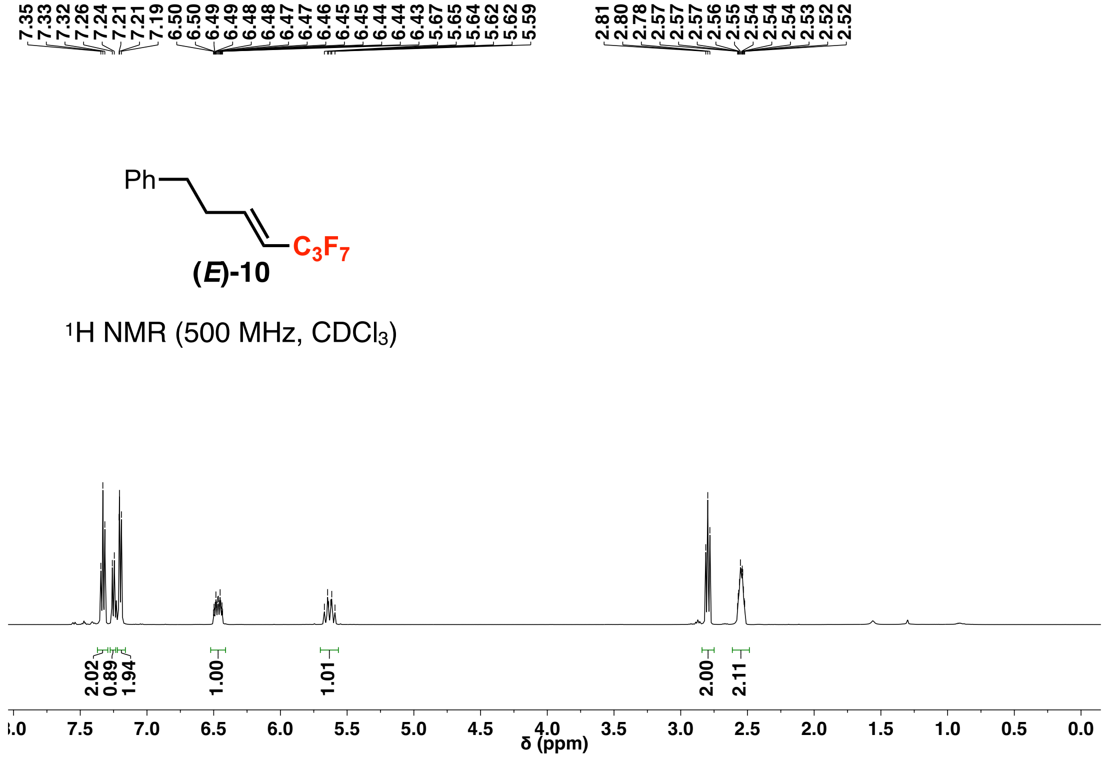

7.35
7.33
7.32
7.26
7.24
7.21
7.21
7.19
6.50
6.50
6.49
6.49
6.48
6.48
6.47
6.47
6.46
6.45
6.45
6.44
6.44
6.43
5.67
5.65
5.64
5.62
5.62
5.59
2.81
2.80
2.78
2.57
2.57
2.56
2.55
2.54
2.54
2.54
2.53
2.52
2.52
Ph
C_3F_7
(*E*)-10
^{1}H NMR (500 MHz, $CDCl_3$)
2.02
0.89
1.94
1.00
1.01
2.00
2.11
7.5
7.0
6.5
6.0
5.5
5.0
4.5
4.0
3.5
3.0
2.5
2.0
1.5
1.0
0.5
0.0
δ (ppm)

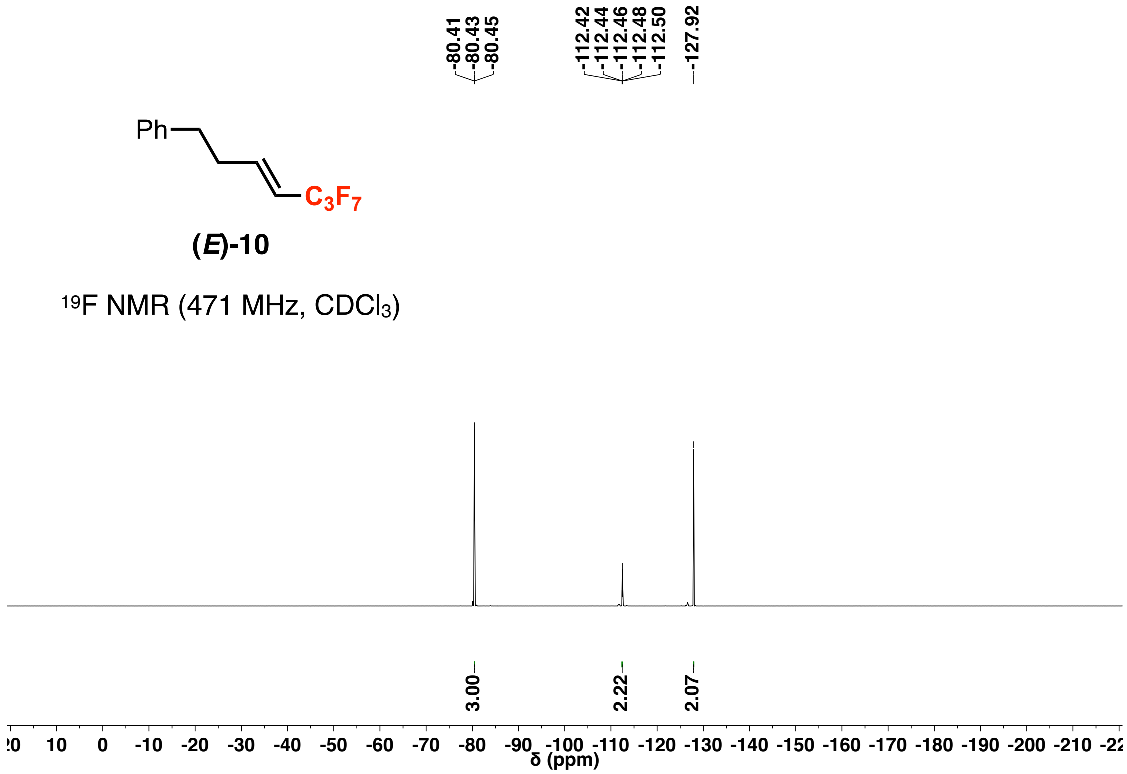
-80.41
-80.43
-80.45
-112.42
-112.44
-112.46
-112.48
-112.50
-127.92
Ph
C_3F_7
(*E*)-10
^{19}F NMR (471 MHz, $CDCl_3$)
3.00
2.22
2.07
δ (ppm)

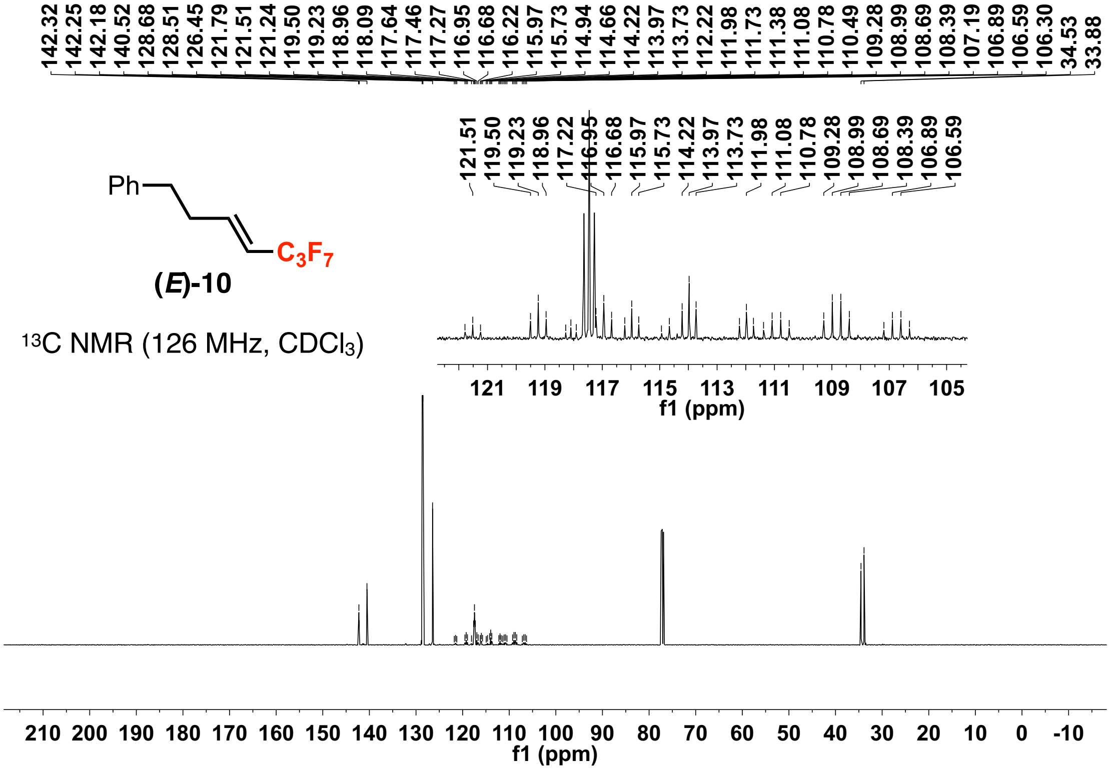
142.32
142.25
142.18
140.52
128.68
128.51
126.45
121.79
121.51
121.24
119.50
119.23
118.96
118.09
117.64
117.46
117.27
116.95
116.68
116.22
115.97
115.73
114.94
114.66
114.22
113.97
113.73
112.22
111.98
111.73
111.38
111.08
110.78
110.49
109.28
108.99
108.69
108.39
107.19
106.89
106.59
106.30
34.53
33.88
Ph
C_3F_7
(*E*)-10
^{13}C NMR (126 MHz, $CDCl_3$)
f1 (ppm)

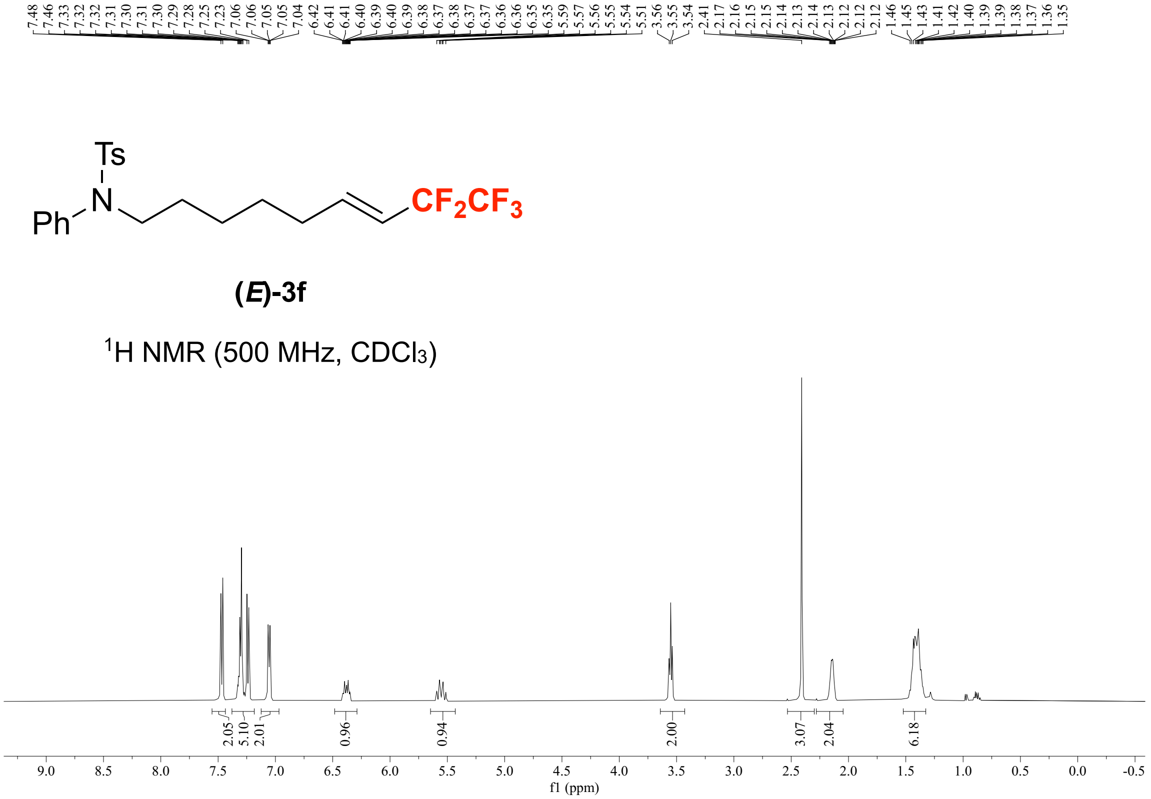
7.48 7.46 7.33 7.32 7.32 7.31 7.31 7.30 7.30 7.29 7.28 7.25 7.23 7.06 7.06 7.05 7.05 7.04 6.42 6.41 6.41 6.40 6.40 6.39 6.39 6.38 6.38 6.37 6.37 6.37 6.37 6.36 6.36 6.35 6.35 5.59 5.57 5.56 5.55 5.54 5.51 3.56 3.55 3.54 2.41 2.17 2.16 2.15 2.15 2.14 2.14 2.13 2.13 2.13 2.12 2.12 2.12 1.46 1.45 1.43 1.41 1.42 1.40 1.39 1.39 1.38 1.37 1.36 1.35
Ts
N
Ph
CF2CF3
(E)-3f
1H NMR (500 MHz, CDCl3)
2.05 5.10 2.01 0.96 0.94 2.00 3.07 2.04 6.18
9.0 8.5 8.0 7.5 7.0 6.5 6.0 5.5 5.0 4.5 4.0 3.5 3.0 2.5 2.0 1.5 1.0 0.5 0.0 -0.5
f1 (ppm)

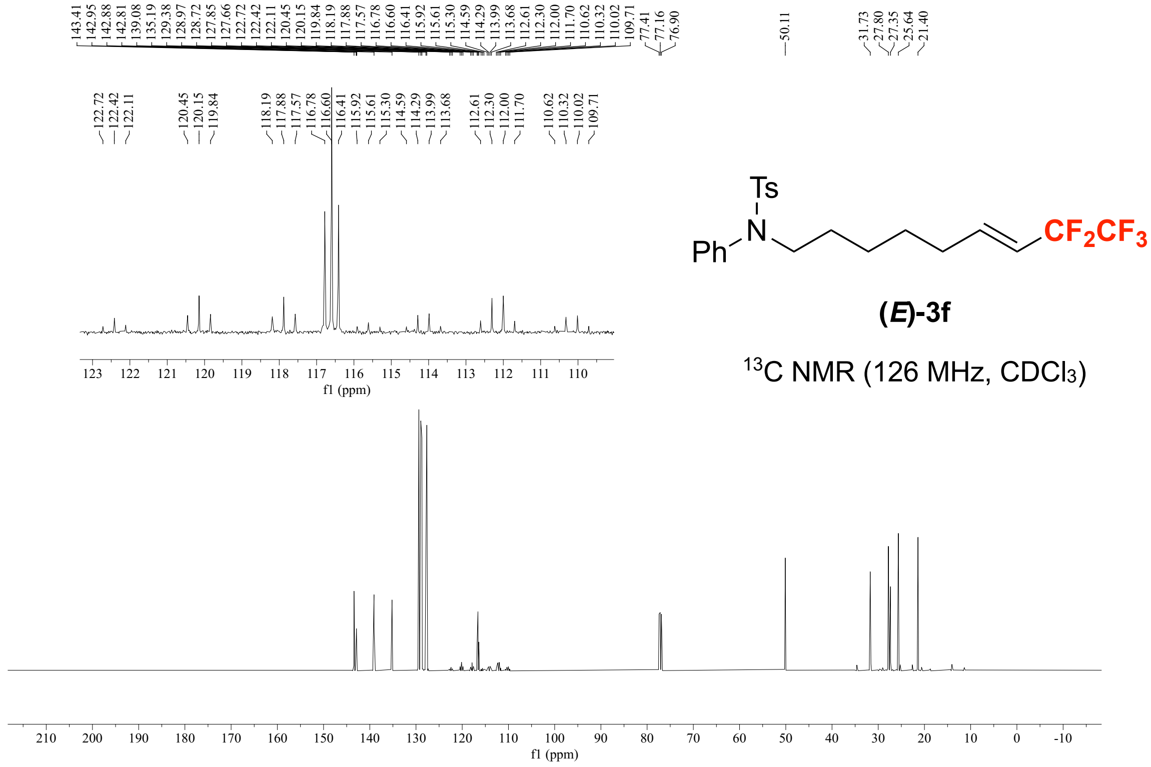
143.41 142.95 142.88 142.81 139.08 135.19 129.38 128.97 128.72 127.85 127.66 122.72 122.42 122.11 120.45 120.15 119.84 118.19 117.88 117.57 116.78 116.60 116.41 115.92 115.61 115.30 114.59 114.29 113.99 113.68 112.61 112.30 112.00 111.70 110.62 110.32 110.02 109.71 77.41 77.16 76.90
50.11
31.73 27.80 27.35 25.64 21.40
122.72 122.42 122.11 120.45 120.15 119.84 118.19 117.88 117.57 116.78 116.60 116.41 115.92 115.61 115.30 114.59 114.29 113.99 113.68 112.61 112.30 112.00 111.70 110.62 110.32 110.02 109.71
123 122 121 120 119 118 117 116 115 114 113 112 111 110
f1 (ppm)
Ts
N
Ph
CF2CF3
(E)-3f
13C NMR (126 MHz, CDCl3)
210 200 190 180 170 160 150 140 130 120 110 100 90 80 70 60 50 40 30 20 10 0 -10
f1 (ppm)

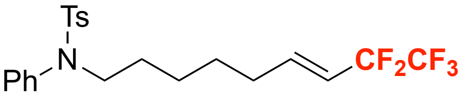

(*E*)-3f

^{19}F NMR (471 MHz, $CDCl_3$)

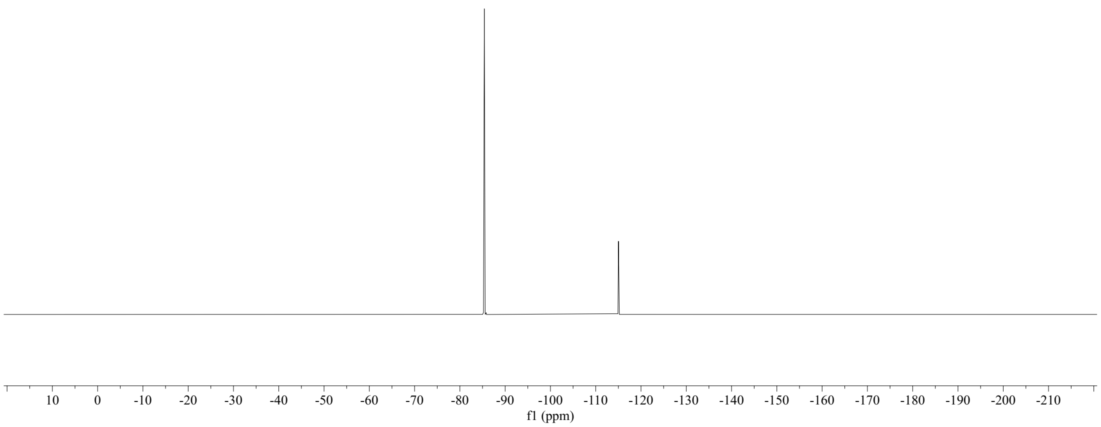

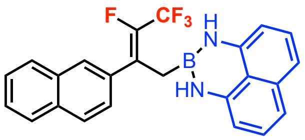

2a (*Z*/*E* > 99:1)

^{1}H NMR (500 MHz, $CDCl_3$)

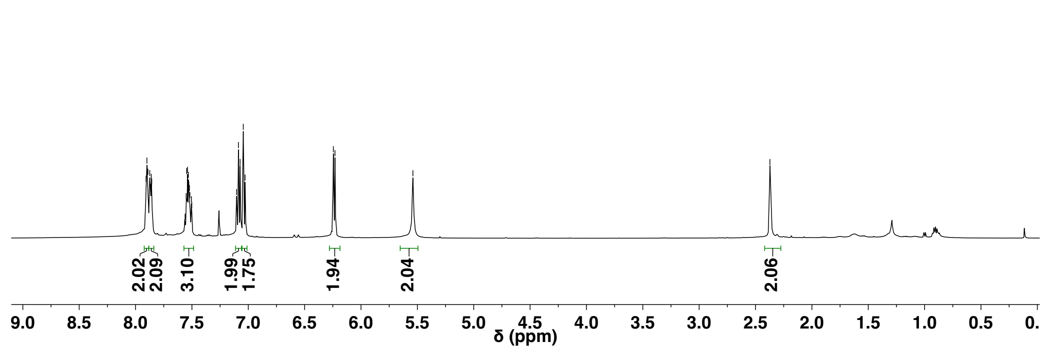

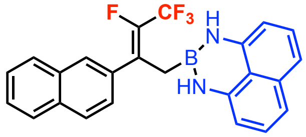

2a (*Z*/*E* > 99:1)

^{19}F NMR (471 MHz, $CDCl_3$)

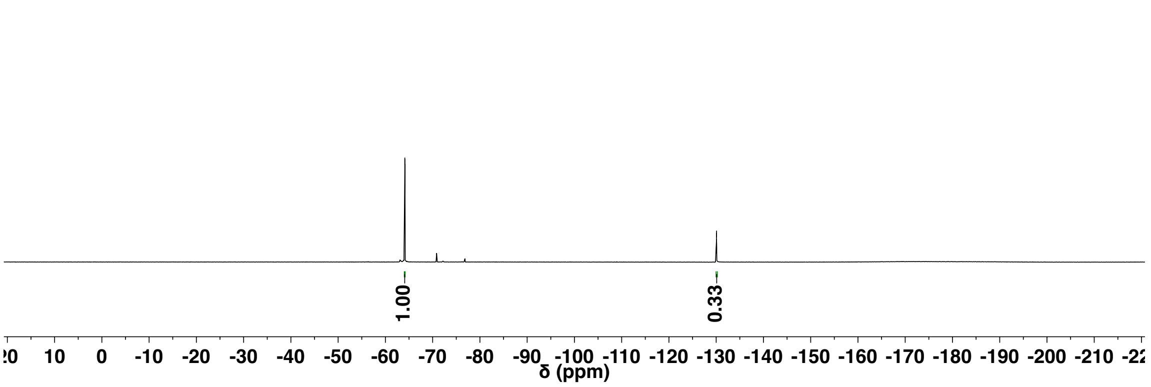

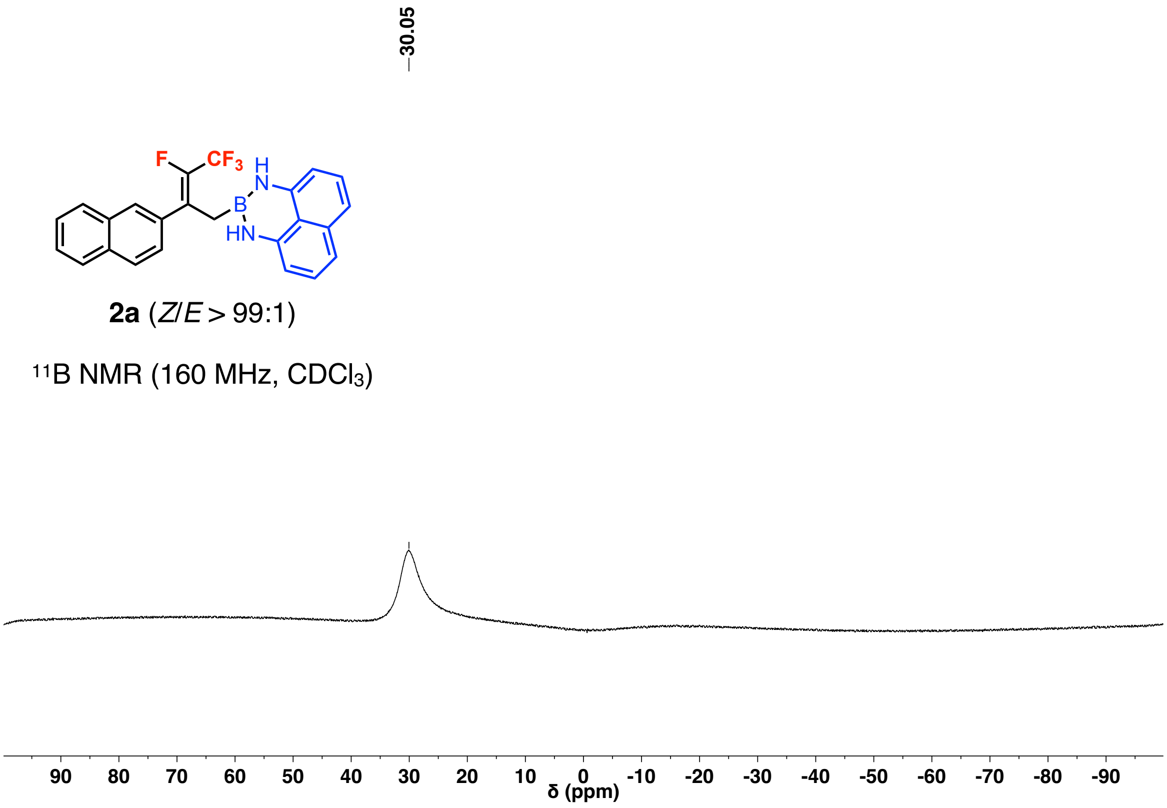
30.05
F
CF3
H
N
B
HN
2a (Z/E > 99:1)
11B NMR (160 MHz, CDCl3)
90 80 70 60 50 40 30 20 10 0 -10 -20 -30 -40 -50 -60 -70 -80 -90
δ (ppm)

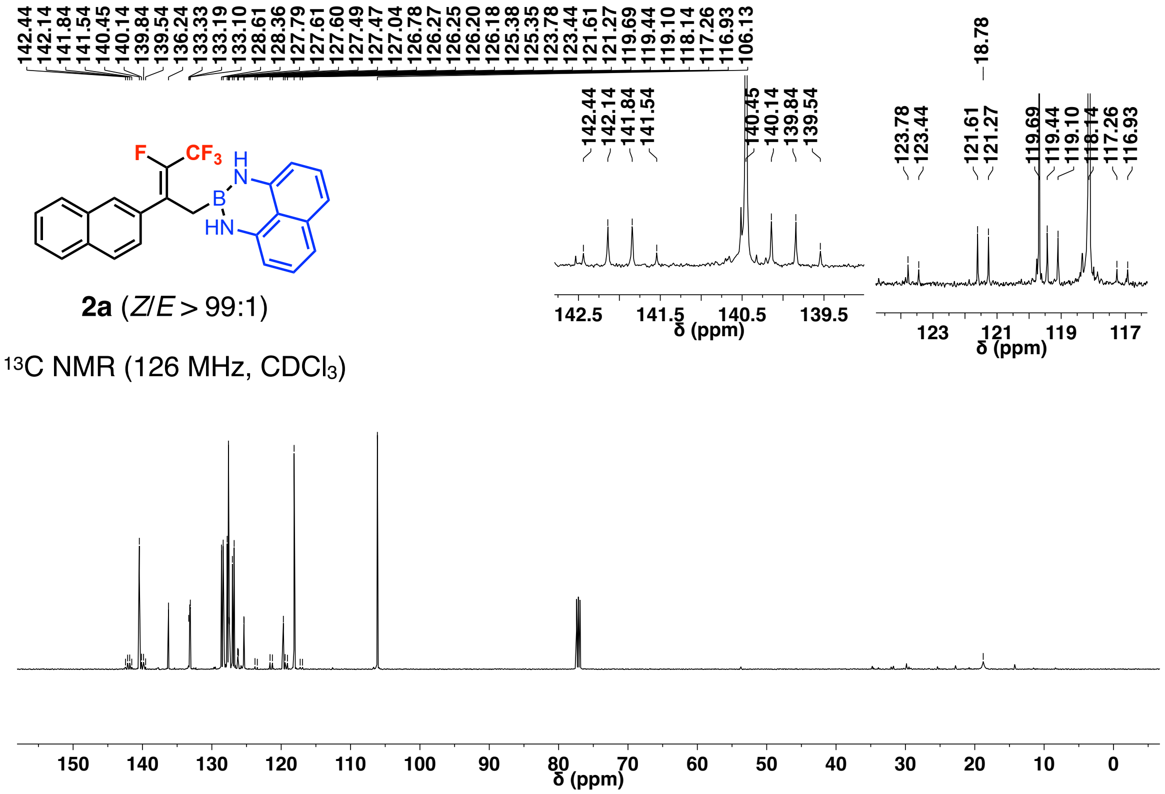
142.44 142.14 141.84 141.54 140.45 140.14 139.84 139.54 136.24 133.33 133.19 133.10 128.61 128.36 127.79 127.61 127.60 127.49 127.47 127.04 126.78 126.27 126.25 126.20 126.18 125.38 125.35 123.78 123.44 121.61 121.27 119.69 119.44 119.10 118.14 117.26 116.93 106.13
18.78
F
CF3
H
N
B
HN
2a (Z/E > 99:1)
13C NMR (126 MHz, CDCl3)
142.44 142.14 141.84 141.54
140.45 140.14 139.84 139.54
142.5 141.5 140.5 139.5
δ (ppm)
123.78 123.44 121.61 121.27 119.69 119.44 119.10 118.14 117.26 116.93
123 121 119 117
δ (ppm)
150 140 130 120 110 100 90 80 70 60 50 40 30 20 10 0
δ (ppm)

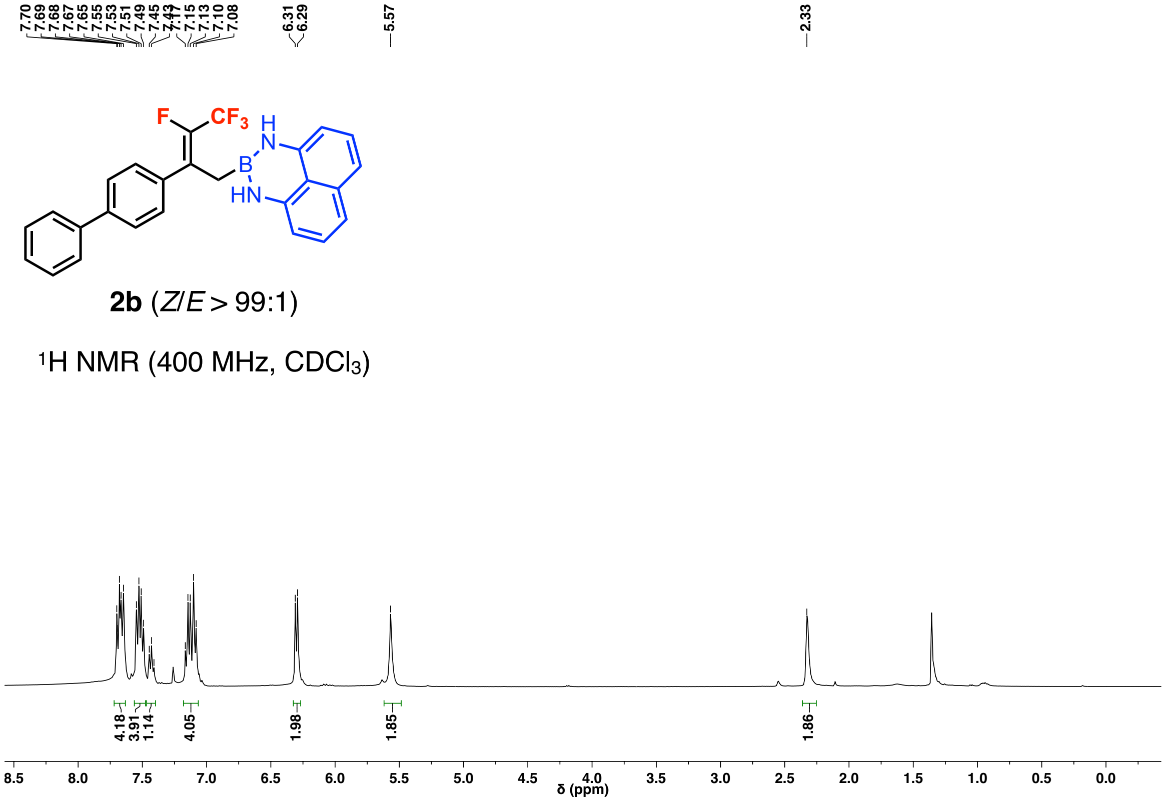
7.70
7.69
7.68
7.67
7.65
7.55
7.53
7.51
7.49
7.45
7.43
7.17
7.15
7.13
7.10
7.08
6.31
6.29
5.57
2.33
F
CF3
H
N
B
HN
2b (Z/E > 99:1)
1H NMR (400 MHz, CDCl3)
4.18
3.91
1.14
4.05
1.98
1.85
1.86
8.5 8.0 7.5 7.0 6.5 6.0 5.5 5.0 4.5 4.0 3.5 3.0 2.5 2.0 1.5 1.0 0.5 0.0
δ (ppm)

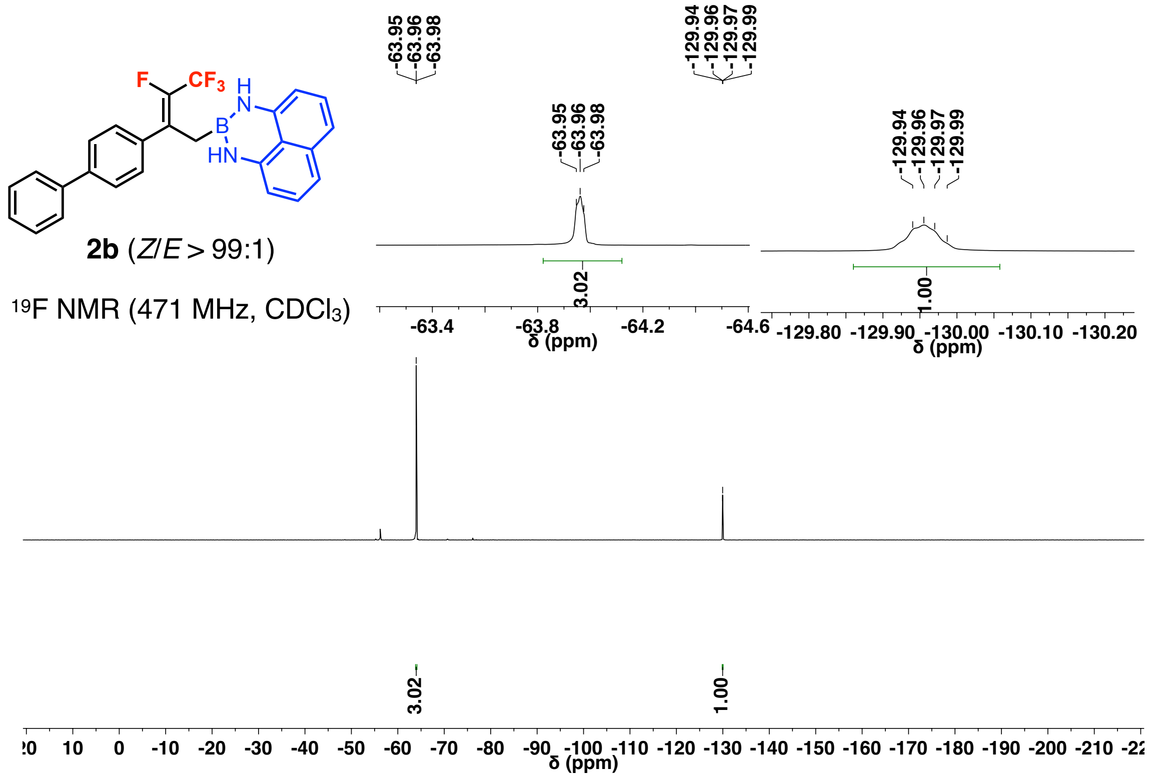
-63.95
-63.96
-63.98
-129.94
-129.96
-129.97
-129.99
F
CF3
H
N
B
HN
2b (Z/E > 99:1)
19F NMR (471 MHz, CDCl3)
3.02
-63.4 -63.8 -64.2 -64.6
δ (ppm)
1.00
-129.80 -129.90 -130.00 -130.10 -130.20
δ (ppm)
3.02
1.00
10 0 -10 -20 -30 -40 -50 -60 -70 -80 -90 -100 -110 -120 -130 -140 -150 -160 -170 -180 -190 -200 -210
δ (ppm)

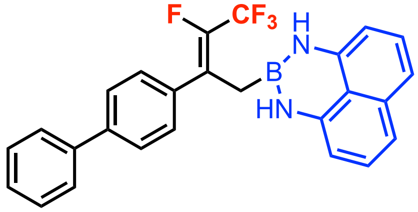

2b (*Z*/*E* > 99:1)

^{11}B NMR (160 MHz, $CDCl_3$)

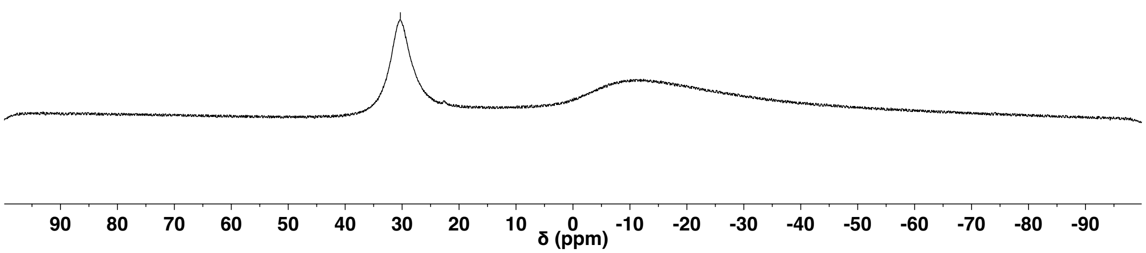

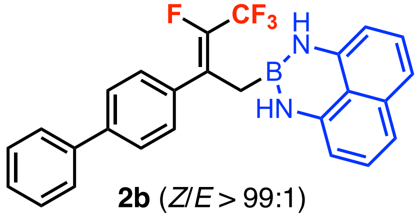

2b (*Z*/*E* > 99:1)

^{13}C NMR (126 MHz, $CDCl_3$)

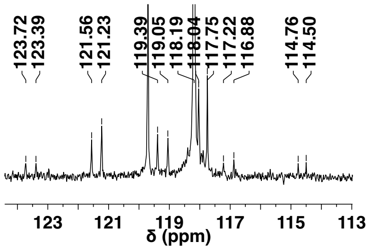

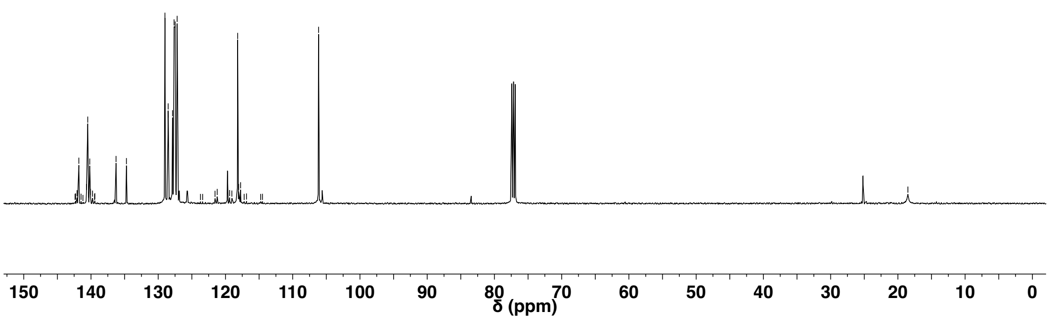

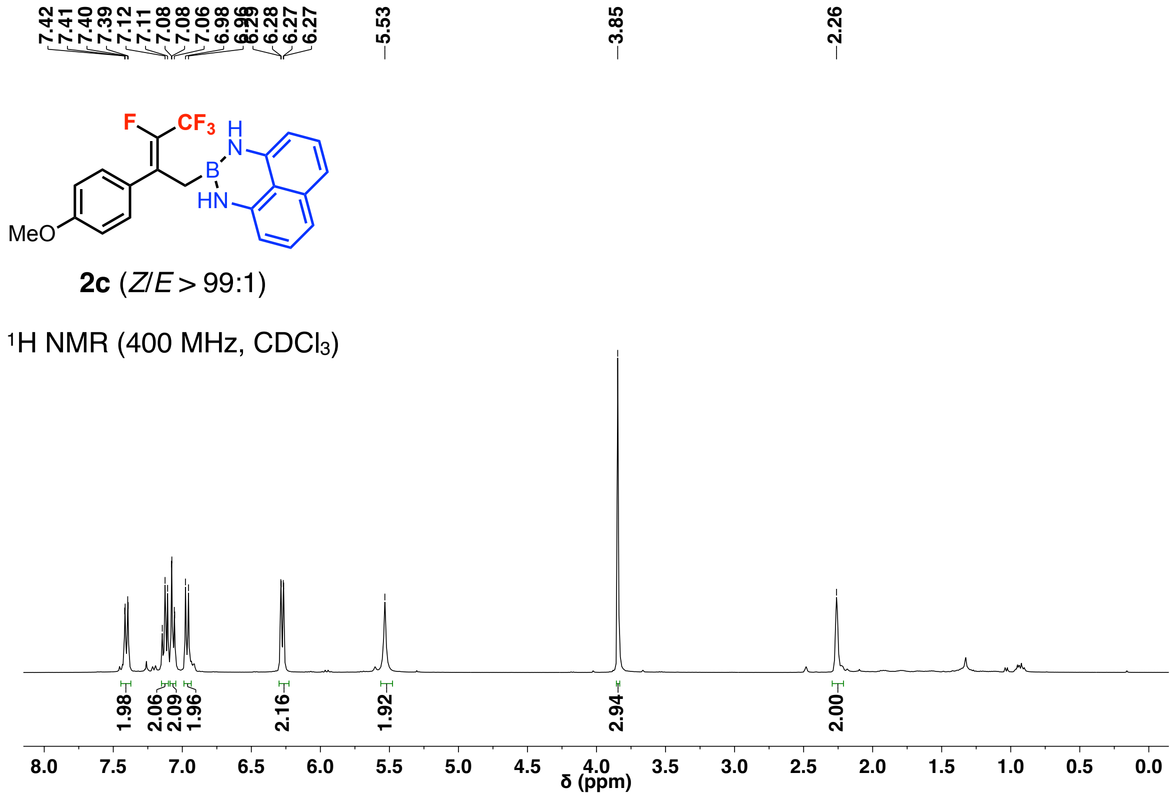
7.42
7.41
7.40
7.39
7.12
7.11
7.08
7.08
7.06
6.98
6.96
6.29
6.28
6.27
6.27
5.53
3.85
2.26
F
CF3
H
N
B
HN
MeO
2c (Z/E > 99:1)
1H NMR (400 MHz, CDCl3)
1.98
2.06
2.09
1.96
2.16
1.92
2.94
2.00
8.0 7.5 7.0 6.5 6.0 5.5 5.0 4.5 4.0 3.5 3.0 2.5 2.0 1.5 1.0 0.5 0.0
δ (ppm)

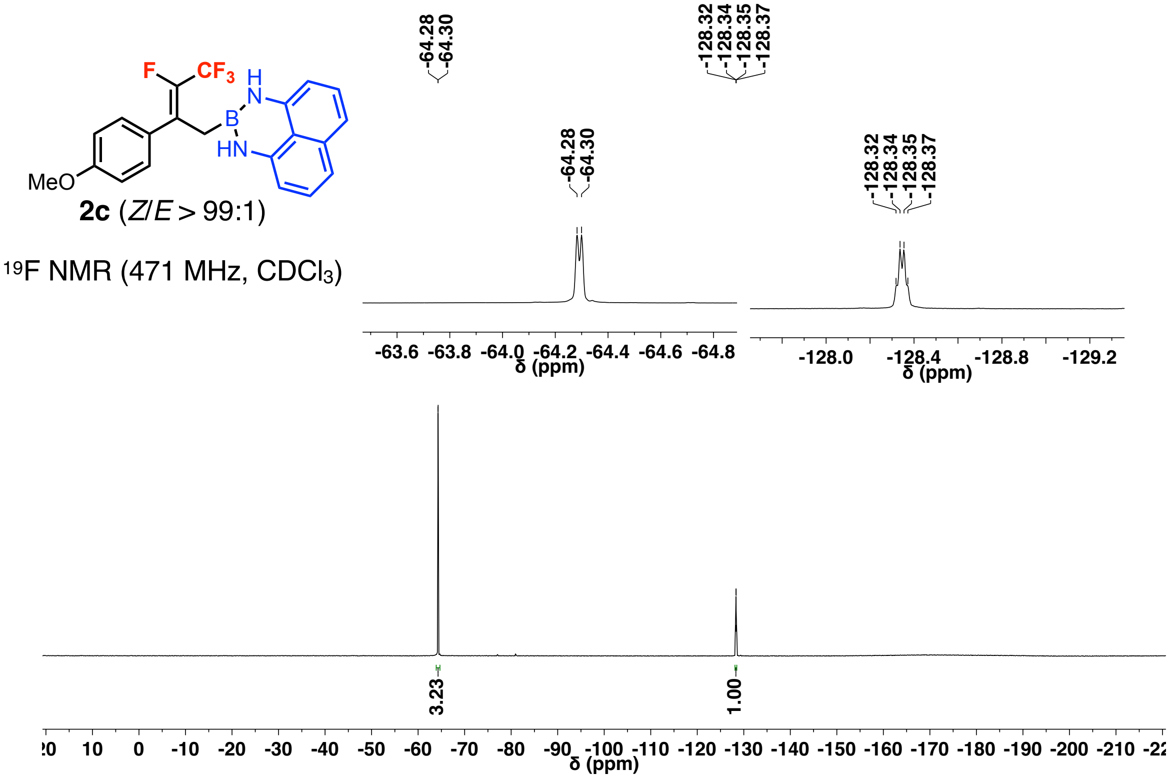
-64.28
-64.30
-128.32
-128.34
-128.35
-128.37
F
CF3
H
N
B
HN
MeO
2c (Z/E > 99:1)
19F NMR (471 MHz, CDCl3)
-63.6 -63.8 -64.0 -64.2 -64.4 -64.6 -64.8
δ (ppm)
-128.0 -128.4 -128.8 -129.2
δ (ppm)
3.23
1.00
10 0 -10 -20 -30 -40 -50 -60 -70 -80 -90 -100 -110 -120 -130 -140 -150 -160 -170 -180 -190 -200 -210
δ (ppm)

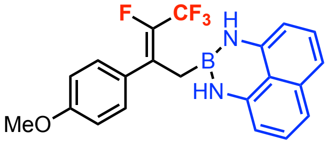

2c (*Z*/*E* > 99:1)

^{11}B NMR (160 MHz, $CDCl_3$)

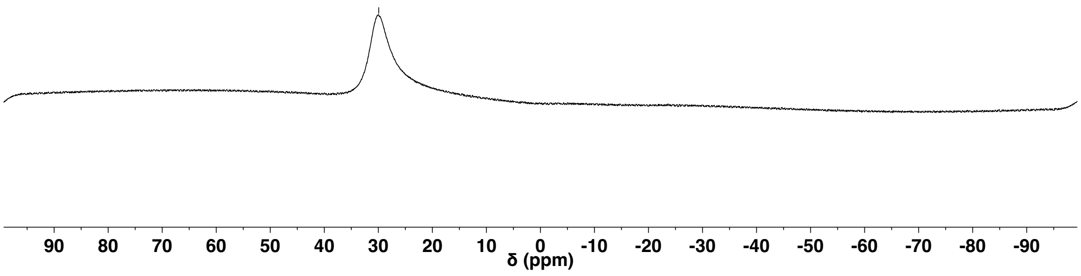

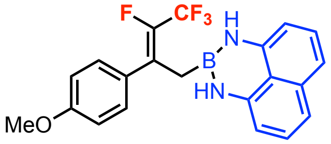

2c (*Z*/*E* > 99:1)

^{13}C NMR (101 MHz, $CDCl_3$)

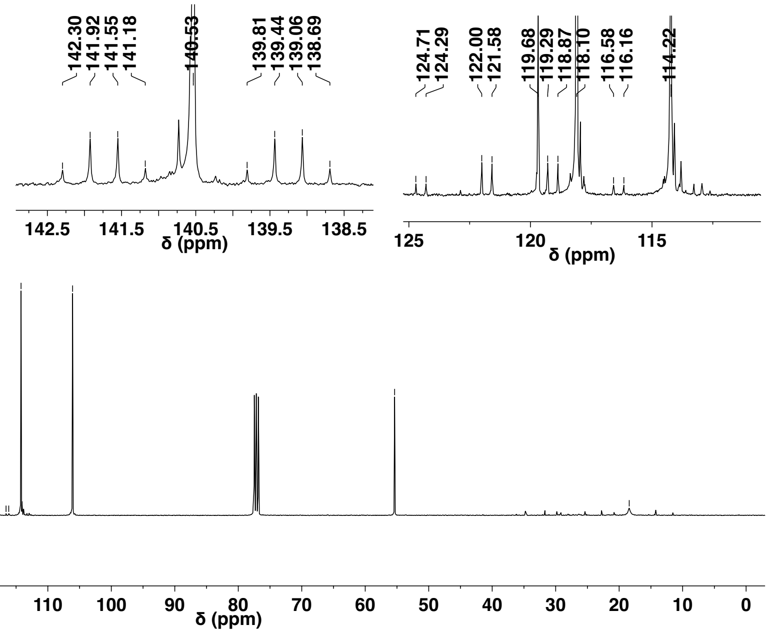

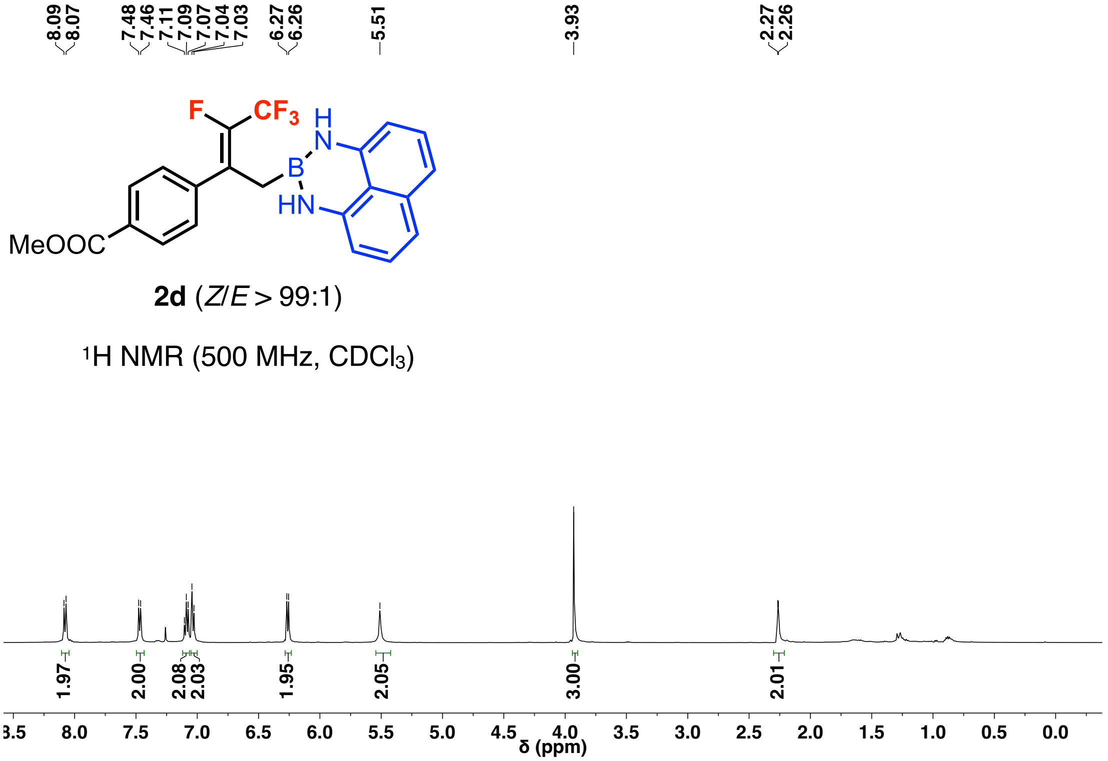
8.09
8.07
7.48
7.46
7.11
7.09
7.07
7.04
7.03
6.27
6.26
5.51
3.93
2.27
2.26
F
CF_3
H
N
B
HN
MeOOC
2d (*Z*/*E* > 99:1)
^{1}H NMR (500 MHz, $CDCl_3$)
1.97
2.00
2.08
2.03
1.95
2.05
3.00
2.01
δ (ppm)

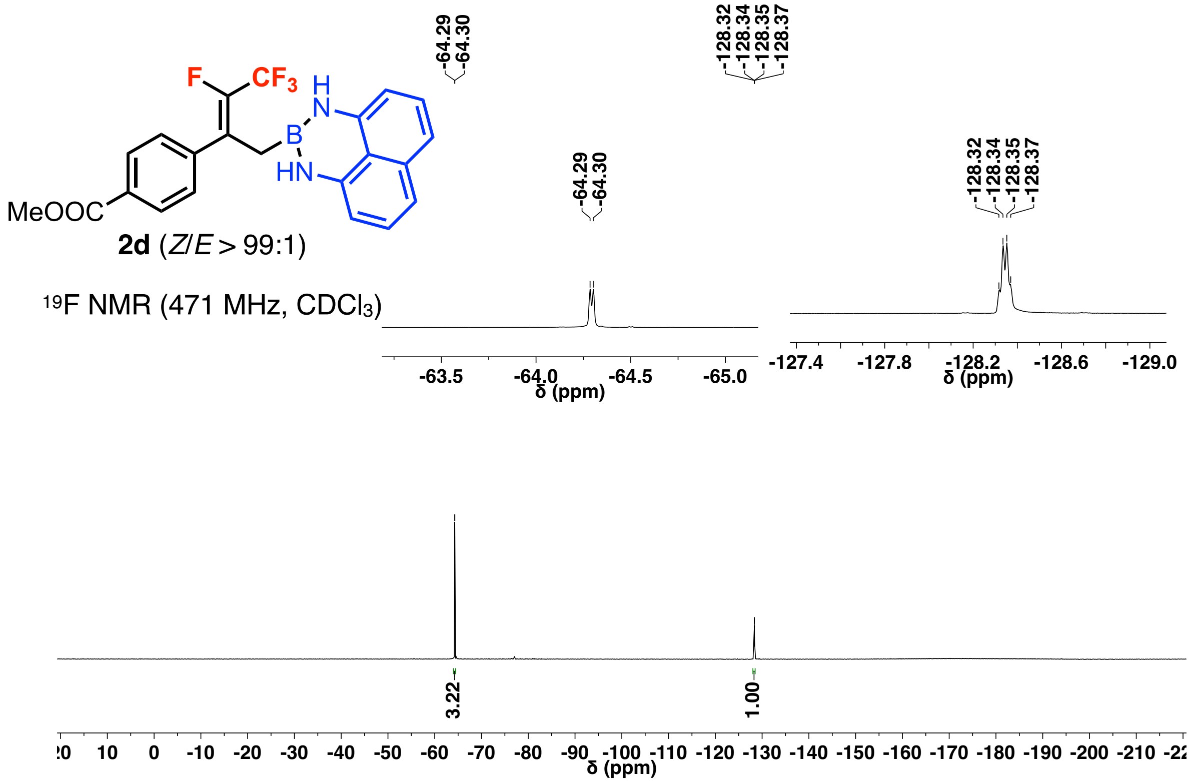
-64.29
-64.30
-128.32
-128.34
-128.35
-128.37
F
CF_3
H
N
B
HN
MeOOC
2d (*Z*/*E* > 99:1)
^{19}F NMR (471 MHz, $CDCl_3$)
3.22
1.00
δ (ppm)

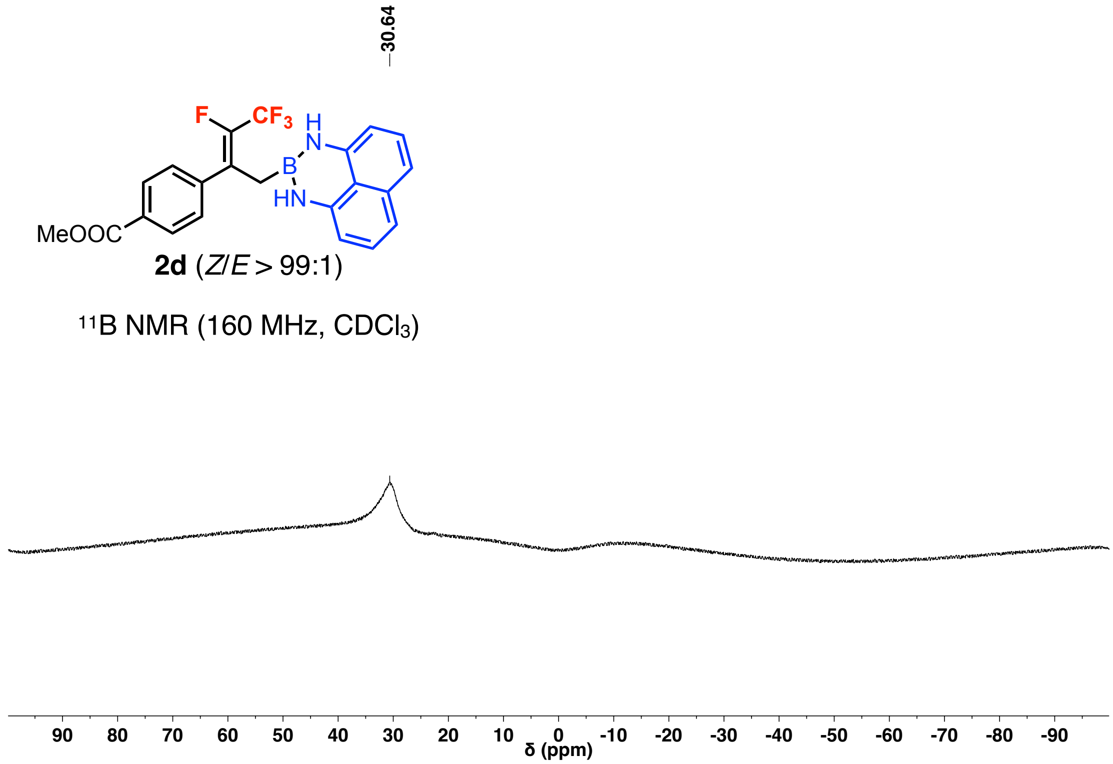

30.64
F
CF_3
H
N
B
HN
MeOOC
2d (Z/E > 99:1)
^{11}B NMR (160 MHz, $CDCl_3$)
90 80 70 60 50 40 30 20 10 0 -10 -20 -30 -40 -50 -60 -70 -80 -90
δ (ppm)

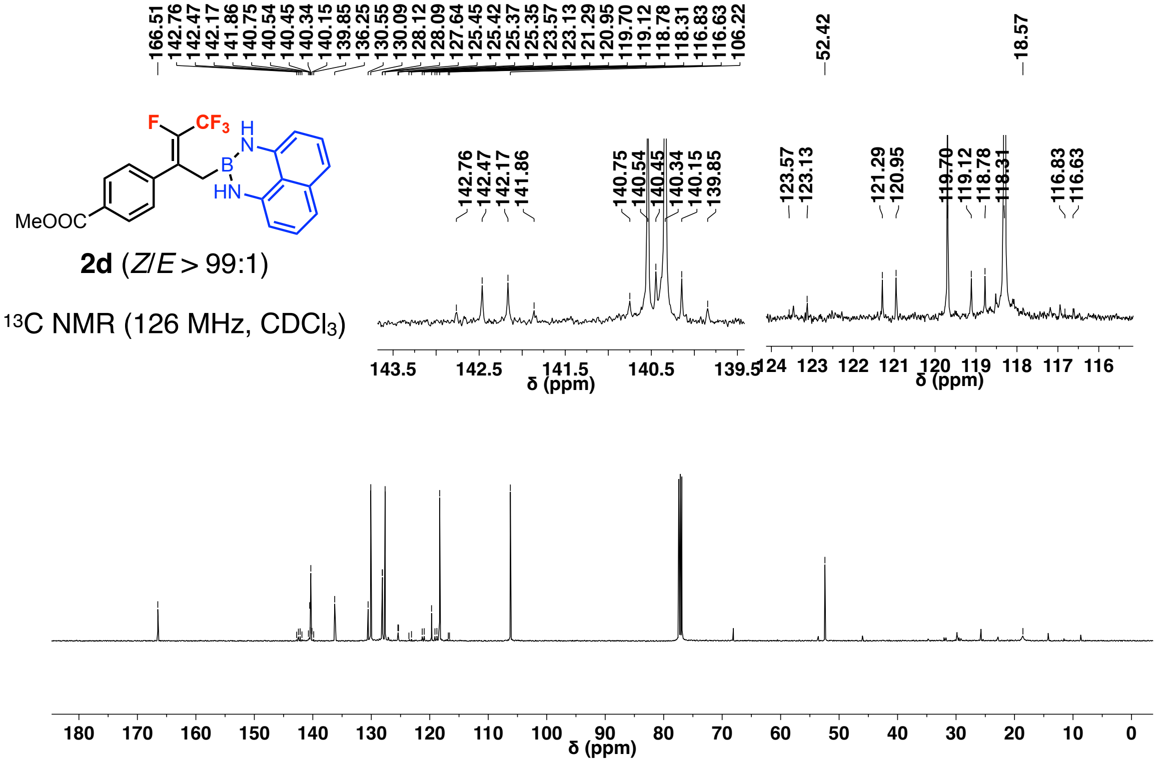

166.51 142.76 142.47 142.17 141.86 140.75 140.54 140.45 140.34 140.15 139.85 136.25 130.55 130.09 128.12 128.09 127.64 125.45 125.42 125.37 125.35 123.57 123.13 121.29 120.95 119.70 119.12 118.78 118.31 116.83 116.63 106.22
52.42
18.57
F
CF_3
H
N
B
HN
MeOOC
2d (Z/E > 99:1)
^{13}C NMR (126 MHz, $CDCl_3$)
142.76 142.47 142.17 141.86 140.75 140.54 140.45 140.34 140.15 139.85
143.5 142.5 141.5 140.5 139.5
δ (ppm)
123.57 123.13 121.29 120.95 119.70 119.12 118.78 118.31 116.83 116.63
124 123 122 121 120 119 118 117 116
δ (ppm)
180 170 160 150 140 130 120 110 100 90 80 70 60 50 40 30 20 10 0
δ (ppm)

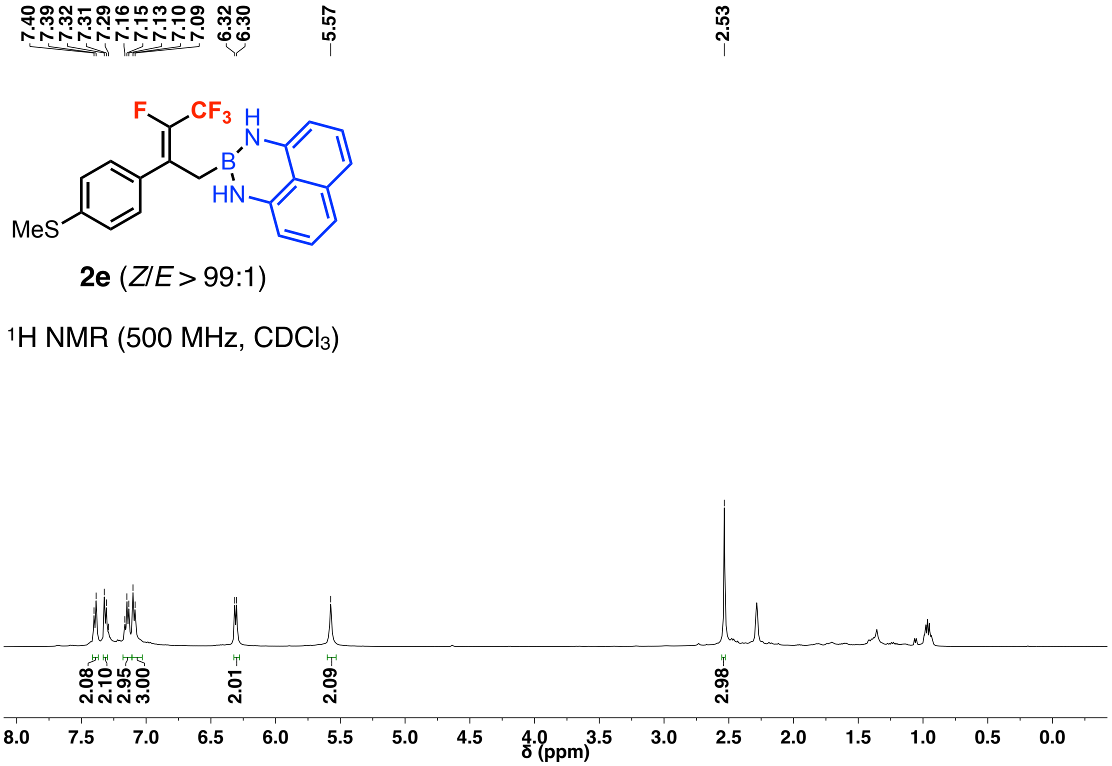
2e (Z/E > 99:1)
1H NMR (500 MHz, CDCl3)
δ (ppm)

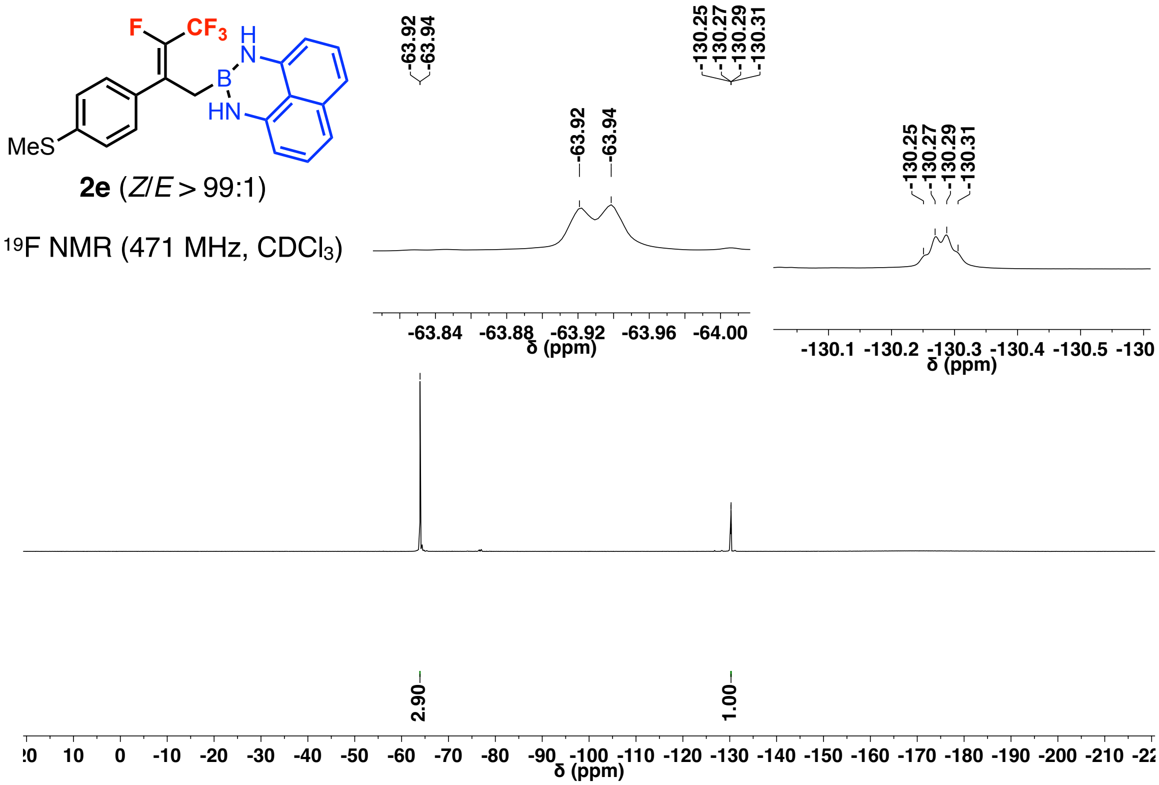
2e (Z/E > 99:1)
19F NMR (471 MHz, CDCl3)
δ (ppm)

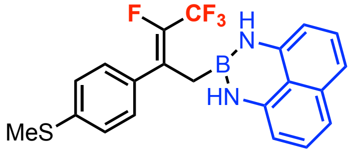

2e (*Z*/*E* > 99:1)

^{11}B NMR (160 MHz, $CDCl_3$)

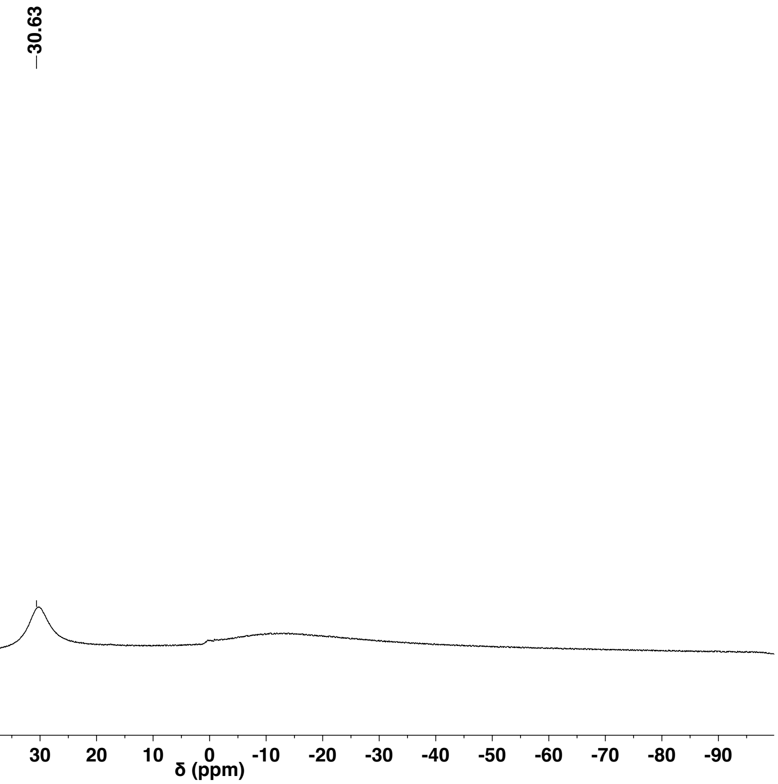

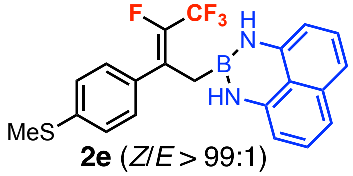

2e (*Z*/*E* > 99:1)

^{13}C NMR (126 MHz, $CDCl_3$)

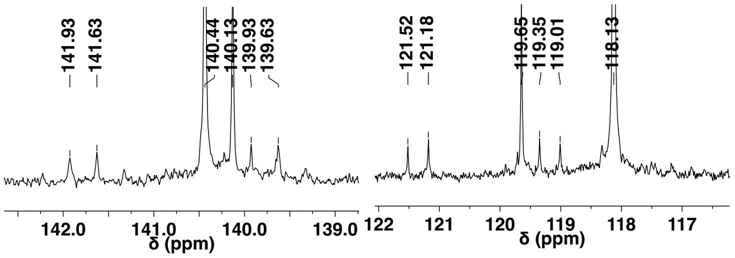

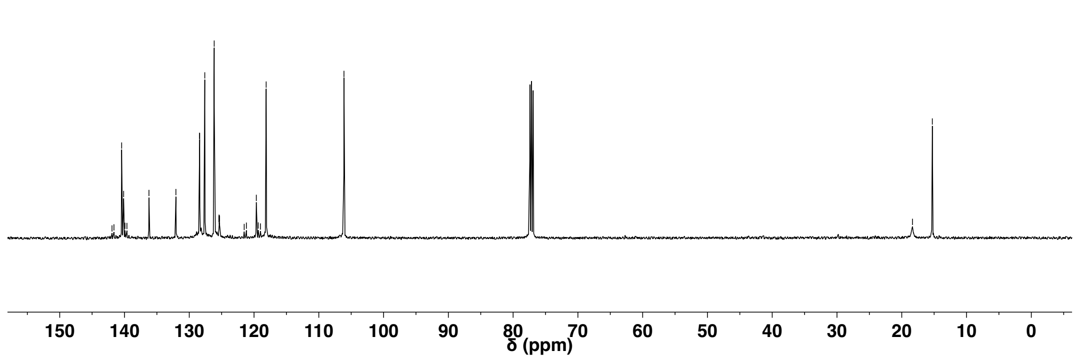

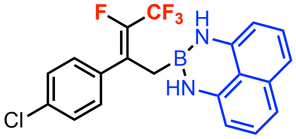

2f (*Z*/*E* > 99:1)

^{1}H NMR (400 MHz, $CDCl_3$)

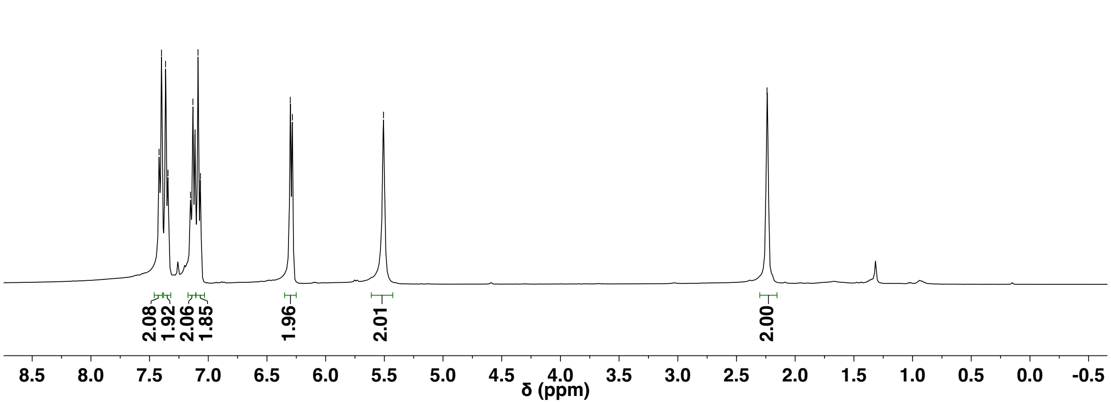

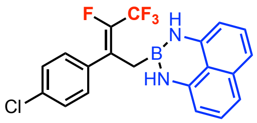

2f (*Z*/*E* > 99:1)

^{19}F NMR (471 MHz, $CDCl_3$)

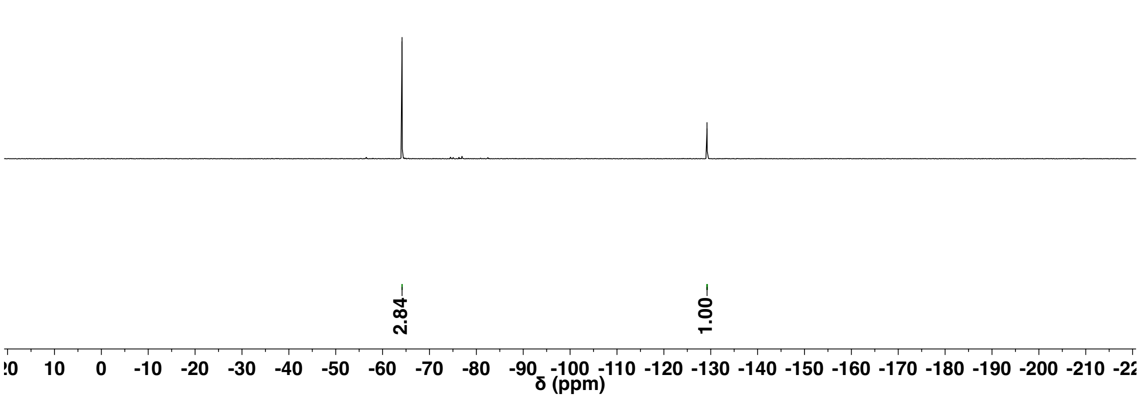

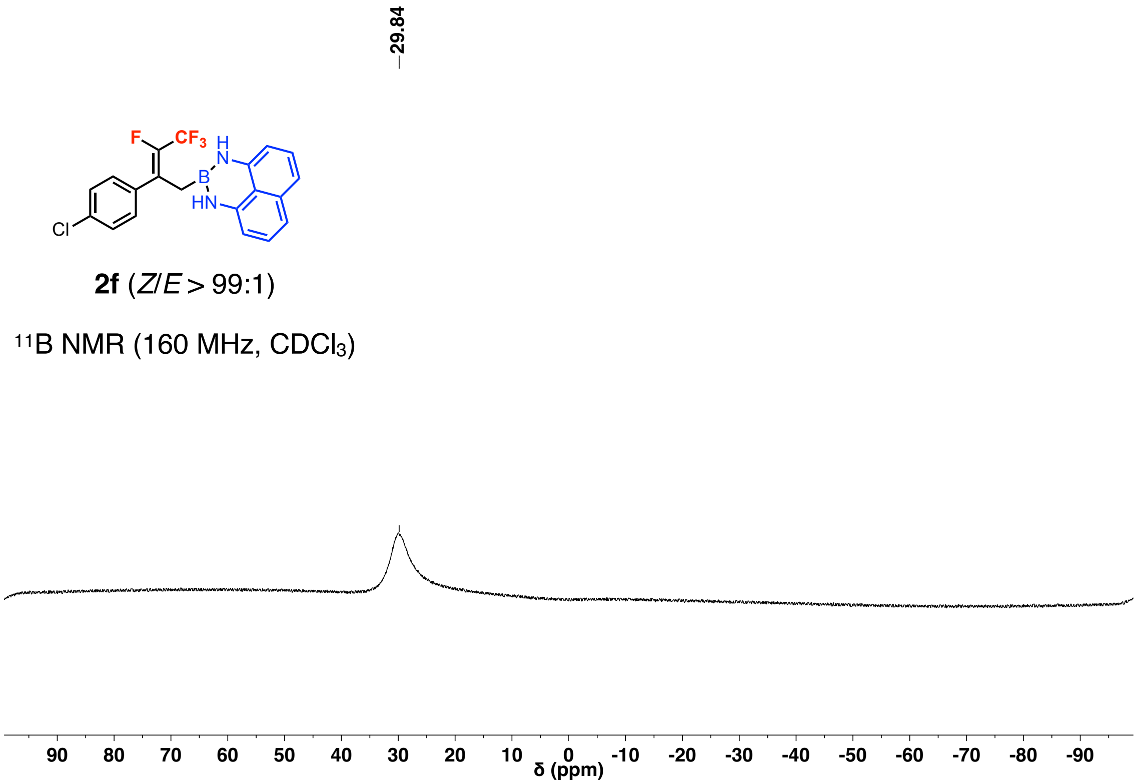

29.84
F
CF3
H
N
B
HN
Cl
2f (Z/E > 99:1)
11B NMR (160 MHz, CDCl3)
90 80 70 60 50 40 30 20 10 0 -10 -20 -30 -40 -50 -60 -70 -80 -90
δ (ppm)

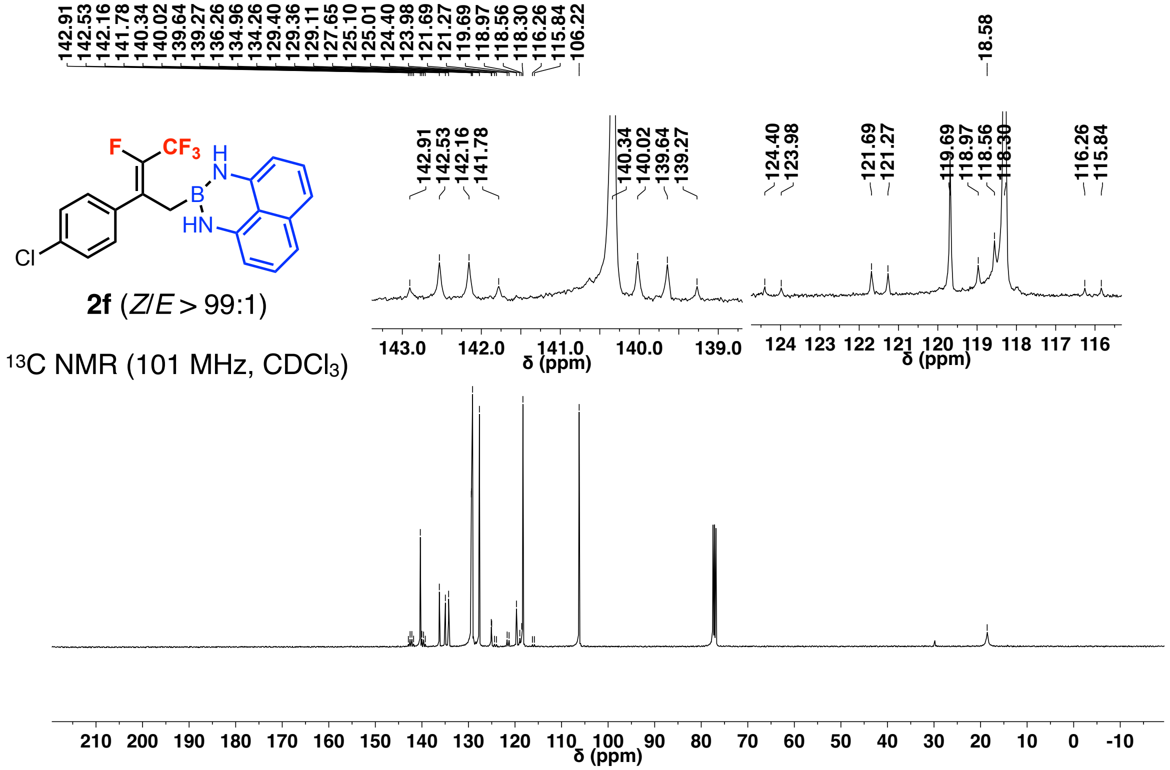

142.91 142.53 142.16 141.78 140.34 140.02 139.64 139.27 136.26 134.96 134.26 129.40 129.36 129.11 127.65 125.10 125.01 124.40 123.98 121.69 121.27 119.69 118.97 118.56 118.30 116.26 115.84 106.22
18.58
F
CF3
H
N
B
HN
Cl
2f (Z/E > 99:1)
13C NMR (101 MHz, CDCl3)
142.91 142.53 142.16 141.78
140.34 140.02 139.64 139.27
143.0 142.0 141.0 140.0 139.0
δ (ppm)
124.40 123.98 121.69 121.27 119.69 118.97 118.56 118.30 116.26 115.84
124 123 122 121 120 119 118 117 116
δ (ppm)
210 200 190 180 170 160 150 140 130 120 110 100 90 80 70 60 50 40 30 20 10 0 -10
δ (ppm)

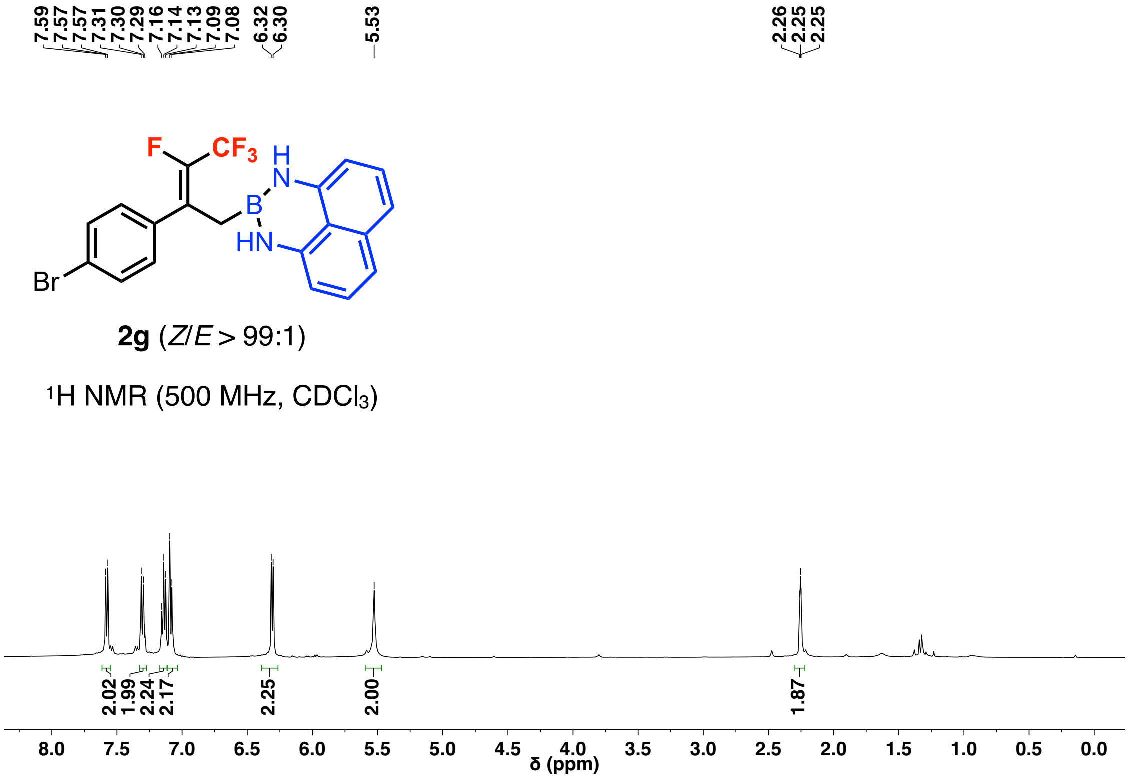

7.59 7.57 7.57 7.31 7.30 7.29 7.16 7.14 7.13 7.09 7.08 6.32 6.30
5.53
2.26 2.25 2.25
F
CF3
H
N
B
HN
Br
2g (Z/E > 99:1)
1H NMR (500 MHz, CDCl3)
2.02 1.99 2.24 2.17
2.25
2.00
1.87
8.0 7.5 7.0 6.5 6.0 5.5 5.0 4.5 4.0 3.5 3.0 2.5 2.0 1.5 1.0 0.5 0.0
δ (ppm)

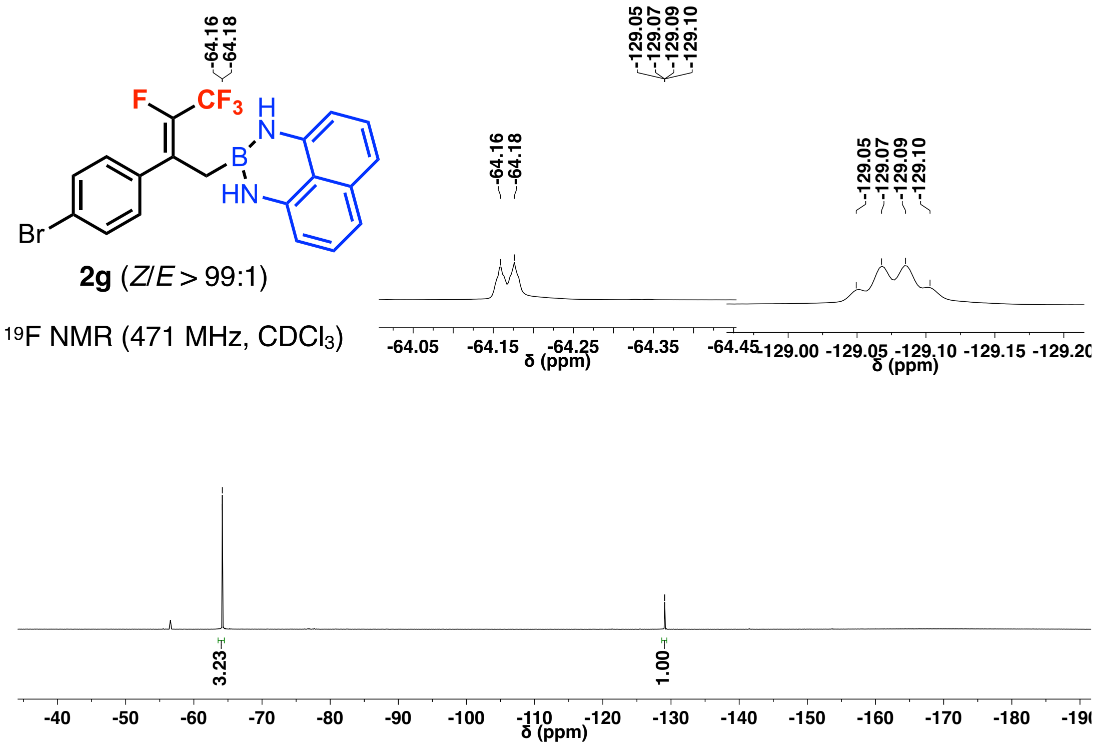

-64.16 -64.18
-129.05 -129.07 -129.09 -129.10
F
CF3
H
N
B
HN
Br
2g (Z/E > 99:1)
19F NMR (471 MHz, CDCl3)
-64.05 -64.15 -64.25 -64.35 -64.45
δ (ppm)
-129.00 -129.05 -129.10 -129.15 -129.20
δ (ppm)
3.23
1.00
-40 -50 -60 -70 -80 -90 -100 -110 -120 -130 -140 -150 -160 -170 -180 -190
δ (ppm)

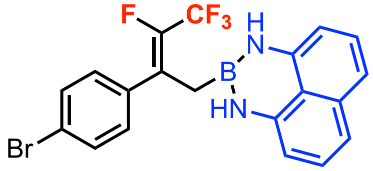

2g (*Z*/*E* > 99:1)

^{11}B NMR (160 MHz, CDCl$_3$)

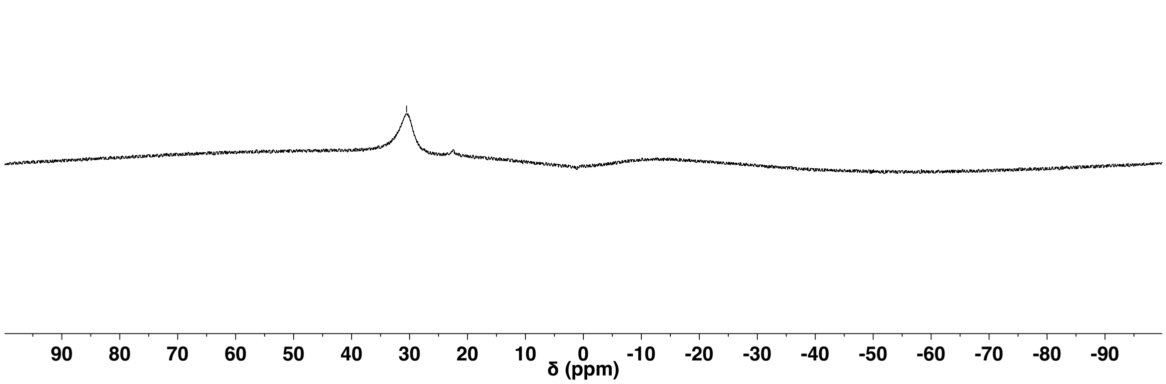

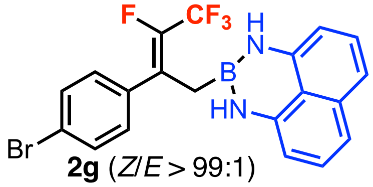

2g (*Z*/*E* > 99:1)

^{13}C NMR (126 MHz, CDCl$_3$)

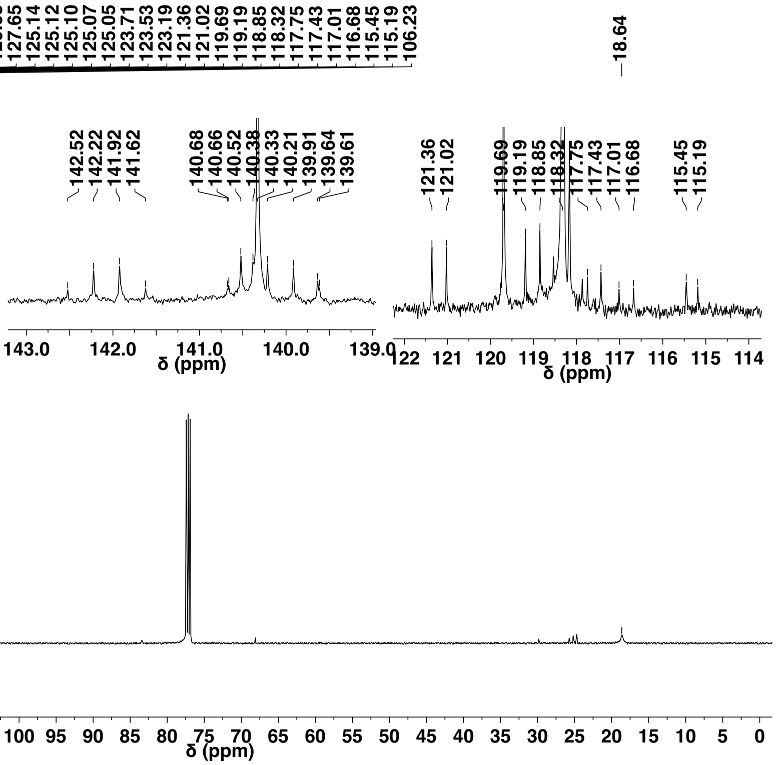

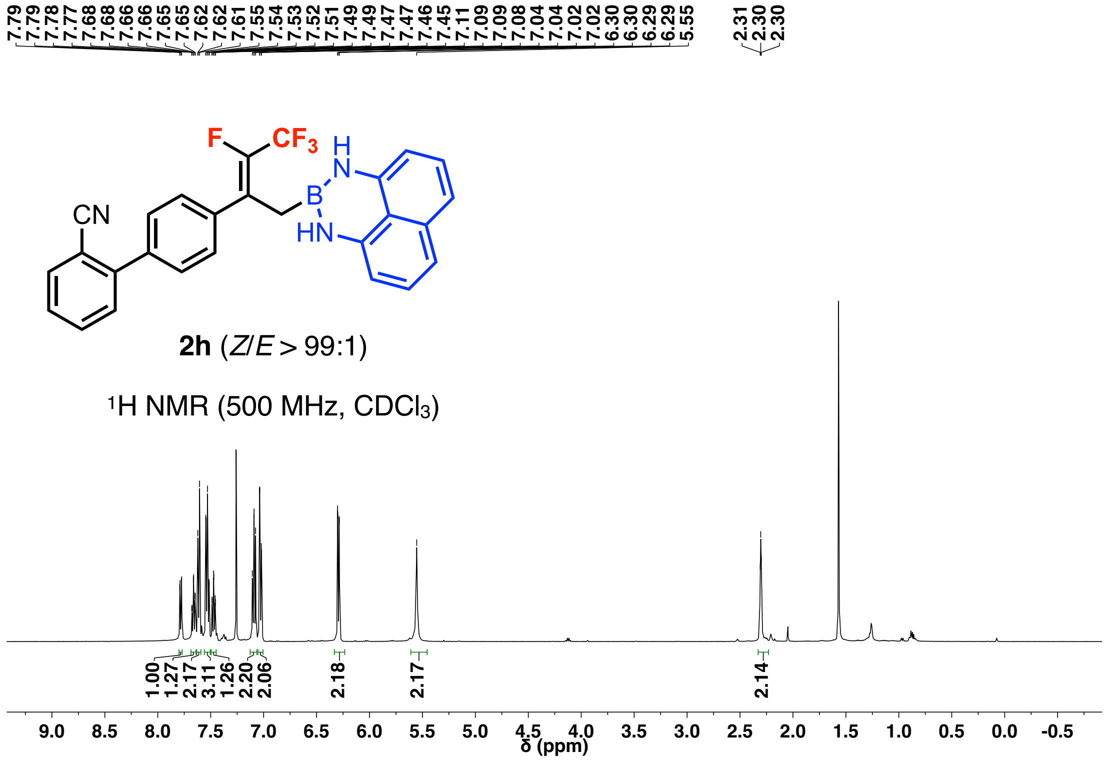
7.79
7.79
7.78
7.77
7.68
7.66
7.66
7.65
7.65
7.62
7.62
7.61
7.55
7.54
7.53
7.52
7.51
7.49
7.49
7.47
7.47
7.46
7.45
7.11
7.09
7.09
7.08
7.04
7.04
7.02
7.02
6.30
6.30
6.29
6.29
5.55
2.31
2.30
2.30
F
CF3
H
N
B
HN
CN
2h (Z/E > 99:1)
1H NMR (500 MHz, CDCl3)
1.00
1.27
2.17
3.11
1.26
2.20
2.06
2.18
2.17
2.14
9.0 8.5 8.0 7.5 7.0 6.5 6.0 5.5 5.0 4.5 4.0 3.5 3.0 2.5 2.0 1.5 1.0 0.5 0.0 -0.5
δ (ppm)

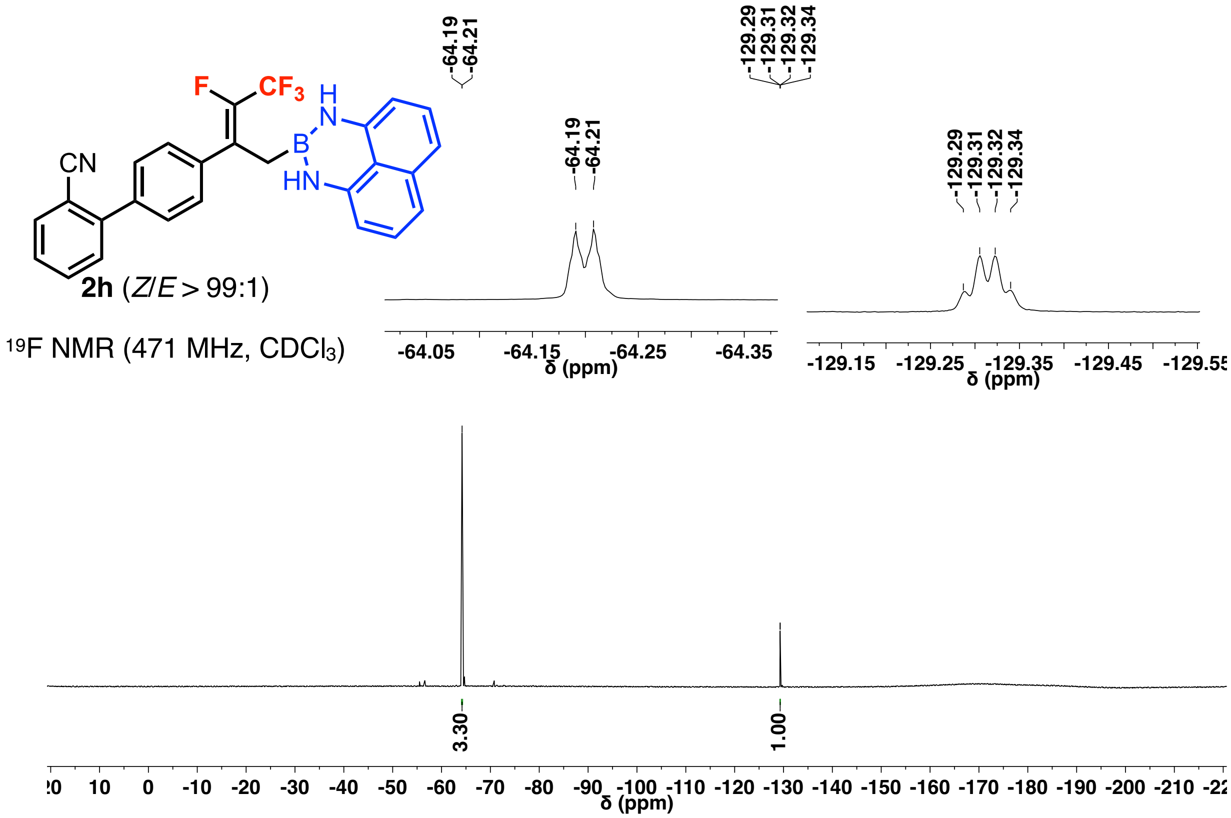
-64.19
-64.21
-129.29
-129.31
-129.32
-129.34
F
CF3
H
N
B
HN
CN
2h (Z/E > 99:1)
19F NMR (471 MHz, CDCl3)
-64.19
-64.21
-64.05 -64.15 -64.25 -64.35
δ (ppm)
-129.29
-129.31
-129.32
-129.34
-129.15 -129.25 -129.35 -129.45
δ (ppm)
3.30
1.00
10 0 -10 -20 -30 -40 -50 -60 -70 -80 -90 -100 -110 -120 -130 -140 -150 -160 -170 -180 -190 -200 -210
δ (ppm)

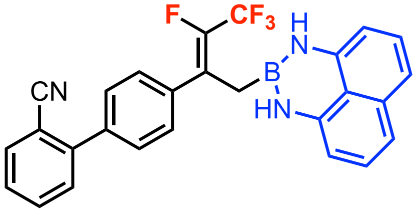

2h (*Z*/*E* > 99:1)

^{11}B NMR (160 MHz, $CDCl_3$)

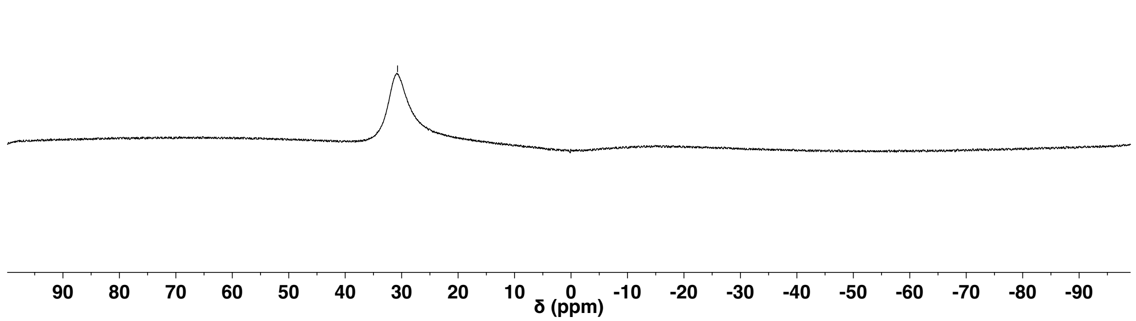

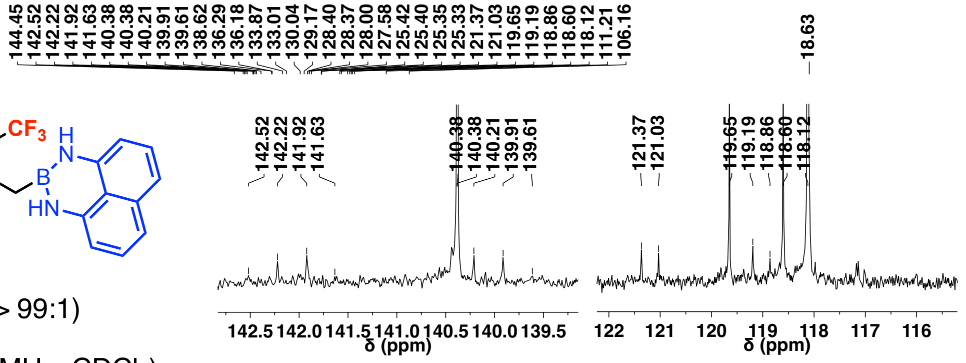

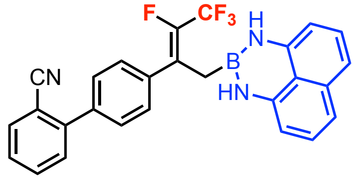

2h (*Z*/*E* > 99:1)

^{13}C NMR (126 MHz, $CDCl_3$)

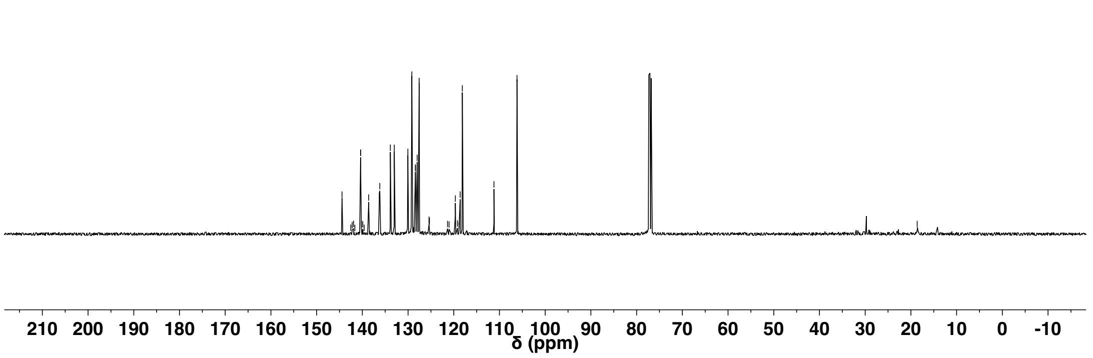

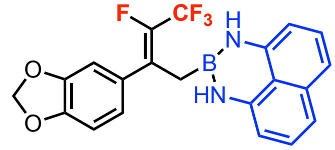

2i (*Z*/*E* > 99:1)

^{1}H NMR (500 MHz, CDCl$_3$)

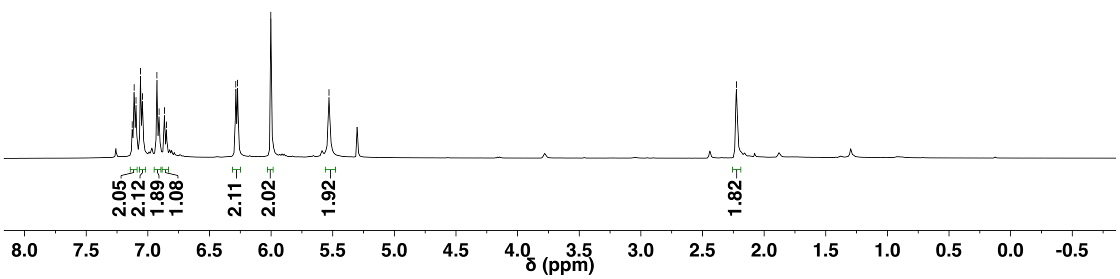

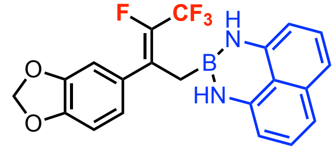

2i (*Z*/*E* > 99:1)

^{19}F NMR (471 MHz, CDCl$_3$)

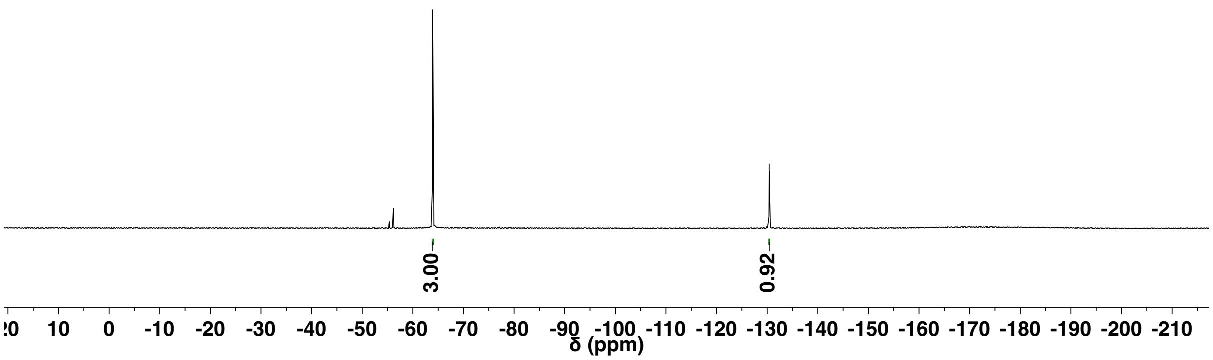

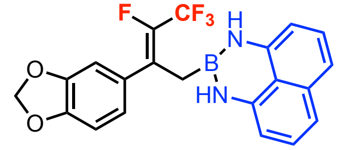

2i (*Z*/*E* > 99:1)

^{11}B NMR (160 MHz, CDCl$_3$)

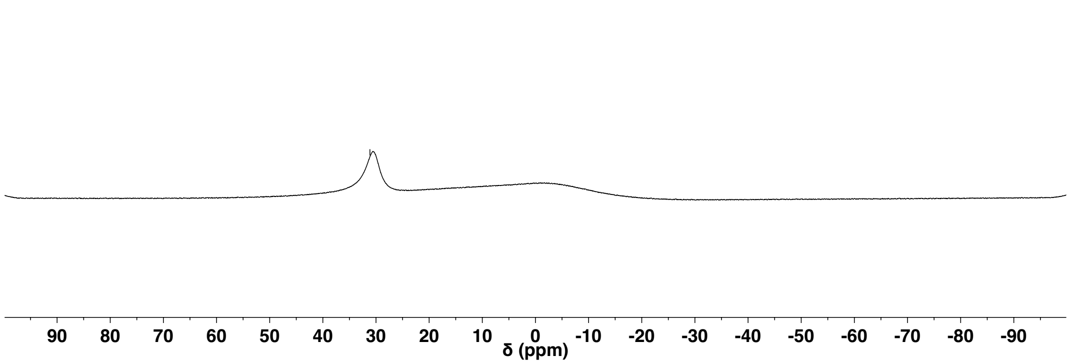

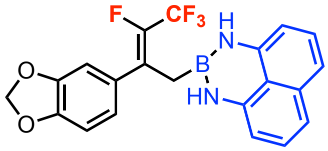

2i (*Z*/*E* > 99:1)

^{13}C NMR (126 MHz, CDCl$_3$)

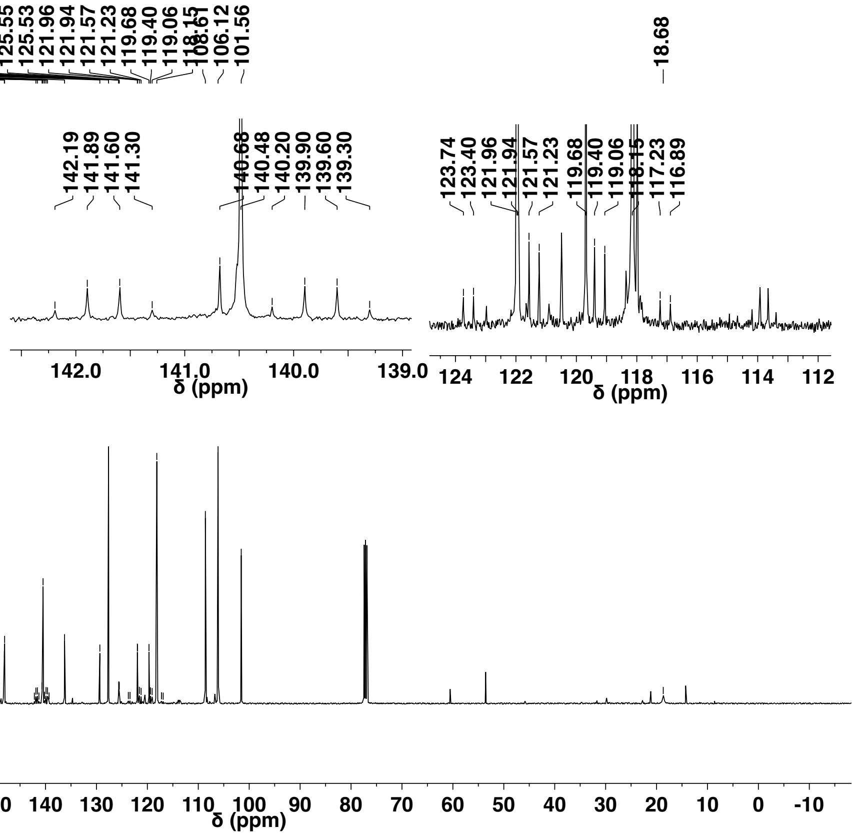

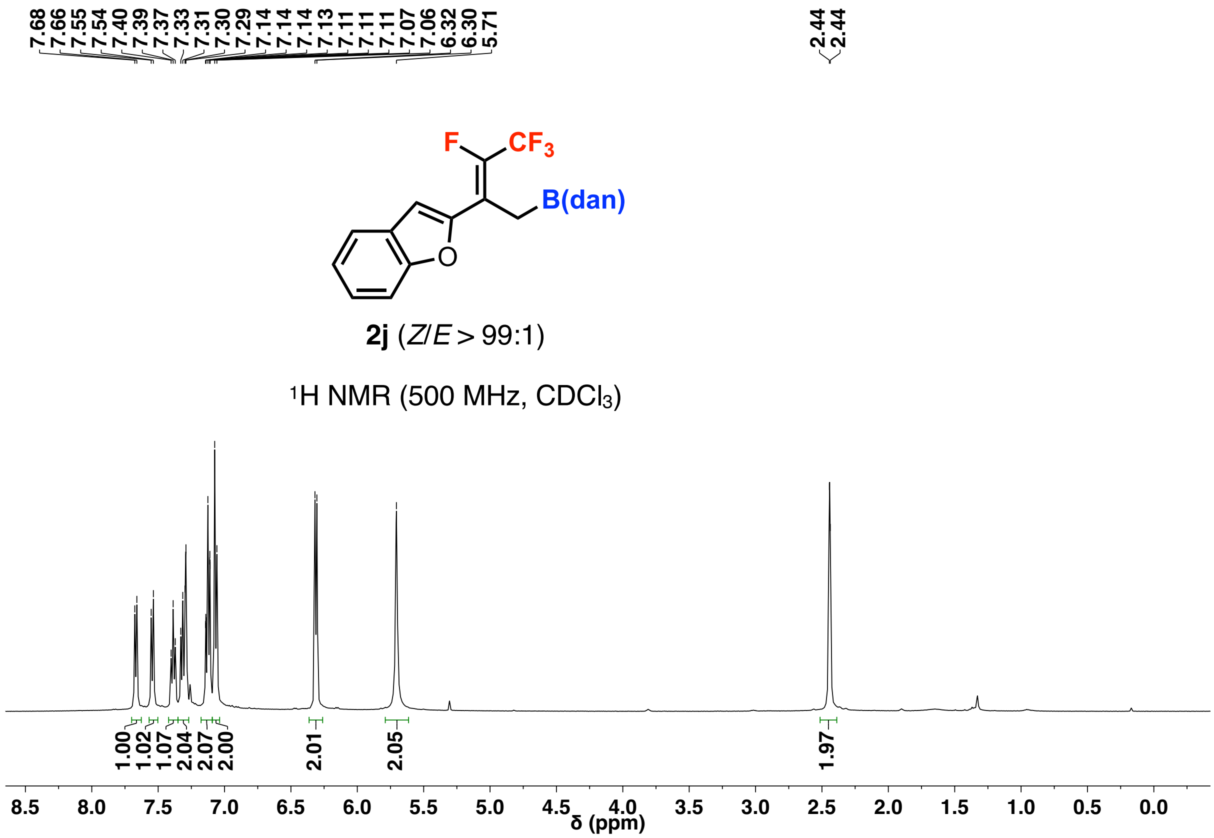

7.68
7.66
7.55
7.54
7.40
7.39
7.37
7.33
7.31
7.30
7.29
7.14
7.14
7.14
7.13
7.11
7.11
7.11
7.07
7.06
6.32
6.30
5.71
2.44
2.44
F
CF3
B(dan)
O
2j (Z/E > 99:1)
1H NMR (500 MHz, CDCl3)
1.00
1.02
1.07
2.04
2.07
2.00
2.01
2.05
1.97
8.5
8.0
7.5
7.0
6.5
6.0
5.5
5.0
4.5
4.0
3.5
3.0
2.5
2.0
1.5
1.0
0.5
0.0
δ (ppm)

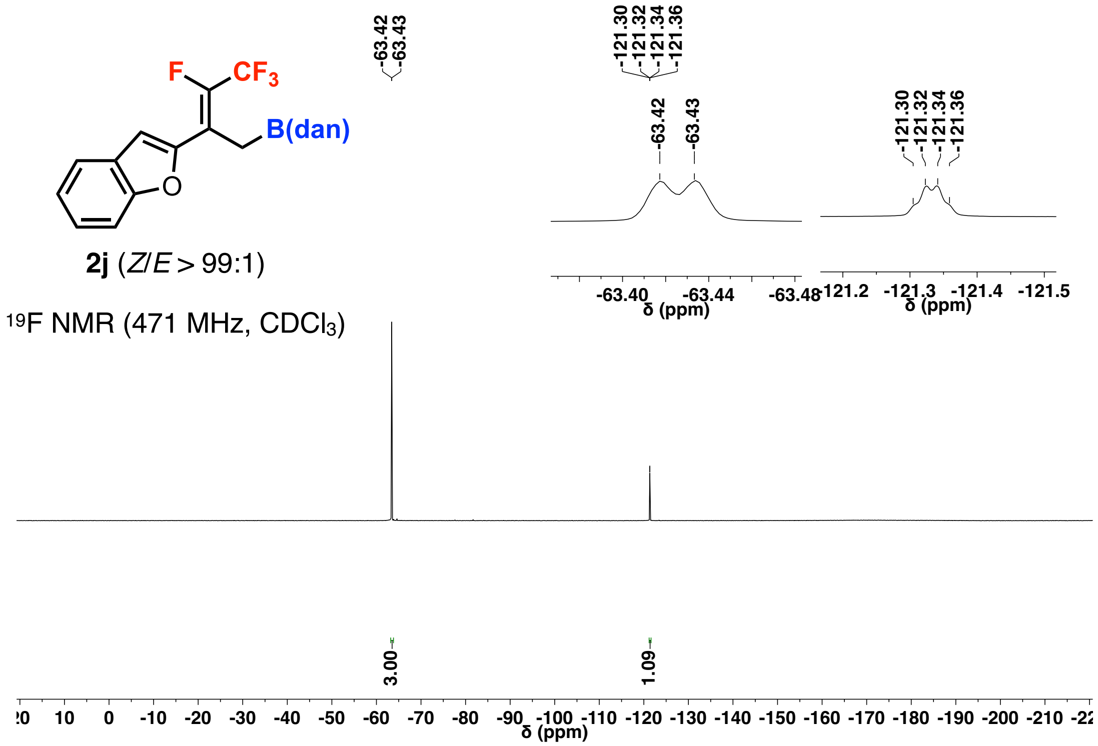

F
CF3
B(dan)
O
2j (Z/E > 99:1)
19F NMR (471 MHz, CDCl3)
-63.42
-63.43
-121.30
-121.32
-121.34
-121.36
-63.40
-63.44
-63.48
δ (ppm)
-121.2
-121.3
-121.4
-121.5
δ (ppm)
3.00
1.09
10
0
-10
-20
-30
-40
-50
-60
-70
-80
-90
-100
-110
-120
-130
-140
-150
-160
-170
-180
-190
-200
-210
δ (ppm)

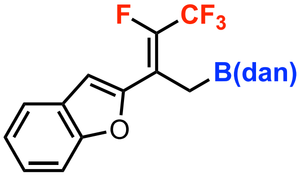

2j (*Z*/*E* > 99:1)

^{11}B NMR (160 MHz, $CDCl_3$)

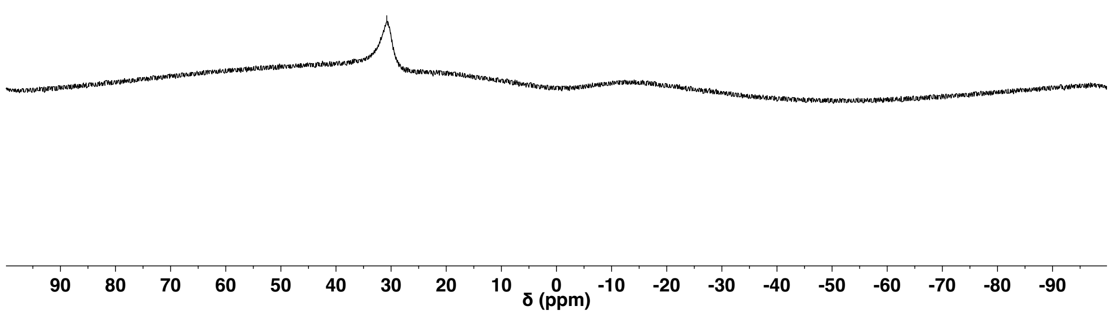

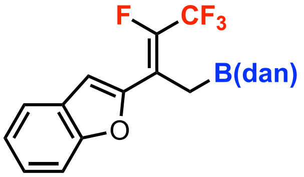

2j (*Z*/*E* > 99:1)

^{13}C NMR (126 MHz, $CDCl_3$)

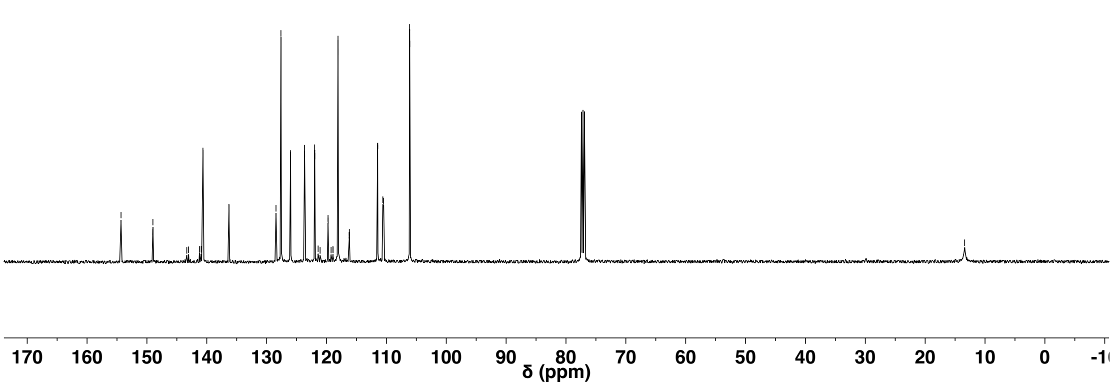

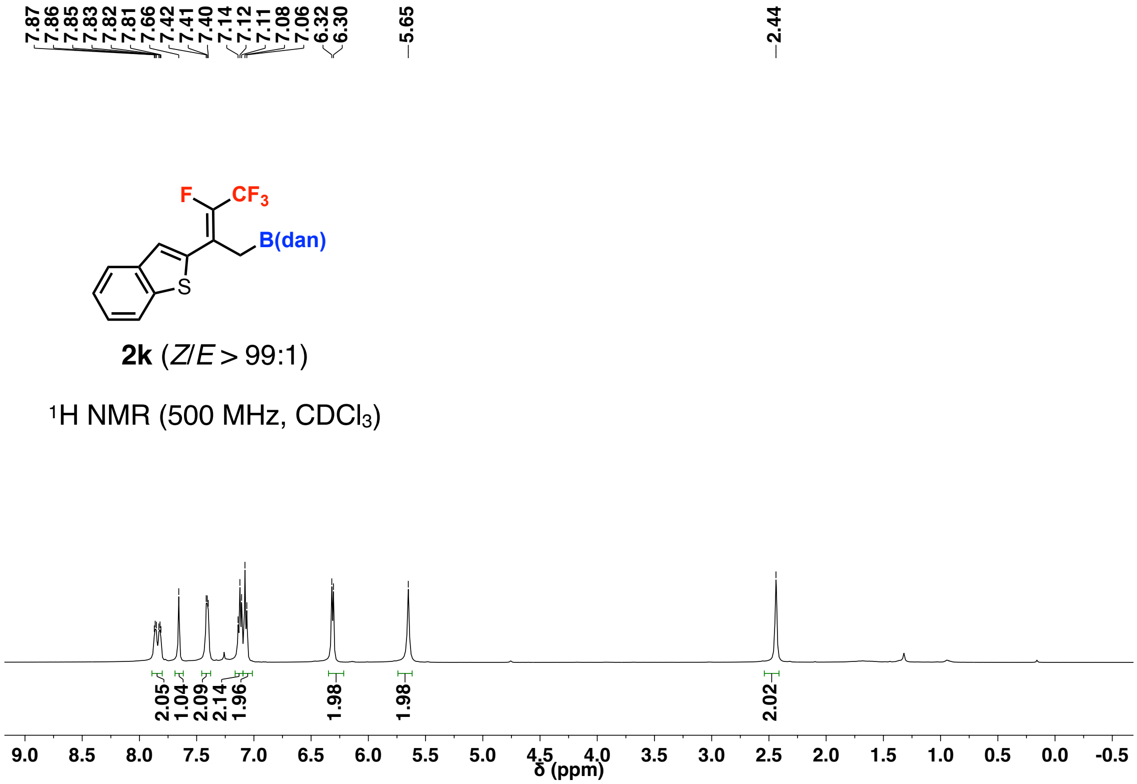
7.87 7.86 7.85 7.83 7.82 7.81 7.66 7.42 7.41 7.40 7.14 7.12 7.11 7.08 7.06 6.32 6.30
5.65
2.44
F
CF3
B(dan)
S
2k (Z/E > 99:1)
1H NMR (500 MHz, CDCl3)
2.05 1.04 2.09 2.14 1.96
1.98
1.98
2.02
9.0 8.5 8.0 7.5 7.0 6.5 6.0 5.5 5.0 4.5 4.0 3.5 3.0 2.5 2.0 1.5 1.0 0.5 0.0 -0.5
δ (ppm)

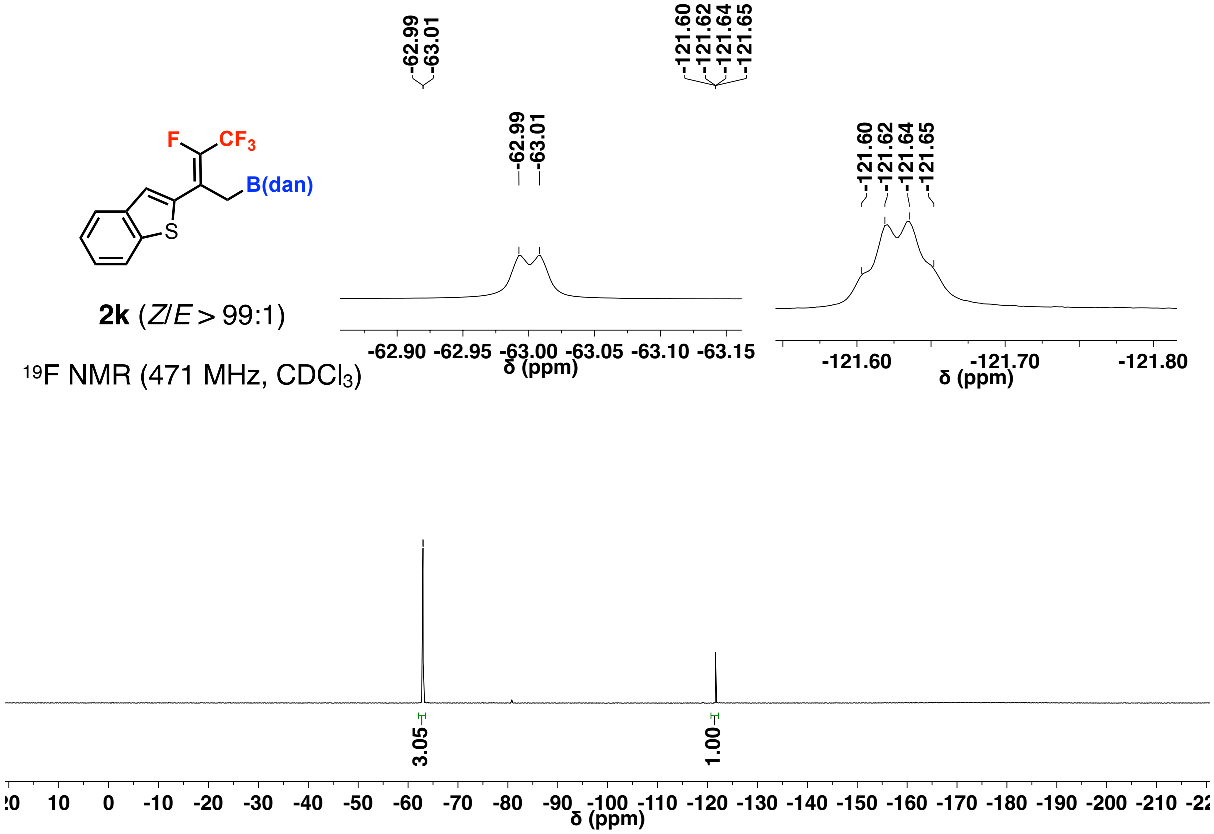
-62.99 -63.01
-121.60 -121.62 -121.64 -121.65
F
CF3
B(dan)
S
2k (Z/E > 99:1)
19F NMR (471 MHz, CDCl3)
-62.90 -62.95 -63.00 -63.05 -63.10 -63.15
δ (ppm)
-121.60 -121.70 -121.80
δ (ppm)
3.05
1.00
20 10 0 -10 -20 -30 -40 -50 -60 -70 -80 -90 -100 -110 -120 -130 -140 -150 -160 -170 -180 -190 -200 -210 -220
δ (ppm)

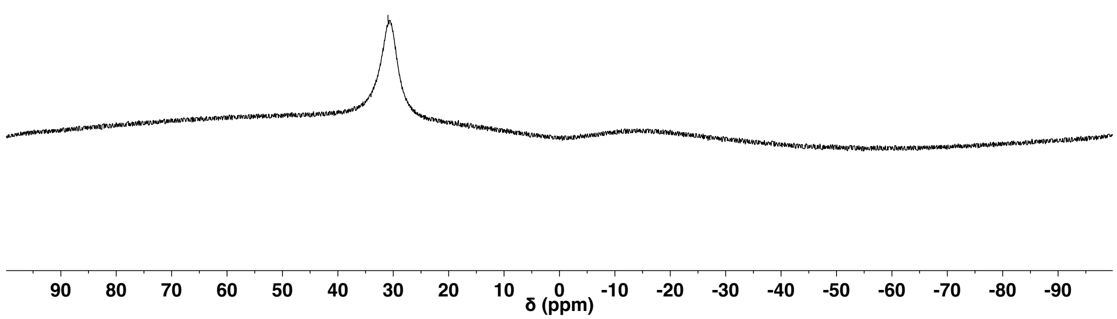

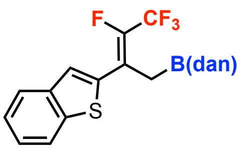

2k (*Z*/*E* > 99:1)

^{11}B NMR (160 MHz, $CDCl_3$)

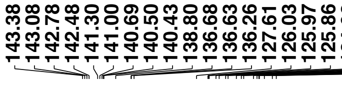
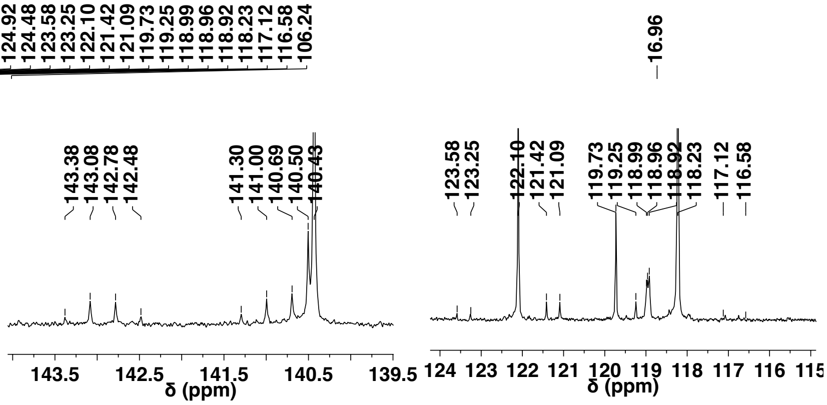

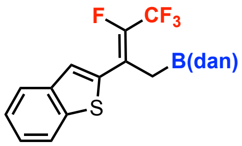

2k (*Z*/*E* > 99:1)

^{13}C NMR (126 MHz, $CDCl_3$)

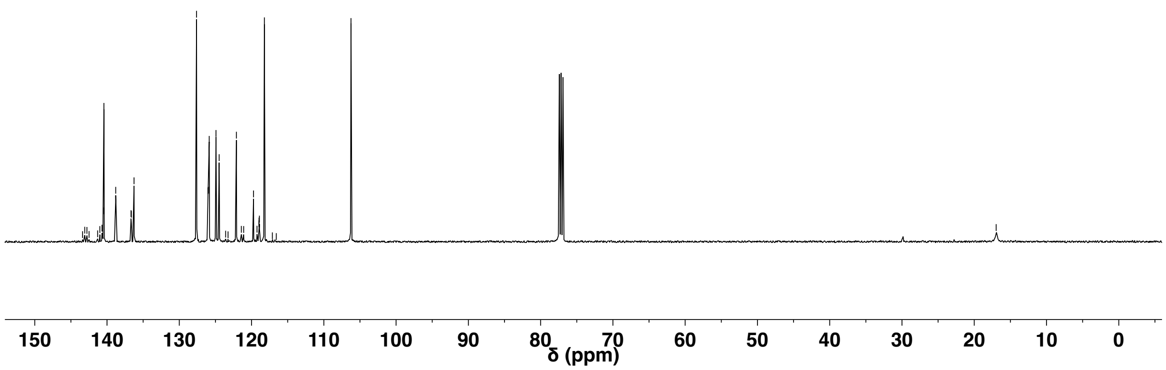

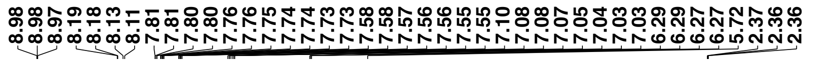

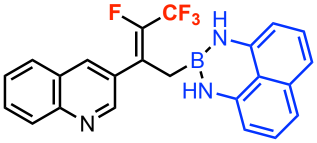

2l (*Z*/*E* > 99:1)

^1H NMR (500 MHz, $CDCl_3$)

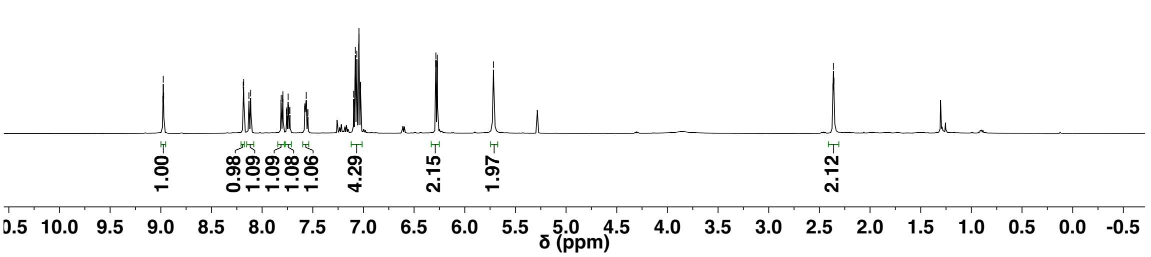

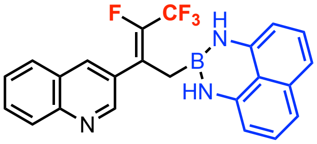

2l (*Z*/*E* > 99:1)

^{19}F NMR (471 MHz, $CDCl_3$)

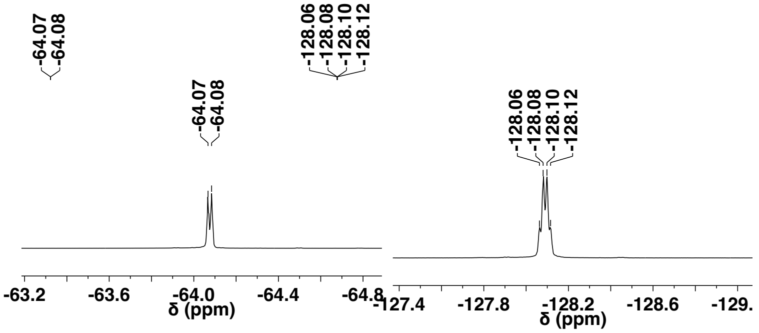

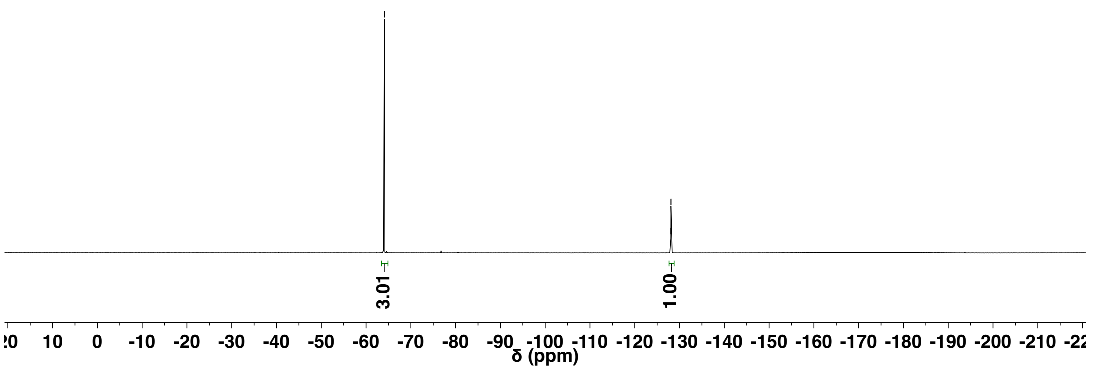

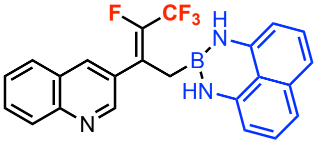

2l (*Z*/*E* > 99:1)

^{11}B NMR (160 MHz, $CDCl_3$)

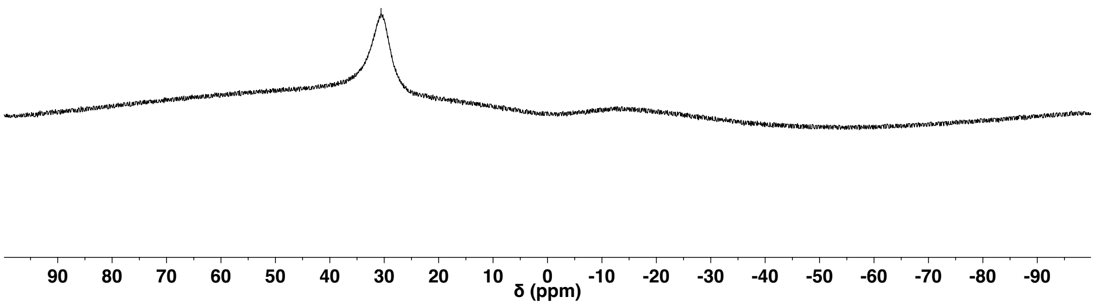

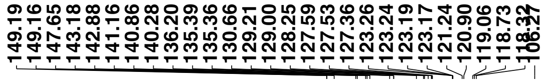

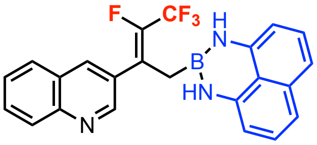

2l (*Z*/*E* > 99:1)

^{13}C NMR (101 MHz, $CDCl_3$)

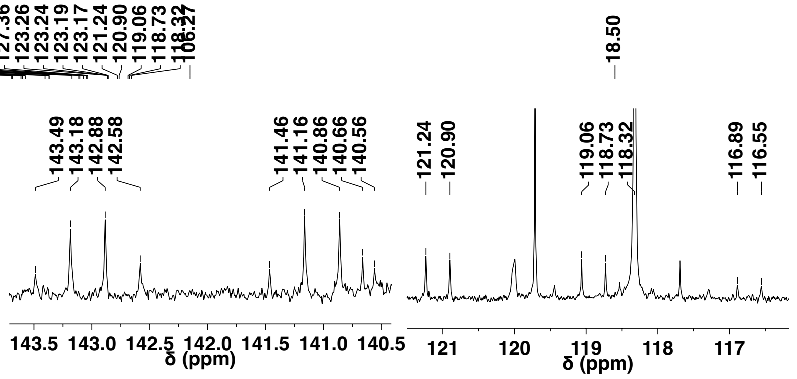

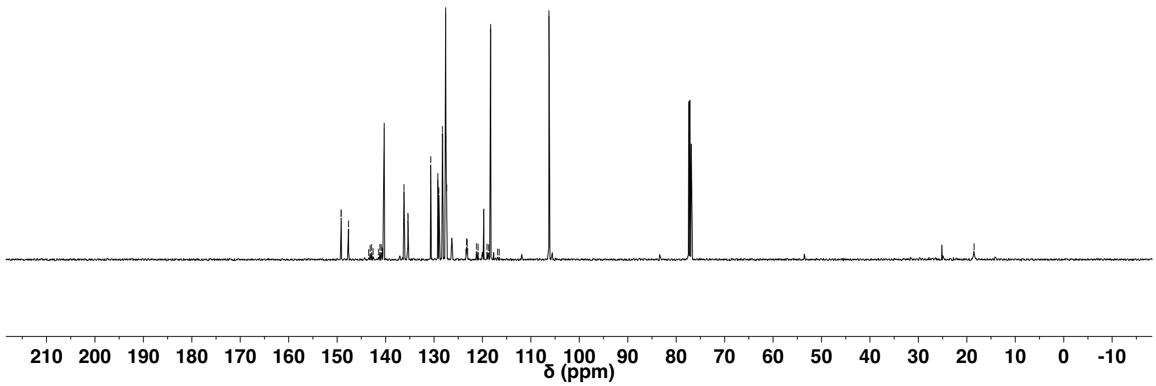

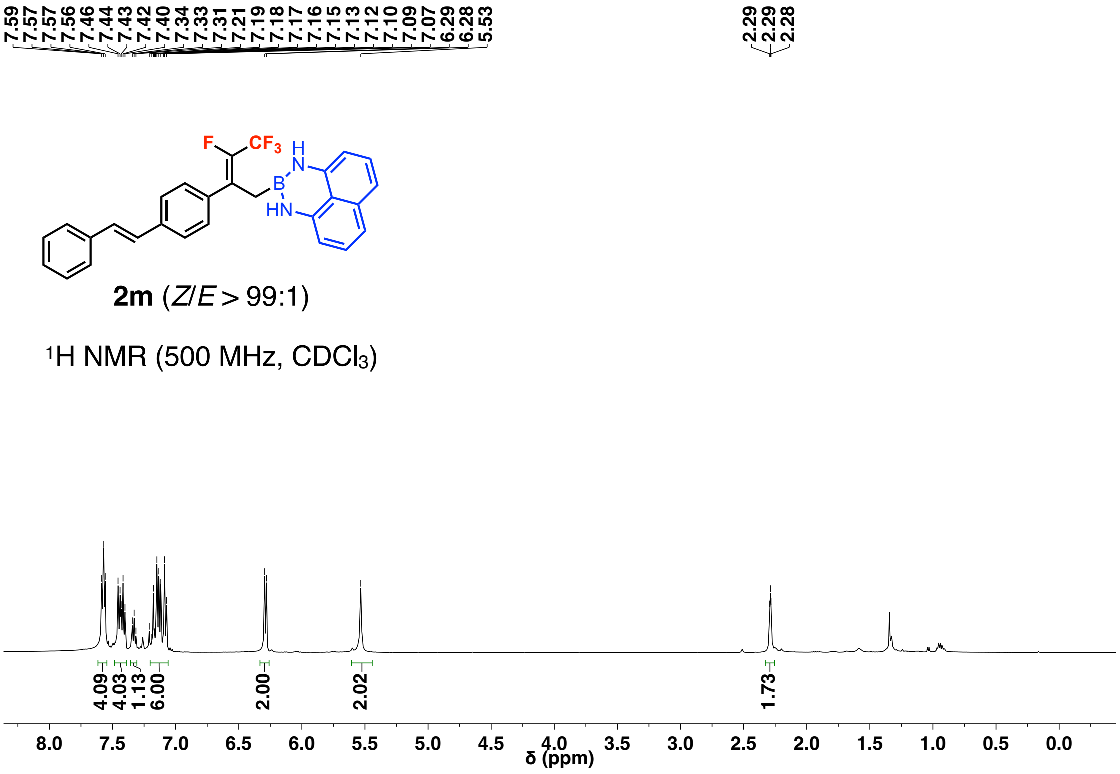

2m (*Z*/*E* > 99:1)

^{1}H NMR (500 MHz, $CDCl_3$)

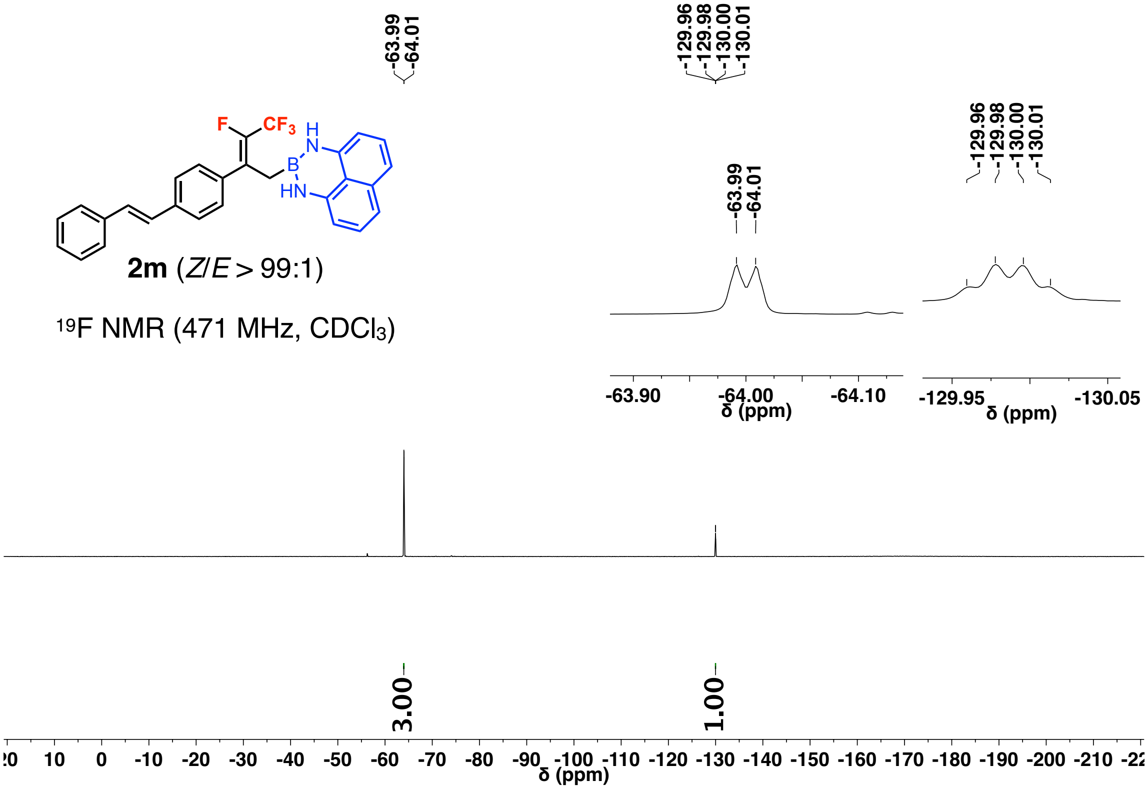

2m (*Z*/*E* > 99:1)

^{19}F NMR (471 MHz, $CDCl_3$)

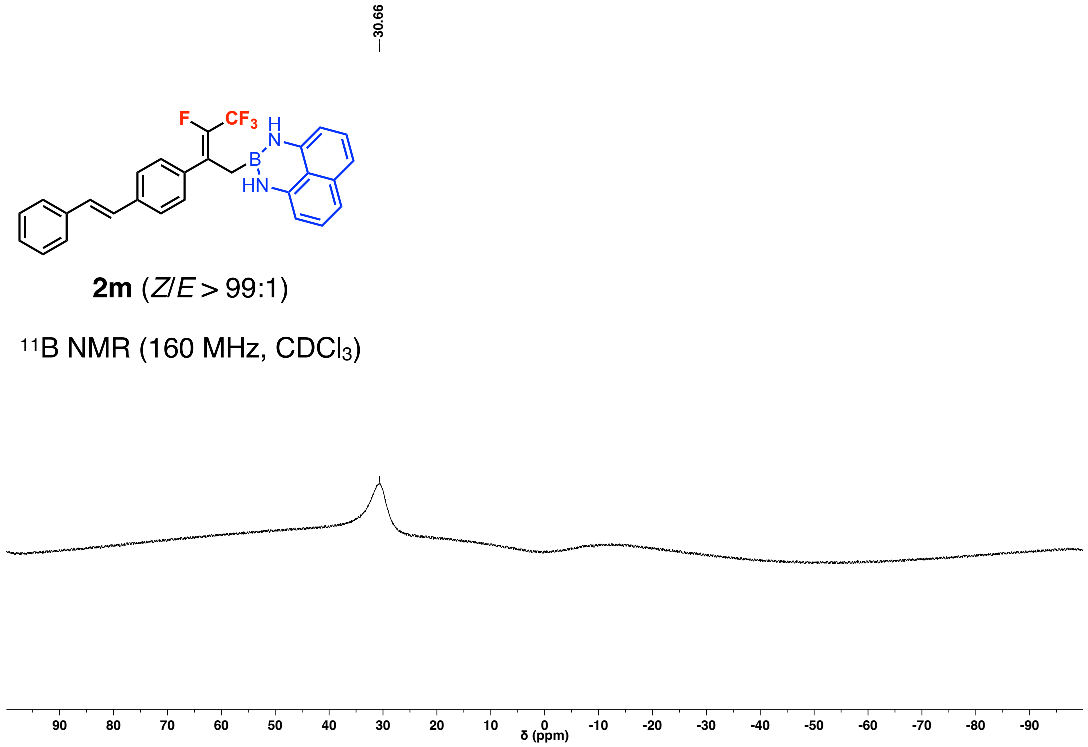
30.66
F
CF3
H
N
B
HN
2m (Z/E > 99:1)
11B NMR (160 MHz, CDCl3)
δ (ppm)

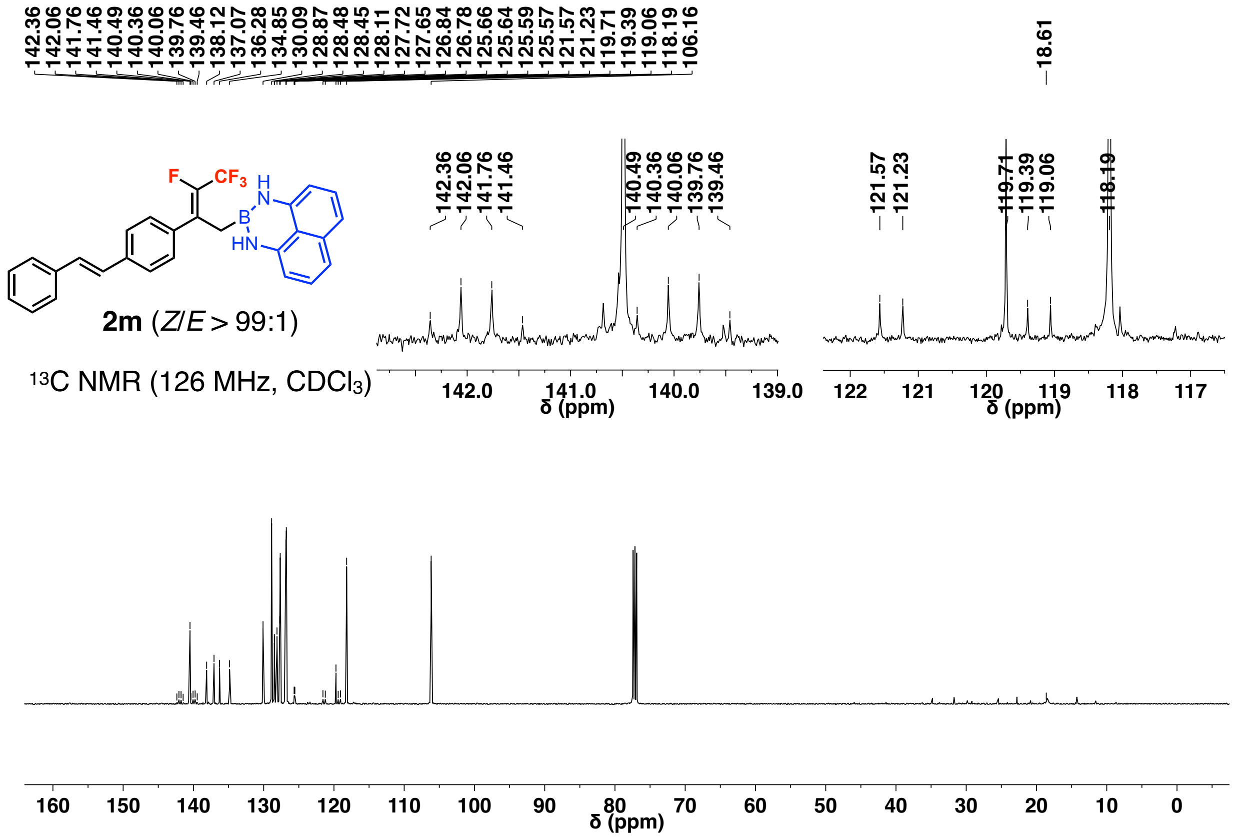
142.36
142.06
141.76
141.46
140.49
140.36
140.06
139.76
139.46
138.12
137.07
136.28
134.85
130.09
128.87
128.48
128.45
128.11
127.72
127.65
126.84
126.78
125.66
125.64
125.59
125.57
121.57
121.23
119.71
119.39
119.06
118.19
106.16
18.61
F
CF3
H
N
B
HN
2m (Z/E > 99:1)
13C NMR (126 MHz, CDCl3)
δ (ppm)

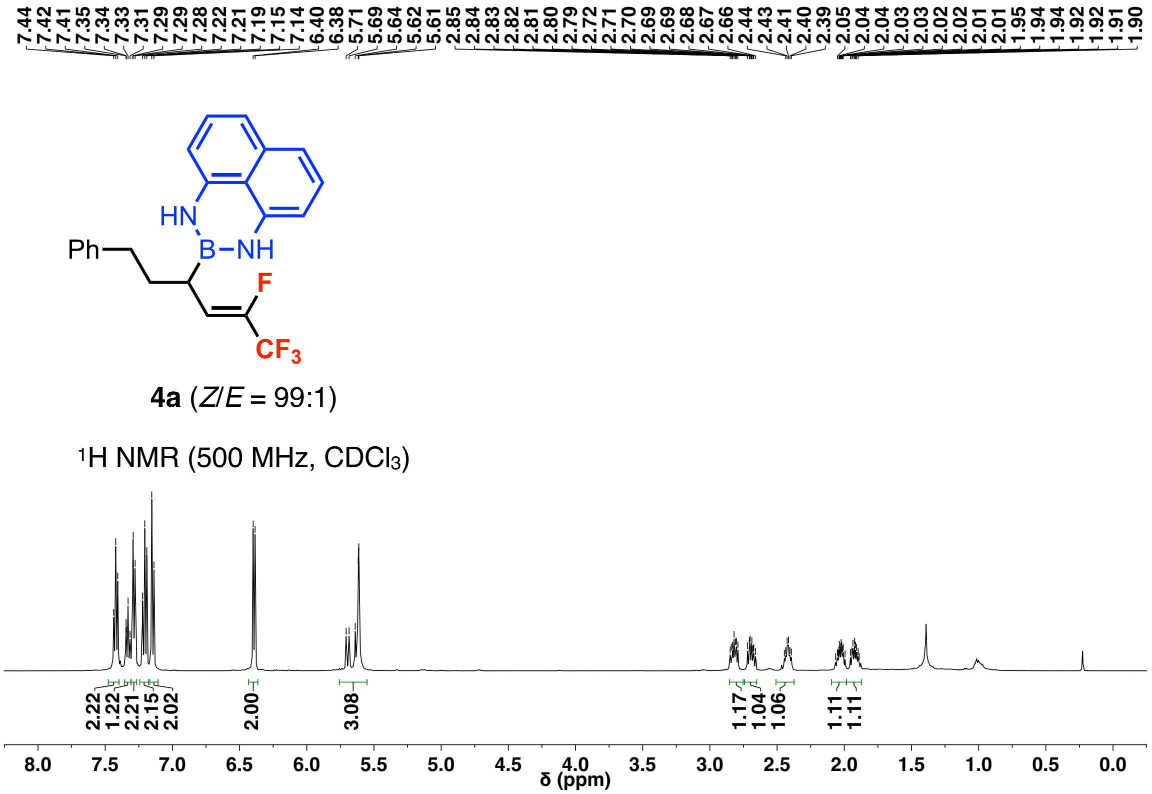
7.44 7.42 7.41 7.35 7.34 7.33 7.31 7.29 7.29 7.28 7.22 7.21 7.19 7.15 7.14 6.40 6.38 5.71 5.69 5.64 5.62 5.61 2.85 2.84 2.83 2.82 2.81 2.80 2.79 2.72 2.71 2.70 2.69 2.69 2.68 2.67 2.66 2.44 2.43 2.41 2.40 2.39 2.05 2.04 2.04 2.03 2.03 2.02 2.02 2.01 2.01 1.95 1.94 1.94 1.92 1.92 1.91 1.90
HN
B
NH
Ph
F
CF3
4a (Z/E = 99:1)
1H NMR (500 MHz, CDCl3)
2.22 1.22 2.21 2.15 2.02 2.00 3.08 1.17 1.04 1.06 1.11 1.11
δ (ppm)

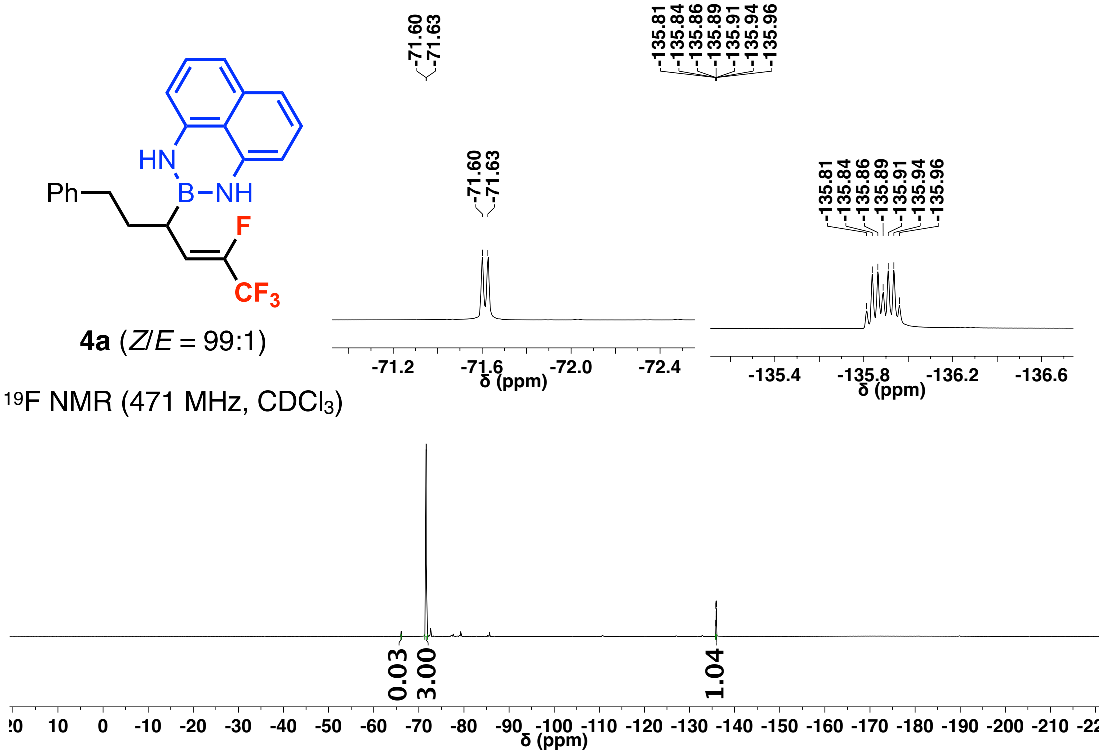
-71.60 -71.63
-135.81 -135.84 -135.86 -135.89 -135.91 -135.94 -135.96
HN
B
NH
Ph
F
CF3
4a (Z/E = 99:1)
19F NMR (471 MHz, CDCl3)
0.03 3.00 1.04
δ (ppm)

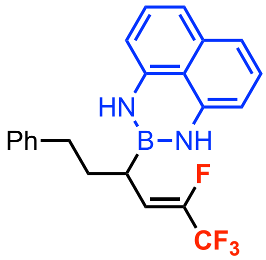

4a (*Z*/*E* = 99:1)

^{11}B NMR (160 MHz, $CDCl_3$)

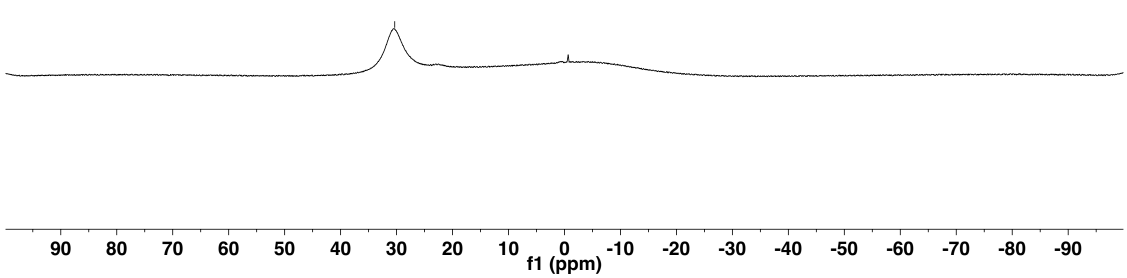

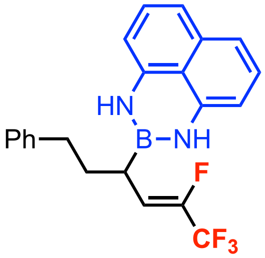

4a (*Z*/*E* = 99:1)

^{13}C NMR (126 MHz, $CDCl_3$)

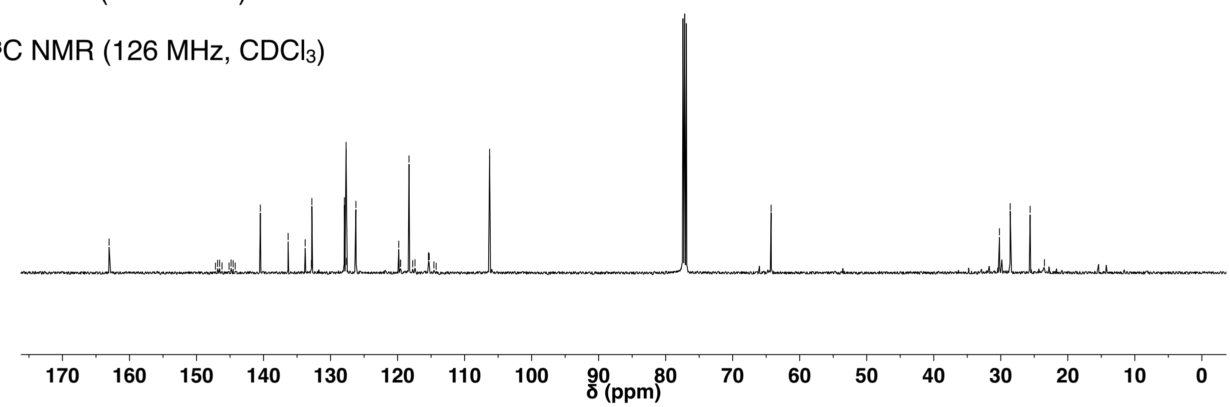

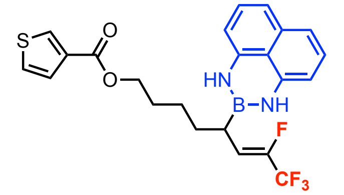

4b (*Z*/*E* = 98:2)

^{1}H NMR (500 MHz, $CDCl_3$)

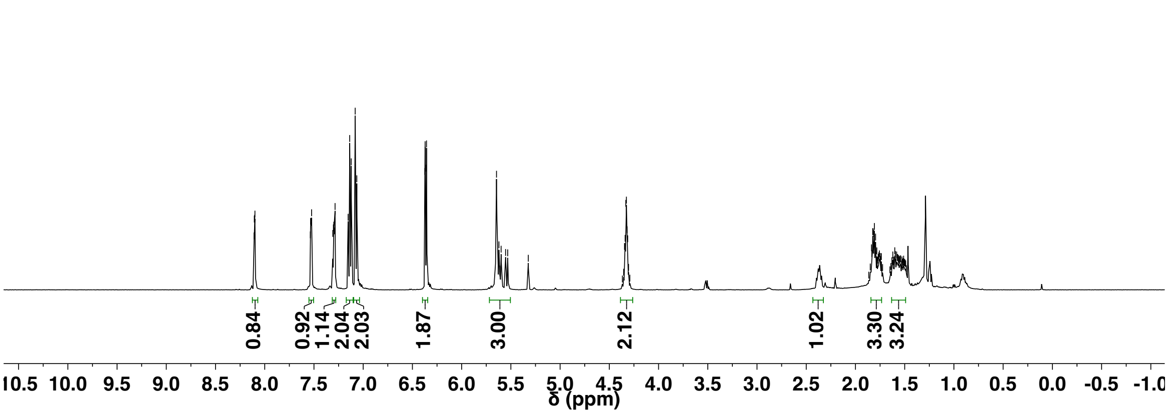

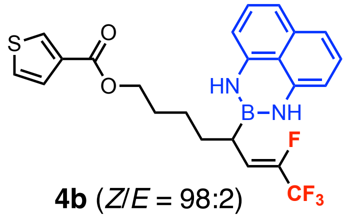

4b (*Z*/*E* = 98:2)

^{19}F NMR (471 MHz, $CDCl_3$)

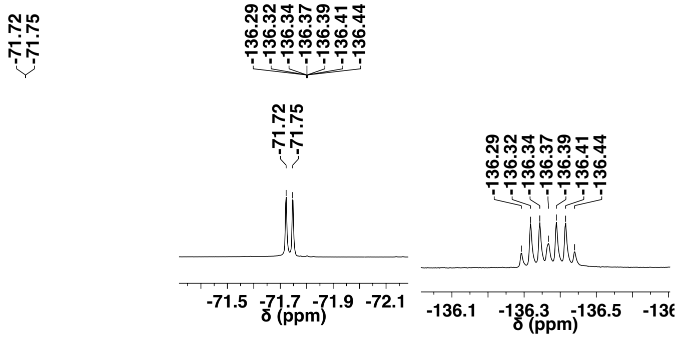

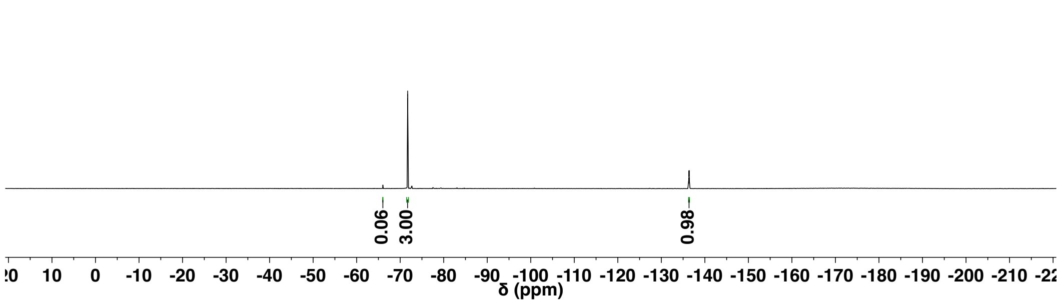

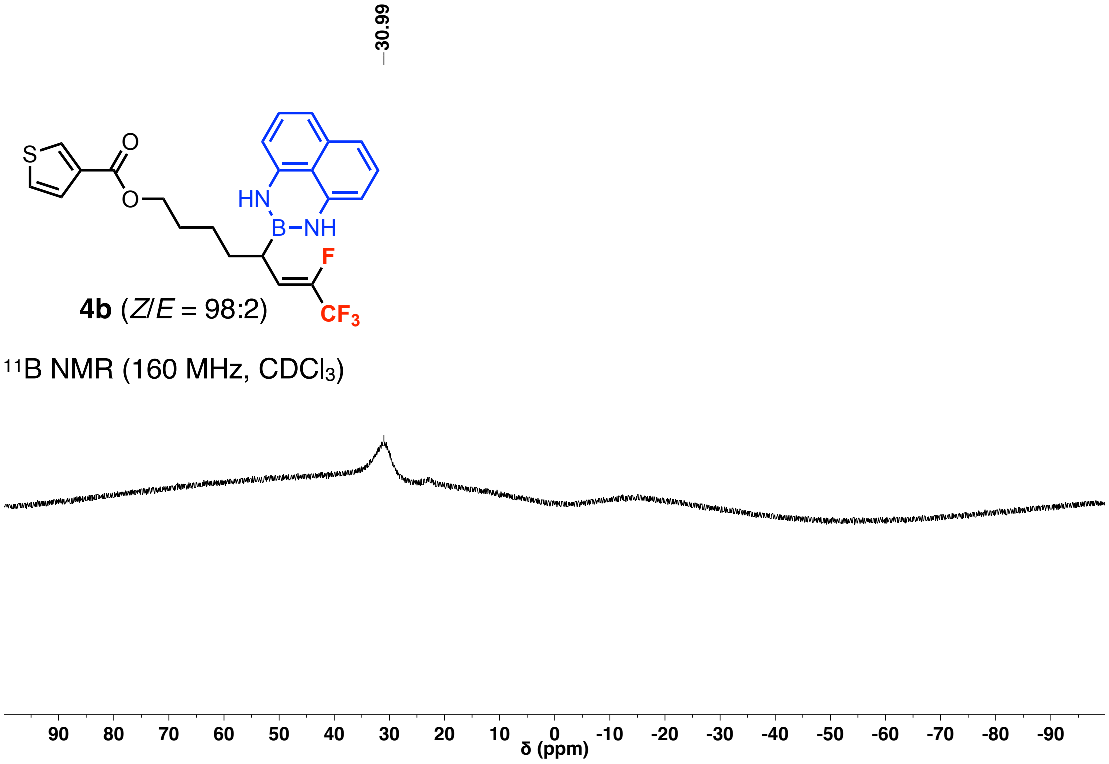

4b (*Z*/*E* = 98:2)

^{11}B NMR (160 MHz, $CDCl_3$)

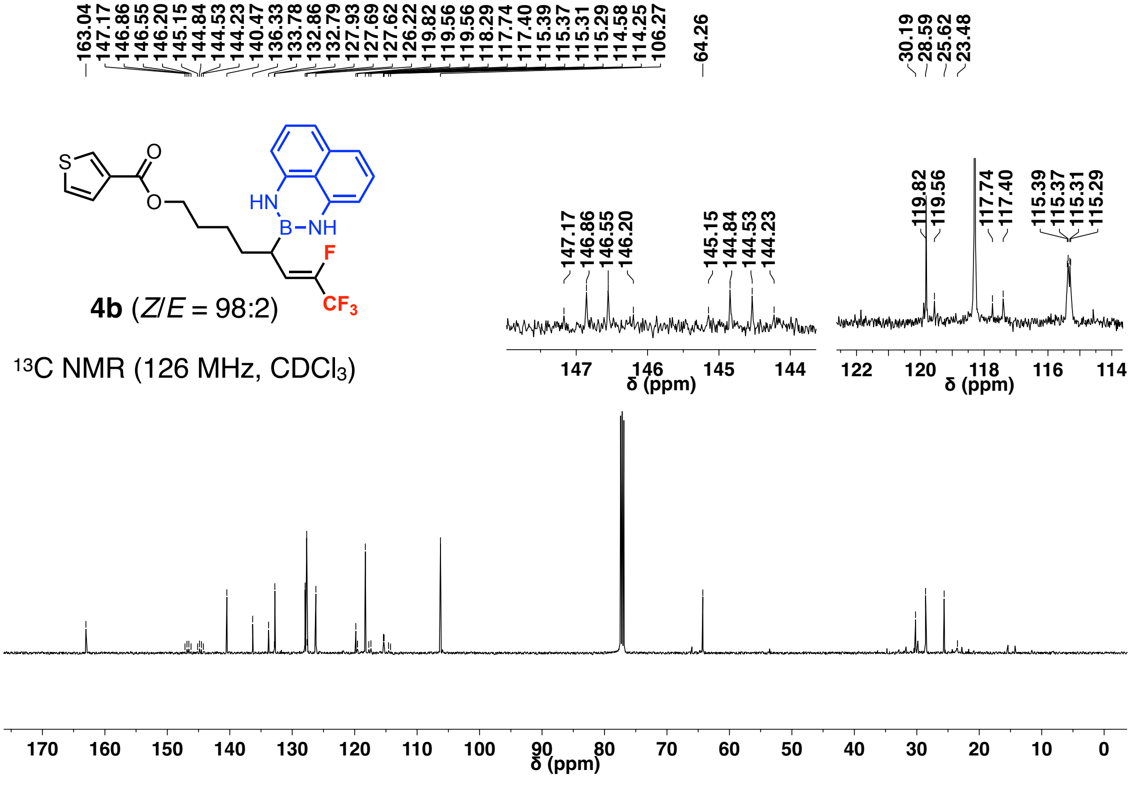

4b (*Z*/*E* = 98:2)

^{13}C NMR (126 MHz, $CDCl_3$)

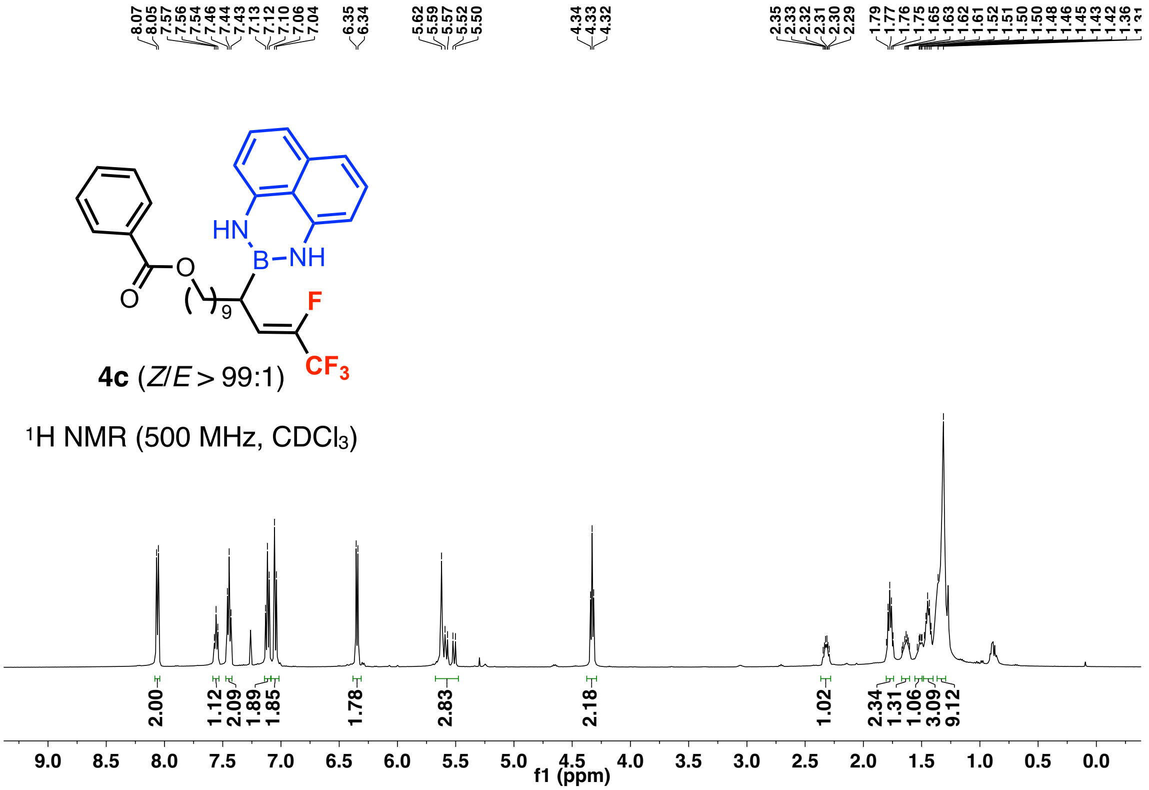

8.07
8.05
7.57
7.56
7.54
7.46
7.44
7.43
7.13
7.12
7.10
7.06
7.04
6.35
6.34
5.62
5.59
5.57
5.52
5.50
4.34
4.33
4.32
2.35
2.33
2.32
2.31
2.30
2.29
1.79
1.77
1.76
1.75
1.65
1.63
1.62
1.61
1.52
1.51
1.50
1.48
1.46
1.45
1.43
1.42
1.36
HN
B
NH
O
O
9
F
CF_3
4c (*Z*/*E* > 99:1)
^{1}H NMR (500 MHz, $CDCl_3$)
2.00
1.12
2.09
1.89
1.85
1.78
2.83
2.18
1.02
2.34
1.31
1.06
3.09
9.12
9.0 8.5 8.0 7.5 7.0 6.5 6.0 5.5 5.0 4.5 4.0 3.5 3.0 2.5 2.0 1.5 1.0 0.5 0.0
f1 (ppm)

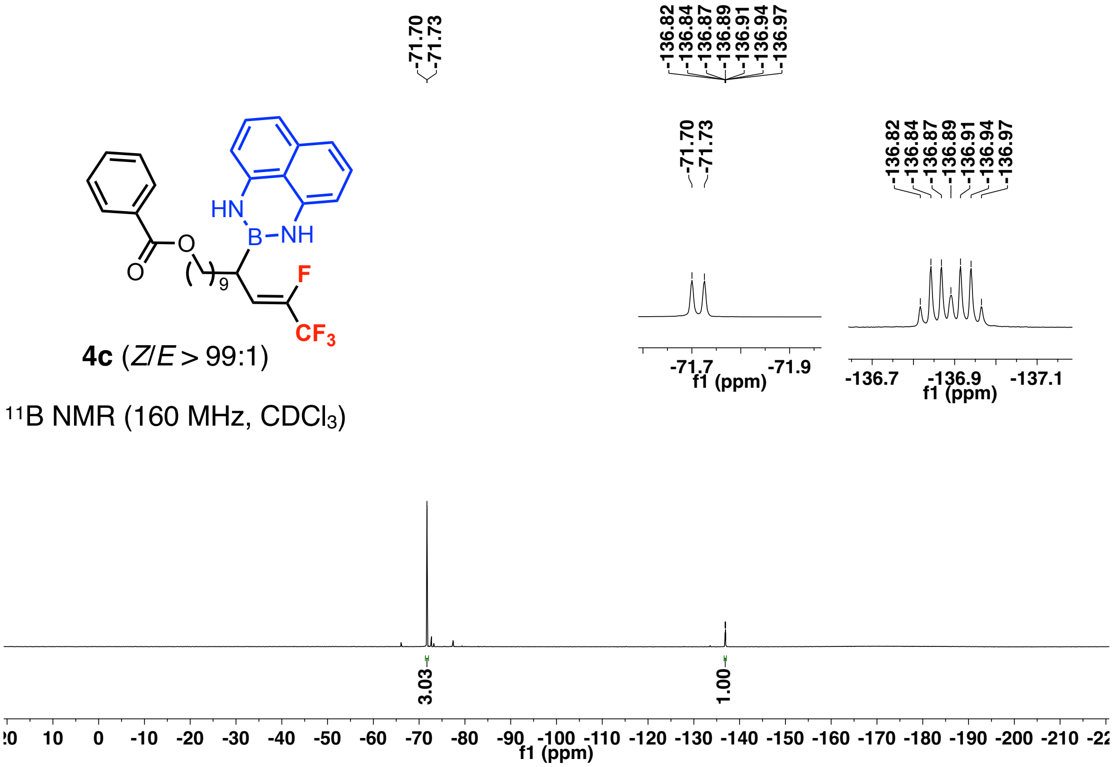

-71.70
-71.73
-136.82
-136.84
-136.87
-136.89
-136.91
-136.94
-136.97
HN
B
NH
O
O
9
F
CF_3
4c (*Z*/*E* > 99:1)
^{11}B NMR (160 MHz, $CDCl_3$)
-71.7
-71.9
f1 (ppm)
-136.7
-136.9
-137.1
f1 (ppm)
3.03
1.00
10 0 -10 -20 -30 -40 -50 -60 -70 -80 -90 -100 -110 -120 -130 -140 -150 -160 -170 -180 -190 -200 -210
f1 (ppm)

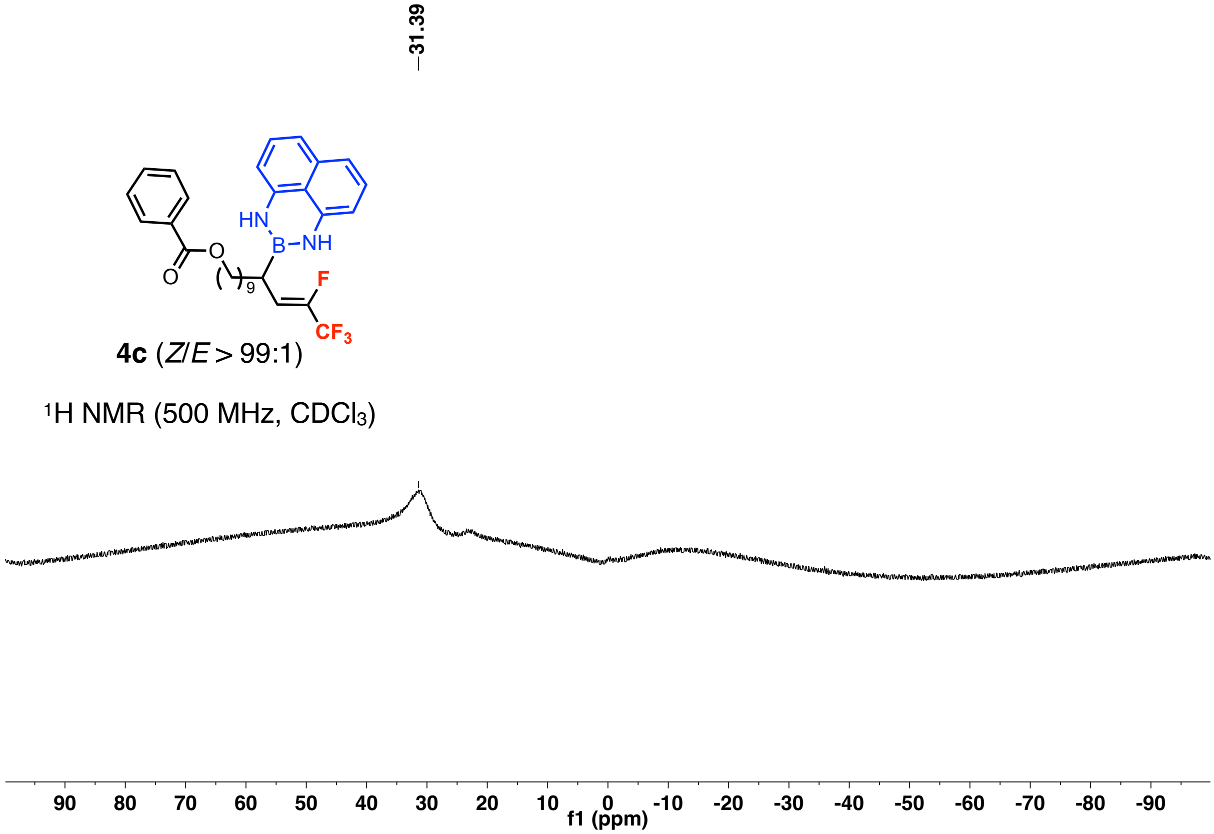

31.39
HN
B
NH
O
O
9
F
CF3
4c (Z/E > 99:1)
1H NMR (500 MHz, CDCl3)
90 80 70 60 50 40 30 20 10 0 -10 -20 -30 -40 -50 -60 -70 -80 -90
f1 (ppm)

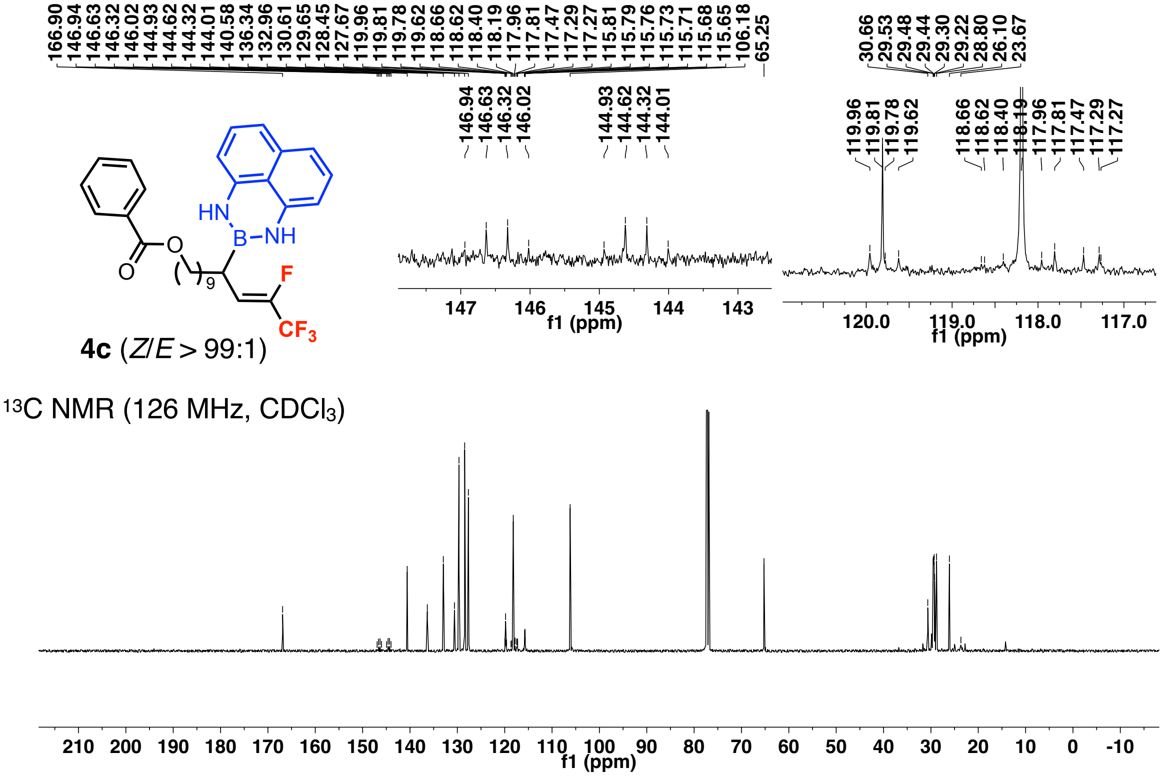

166.90 146.94 146.63 146.32 146.02 144.93 144.62 144.32 144.01 140.58 136.34 132.96 130.61 129.65 128.45 127.67 119.96 119.81 119.78 119.62 118.66 118.62 118.40 118.19 117.96 117.81 117.47 117.29 117.27 115.81 115.79 115.76 115.73 115.71 115.68 115.65 106.18
65.25
30.66 29.53 29.48 29.44 29.30 29.22 28.80 26.10 23.67
146.94 146.63 146.32 146.02
144.93 144.62 144.32 144.01
147 146 145 144 143
f1 (ppm)
119.96 119.81 119.78 119.62
118.66 118.62 118.40 118.19 117.96 117.81 117.47 117.29 117.27
120.0 119.0 118.0 117.0
f1 (ppm)
HN
B
NH
O
O
9
F
CF3
4c (Z/E > 99:1)
13C NMR (126 MHz, CDCl3)
210 200 190 180 170 160 150 140 130 120 110 100 90 80 70 60 50 40 30 20 10 0 -10
f1 (ppm)

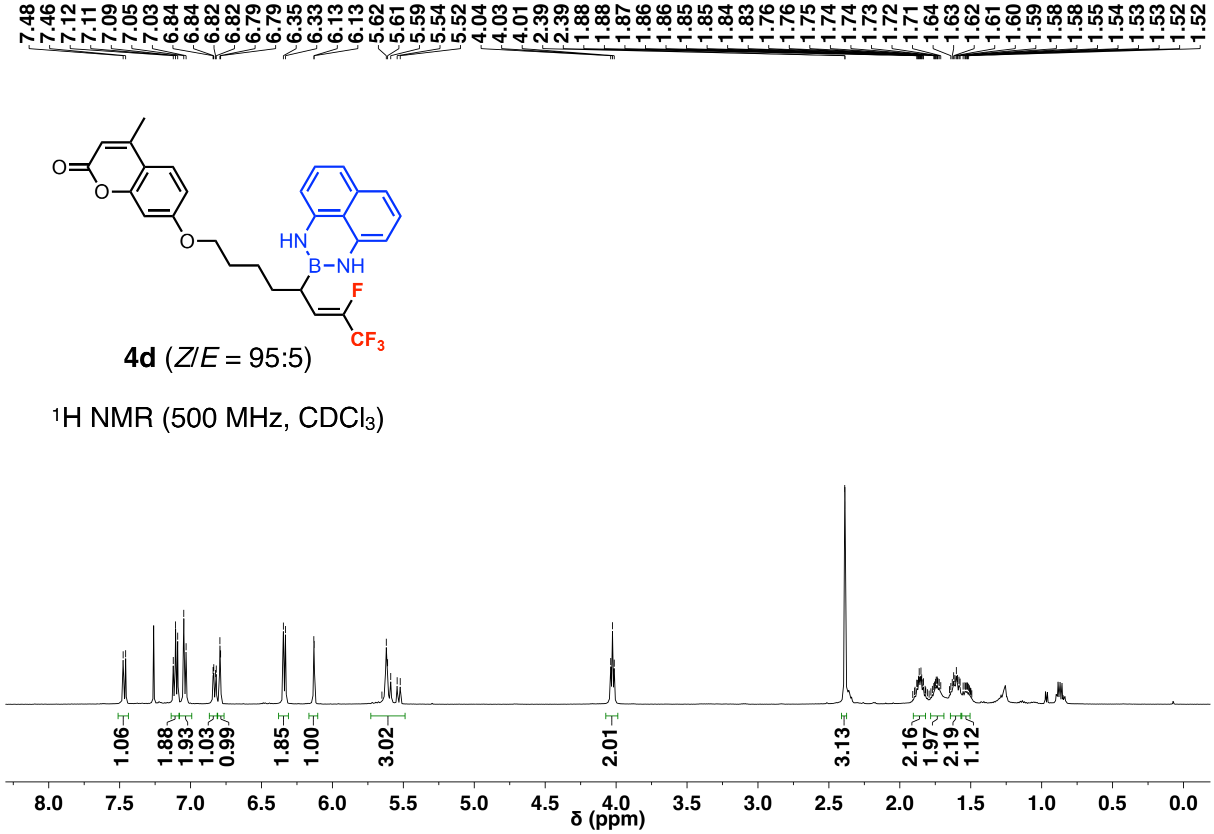
7.48
7.46
7.12
7.11
7.09
7.05
7.03
6.84
6.84
6.82
6.82
6.79
6.79
6.35
6.33
6.13
6.13
5.62
5.61
5.59
5.54
5.52
4.04
4.03
4.01
2.39
2.39
1.88
1.88
1.87
1.86
1.86
1.85
1.85
1.84
1.83
1.76
1.76
1.75
1.74
1.74
1.73
1.72
1.71
1.64
1.63
1.62
1.61
1.60
1.59
1.58
1.58
1.55
1.54
1.53
1.53
1.52
1.52
O
O
O
HN
B
NH
F
CF_3
4d (*Z*/*E* = 95:5)
^{1}H NMR (500 MHz, $CDCl_3$)
1.06
1.88
1.93
1.03
0.99
1.85
1.00
3.02
2.01
3.13
2.16
1.97
2.19
1.12
8.0
7.5
7.0
6.5
6.0
5.5
5.0
4.5
4.0
3.5
3.0
2.5
2.0
1.5
1.0
0.5
0.0
δ (ppm)

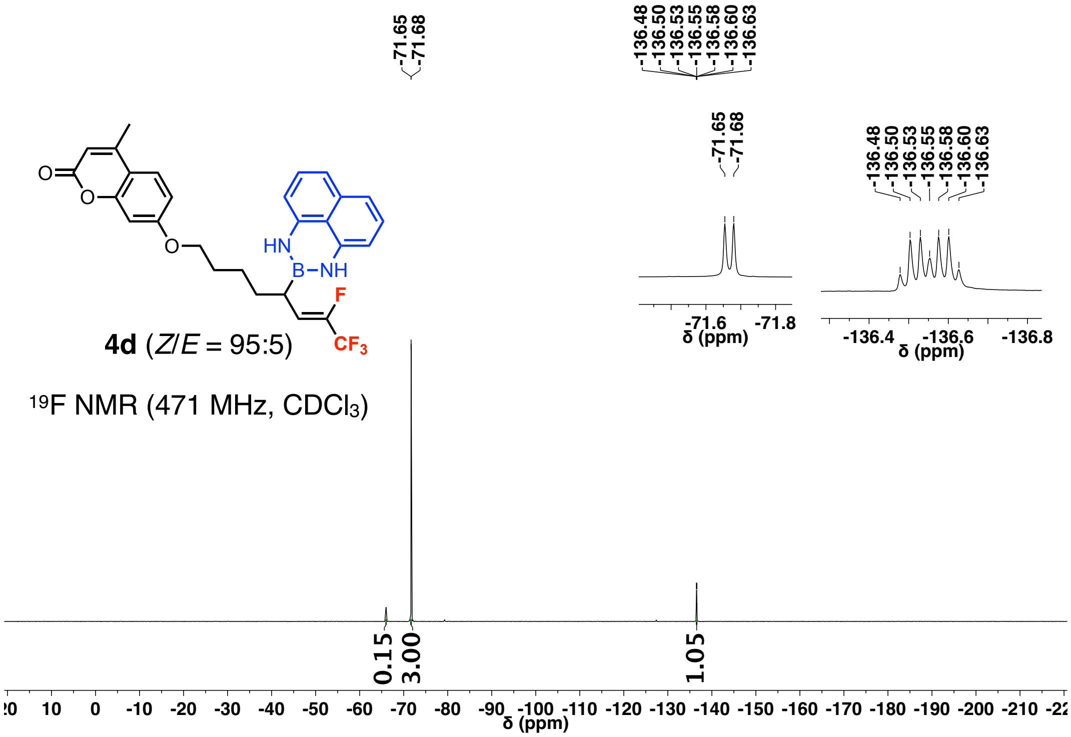
-71.65
-71.68
-136.48
-136.50
-136.53
-136.55
-136.58
-136.60
-136.63
O
O
O
HN
B
NH
F
CF_3
4d (*Z*/*E* = 95:5)
^{19}F NMR (471 MHz, $CDCl_3$)
-71.6
-71.8
δ (ppm)
-136.4
-136.6
-136.8
δ (ppm)
0.15
3.00
1.05
10
0
-10
-20
-30
-40
-50
-60
-70
-80
-90
-100
-110
-120
-130
-140
-150
-160
-170
-180
-190
-200
-210
δ (ppm)

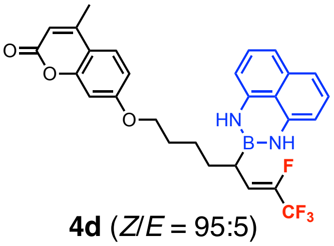

4d (*Z*/*E* = 95:5)

^{11}B NMR (160 MHz, $CDCl_3$)

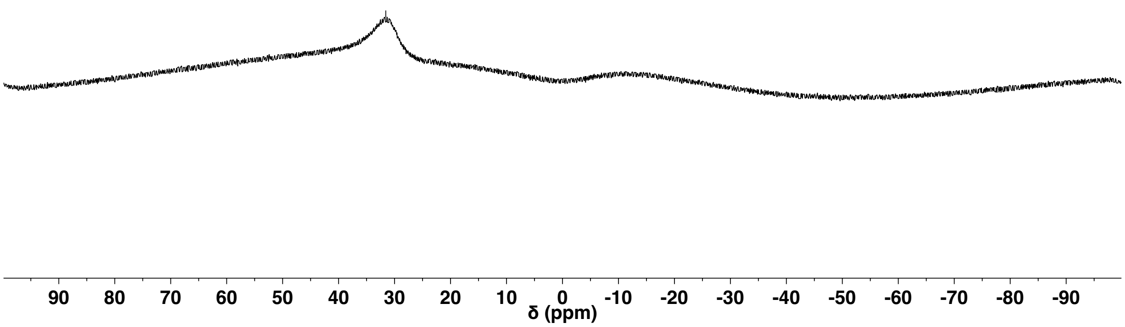

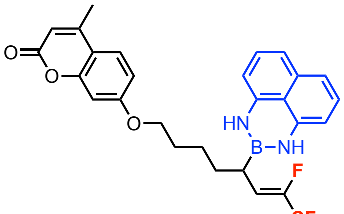

4d (*Z*/*E* = 95:5)

^{13}C NMR (126 MHz, $CDCl_3$)

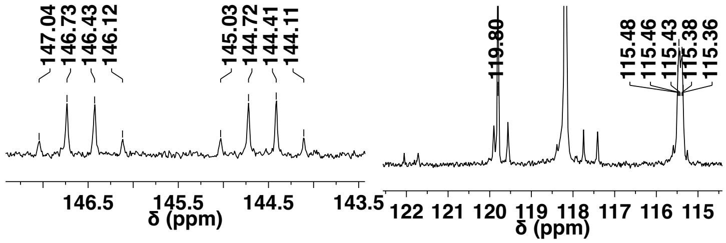

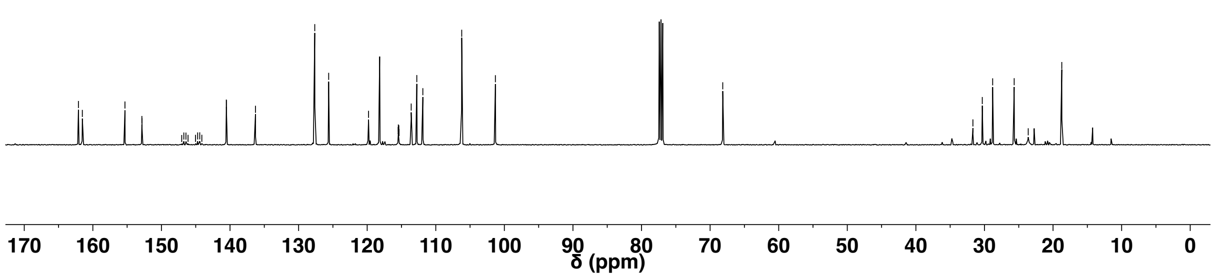

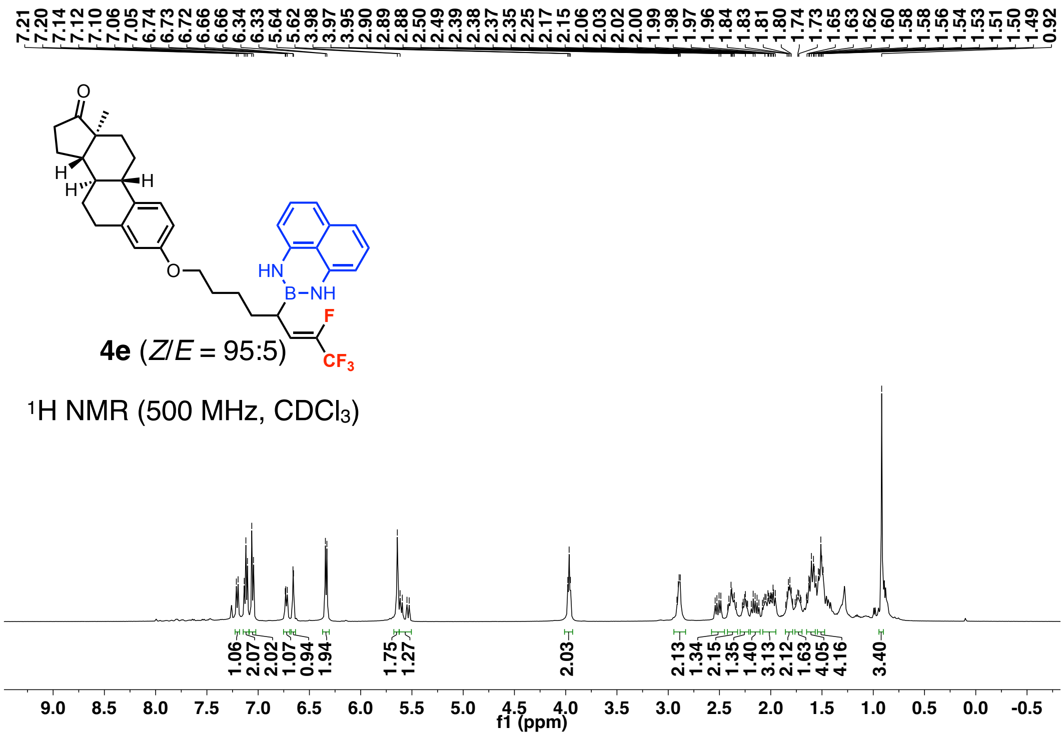
7.21
7.20
7.14
7.12
7.10
7.06
7.05
6.74
6.73
6.72
6.66
6.66
6.34
6.33
5.64
5.62
3.98
3.97
3.95
2.90
2.89
2.88
2.50
2.49
2.39
2.38
2.37
2.35
2.25
2.17
2.15
2.06
2.03
2.02
2.00
1.99
1.98
1.97
1.96
1.84
1.83
1.81
1.80
1.74
1.73
1.65
1.63
1.62
1.60
1.58
1.58
1.56
1.54
1.53
1.51
1.50
1.49
0.92
O
H
H
H
O
HN
B–NH
F
CF3
4e (Z/E = 95:5)
1H NMR (500 MHz, CDCl3)
1.06
2.07
2.02
1.07
0.94
1.94
1.75
1.27
2.03
2.13
1.34
2.15
1.35
1.40
3.13
2.12
1.63
4.05
4.16
3.40
9.0
8.5
8.0
7.5
7.0
6.5
6.0
5.5
5.0
4.5
4.0
3.5
3.0
2.5
2.0
1.5
1.0
0.5
0.0
-0.5
f1 (ppm)

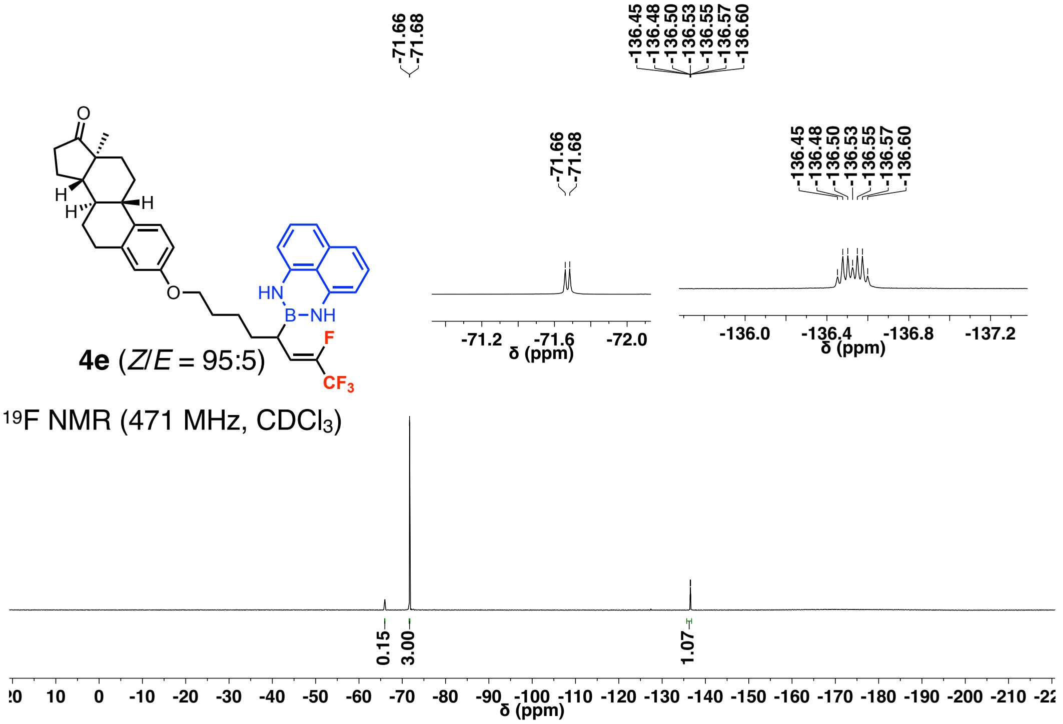
-71.66
-71.68
-136.45
-136.48
-136.50
-136.53
-136.55
-136.57
-136.60
O
H
H
H
O
HN
B–NH
F
CF3
4e (Z/E = 95:5)
-71.66
-71.68
-71.2
-71.6
-72.0
δ (ppm)
-136.45
-136.48
-136.50
-136.53
-136.55
-136.57
-136.60
-136.0
-136.4
-136.8
-137.2
δ (ppm)
19F NMR (471 MHz, CDCl3)
0.15
3.00
1.07
10
0
-10
-20
-30
-40
-50
-60
-70
-80
-90
-100
-110
-120
-130
-140
-150
-160
-170
-180
-190
-200
-210
δ (ppm)

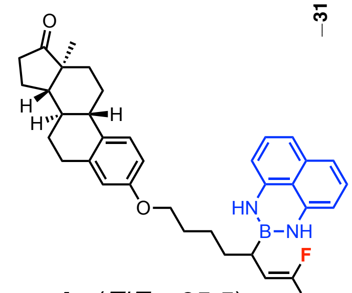

4e (*Z*/*E* = 95:5)

^{11}B NMR (160 MHz, CDCl$_3$)

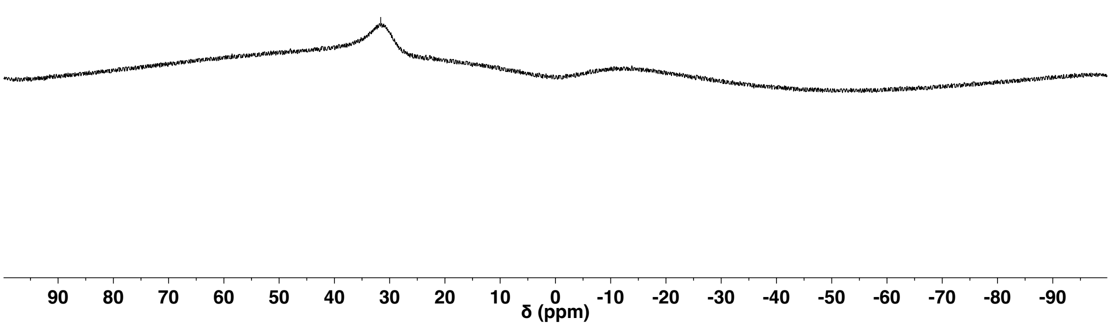

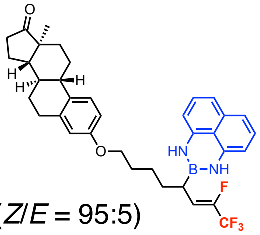

4e (*Z*/*E* = 95:5)

^{13}C NMR (126 MHz, CDCl$_3$)

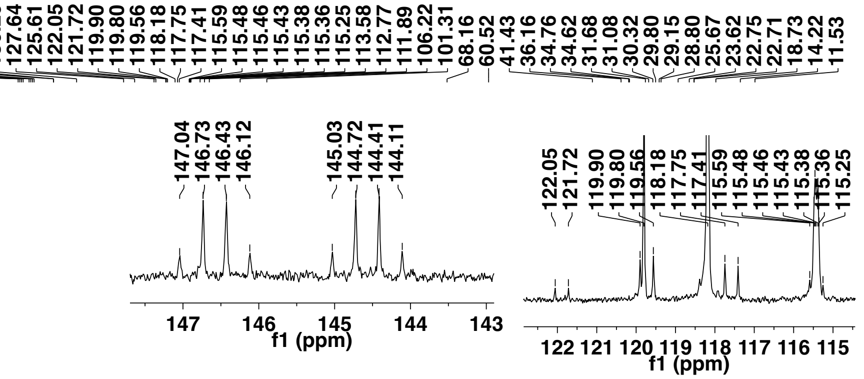

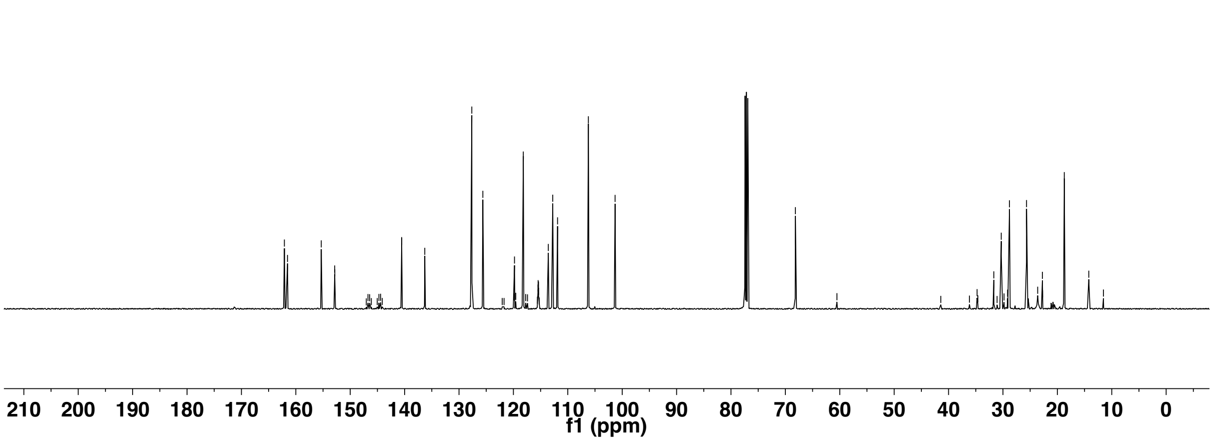

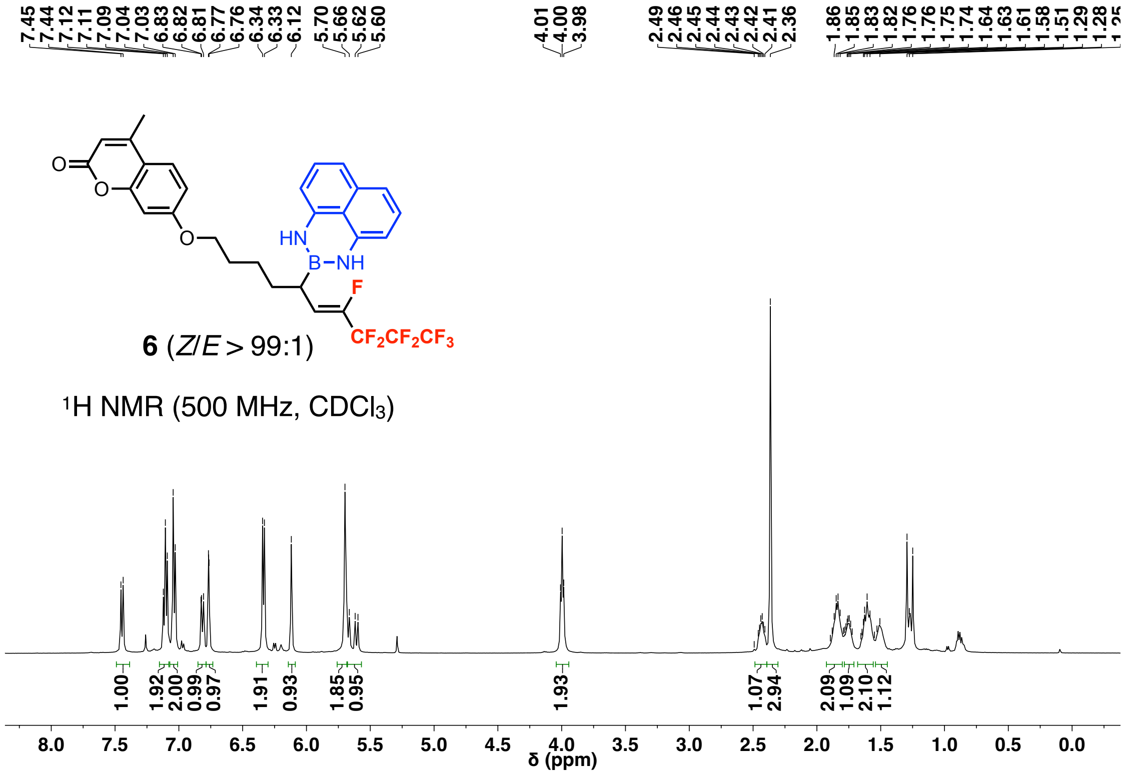
6 (Z/E > 99:1)
CF2CF2CF3
1H NMR (500 MHz, CDCl3)
δ (ppm)

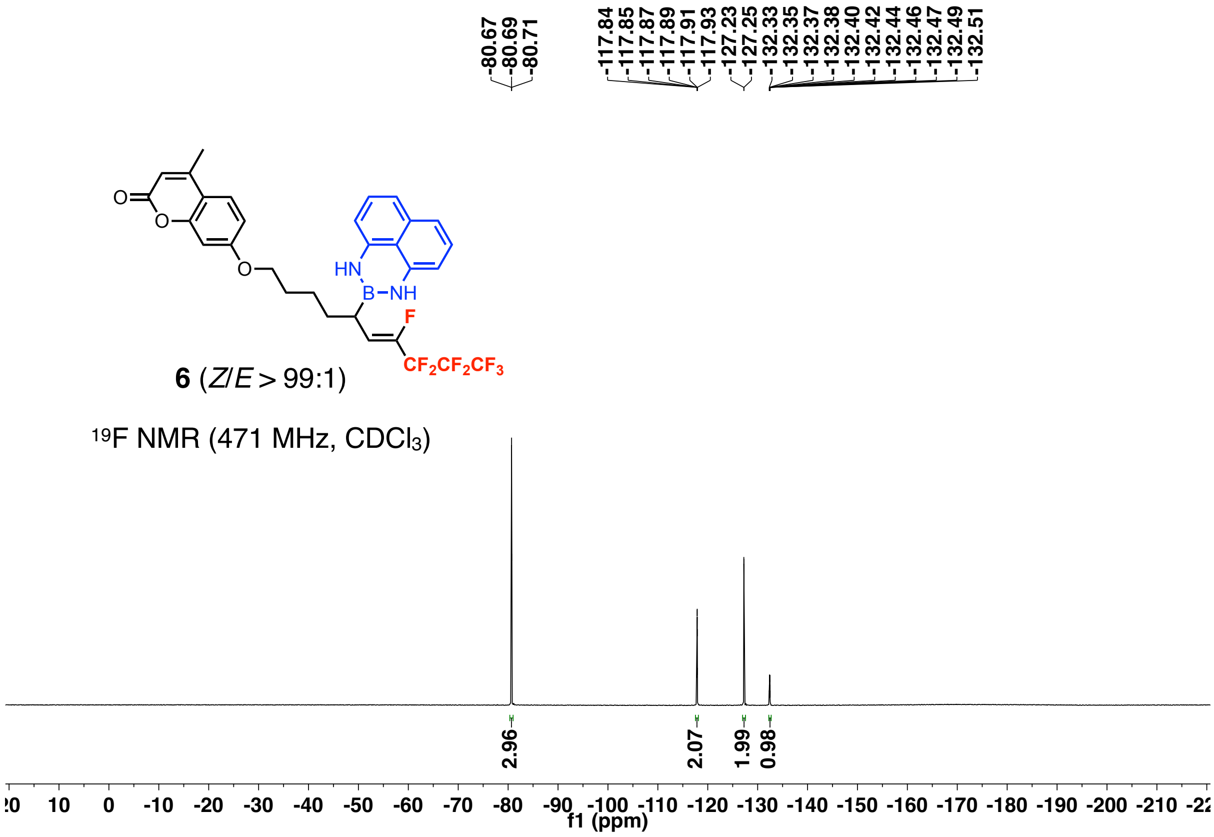
6 (Z/E > 99:1)
CF2CF2CF3
19F NMR (471 MHz, CDCl3)
f1 (ppm)

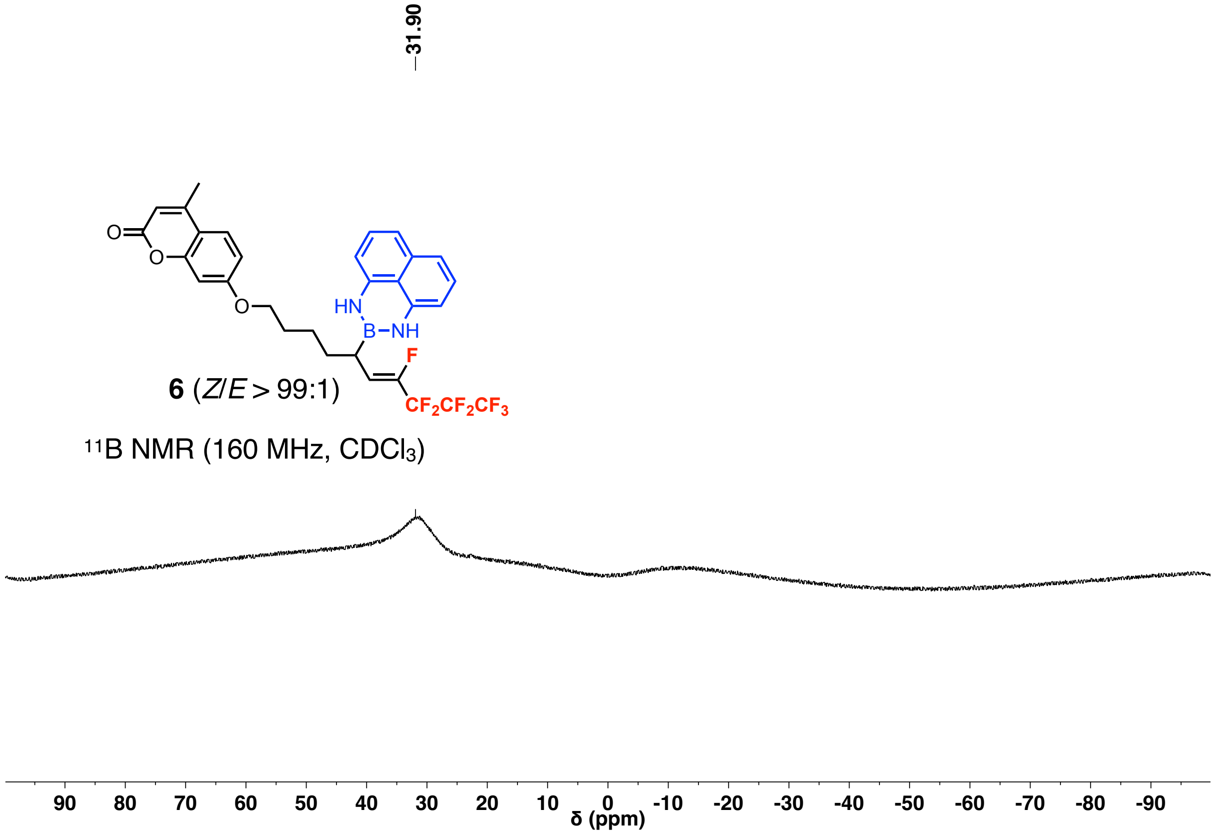

31.90
HN
B
NH
F
$CF_2CF_2CF_3$
6 (*Z*/*E* > 99:1)
^{11}B NMR (160 MHz, $CDCl_3$)
90 80 70 60 50 40 30 20 10 0 -10 -20 -30 -40 -50 -60 -70 -80 -90
δ (ppm)

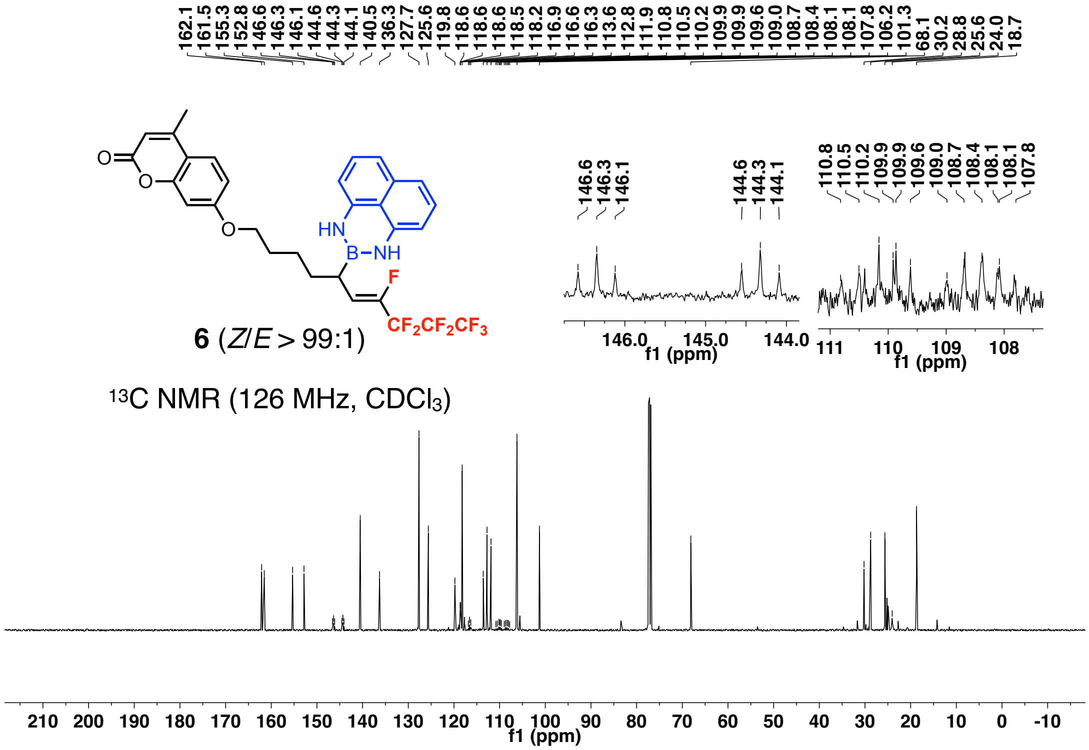

162.1 161.5 155.3 152.8 146.6 146.3 146.1 144.6 144.3 144.1 140.5 136.3 127.7 125.6 119.8 118.6 118.6 118.6 118.5 118.2 116.9 116.6 116.3 113.6 112.8 111.9 110.8 110.5 110.2 109.9 109.9 109.6 109.0 108.7 108.4 108.1 108.1 107.8 106.2 101.3 68.1 30.2 28.8 25.6 24.0 18.7
HN
B
NH
F
$CF_2CF_2CF_3$
6 (*Z*/*E* > 99:1)
146.6 146.3 146.1 144.6 144.3 144.1
146.0 145.0 144.0
f1 (ppm)
110.8 110.5 110.2 109.9 109.9 109.6 109.0 108.7 108.4 108.1 108.1 107.8
111 110 109 108
f1 (ppm)
^{13}C NMR (126 MHz, $CDCl_3$)
210 200 190 180 170 160 150 140 130 120 110 100 90 80 70 60 50 40 30 20 10 0 -10
f1 (ppm)

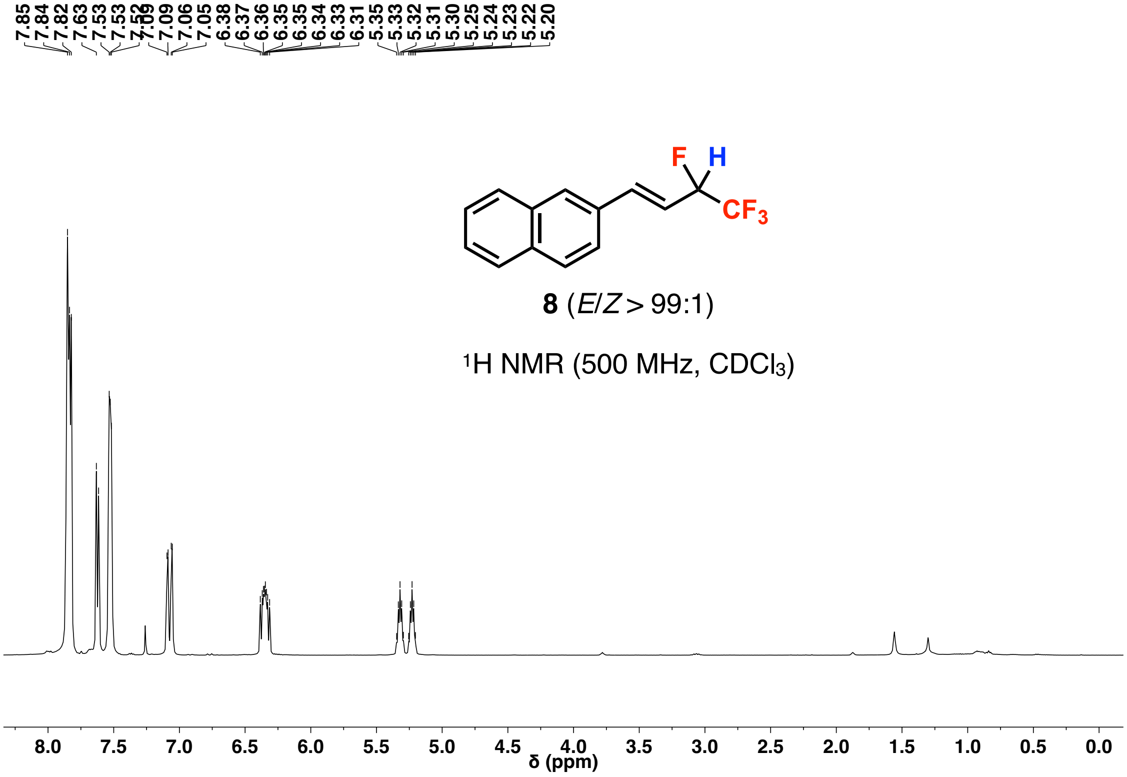

8 (E/Z > 99:1)
1H NMR (500 MHz, CDCl3)
8.0 7.5 7.0 6.5 6.0 5.5 5.0 4.5 4.0 3.5 3.0 2.5 2.0 1.5 1.0 0.5 0.0
δ (ppm)

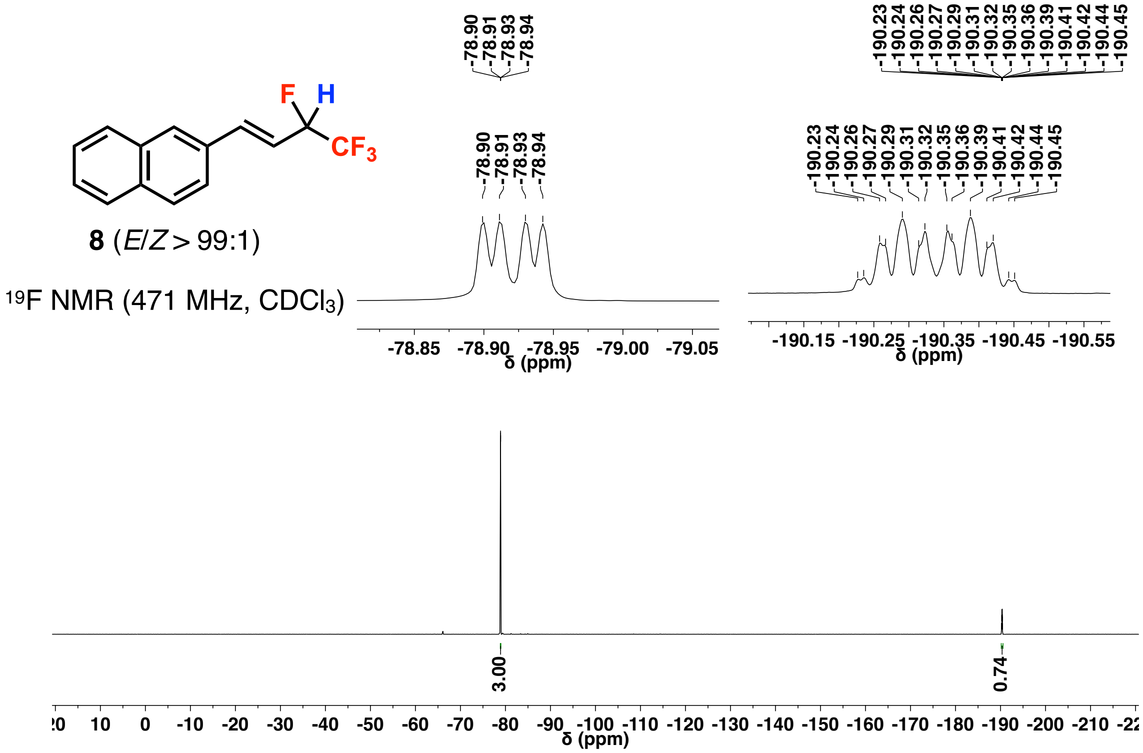

8 (E/Z > 99:1)
19F NMR (471 MHz, CDCl3)
-78.90 -78.91 -78.93 -78.94
-190.23 -190.24 -190.26 -190.27 -190.29 -190.31 -190.32 -190.35 -190.36 -190.39 -190.41 -190.42 -190.44 -190.45
3.00
0.74
δ (ppm)

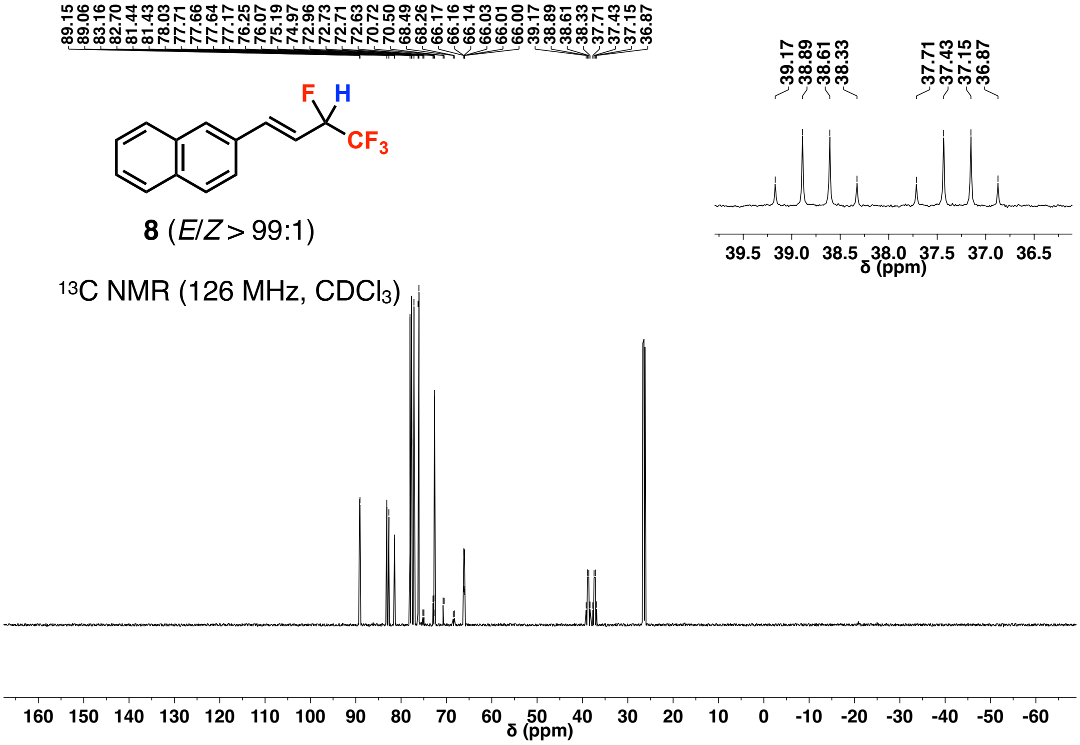
89.15
89.06
83.16
82.70
81.44
81.43
78.03
77.71
77.66
77.64
77.17
76.25
76.07
75.19
74.97
72.96
72.73
72.71
72.63
70.72
70.50
68.49
68.26
66.17
66.16
66.14
66.03
66.01
66.00
39.17
38.89
38.61
38.33
37.71
37.43
37.15
36.87
F
H
CF3
8 (E/Z > 99:1)
13C NMR (126 MHz, CDCl3)
δ (ppm)

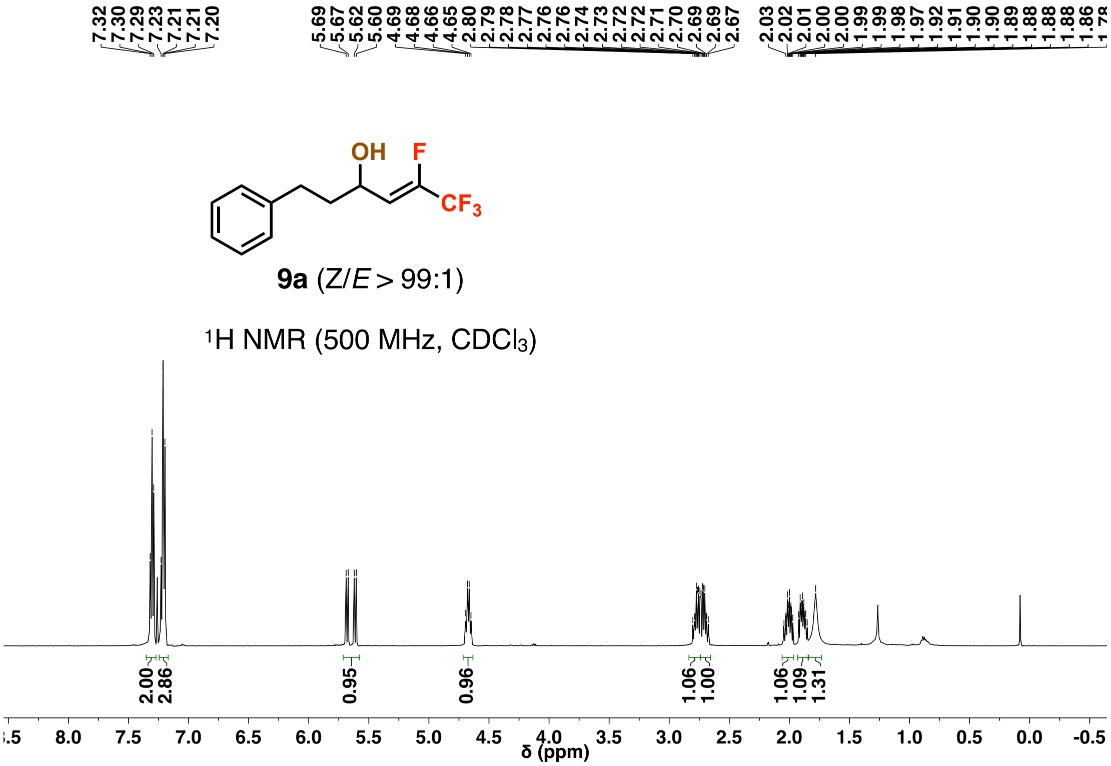
7.32
7.30
7.29
7.23
7.21
7.21
7.20
5.69
5.67
5.62
5.60
4.69
4.68
4.66
4.65
2.80
2.79
2.78
2.77
2.76
2.74
2.73
2.72
2.72
2.71
2.70
2.69
2.69
2.67
2.03
2.02
2.01
2.00
1.99
1.99
1.98
1.97
1.92
1.91
1.90
1.90
1.89
1.88
1.88
1.86
OH
F
CF3
9a (Z/E > 99:1)
1H NMR (500 MHz, CDCl3)
2.00
2.86
0.95
0.96
1.06
1.00
1.06
1.09
1.31
δ (ppm)

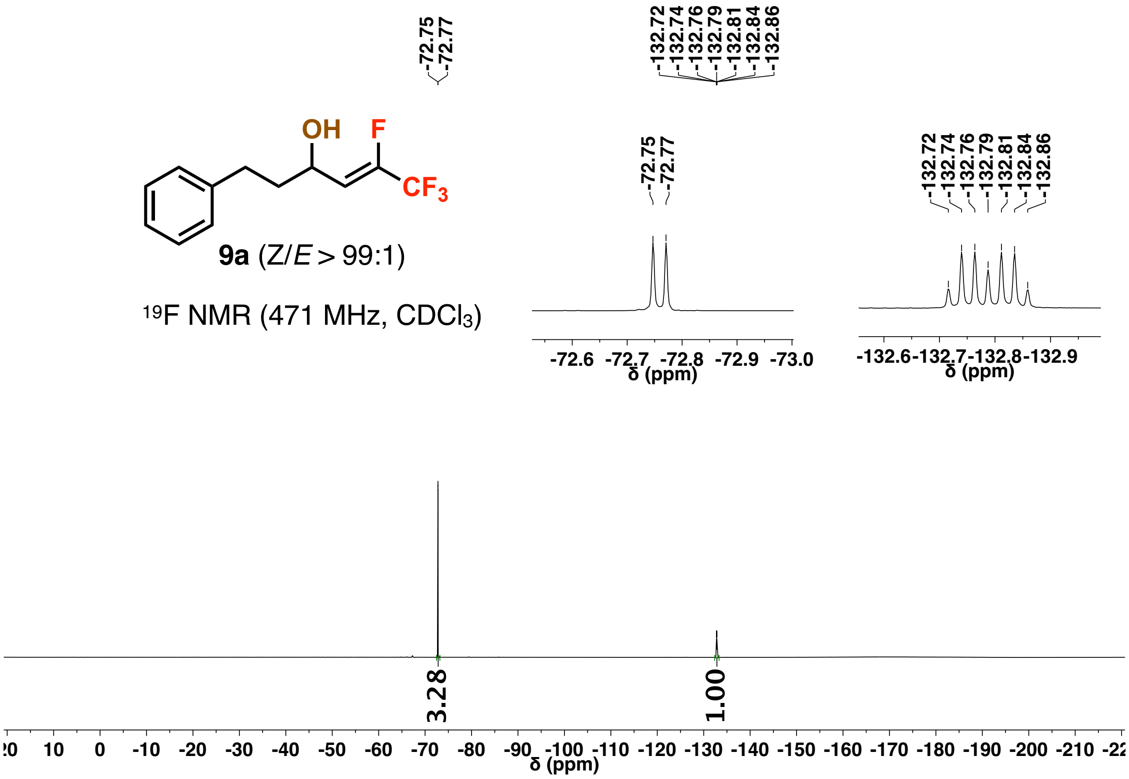

9a (Z/E > 99:1)
19F NMR (471 MHz, CDCl3)
3.28
1.00
δ (ppm)

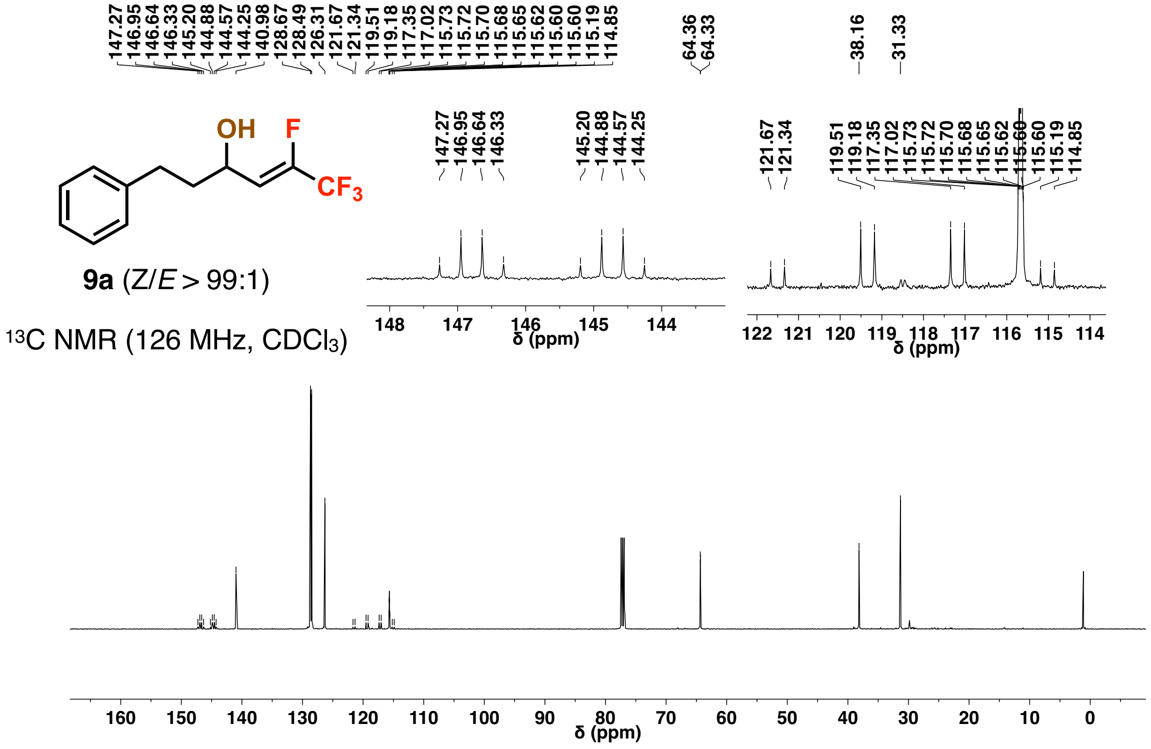

9a (Z/E > 99:1)
13C NMR (126 MHz, CDCl3)
δ (ppm)

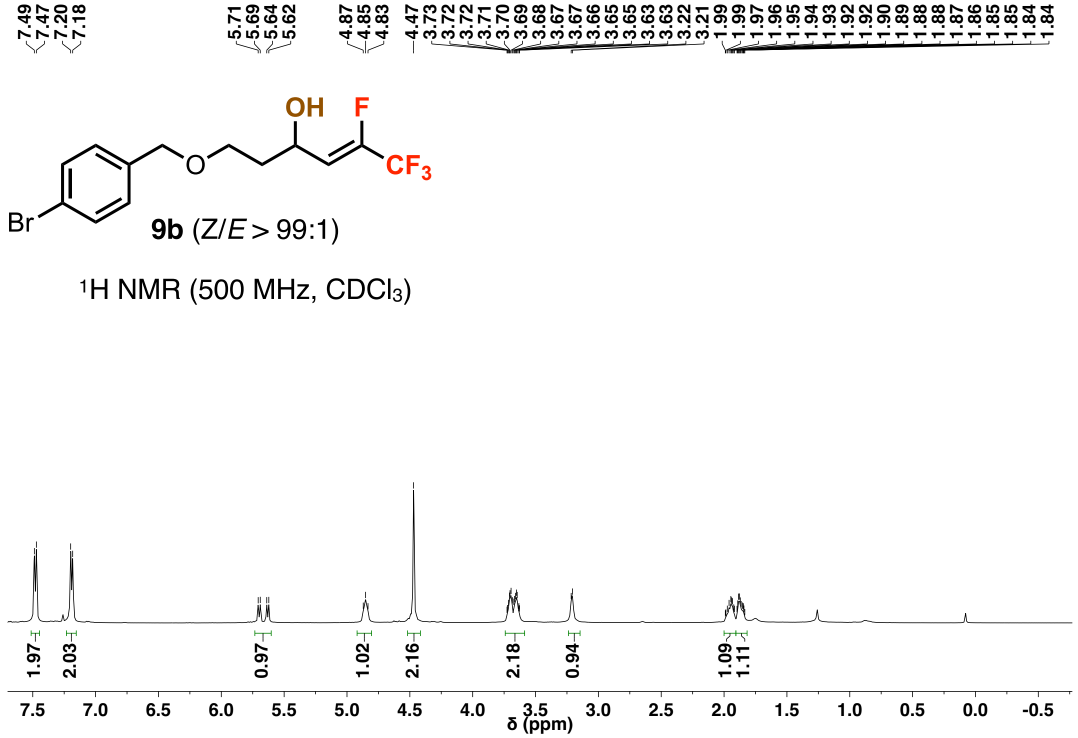

OH
F
O
CF3
Br
9b (Z/E > 99:1)
1H NMR (500 MHz, CDCl3)
δ (ppm)

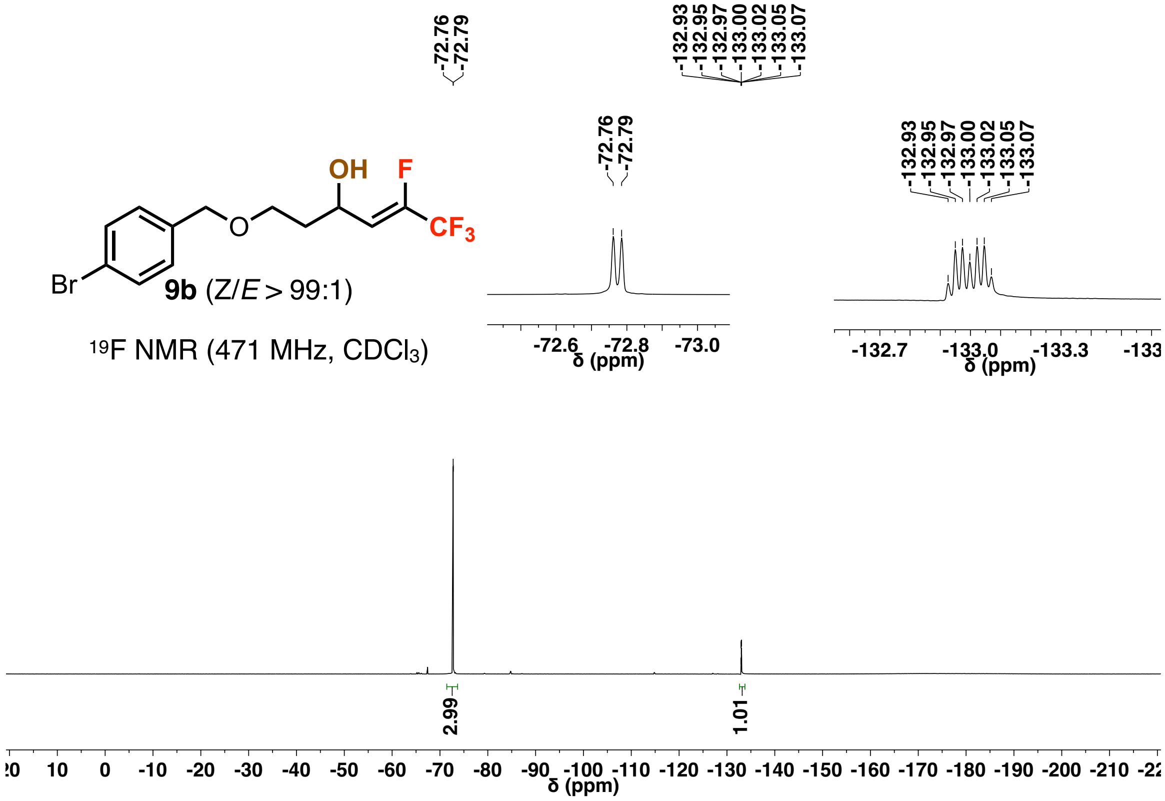

OH
F
O
CF3
Br
9b (Z/E > 99:1)
19F NMR (471 MHz, CDCl3)
δ (ppm)

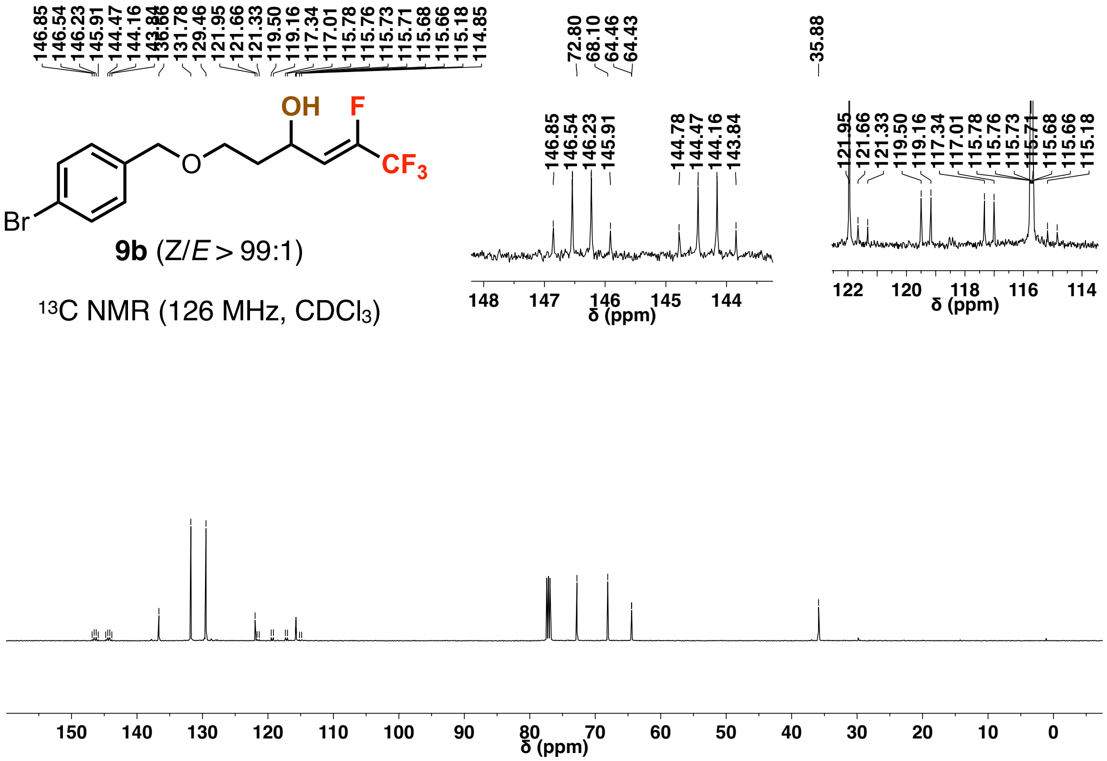
146.85
146.54
146.23
145.91
144.47
144.16
143.84
136.66
131.78
129.46
121.95
121.66
121.33
119.50
119.16
117.34
117.01
115.78
115.76
115.73
115.71
115.68
115.66
115.18
114.85
72.80
68.10
64.46
64.43
35.88
OH
F
O
CF3
Br
9b (Z/E > 99:1)
13C NMR (126 MHz, CDCl3)
146.85
146.54
146.23
145.91
144.78
144.47
144.16
143.84
148
147
146
145
144
δ (ppm)
121.95
121.66
121.33
119.50
119.16
117.34
117.01
115.78
115.76
115.73
115.71
115.68
115.66
115.18
122
120
118
116
114
δ (ppm)
150
140
130
120
110
100
90
80
70
60
50
40
30
20
10
0
δ (ppm)

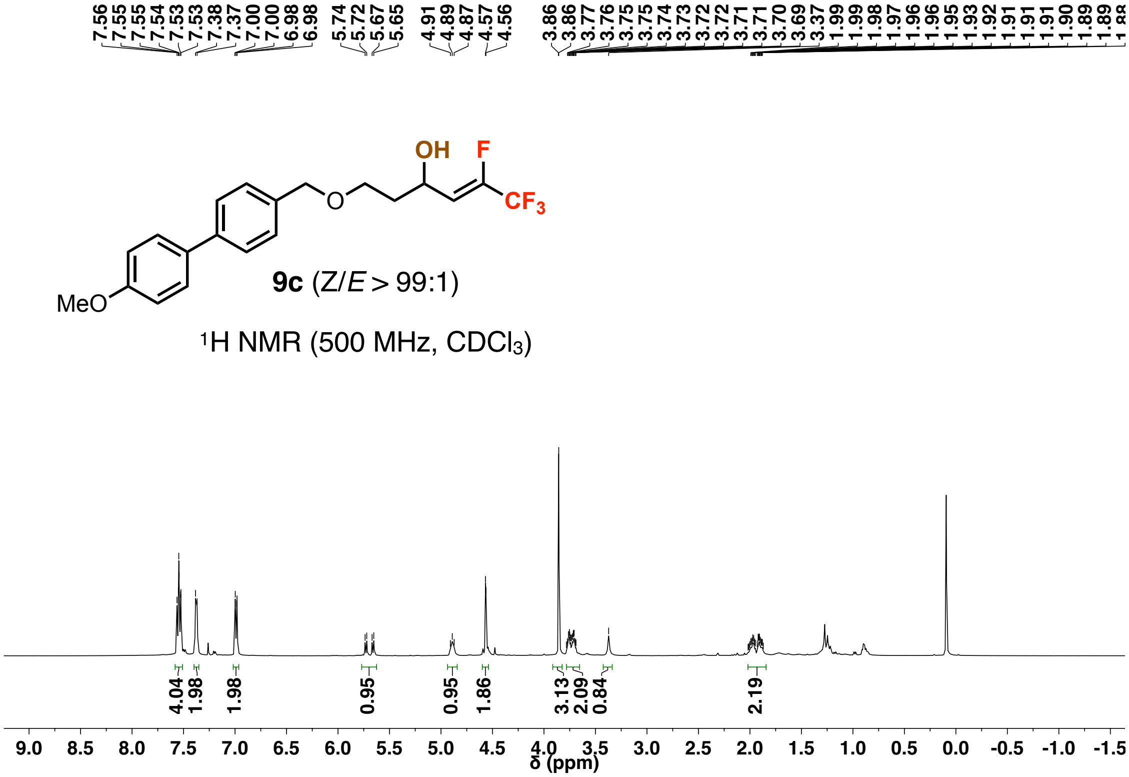
7.56
7.55
7.55
7.54
7.53
7.53
7.38
7.37
7.00
7.00
6.98
6.98
5.74
5.72
5.67
5.65
4.91
4.89
4.87
4.57
4.56
3.86
3.86
3.77
3.76
3.75
3.74
3.74
3.73
3.72
3.72
3.71
3.71
3.70
3.69
3.37
1.99
1.99
1.98
1.97
1.96
1.96
1.95
1.93
1.92
1.91
1.91
1.91
1.90
1.89
1.89
OH
F
O
CF3
MeO
9c (Z/E > 99:1)
1H NMR (500 MHz, CDCl3)
4.04
1.98
1.98
0.95
0.95
1.86
3.13
2.09
0.84
2.19
9.0
8.5
8.0
7.5
7.0
6.5
6.0
5.5
5.0
4.5
4.0
3.5
3.0
2.5
2.0
1.5
1.0
0.5
0.0
-0.5
-1.0
-1.5
δ (ppm)

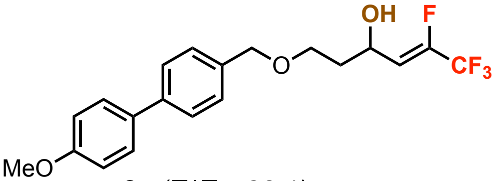

9c (Z/*E* > 99:1)

^{19}F NMR (471 MHz, CDCl$_3$)

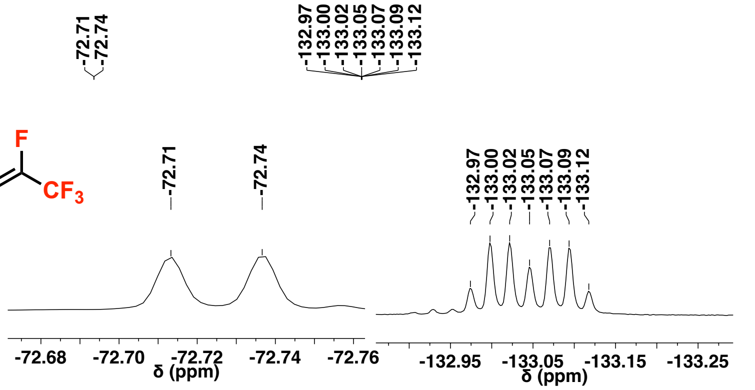

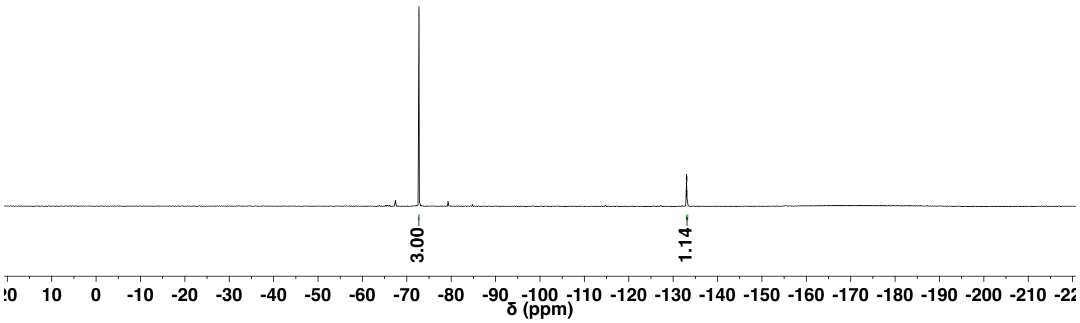

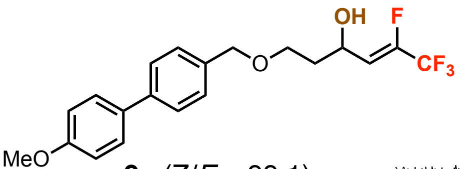

9c (Z/*E* > 99:1)

^{13}C NMR (126 MHz, CDCl$_3$)

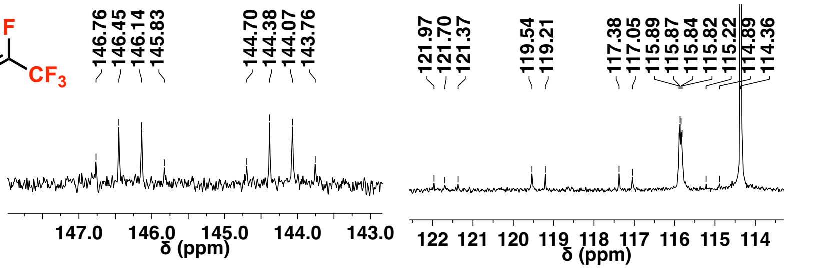

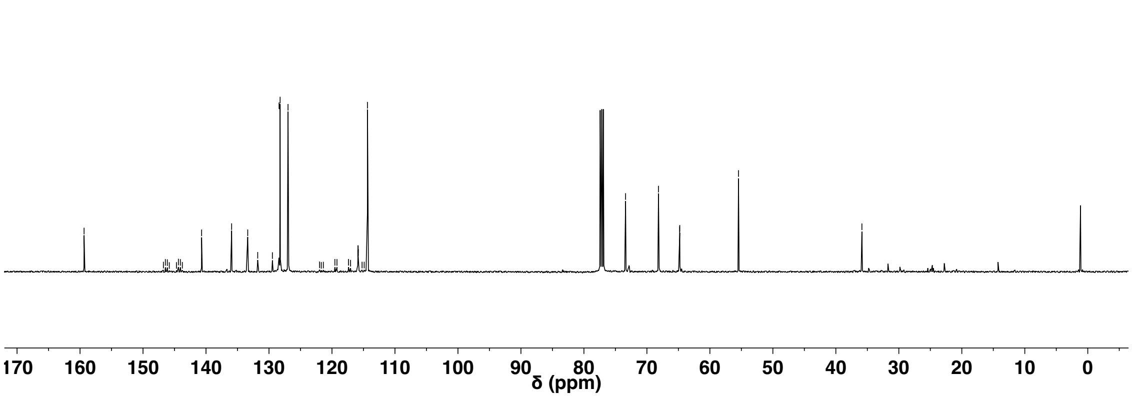

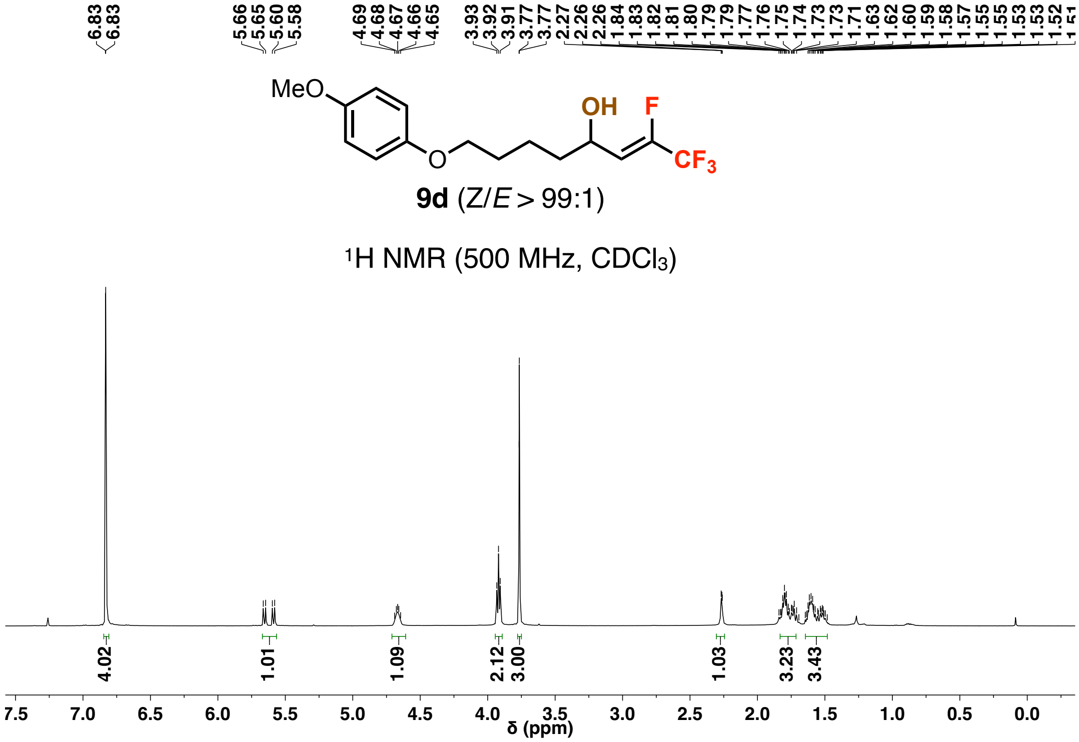
6.83
6.83
5.66
5.65
5.60
5.58
4.69
4.68
4.67
4.66
4.65
3.93
3.92
3.91
3.77
3.77
2.27
2.26
2.26
1.84
1.83
1.82
1.81
1.80
1.79
1.79
1.77
1.76
1.75
1.74
1.73
1.73
1.71
1.63
1.62
1.60
1.59
1.58
1.57
1.55
1.55
1.53
1.53
1.52
MeO
O
OH
F
CF3
9d (Z/E > 99:1)
1H NMR (500 MHz, CDCl3)
4.02
1.01
1.09
2.12
3.00
1.03
3.23
3.43
7.5
7.0
6.5
6.0
5.5
5.0
4.5
4.0
3.5
3.0
2.5
2.0
1.5
1.0
0.5
0.0
δ (ppm)

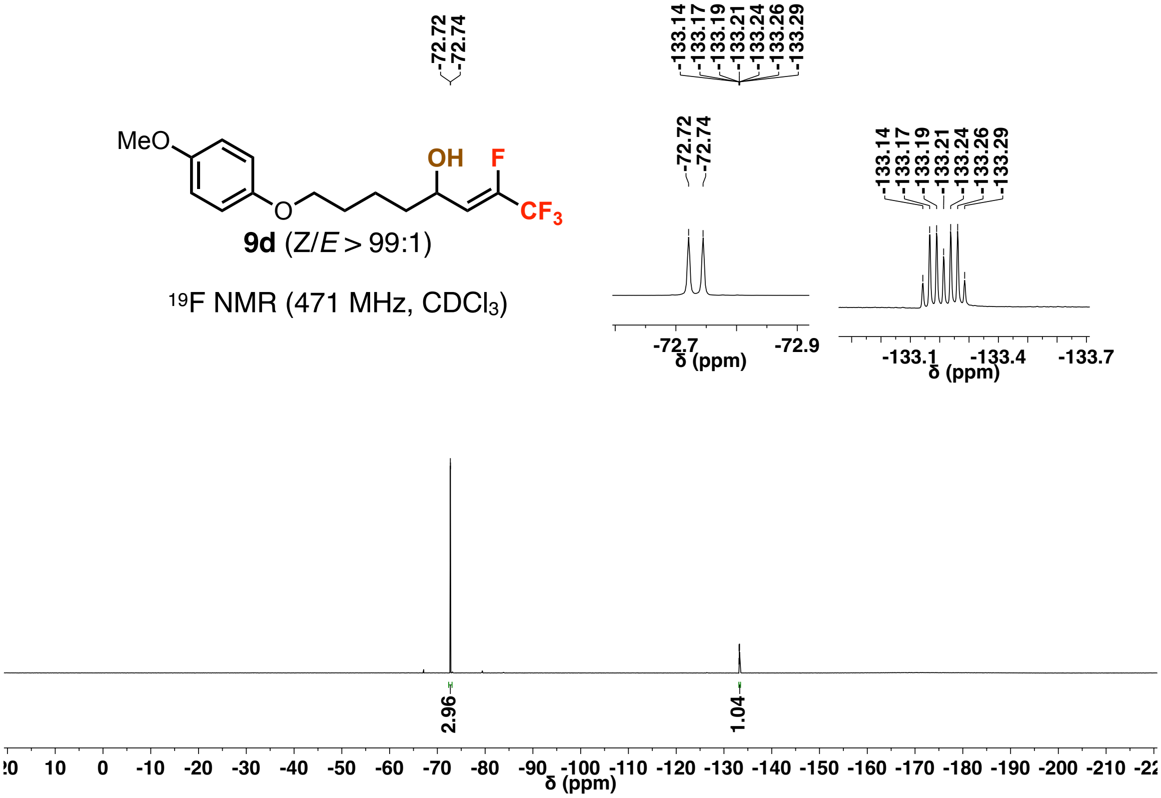
-72.72
-72.74
-133.14
-133.17
-133.19
-133.21
-133.24
-133.26
-133.29
MeO
O
OH
F
CF3
9d (Z/E > 99:1)
19F NMR (471 MHz, CDCl3)
-72.7
-72.9
δ (ppm)
-133.1
-133.4
-133.7
δ (ppm)
2.96
1.04
10
0
-10
-20
-30
-40
-50
-60
-70
-80
-90
-100
-110
-120
-130
-140
-150
-160
-170
-180
-190
-200
-210
δ (ppm)

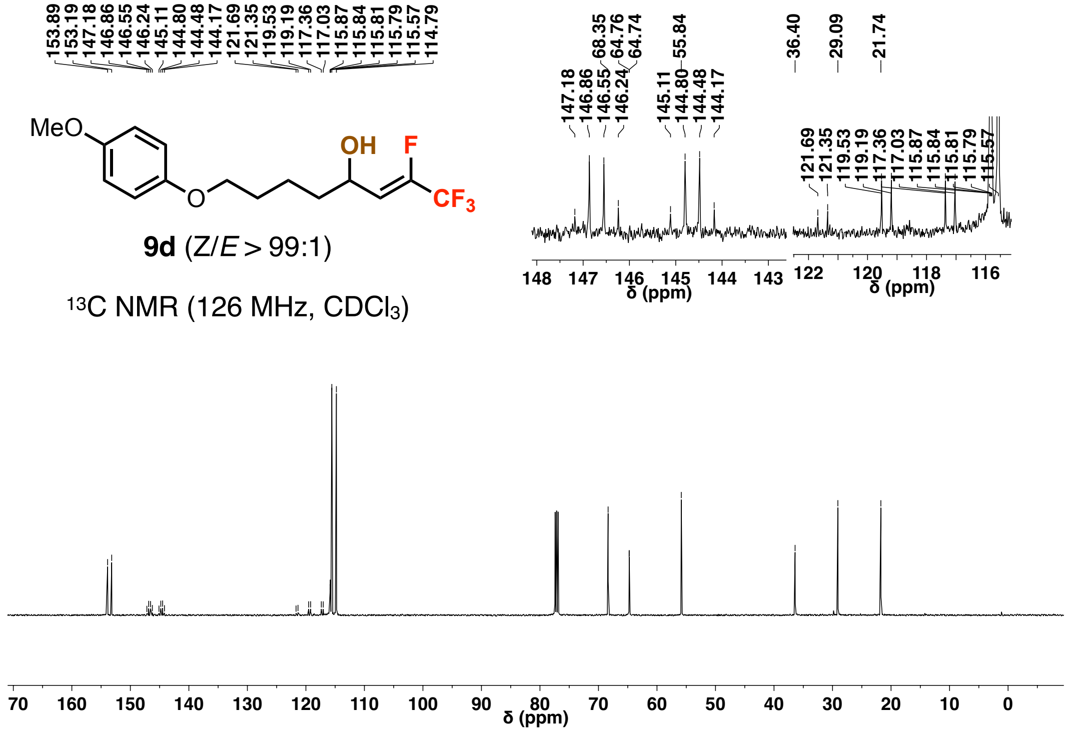

MeO
O
OH
F
CF_3
9d (Z/E > 99:1)
^{13}C NMR (126 MHz, $CDCl_3$)
δ (ppm)

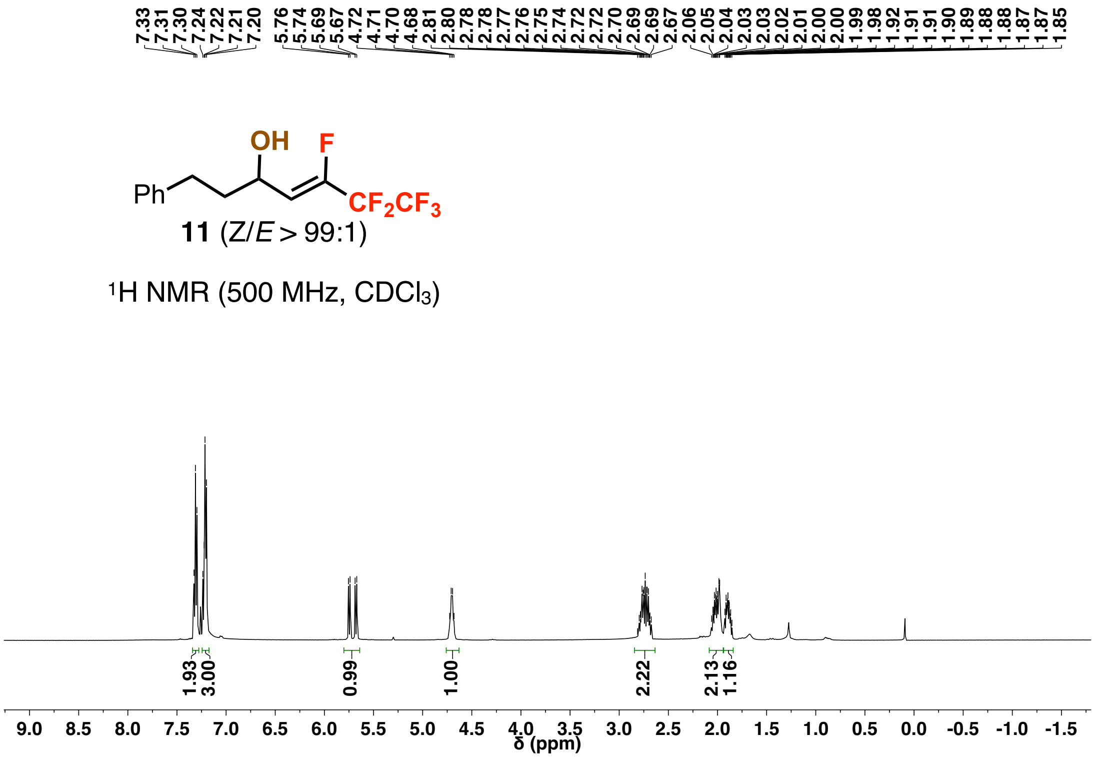

Ph
OH
F
CF_2CF_3
11 (Z/E > 99:1)
^{1}H NMR (500 MHz, $CDCl_3$)
δ (ppm)

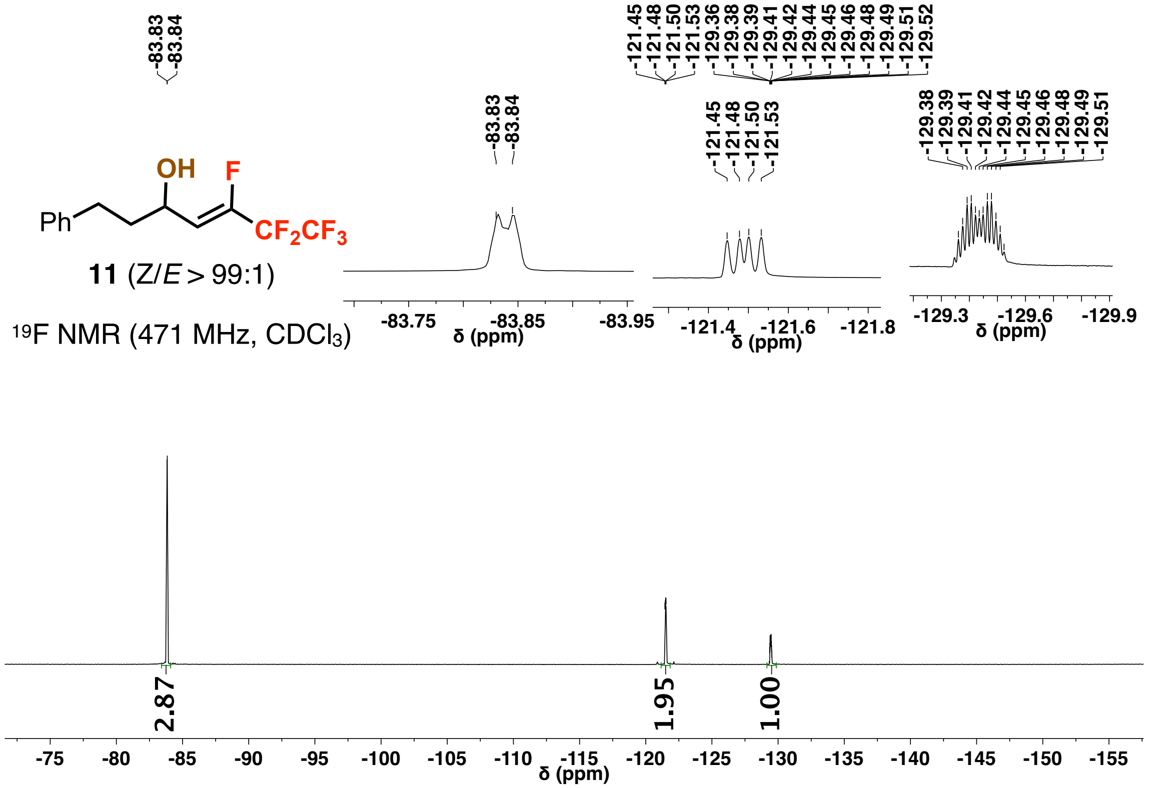
OH
F
Ph
CF2CF3
11 (Z/E > 99:1)
19F NMR (471 MHz, CDCl3)
2.87
1.95
1.00
δ (ppm)

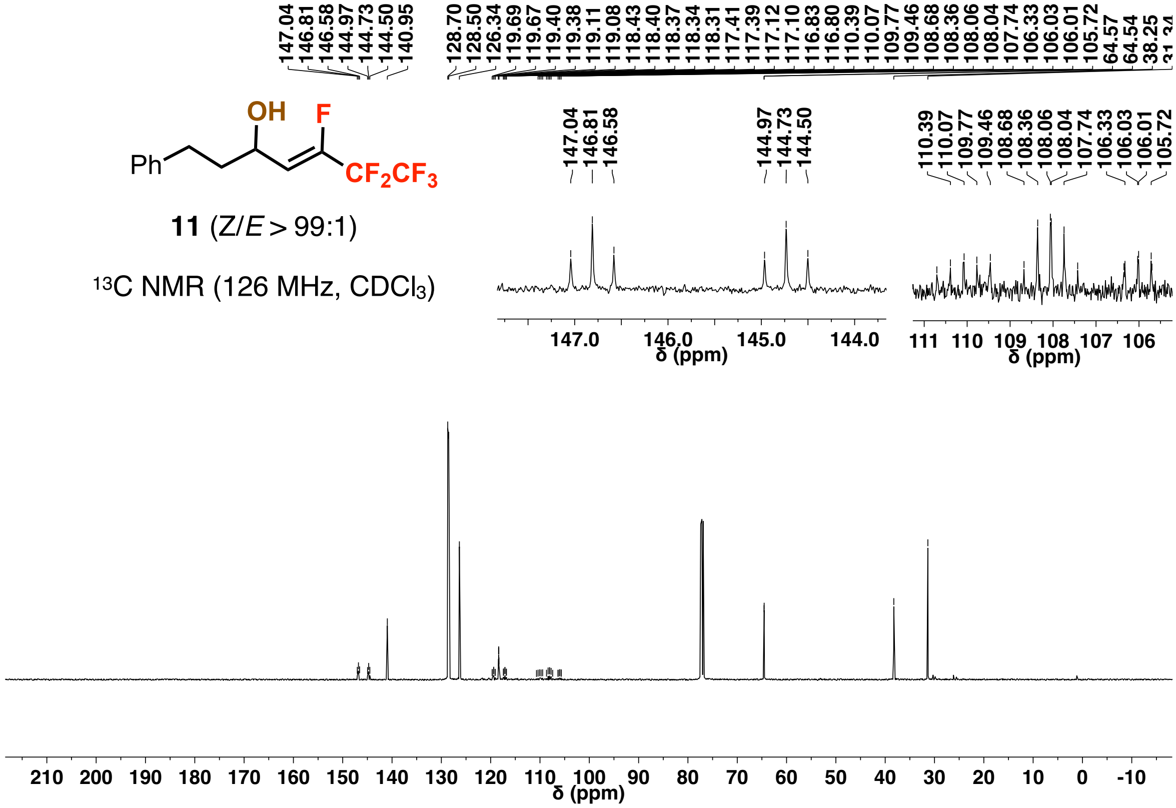
OH
F
Ph
CF2CF3
11 (Z/E > 99:1)
13C NMR (126 MHz, CDCl3)
δ (ppm)

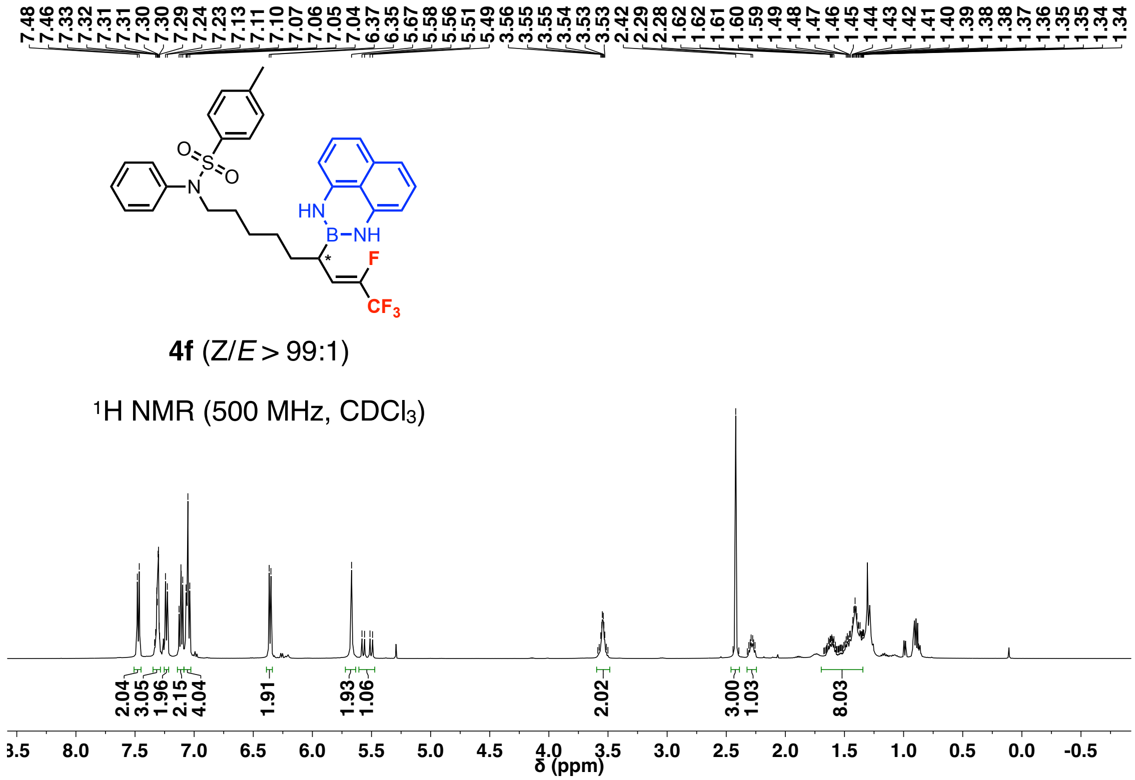
7.48
7.46
7.33
7.32
7.31
7.31
7.30
7.29
7.24
7.23
7.13
7.11
7.10
7.07
7.06
7.05
7.04
6.37
6.35
5.67
5.58
5.56
5.51
5.49
3.56
3.55
3.55
3.54
3.53
3.53
2.42
2.29
2.28
1.62
1.62
1.61
1.60
1.59
1.49
1.48
1.47
1.46
1.45
1.44
1.43
1.42
1.41
1.40
1.39
1.38
1.38
1.37
1.36
1.35
1.35
1.34
1.34
O
S
O
N
HN
B
NH
*
F
CF3
4f (Z/E > 99:1)
¹H NMR (500 MHz, CDCl3)
2.04
3.05
1.96
2.15
4.04
1.91
1.93
1.06
2.02
3.00
1.03
8.03
δ (ppm)

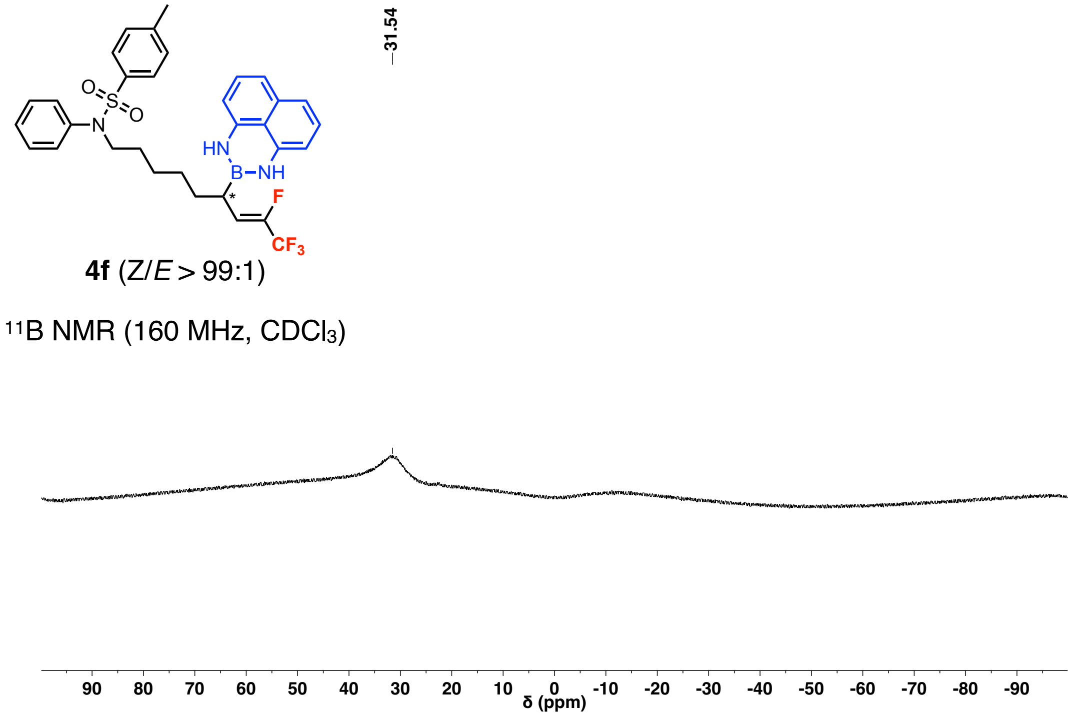
31.54
O
S
O
N
HN
B
NH
*
F
CF3
4f (Z/E > 99:1)
¹¹B NMR (160 MHz, CDCl3)
δ (ppm)

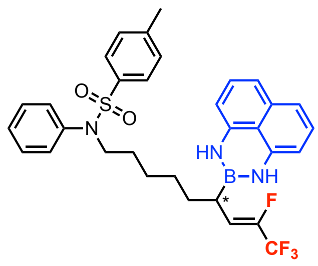

4f (Z/*E* > 99:1)

^{19}F NMR (471 MHz, $CDCl_3$)

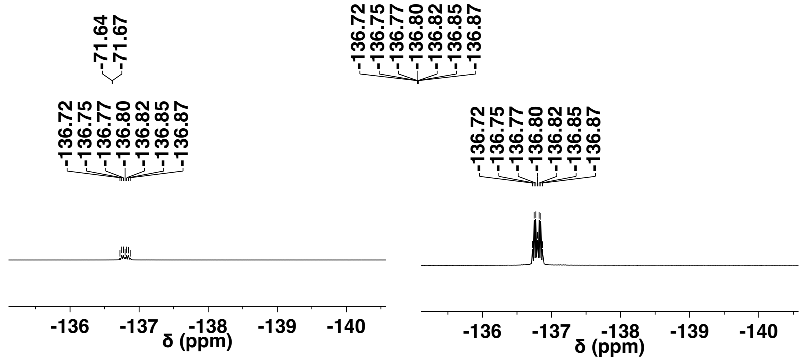

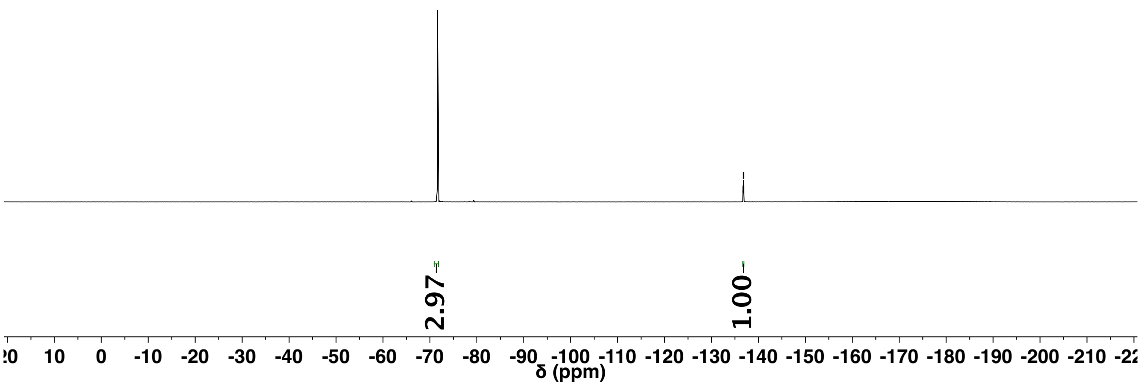

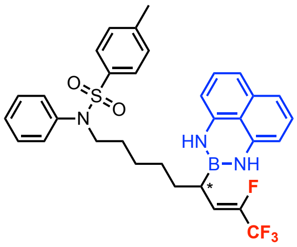

4f (Z/*E* > 99:1)

^{13}C NMR (126 MHz, $CDCl_3$)

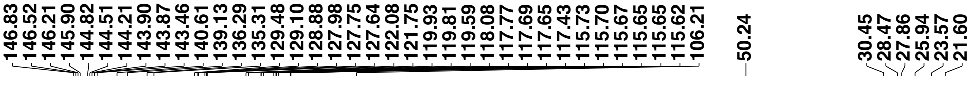

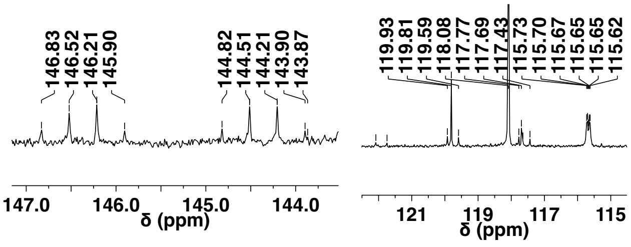

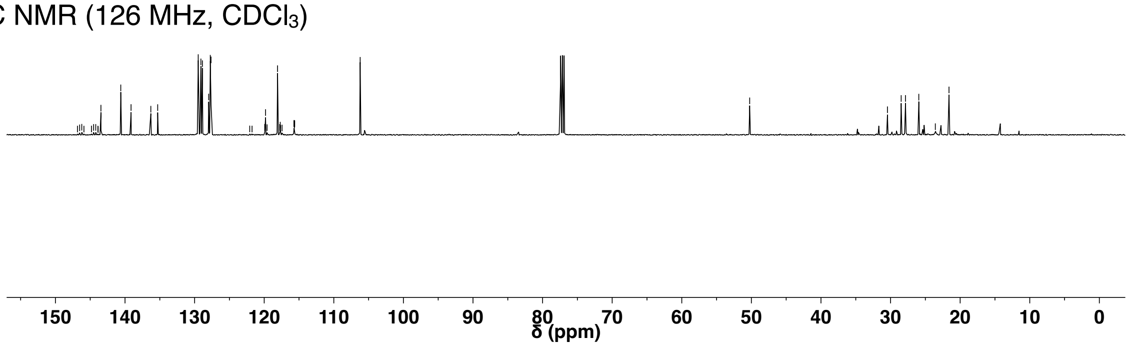

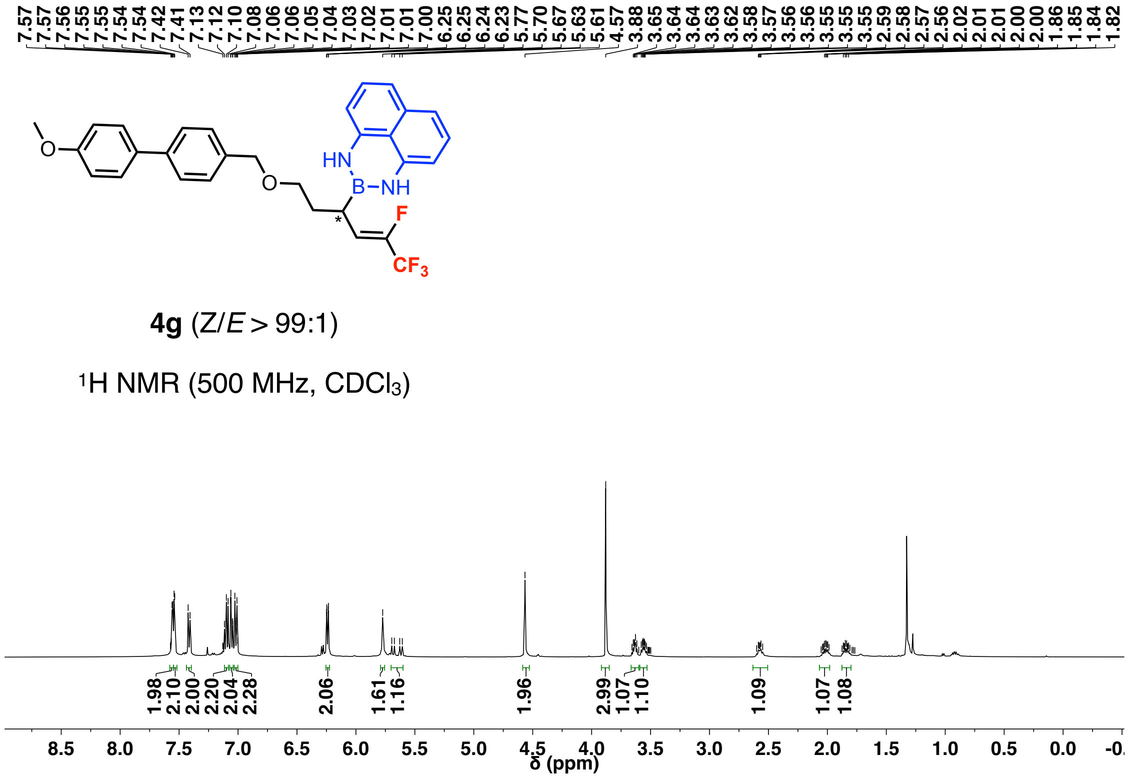

4g (Z/*E* > 99:1)

^{1}H NMR (500 MHz, $CDCl_3$)

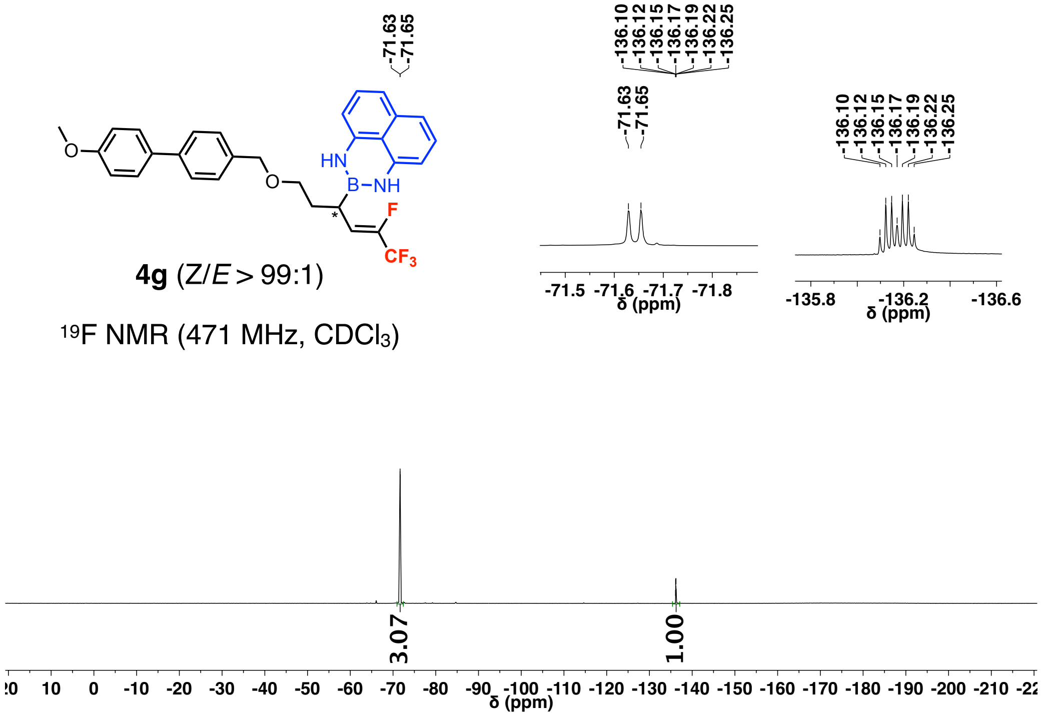

4g (Z/*E* > 99:1)

^{19}F NMR (471 MHz, $CDCl_3$)

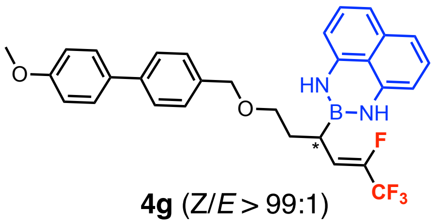

4g (*Z*/*E* > 99:1)

^{11}B NMR (160 MHz, $CDCl_3$)

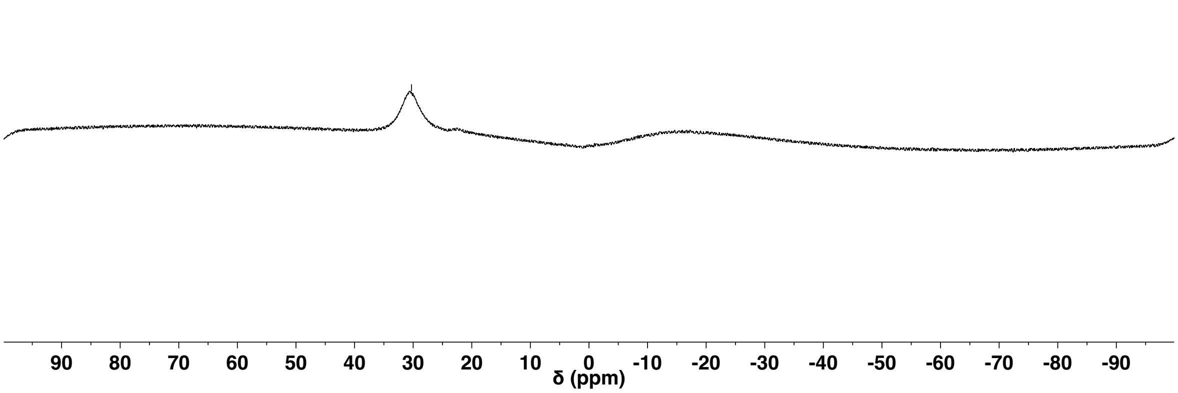

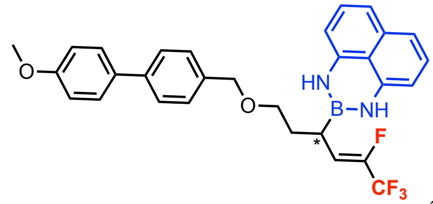

4g (*Z*/*E* > 99:1)

^{13}C NMR (126 MHz, $CDCl_3$)

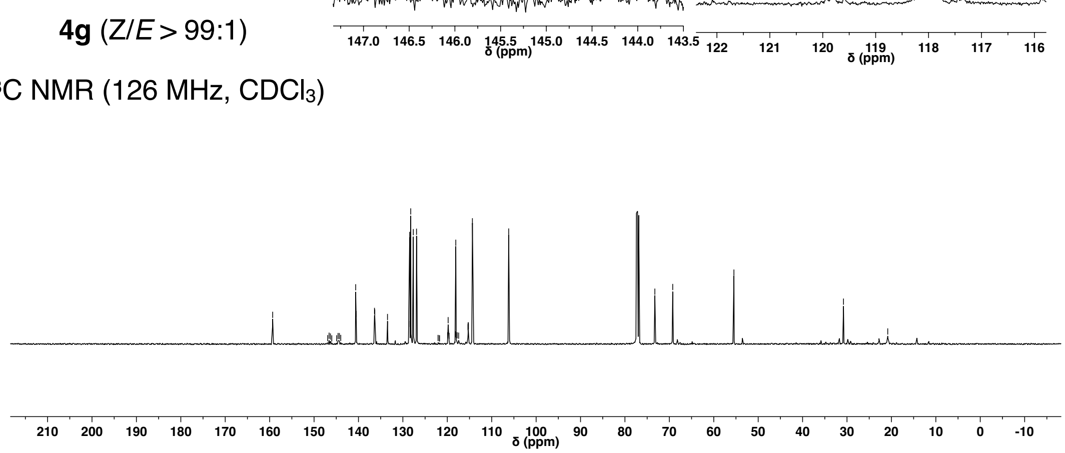

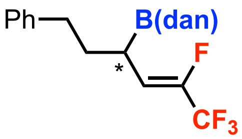

chiral-4a

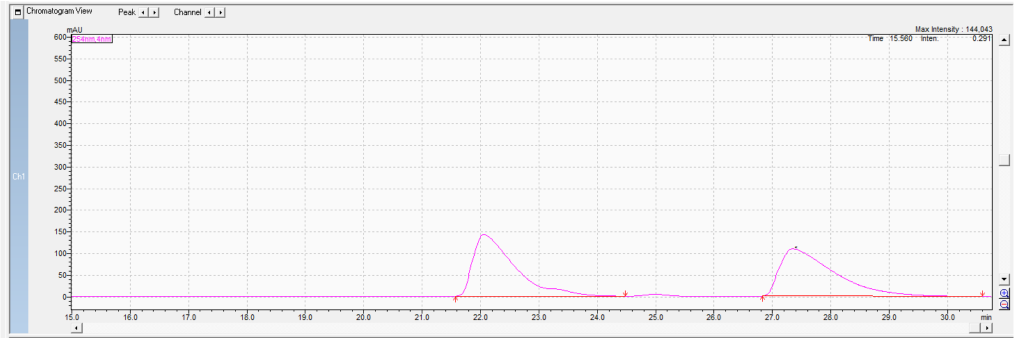

Results View - Peak Table

Peak Table | Compound | Group | Calibration Curve

Peak#	Ret. Time	Area	Height	Mark	Conc.	Unit	ID#	Name	Area%
1	22.058	7062198	142624	M	0.000				49.983
2	27.348	7066982	109370	M	0.000				50.017
Total		14129180	251994		0.000				100.000

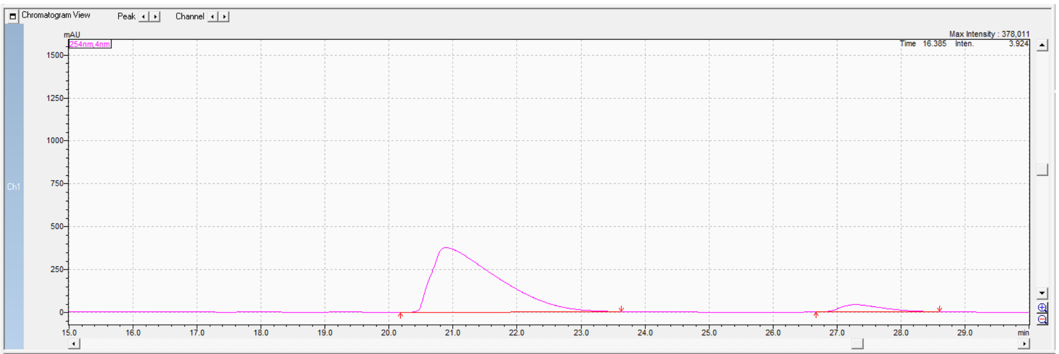

Results View - Peak Table

Peak Table | Compound | Group | Calibration Curve

Peak#	Ret. Time	Area	Height	Mark	Conc.	Unit	ID#	Name	Area%
1	20.891	26736713	374346	M	0.000				93.130
2	27.286	1972161	42474	M	0.000				6.870
Total		28708874	416820		0.000				100.000

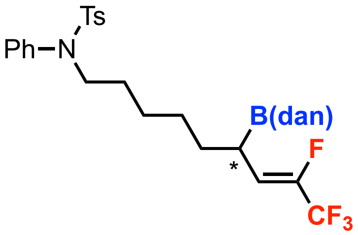

chiral-4f

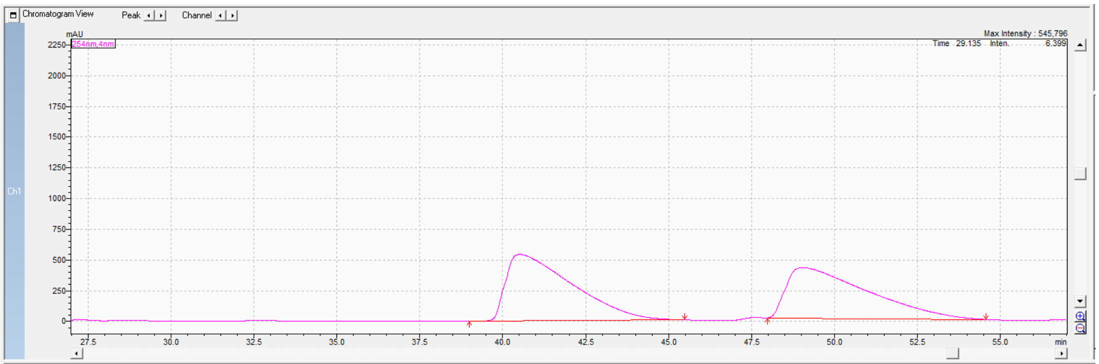

Results View - Peak Table

Peak Table | Compound | Group | Calibration Curve

Peak#	Ret. Time	Area	Height	Mark	Conc.	Unit	ID#	Name	Area%
1	40.519	75208591	539839	M	0.000				51.747
2	49.046	70131243	410221	M	0.000				48.253
Total		145339835	950061		0.000				100.000

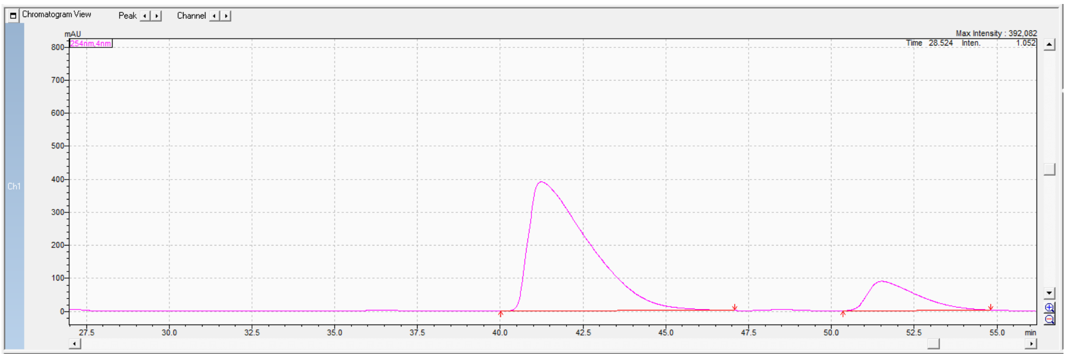

Results View - Peak Table

Peak Table | Compound | Group | Calibration Curve

Peak#	Ret. Time	Area	Height	Mark	Conc.	Unit	ID#	Name	Area%
1	41.239	49474861	391016	M	0.000				84.373
2	51.522	9163693	88781	M	0.000				15.627
Total		58638554	479797		0.000				100.000

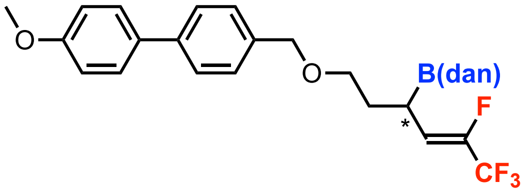

chiral-4g

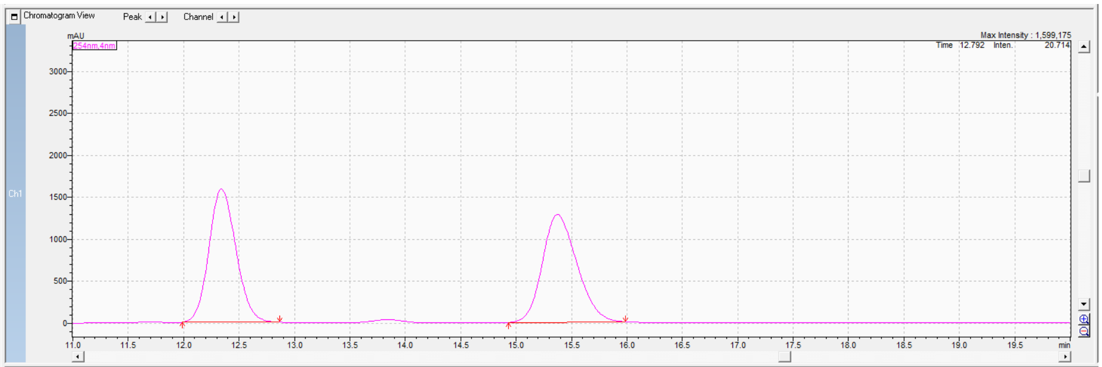

Results View - Peak Table

Peak Table | Compound | Group | Calibration Curve

Peak#	Ret. Time	Area	Height	Mark	Conc.	Unit	ID#	Name	Area%
1	12.341	26983705	1585439	M	0.000				49.230
2	15.373	27827400	1281200	M	0.000				50.770
Total		54811105	2866639		0.000				100.000

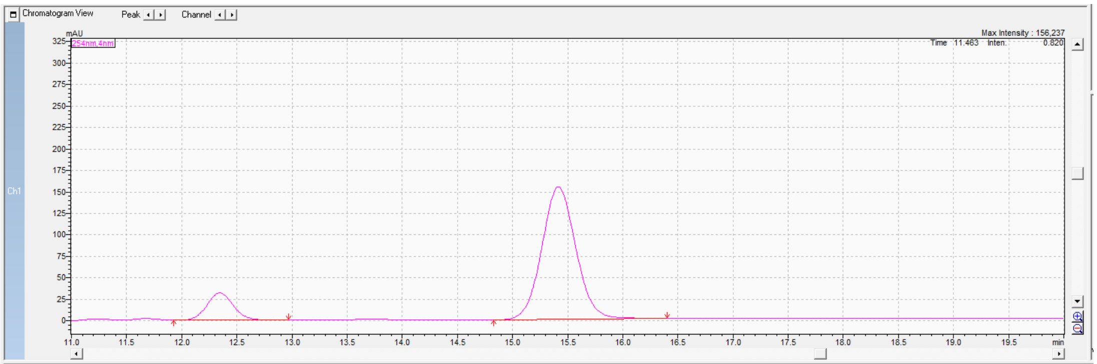

Results View - Peak Table

Peak Table | Compound | Group | Calibration Curve

Peak#	Ret. Time	Area	Height	Mark	Conc.	Unit	ID#	Name	Area%
1	12.345	524995	31611	M	0.000				13.674
2	15.416	3314345	154620	M	0.000				86.326
Total		3839341	186231		0.000				100.000

11. Coordinations of DFT calculations

Z-4a

C	1.55838100	1.38590600	-0.27332000
H	1.77352300	1.40478500	-1.35547600
C	2.73199500	0.75133700	0.41587500
H	2.92317800	0.98736600	1.46524500
C	3.53796000	-0.13962200	-0.15870200
F	3.37148700	-0.52461700	-1.43982600
C	1.32321300	2.83198400	0.19945300
H	0.98553600	2.83625900	1.25226400
H	2.28394200	3.37463700	0.19443400
C	0.31618600	3.61985300	-0.66151300
H	0.69157100	3.65813000	-1.69755900
H	0.28700300	4.65964200	-0.29707200
C	4.69600800	-0.84668800	0.48523500
F	4.51667900	-2.17684400	0.48288600
F	5.84115200	-0.60138800	-0.17194000
F	4.85294100	-0.45051500	1.75615400
C	-1.07520000	3.03306400	-0.64652800
C	-1.93369500	3.26871800	0.43900000
C	-1.50176500	2.15648300	-1.65313100
C	-3.16631300	2.62102900	0.53175100
H	-1.62222000	3.95968000	1.22848200
C	-2.73239400	1.49876700	-1.56052300
H	-0.85108200	1.96688300	-2.51027800
C	-3.56440500	1.72326100	-0.46351400
H	-3.81597500	2.80474400	1.39066100
H	-3.03174200	0.79389900	-2.33916900
H	-4.51292600	1.18988800	-0.37479600
C	-0.97578200	-1.54041100	-0.92687000

C	-1.59283800	-0.46394100	1.22984700
C	-1.84138500	-1.43307600	0.20801900
C	-1.23778400	-2.48647100	-1.90988700
C	-2.44757200	-0.37231500	2.32010900
C	-2.97929200	-2.29528500	0.31340300
C	-2.35616700	-3.33869000	-1.79343000
H	-0.57282600	-2.56365800	-2.77376400
C	-3.56802100	-1.22338700	2.41586400
H	-2.25458000	0.37520800	3.09325000
C	-3.21165700	-3.25094300	-0.71428400
C	-3.83696500	-2.16221000	1.44078000
H	-2.54230100	-4.07725700	-2.57724900
H	-4.23102500	-1.13080300	3.27986600
B	0.34078000	0.37028500	-0.06649800
N	-0.49868200	0.38336900	1.07715700
N	0.09963000	-0.66211200	-1.01286700
H	-4.70651500	-2.81797400	1.52231100
H	-4.07665700	-3.91278500	-0.63332100
H	0.69478700	-0.77121200	-1.82496800
H	-0.42761900	1.11843300	1.76832700

INT2

C	-1.93724000	1.12993100	1.55184000
C	-2.83241200	1.02029400	0.27670100
H	-3.80682600	0.57415800	0.54213200
Cu	-0.02487400	0.95475000	1.08176200
C	-2.29178000	0.10498400	2.57036400
C	-1.53836000	0.22766600	3.91618700
F	-3.63043300	0.09969500	2.91591400
F	-1.65401500	1.46063500	4.42272900

F	-2.01176100	-0.63920000	4.81471000
F	-0.22317400	-0.03134400	3.76613800
F	-2.05102400	-1.19219500	2.13876700
H	-2.12164800	2.10141800	2.04139200
C	-3.12206900	2.41964900	-0.28213700
H	-3.66712900	2.99831000	0.48408100
H	-2.17097300	2.96647100	-0.43361300
C	-3.92498600	2.43671700	-1.59825900
H	-4.18770400	3.48007300	-1.84140100
H	-3.29337200	2.07051500	-2.42248600
C	-5.17721500	1.59423200	-1.53614900
C	-6.29748700	2.02605700	-0.80987500
C	-5.22651600	0.33344300	-2.14826600
C	-7.43199900	1.22068700	-0.69388400
H	-6.27708400	3.00629900	-0.32490600
C	-6.35949900	-0.47702700	-2.03488700
H	-4.36288200	-0.01960800	-2.71734000
C	-7.46626700	-0.03665700	-1.30522600
H	-8.29431300	1.57508100	-0.12345200
H	-6.37580100	-1.45681000	-2.51896800
H	-8.35313900	-0.66844100	-1.21420000
C	-0.34801200	-0.25778800	-2.49569500
H	-0.95072100	1.55131900	-1.77596300
C	-1.51548900	-2.21956200	-1.51198000
H	-2.87186200	-1.76486400	-0.05414600
C	-0.53424600	-1.67359900	-2.40113500
C	0.61567400	0.25301600	-3.35936400
C	-1.68101600	-3.59903800	-1.43386200
C	0.28218900	-2.55097300	-3.18556400
C	1.41977800	-0.61744500	-4.12391000

H	0.73556300	1.33477000	-3.44791500
C	-0.87470500	-4.45526100	-2.21070900
H	-2.43174100	-4.01099700	-0.75537400
C	1.26636700	-1.98660500	-4.04423900
C	0.08826900	-3.95508900	-3.06506700
H	2.17457100	-0.19204900	-4.79027200
H	-1.01859400	-5.53569100	-2.12804200
H	1.89332900	-2.65408900	-4.63924700
H	0.71019100	-4.62715000	-3.66021400
N	-1.14316200	0.55995000	-1.70566100
N	-2.25719200	-1.34139000	-0.73886900
B	-2.08846300	0.07243600	-0.74498400
C	3.67493800	-0.61561300	0.16667900
C	3.90801800	0.72447900	-0.23395000
C	1.79592600	0.57247500	0.55808100
N	2.72271300	1.41779500	0.02828600
N	2.36400700	-0.65895900	0.64424500
C	2.36057700	2.84234900	-0.13786600
C	3.09329400	3.79502200	0.82464800
C	2.35315300	3.34587300	-1.59367100
H	1.31101200	2.82236400	0.18751300
C	4.40359000	4.38749700	0.29743200
H	3.23839900	3.28730700	1.79077200
H	2.39697800	4.62970800	1.01732300
C	3.64999400	3.98903100	-2.09886000
H	2.03586000	2.52412100	-2.25181200
H	1.55807600	4.11033500	-1.64245200
C	4.21580700	5.00096700	-1.09535500
H	5.19017800	3.61748300	0.27166500
H	4.76025400	5.15549300	1.00275200

H	3.44823400	4.48602500	-3.06167100
H	4.40040600	3.21545100	-2.32048500
H	5.16874200	5.41230900	-1.46581100
H	3.51889800	5.85559700	-1.01639600
C	1.57295500	-1.76892200	1.23447500
C	1.35995700	-2.98416900	0.31569900
C	2.01638400	-2.14975700	2.65766400
H	0.58961500	-1.29230600	1.33948600
C	2.33502600	-4.15668500	0.47020600
H	1.30871100	-2.64994700	-0.72841600
H	0.35119500	-3.35601300	0.55309100
C	3.05706600	-3.26953900	2.73763000
H	2.34915700	-1.24377600	3.18664500
H	1.10507500	-2.48618300	3.18005000
C	2.60434000	-4.49834500	1.93990500
H	3.28432100	-3.94755200	-0.04647300
H	1.90936800	-5.02821400	-0.05241800
H	3.21555100	-3.54050100	3.79421400
H	4.03349500	-2.91543400	2.36832200
H	3.35261600	-5.30439600	2.01446400
H	1.67652000	-4.89171700	2.39434000
C	5.14606700	1.10262300	-0.76450700
C	4.67814700	-1.58196500	0.04268200
C	6.13760100	0.13095200	-0.88795600
H	5.34354300	2.12319600	-1.07351100
C	5.90670900	-1.19498800	-0.48852100
H	4.51276300	-2.60856100	0.35046600
H	7.10884200	0.41098900	-1.30092000
H	6.69988700	-1.93801700	-0.59314100

NHC-Cu-F

Cu	-0.00053600	2.68486100	-0.00098100
C	-0.70882600	-1.34591000	-0.00136500
C	0.70938900	-1.34578900	-0.00130700
C	0.00011300	0.80336600	-0.00151400
N	1.09975500	-0.00472000	-0.00136700
N	-1.09942200	-0.00496600	-0.00144200
C	2.44023400	0.62493400	-0.00058200
C	3.25613300	0.39339000	1.28495300
C	3.25866200	0.39224100	-1.28426600
H	2.16415400	1.68849200	-0.00128800
C	4.19738100	-0.81588500	1.27485400
H	2.57140400	0.36456700	2.14675100
H	3.87251600	1.29946000	1.41544400
C	4.20059800	-0.81658400	-1.27104500
H	2.57561100	0.36202100	-2.14733700
H	3.87481200	1.29846100	-1.41472000
C	5.05378200	-0.85252400	0.00301900
H	3.62546700	-1.75111900	1.37543400
H	4.84711900	-0.77012500	2.16393900
H	4.85266300	-0.77072900	-2.15840900
H	3.62954400	-1.75220500	-1.37289900
H	5.70216300	-1.74379500	0.00405600
H	5.72952800	0.02237200	0.00365700
C	-2.44002300	0.62437500	-0.00073900
C	-3.25859300	0.39145000	-1.28433900
C	-3.25581600	0.39277300	1.28493100
H	-2.16426700	1.68801600	-0.00165300
C	-4.20050200	-0.81740700	-1.27095200
H	-2.57563100	0.36123400	-2.14747500

H	-3.87484900	1.29760000	-1.41473200
C	-4.19715700	-0.81648000	1.27488900
H	-2.57089600	0.36376300	2.14656300
H	-3.87215700	1.29881600	1.41571900
C	-5.05365600	-0.85310100	0.00312000
H	-3.62944200	-1.75304200	-1.37258400
H	-4.85252400	-0.77174200	-2.15836000
H	-4.84684300	-0.77060900	2.16400800
H	-3.62533900	-1.75177800	1.37546300
H	-5.70217100	-1.74427600	0.00428500
H	-5.72927900	0.02189700	0.00373300
C	1.42039700	-2.55053200	-0.00137100
C	-1.41970600	-2.55072300	-0.00136400
C	0.70196700	-3.74519500	-0.00137700
H	2.50452100	-2.56794500	-0.00140500
C	-0.70114700	-3.74529100	-0.00134800
H	-2.50383000	-2.56822300	-0.00127000
H	1.24439100	-4.69280100	-0.00138700
H	-1.24345100	-4.69296800	-0.00128200
F	-0.00104000	4.44876100	-0.00014700

TS2E

C	-2.19235900	1.49030500	1.27196300
C	-1.64847000	2.36992400	0.13238600
H	-2.21399800	2.15115700	-0.78535600
Cu	-1.23087900	-0.41082300	1.16854800
C	-3.44936800	0.88952000	1.27539000
F	-3.30944400	-0.91952200	1.07552900
H	-1.87568000	1.78193300	2.28162900
C	-1.86365800	3.86164300	0.46096600

H	-2.92974100	4.01085500	0.69702400
H	-1.31325600	4.11878000	1.38508400
C	-1.45708300	4.84583000	-0.65309700
H	-2.00781800	4.59308200	-1.57428400
H	-1.78342000	5.85726400	-0.35852000
C	0.02726300	4.85273200	-0.93232800
C	0.56213900	4.19796400	-2.04906800
C	0.91798000	5.43200900	-0.01362000
C	1.94463300	4.08246300	-2.22461800
H	-0.11484100	3.74669000	-2.77816300
C	2.29833800	5.32658600	-0.18705000
H	0.51929800	5.95981700	0.85813100
C	2.81708900	4.63843600	-1.28878400
H	2.33905600	3.53817400	-3.08551400
H	2.97591300	5.77320500	0.54489500
H	3.89673800	4.52878900	-1.41040800
C	2.22745000	1.78265200	0.53302900
H	0.79551500	2.85162500	1.51109900
C	1.45886200	0.64574500	-1.54081200
H	-0.52783900	0.86813400	-1.95736400
C	2.49871500	0.96933400	-0.61184200
C	3.25274300	2.09023600	1.42050100
C	1.75309200	-0.12467500	-2.65994400
C	3.82623700	0.48250800	-0.83689700
C	4.55489300	1.59769900	1.19916500
H	3.03891700	2.71610000	2.28985100
C	3.06269500	-0.59815500	-2.87511600
H	0.96006400	-0.37025200	-3.36818800
C	4.84434400	0.80928500	0.10267000
C	4.07973400	-0.30977700	-1.98944900

H	5.34458200	1.84971000	1.91168900
H	3.26365700	-1.21586500	-3.75310700
H	5.85690700	0.43588900	-0.06513900
H	5.08888800	-0.69154100	-2.15646300
N	0.92925700	2.22725600	0.72648100
N	0.18215900	1.12167900	-1.28215200
B	-0.15433900	1.92207900	-0.14902200
C	1.04790600	-3.00273800	-1.00151600
C	2.04668400	-2.66340500	-0.06568300
C	0.12958500	-1.63937900	0.56667500
N	1.42946200	-1.85833900	0.89659200
N	-0.10481100	-2.34466800	-0.57895300
C	2.14319900	-1.38325600	2.10200900
C	1.32922600	-0.41005100	2.96660500
C	2.61504900	-2.57679300	2.95705400
H	3.02409900	-0.84205400	1.72402700
C	0.24732600	-1.05567700	3.84358000
H	0.88881800	0.36941500	2.33239000
H	2.06501000	0.09446600	3.61465600
C	1.47032600	-3.24766400	3.71850200
H	3.14633700	-3.31197700	2.33785900
H	3.35337500	-2.18413900	3.67737900
C	0.77308300	-2.24819100	4.64376700
H	-0.58868100	-1.39906800	3.20964500
H	-0.17433900	-0.28969200	4.51479400
H	1.85721000	-4.10651800	4.29039400
H	0.73932500	-3.65530000	2.99748400
H	-0.05158000	-2.73246800	5.19132100
H	1.49454000	-1.89794200	5.40514800
C	-1.45745700	-2.43596400	-1.14608900

C	-1.54502000	-2.07103900	-2.62983900
C	-2.10736200	-3.78963300	-0.84511800
H	-2.03941000	-1.68447400	-0.59415300
C	-3.01072200	-2.07376300	-3.08317300
H	-0.96772800	-2.77988300	-3.24475000
H	-1.09453900	-1.07982300	-2.78849000
C	-3.57304400	-3.76744100	-1.28927400
H	-1.55894800	-4.59770700	-1.36180900
H	-2.03284900	-3.98168200	0.23668100
C	-3.69616800	-3.40955100	-2.77431800
H	-3.06732200	-1.85004900	-4.16103000
H	-3.54664700	-1.26511200	-2.56062600
H	-4.10391300	-3.01502200	-0.68070700
H	-4.04651800	-4.74123700	-1.08497200
H	-3.23246700	-4.20837100	-3.38287400
H	-4.75667900	-3.36592000	-3.07115200
C	3.36838800	-3.08542000	-0.22839100
C	1.33638000	-3.83168400	-2.09035300
C	3.65546200	-3.89827800	-1.32483100
H	4.15387600	-2.77500900	0.45924300
C	2.65197400	-4.27482100	-2.23445400
H	0.57011100	-4.12002800	-2.80762900
H	4.68015900	-4.24094100	-1.48254000
H	2.90679500	-4.92034700	-3.07772500
C	-4.42841400	0.98943700	0.10683400
F	-4.10749600	0.82253400	2.43535700
F	-4.79750800	2.28106800	-0.05504300
F	-5.53466100	0.28487800	0.29984200
F	-3.87158700	0.59966300	-1.05664500

Z-3a

C	1.47483300	-0.33986000	0.76888900
C	0.45608000	-0.94481400	0.15349900
H	0.68039600	-1.63126200	-0.67107900
C	2.90502500	-0.55549400	0.38627900
C	3.54297500	0.67726100	-0.29843000
F	3.65667200	-0.81696200	1.49056100
F	3.43848400	1.74688200	0.50078700
F	4.83167600	0.46893000	-0.56197100
F	3.05076400	-1.59551700	-0.47306400
H	1.32158600	0.36873300	1.58768300
C	-0.98845300	-0.73546000	0.48174200
H	-1.44511300	-1.70362100	0.75432700
H	-1.09326400	-0.07784200	1.35938600
C	-1.77918700	-0.14345900	-0.70590400
H	-1.32745900	0.82149400	-0.98663900
H	-1.66978800	-0.80897200	-1.57844200
C	-3.24188600	0.04385100	-0.38330300
C	-3.70620300	1.24683900	0.16854200
C	-4.15939300	-0.99908300	-0.57964700
C	-5.05042600	1.40560300	0.51417300
H	-3.00459500	2.07141600	0.32425900
C	-5.50434500	-0.84522800	-0.23565100
H	-3.81500100	-1.94252900	-1.01327700
C	-5.95421900	0.35874700	0.31361400
H	-5.39418300	2.35212200	0.93861600
H	-6.20508900	-1.66738300	-0.40073400
H	-7.00643700	0.48190300	0.58073800
F	2.91019900	0.94361600	-1.44494200

INT1

C	-1.01183600	2.78183300	-0.67414500
C	0.36541700	2.86518600	-0.32749500
H	1.09389100	2.74275700	-1.13512000
Cu	-0.26654500	1.03273800	0.11827500
C	-1.41410100	2.59507200	-2.08917800
C	-2.89989100	2.21517000	-2.30131700
F	-1.22608800	3.73168100	-2.83913000
F	-3.69591100	3.05765400	-1.62478000
F	-3.22803700	2.27196700	-3.59169600
F	-3.16577800	0.97227800	-1.86903700
F	-0.66316600	1.62388200	-2.71330700
H	-1.75528000	3.29766200	-0.06076100
C	0.80973800	3.75971400	0.81132700
H	1.08348900	4.73952800	0.37583100
H	-0.05733500	3.96581100	1.46122200
C	1.95467200	3.26097200	1.69867600
H	2.17335300	4.04766600	2.44458800
H	1.60274200	2.38650000	2.26870200
C	3.24449600	2.87537400	1.00562400
C	3.63136100	3.41278500	-0.22982900
C	4.10178200	1.94877700	1.61941800
C	4.82500800	3.01650400	-0.84264300
H	2.99622900	4.14588800	-0.73149500
C	5.29508800	1.55413100	1.01600400
H	3.81353400	1.50590500	2.57542400
C	5.65861900	2.08225800	-0.22543000
H	5.10022100	3.43926000	-1.81248100
H	5.92721000	0.80825600	1.50172900
H	6.57864100	1.75344100	-0.71306100

C	3.06749700	-1.31658400	1.80849400
H	1.31178300	-0.55363000	2.49305200
C	3.59642500	-0.78801200	-0.55785800
H	2.29848500	0.47917300	-1.46640700
C	3.92968200	-1.43511800	0.67275400
C	3.42090900	-1.92928700	3.00717100
C	4.44411200	-0.91401500	-1.65405800
C	5.14603800	-2.18340100	0.77685900
C	4.61962100	-2.66642700	3.10179600
H	2.76154500	-1.83475300	3.87413500
C	5.63746100	-1.65466000	-1.54563700
H	4.18975600	-0.40825100	-2.58875900
C	5.46691500	-2.79746400	2.01980600
C	5.99175900	-2.27463600	-0.36300600
H	4.87610700	-3.13977200	4.05334700
H	6.29391000	-1.73161000	-2.41658700
H	6.39334800	-3.37016000	2.10257400
H	6.92172600	-2.84247000	-0.28697300
N	1.89819200	-0.57944900	1.66931300
N	2.43437100	-0.03306200	-0.60421400
B	1.51254700	0.13511700	0.48504800
C	-2.69634600	-2.32569300	-0.16518000
C	-3.33165700	-1.66298400	0.91634100
C	-1.60665500	-0.38457900	0.20719400
N	-2.61835100	-0.47513700	1.11312100
N	-1.64434800	-1.49839400	-0.56795400
C	-2.81475100	0.65208200	2.04868800
C	-4.07724500	1.48811700	1.77117800
C	-2.63812900	0.30255100	3.53822500
H	-1.96248900	1.29075100	1.78223500

C	-5.33709800	1.03920400	2.51708700
H	-4.24328100	1.53877000	0.68518900
H	-3.83993900	2.51703700	2.09399800
C	-3.91062300	-0.10215000	4.29141000
H	-1.85300800	-0.46356400	3.63888000
H	-2.24116800	1.21547100	4.01521300
C	-5.06688800	0.86790000	4.01715900
H	-5.72055800	0.10022500	2.08890000
H	-6.13284400	1.78531700	2.36053500
H	-3.69193100	-0.13087100	5.37124300
H	-4.20478300	-1.12806600	4.02397100
H	-5.97839500	0.52842400	4.53530800
H	-4.81522000	1.85466000	4.44776200
C	-0.62689700	-1.65021400	-1.63516800
C	0.40868800	-2.75495300	-1.36269200
C	-1.18941500	-1.67497500	-3.06840500
H	-0.08641600	-0.70330500	-1.53818200
C	0.07496600	-4.13106300	-1.94491400
H	0.60672900	-2.80463400	-0.28133400
H	1.35048100	-2.40789100	-1.81847500
C	-1.47294200	-3.06142000	-3.65767300
H	-2.07347500	-1.02344400	-3.12127100
H	-0.42146400	-1.19187200	-3.69593600
C	-0.30615000	-4.02972800	-3.42693400
H	-0.73952900	-4.60634300	-1.37527900
H	0.94854100	-4.79175700	-1.82416400
H	-1.66661500	-2.95564400	-4.73764300
H	-2.39936300	-3.47910700	-3.23435700
H	-0.55184300	-5.02568200	-3.83040500
H	0.57258000	-3.67258200	-3.99476900

C	-4.44184800	-2.23373400	1.54583200
C	-3.16319300	-3.56458400	-0.61358100
C	-4.90288100	-3.46802400	1.08728900
H	-4.94360000	-1.73900800	2.37029900
C	-4.26994300	-4.12625200	0.02278200
H	-2.68188800	-4.08586500	-1.43385700
H	-5.76993000	-3.92464600	1.56892300
H	-4.64570600	-5.09344400	-0.31711600

TS1

C	-0.43795100	2.44615900	-1.89110600
C	-1.44061900	1.36539600	-1.75389400
H	-2.23603600	1.60484700	-1.04298400
Cu	0.37229700	0.80130000	-1.05522700
C	-0.57436400	3.56731400	-0.95697100
C	0.62698000	4.53457600	-0.91297200
F	-1.68232000	4.37130600	-1.18285500
F	0.89656600	5.00325100	-2.13746600
F	0.38294000	5.57559700	-0.11000000
F	1.72959800	3.92156000	-0.45274700
F	-0.76080400	3.12350600	0.35426100
H	-0.12945500	2.74289800	-2.89654900
C	-2.02128800	0.85270400	-3.06836900
H	-2.43382200	1.74461100	-3.57340400
H	-1.21199100	0.50012800	-3.73066300
C	-3.11866200	-0.22596400	-2.99845400
H	-3.61580400	-0.25683000	-3.98470900
H	-2.67221100	-1.21928600	-2.85631300
C	-4.15659600	-0.01728600	-1.91521700
C	-4.69319600	1.25240400	-1.64498000

C	-4.58144400	-1.09578100	-1.12737400
C	-5.60505600	1.43747400	-0.60328300
H	-4.38231900	2.11462300	-2.24007700
C	-5.48880300	-0.91563100	-0.08104600
H	-4.17654000	-2.09141800	-1.31780400
C	-5.99991100	0.35498500	0.18895700
H	-6.00111200	2.43631200	-0.40317900
H	-5.77350000	-1.76816900	0.53956200
H	-6.69721800	0.50327600	1.01684300
C	-1.90324100	-2.71892600	-0.00945600
H	-0.80338700	-2.11614000	-1.61279300
C	-2.48514000	-0.92299200	1.60650200
H	-1.85749200	0.94552300	1.08075500
C	-2.52797300	-2.29589000	1.20584200
C	-1.96803800	-4.05340700	-0.39322500
C	-3.11077900	-0.52809300	2.78353600
C	-3.22731300	-3.24963400	2.01171100
C	-2.65200800	-4.98975900	0.41159700
H	-1.49110400	-4.36963300	-1.32466800
C	-3.79803400	-1.47308900	3.57143300
H	-3.08284600	0.52355400	3.07832200
C	-3.26949600	-4.60689700	1.58448800
C	-3.86186700	-2.80280400	3.20354400
H	-2.69183900	-6.03410900	0.09135400
H	-4.29049600	-1.13907600	4.48838200
H	-3.80069400	-5.33706100	2.19892500
H	-4.39851200	-3.52732100	3.81992000
N	-1.25407000	-1.75983900	-0.78104200
N	-1.82361700	-0.02354300	0.78468900
B	-1.17108500	-0.37462300	-0.43579100

C	3.46167700	-0.49178500	1.31828900
C	3.71614500	-1.26699700	0.15752300
C	1.94173300	0.07634000	-0.25395600
N	2.75444200	-0.88410100	-0.78305100
N	2.36728200	0.32203100	1.01567700
C	2.45496300	-1.37913200	-2.14125200
C	3.54428700	-1.10255200	-3.19468800
C	1.93229300	-2.82922400	-2.17893800
H	1.60522100	-0.73664900	-2.41146900
C	4.58719500	-2.20831500	-3.38990900
H	4.01900700	-0.13405000	-2.97410000
H	3.01086800	-0.97173800	-4.15210100
C	2.99809000	-3.91330800	-2.37004900
H	1.33036100	-3.01640800	-1.27666800
H	1.24009700	-2.88329300	-3.03855600
C	3.93003600	-3.58548800	-3.54263000
H	5.29946400	-2.21847900	-2.55111900
H	5.18829000	-1.97693200	-4.28411100
H	2.49838000	-4.88024100	-2.54265700
H	3.58051400	-4.04045000	-1.44495900
H	4.70026900	-4.36646500	-3.64846700
H	3.34357900	-3.59976200	-4.47989000
C	1.60722800	1.29549900	1.83403100
C	0.75006800	0.64135300	2.93224700
C	2.40810100	2.51266300	2.33339100
H	0.91109100	1.69451900	1.08985200
C	1.43031300	0.50171800	4.29612500
H	0.36467300	-0.32195600	2.56840900
H	-0.13311800	1.29102100	3.05862100
C	3.02239700	2.40262600	3.73393800

H	3.16282100	2.78506900	1.58074800
H	1.68637000	3.34624500	2.34378200
C	2.02838700	1.83655800	4.75540700
H	2.21281000	-0.27303200	4.25729100
H	0.69054700	0.14367200	5.03022100
H	3.35889300	3.40297800	4.05160200
H	3.93230100	1.78343900	3.71226900
H	2.51472800	1.72421000	5.73817100
H	1.20699000	2.56296200	4.89628800
C	4.76839000	-2.18684200	0.13442500
C	4.25212700	-0.64148700	2.46096400
C	5.55329700	-2.32676900	1.27985500
H	4.97990400	-2.78521900	-0.74500100
C	5.29740400	-1.56512200	2.42875100
H	4.06346800	-0.06081600	3.35728600
H	6.37724800	-3.04320200	1.27596800
H	5.92235400	-1.69311100	3.31486000

NHC-Cu-Bdan

Cu	-0.41450000	-0.00020000	-0.00102100
C	3.70606400	-0.70857300	-0.02085200
C	3.70585700	0.70931000	0.02012400
C	1.55651100	0.00001900	-0.00125700
N	2.36289100	1.09645000	0.03034700
N	2.36326800	-1.09610100	-0.03229800
C	1.72277400	2.43195000	0.06785800
C	1.94640400	3.21306600	1.37559600
C	1.95324400	3.28904500	-1.19030100
H	0.66080500	2.14782000	0.05649800
C	3.15617500	4.15347600	1.39590700

H	1.97175800	2.50550400	2.21891200
H	1.03944200	3.82568000	1.51772700
C	3.16188400	4.23032500	-1.14876600
H	1.98417800	2.63263200	-2.07387400
H	1.04643400	3.90838300	-1.30095500
C	3.19637100	5.04631000	0.14945200
H	4.09046400	3.57755500	1.48174000
H	3.10912000	4.77716400	2.30335000
H	3.11754000	4.90763000	-2.01708000
H	4.09723400	3.66172200	-1.26568900
H	4.08839200	5.69325600	0.17089500
H	2.32232300	5.72285700	0.16797100
C	1.72375500	-2.43185800	-0.06970600
C	1.94960600	-3.21414100	-1.37642600
C	1.95267200	-3.28748000	1.18978600
H	0.66166500	-2.14813900	-0.06024800
C	3.15886900	-4.15531300	-1.39390700
H	1.97692400	-2.50723900	-2.22023400
H	1.04260600	-3.82638300	-1.51984300
C	3.16142800	-4.22871900	1.15075600
H	1.98257900	-2.62991600	2.07253000
H	1.04578000	-3.90672300	1.30022500
C	3.19708300	-5.04652100	-0.14626000
H	4.09365200	-3.58013800	-1.47935000
H	3.11255100	-4.78012400	-2.30061600
H	3.11633700	-4.90476400	2.02001300
H	4.09664200	-3.65986600	1.26763300
H	4.08882900	-5.69390500	-0.16576200
H	2.32274000	-5.72268600	-0.16490100
C	4.91079100	1.42027700	0.04162400

C	4.91115000	-1.41930100	-0.04147700
C	6.10504300	0.70210000	0.02116200
H	4.92833100	2.50411500	0.07364300
C	6.10522900	-0.70086800	-0.02010700
H	4.92889300	-2.50313500	-0.07347500
H	7.05285400	1.24394100	0.03757100
H	7.05318200	-1.24248200	-0.03580800
C	-4.63508000	-0.19283400	1.22459500
H	-2.80201600	-0.32671500	2.07706100
C	-4.63536800	0.19239700	-1.22450100
H	-2.80248600	0.32618400	-2.07738500
C	-5.35054300	-0.00020300	0.00014200
C	-5.34443200	-0.37931300	2.41016700
C	-5.34503900	0.37889500	-2.40988300
C	-6.78287800	-0.00017500	0.00032700
C	-6.75354900	-0.37810800	2.40251400
H	-4.79756000	-0.52631900	3.34547500
C	-6.75415200	0.37774800	-2.40186100
H	-4.79841000	0.52586800	-3.34534000
C	-7.46556800	-0.19415900	1.23333100
C	-7.46588500	0.19383400	-1.23249800
H	-7.28757800	-0.52638300	3.34528700
H	-7.28841500	0.52604200	-3.34450000
N	-3.24977800	-0.18607800	1.18112000
N	-3.25008000	0.18557200	-1.18135800
B	-2.43852400	-0.00027500	-0.00018200
H	-8.55797300	-0.19491500	1.23837900
H	-8.55829000	0.19463700	-1.23726400

TS2Z

C	-0.66647900	2.73370700	0.44146400
C	0.50073700	2.89680200	-0.53539200
H	0.06727800	2.99886500	-1.54479900
Cu	-1.32040100	0.70143900	0.57488700
C	-1.94800900	3.14298800	0.09403800
F	-2.97089400	1.70903400	-0.38089100
H	-0.43299400	2.78881500	1.51132000
C	1.29476700	4.18338800	-0.23754300
H	0.58533200	5.02683500	-0.19328800
H	1.73940000	4.11818900	0.77321600
C	2.40594300	4.51327000	-1.25379000
H	1.95881400	4.60346300	-2.25762800
H	2.82746600	5.50126700	-1.00455400
C	3.50643900	3.47981500	-1.27804300
C	3.55147400	2.48871600	-2.26718600
C	4.44694200	3.42112100	-0.23656300
C	4.47608300	1.44201300	-2.19810500
H	2.83336500	2.52276900	-3.08990800
C	5.37691500	2.38327500	-0.16640300
H	4.43613400	4.19409000	0.53798000
C	5.38509700	1.38059200	-1.14162700
H	4.47180100	0.66203600	-2.96277300
H	6.09320700	2.34720100	0.65817400
H	6.09236200	0.55152100	-1.07206400
C	2.81683200	-0.05947500	0.65160600
H	2.45592500	1.85183300	1.25548200
C	1.67561700	-0.73274300	-1.45250500
H	0.49466900	0.70818500	-2.28677000
C	2.54860300	-1.03518100	-0.35938600

C	3.67119200	-0.37214000	1.70325900
C	1.43702200	-1.69160600	-2.42997800
C	3.16494000	-2.32494400	-0.28248600
C	4.26598300	-1.64825600	1.77905800
H	3.87559000	0.37946200	2.46906700
C	2.05055100	-2.95770100	-2.34768600
H	0.76515600	-1.45916600	-3.25811900
C	4.02260300	-2.60932900	0.81729400
C	2.89478700	-3.27509900	-1.30432200
H	4.93137900	-1.87321400	2.61659300
H	1.83800900	-3.69969200	-3.12036900
H	4.49094900	-3.59399300	0.88056700
H	3.35496500	-4.26300100	-1.24168900
N	2.19053800	1.17313700	0.55392000
N	1.09302200	0.52543000	-1.49141200
B	1.30337300	1.53308000	-0.50284600
C	-1.16340400	-3.17076600	-0.62616800
C	-0.27478300	-3.30663100	0.46005900
C	-1.16681100	-1.22743700	0.55096000
N	-0.32887500	-2.10287200	1.16818700
N	-1.70686700	-1.89439200	-0.51082200
C	0.39995700	-1.91157800	2.44033700
C	0.36862200	-0.47229500	2.97212100
C	-0.13067700	-2.88412500	3.51292600
H	1.44705900	-2.16324300	2.21430200
C	-0.94104200	-0.04661300	3.64906700
H	0.62264300	0.22450000	2.16375900
H	1.19049700	-0.41209700	3.70511500
C	-1.49429500	-2.46461700	4.06546500
H	-0.17358300	-3.90847100	3.11739700

H	0.60866400	-2.89611800	4.33237100
C	-1.42577800	-1.06536500	4.68178700
H	-1.72476000	0.08749600	2.88518900
H	-0.80020600	0.94316300	4.11353100
H	-1.83780100	-3.20293100	4.80800000
H	-2.23870200	-2.47175700	3.24931700
H	-2.40904000	-0.76593100	5.07928000
H	-0.73091700	-1.08479100	5.54189500
C	-2.73659900	-1.26494400	-1.34782500
C	-2.20417200	-0.79171600	-2.70117000
C	-4.00870500	-2.10452600	-1.48710200
H	-3.01336000	-0.35649100	-0.79620600
C	-3.28266100	0.03041800	-3.41381700
H	-1.88672100	-1.65129000	-3.31774600
H	-1.31719200	-0.17023200	-2.51646900
C	-5.08366800	-1.27892500	-2.20563500
H	-3.81183300	-3.02693100	-2.05806400
H	-4.35491900	-2.41284100	-0.48744200
C	-4.58473500	-0.76443400	-3.56125300
H	-2.91933400	0.36323600	-4.39985100
H	-3.46307400	0.93384300	-2.80703600
H	-5.35424100	-0.41723900	-1.57103300
H	-5.99766400	-1.88132400	-2.33327100
H	-4.41487600	-1.62409900	-4.23642200
H	-5.35893100	-0.14126200	-4.03769300
C	0.48822300	-4.46368800	0.63518400
C	-1.31477700	-4.19647200	-1.56463500
C	0.32489700	-5.48925500	-0.29564200
H	1.20763700	-4.55332500	1.44818200
C	-0.56245100	-5.35697500	-1.37837900

H	-1.98038000	-4.09299700	-2.42006700
H	0.91277300	-6.40357800	-0.19194400
H	-0.65745400	-6.17416800	-2.09665300
C	-2.91901900	3.60895800	1.17250800
F	-2.50682100	4.80490900	1.64616400
F	-2.92918900	2.75623800	2.21150300
F	-4.16116900	3.76744600	0.73384600
F	-2.14081800	3.80101900	-1.05311700
